# Supplementary figures and images for: Comparative study on the composition of four different varieties of garlic
Source: PeerJ. 2019 Feb 21;7:e6442. doi: 10.7717/peerj.6442 (PMC6387757; doi:10.7717/peerj.6442)

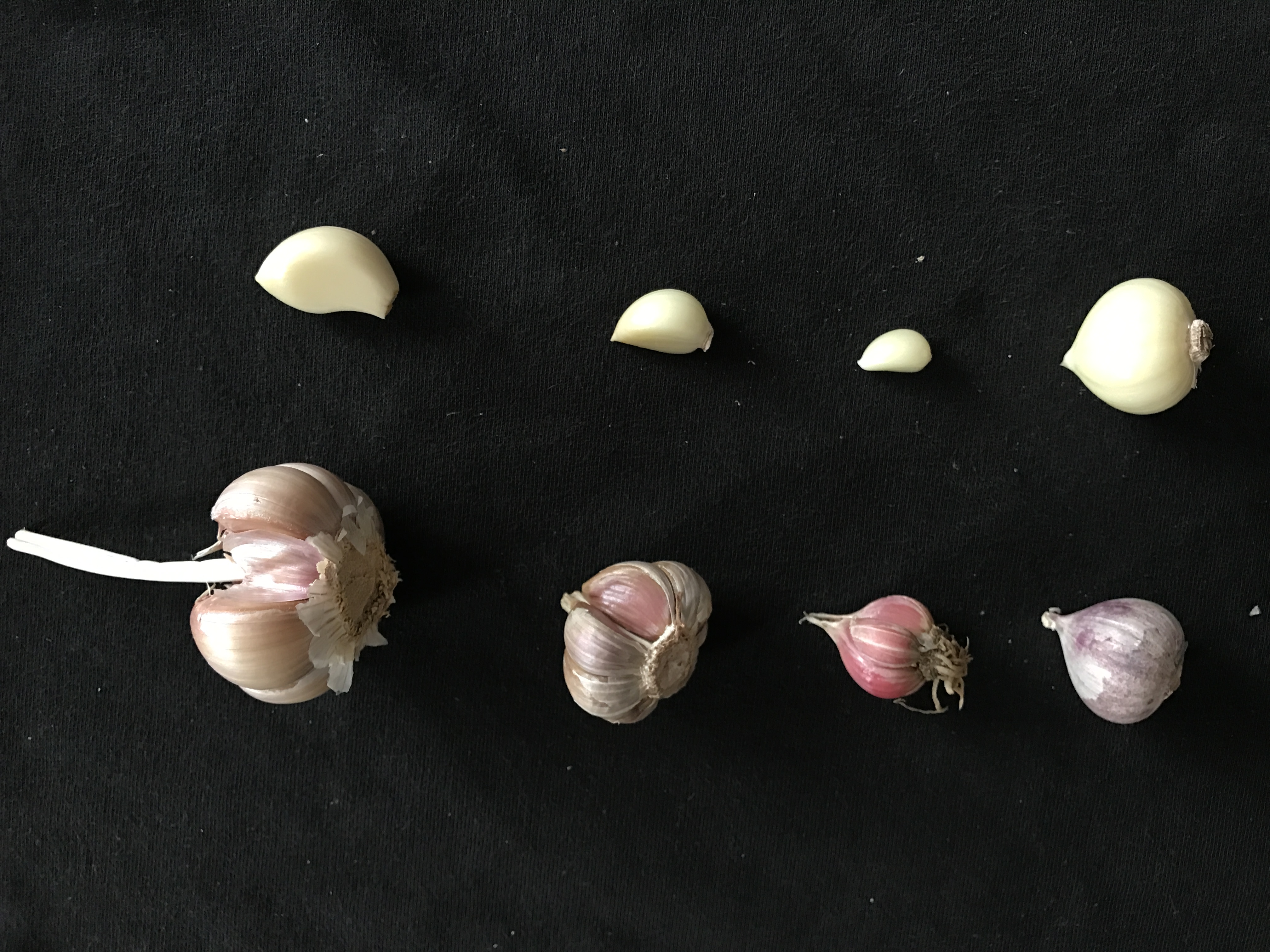

Supplement: Supplemental Information 1 [file peerj-07-6442-s001.jpg]

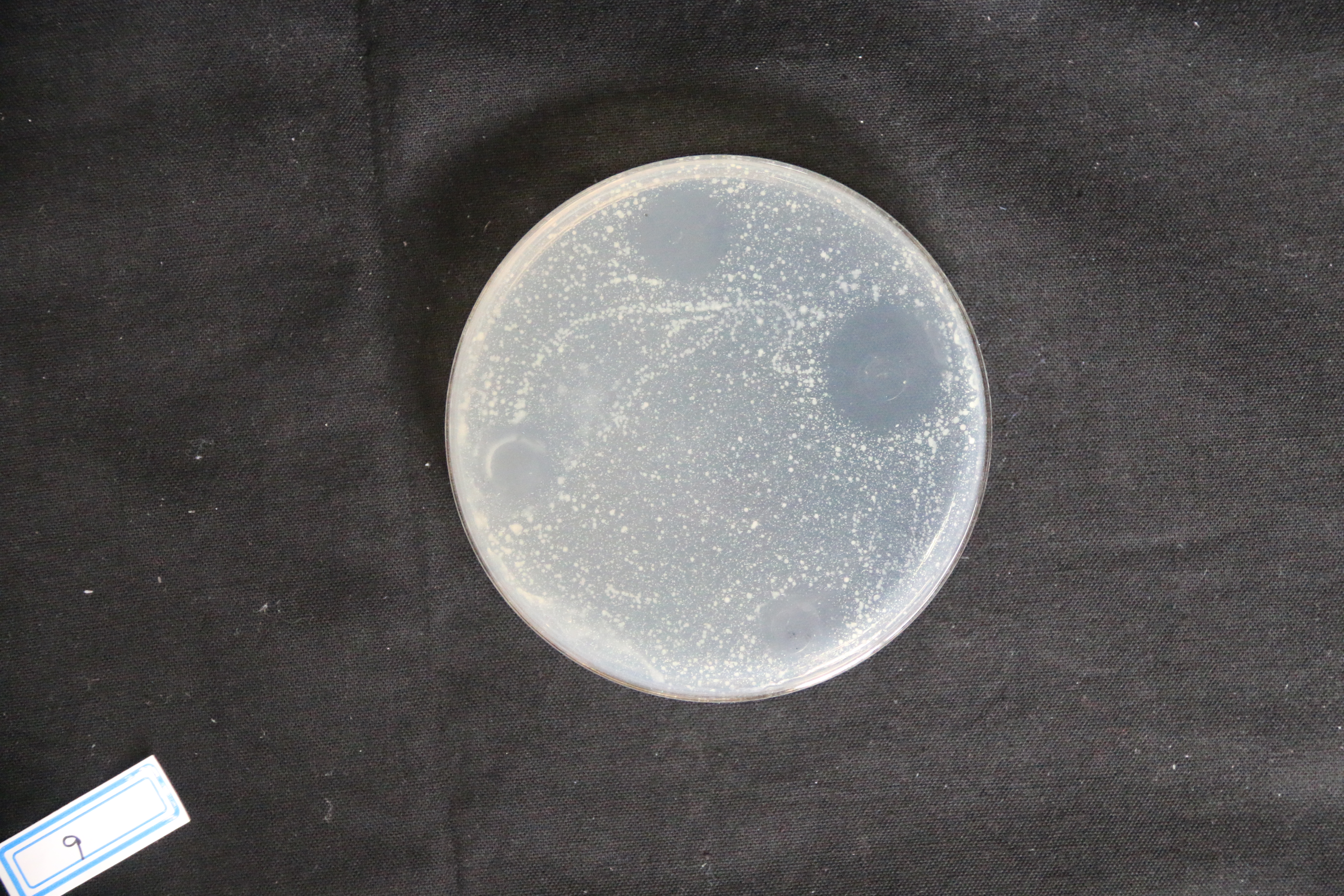

Supplement: Supplemental Information 6 [file peerj-07-6442-s006.jpg]

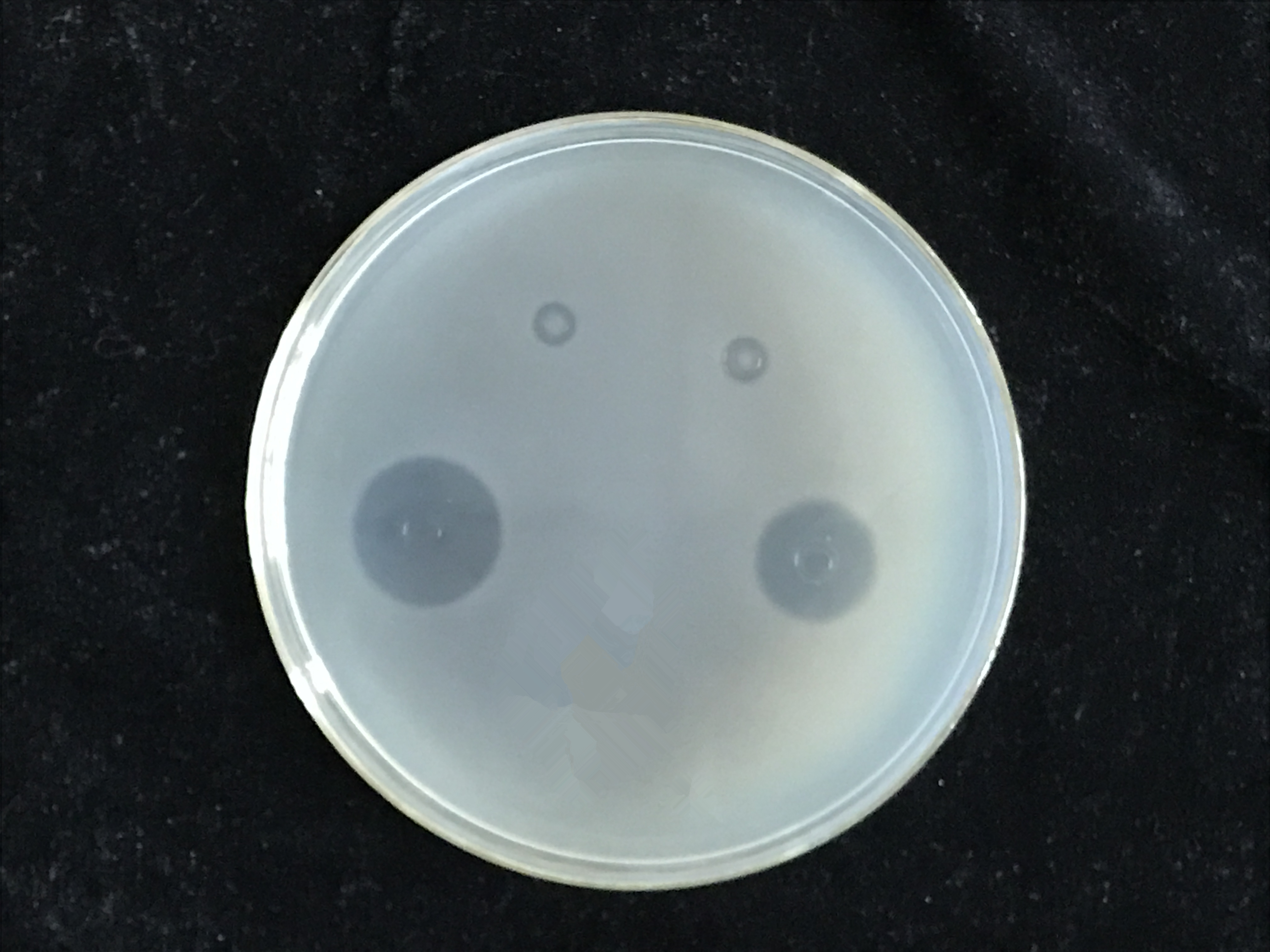

Supplement: Supplemental Information 7 [file peerj-07-6442-s007.jpg]

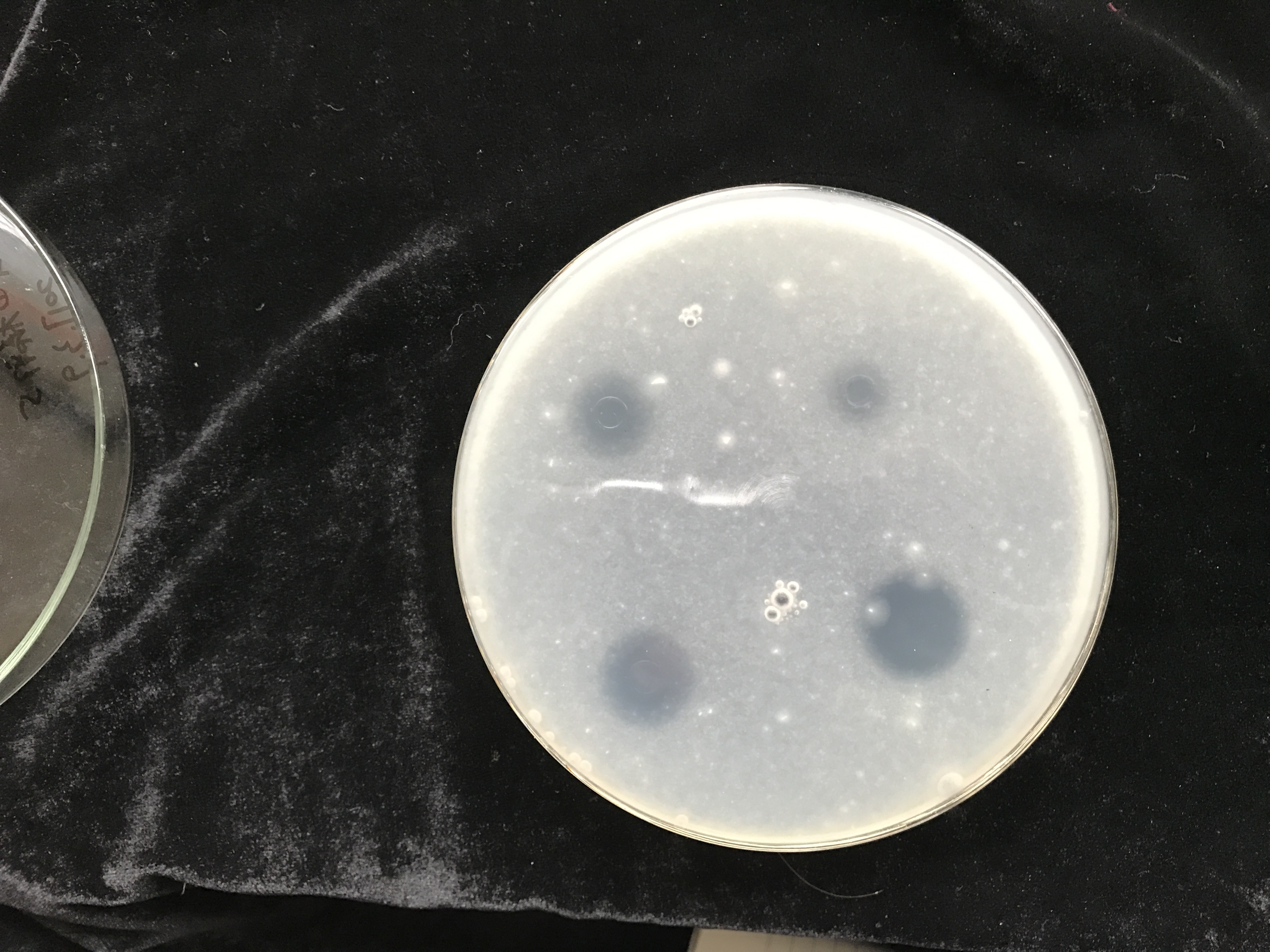

Supplement: Supplemental Information 8 [file peerj-07-6442-s008.jpg]

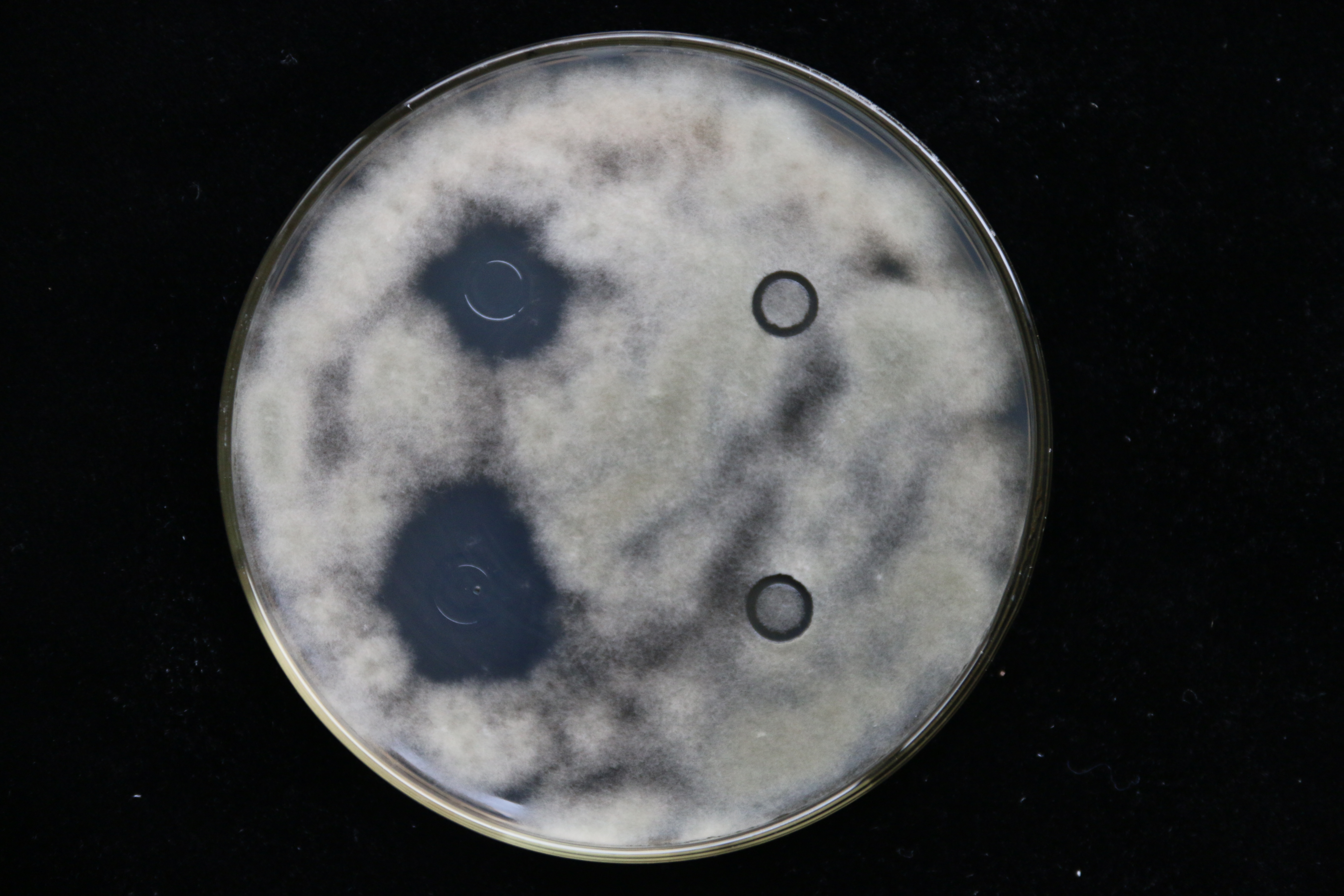

Supplement: Supplemental Information 9 [file peerj-07-6442-s009.jpg]
