# Supplementary material for: Comparative study on the composition of four different varieties of garlic
Source: PeerJ. 2019 Feb 21;7:e6442. doi: 10.7717/peerj.6442 (PMC6387757; doi:10.7717/peerj.6442)

Sample Information

Analyzed by	: Admin
Analyzed	: 2018-5-7 16:45:49
Sample Type	: Unknown
Level #	: 1
Sample Name	: 2018-5-7-1-2
Sample ID	:
IS Amount	: [1]=1.000
Sample Amount	: 1.000
Dilution Factor	: 1.000
Vial #	: 1
Injection Volume	: 1.000
Data File	: E:\陈存\GC-MS硫化物\2018-5-7-1-2.qgd
Org Data File	: E:\刘春宏\2018-5-7-1-2.qgd
Method File	: E:\刘春宏\2018-5-7-1.qgm
Org Method File	: E:\刘春宏\2018-5-7-1.qgm
Report File	:
Tuning File	: C:\GCMSsolution\System\Tune1\2017-3-21.qgt
Modified by	: Admin
Modified	: 2018-5-7 17:14:49

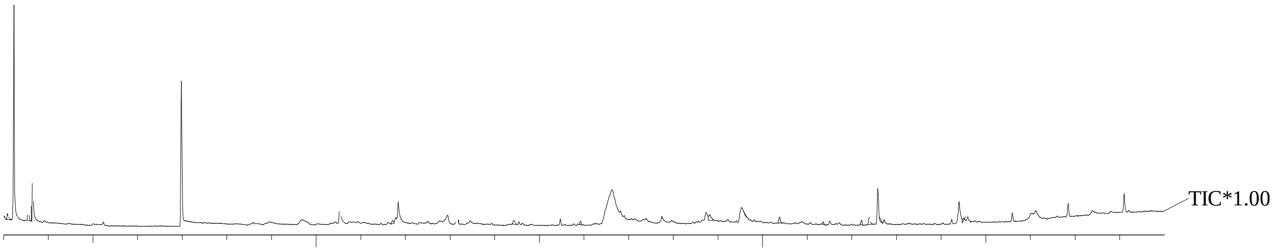

Peak Report TIC

Peak#	R.Time	I.Time	F.Time	Area	Area%	Height	Height%	A/H	Mark	Name
1	3.086	3.067	3.167	6197082	22.40	3995037	39.27	1.55		
2	3.544	3.517	3.617	535090	1.93	330896	3.25	1.61		
3	6.982	6.942	7.050	4717647	17.05	2594017	25.50	1.81		
4	11.708	11.675	11.750	161011	0.58	60734	0.60	2.65		
5	11.840	11.808	11.942	1091518	3.94	374763	3.68	2.91	V	
6	12.943	12.892	13.025	316829	1.14	110250	1.08	2.87		
7	14.426	14.383	14.492	204120	0.74	66653	0.66	3.06		
8	15.923	15.892	15.967	103541	0.37	61036	0.60	1.69		
9	16.626	16.392	16.783	7566252	27.34	574594	5.65	13.16		
10	16.825	16.783	16.883	654212	2.36	153466	1.51	4.26	V	
11	18.825	18.783	18.917	391361	1.41	93736	0.92	4.17	V	
12	19.528	19.450	19.650	1398141	5.05	231960	2.28	6.02		
13	20.381	20.342	20.450	231609	0.84	109224	1.07	2.12		
14	21.358	21.325	21.408	81332	0.29	50445	0.50	1.61		
15	22.216	22.183	22.258	147412	0.53	84145	0.83	1.75		
16	22.390	22.350	22.458	333099	1.20	134552	1.32	2.47		
17	22.584	22.458	22.692	1475557	5.33	643402	6.32	2.29	V	
18	24.401	24.275	24.475	1397061	5.05	394604	3.88	3.54	V	
19	24.594	24.475	24.650	674069	2.44	109132	1.07	6.17	V	
				27676943	100.00	10172646	100.00			

Library

<< Target >>

Line#:1 R.Time:3.083(Scan#:11) MassPeaks:246 BasePeak:46.00(39340)

RawMode:Averaged 3.075-3.092(10-12) BG Mode:Calc. from Peak

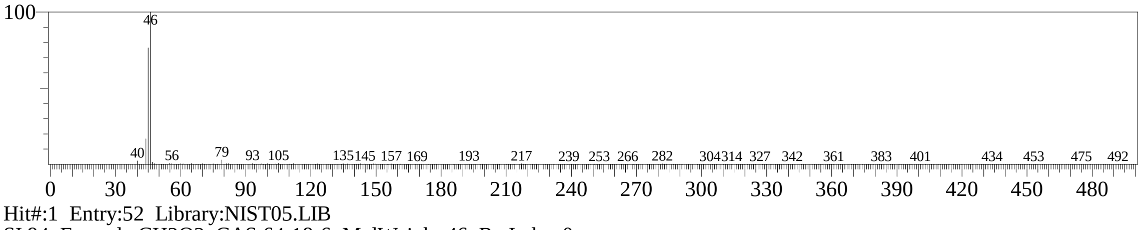

Hit#:1 Entry:52 Library:NIST05.LIB

SI:94 Formula:CH2O2 CAS:64-18-6 MolWeight:46 RetIndex:0

CompName:Formic acid $$ Methanoic acid $$ Aminic acid $$ Bilorin $$ Collo-Bueglatt $$ Collo-Didax $$ Formisoton $$ Formyl

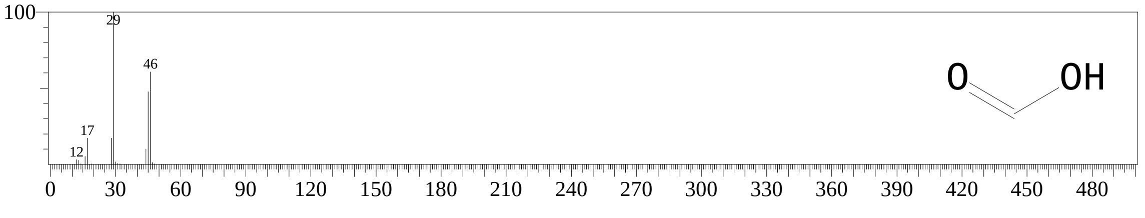

<< Target >>
Line#:1   R.Time:3.083(Scan#:11) MassPeaks:246   BasePeak:46.00(39340)
RawMode:Averaged 3.075-3.092(10-12)   BG Mode:Calc. from Peak

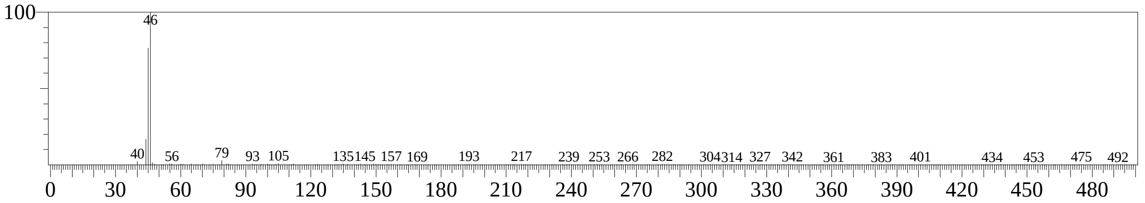

Hit#:2 Entry:41 Library:NIST05s.LIB
SI:93 Formula:CH2O2 CAS:64-18-6 MolWeight:46 RetIndex:0
CompName:Formic acid $$ Methanoic acid $$ Aminic acid $$ Bilorin $$ Collo-Bueglatt $$ Collo-Didax $$ Formisoton $$ Formyl

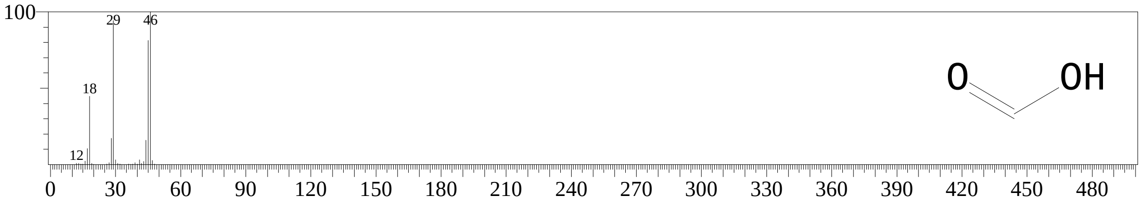

Hit#:3 Entry:42 Library:NIST05s.LIB
SI:92 Formula:CH2O2 CAS:64-18-6 MolWeight:46 RetIndex:0
CompName:Formic acid $$ Methanoic acid $$ Aminic acid $$ Bilorin $$ Collo-Bueglatt $$ Collo-Didax $$ Formisoton $$ Formyl

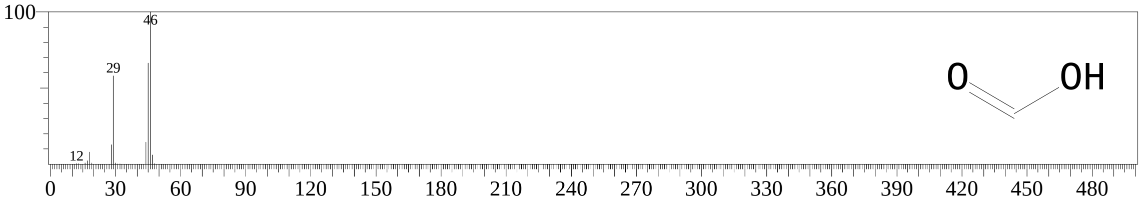

Hit#:4 Entry:55 Library:NIST05.LIB
SI:92 Formula:C2H3F CAS:75-2-5 MolWeight:46 RetIndex:183
CompName:Ethene, fluoro- $$ Ethylene, fluoro- $$ Fluoroethene $$ Fluoroethylene $$ Monofluoroethylene $$ Vinyl fluoride $$ C2

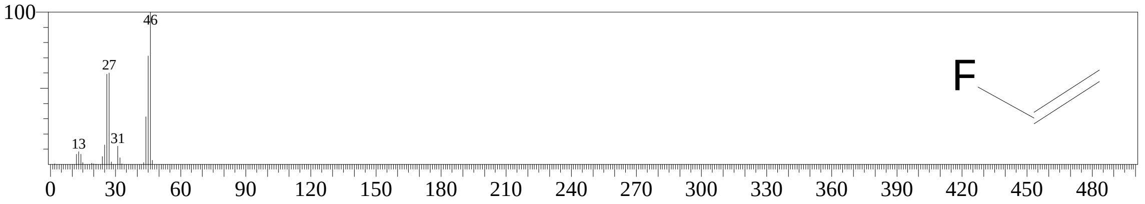

Hit#:5 Entry:53 Library:NIST05.LIB
SI:89 Formula:CH6N2 CAS:60-34-4 MolWeight:46 RetIndex:0
CompName:Hydrazine, methyl- $$ Methylhydrazine $$ Monomethylhydrazine $$ MMH $$ CH3NHNH2 $$ Hydrazomethane $$ 1

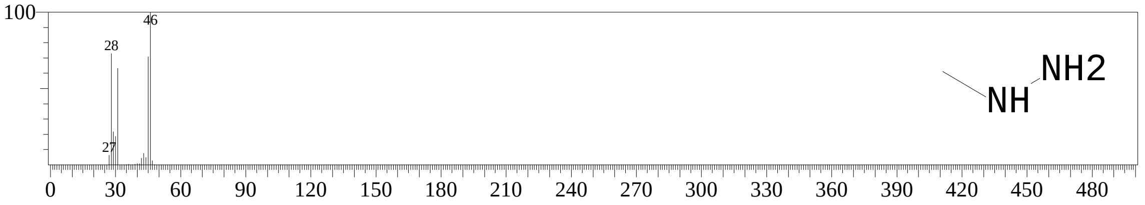

<< Target >>
Line#:2   R.Time:3.233(Scan#:29) MassPeaks:272   BasePeak:43.00(1013023)
RawMode:Averaged 3.225-3.242(28-30)   BG Mode:Calc. from Peak

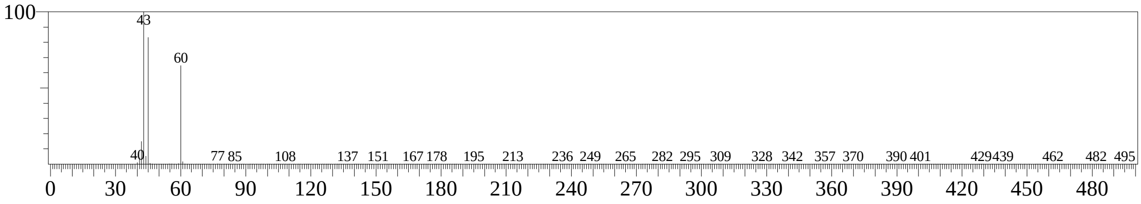

Hit#:1 Entry:133 Library:NIST05s.LIB
SI:98 Formula:C2H4O2 CAS:64-19-7 MolWeight:60 RetIndex:576
CompName:Acetic acid $$ Ethanoic acid $$ Ethylic acid $$ Glacial acetic acid $$ Methanecarboxylic acid $$ Vinegar acid $$ CH3

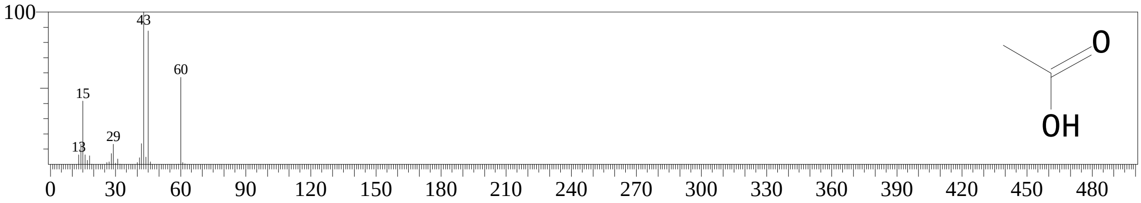

Hit#:2 Entry:134 Library:NIST05s.LIB
SI:98 Formula:C2H4O2 CAS:64-19-7 MolWeight:60 RetIndex:576
CompName:Acetic acid $$ Ethanoic acid $$ Ethylic acid $$ Glacial acetic acid $$ Methanecarboxylic acid $$ Vinegar acid $$ CH3

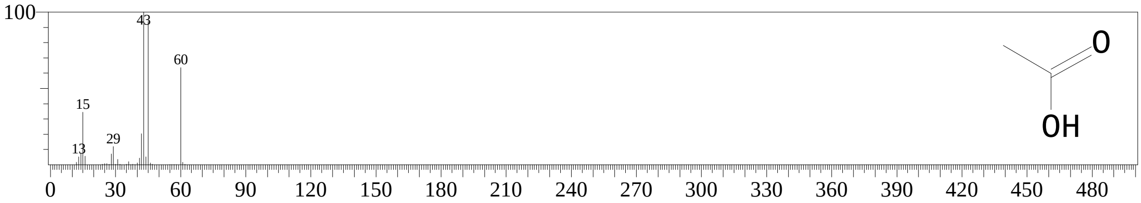

Hit#:3 Entry:455 Library:NIST05.LIB
SI:98 Formula:C2H7NO2 CAS:631-61-8 MolWeight:77 RetIndex:630
CompName:Ammonium acetate $$ Acetic acid, ammonium salt $$ Mindererus's spirit $$

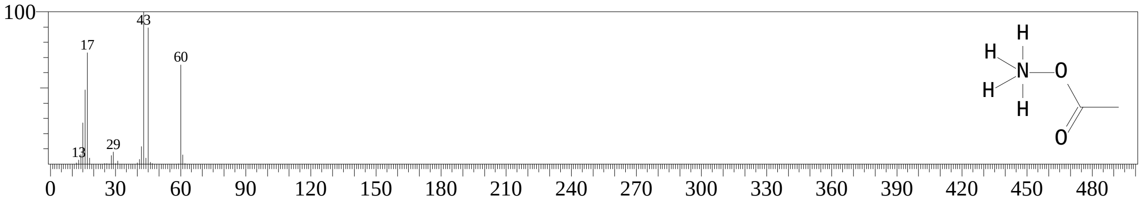

Hit#:4 Entry:136 Library:NIST05s.LIB
SI:98 Formula:C2H4O2 CAS:64-19-7 MolWeight:60 RetIndex:576
CompName:Acetic acid $$ Ethanoic acid $$ Ethylic acid $$ Glacial acetic acid $$ Methanecarboxylic acid $$ Vinegar acid $$ CH3

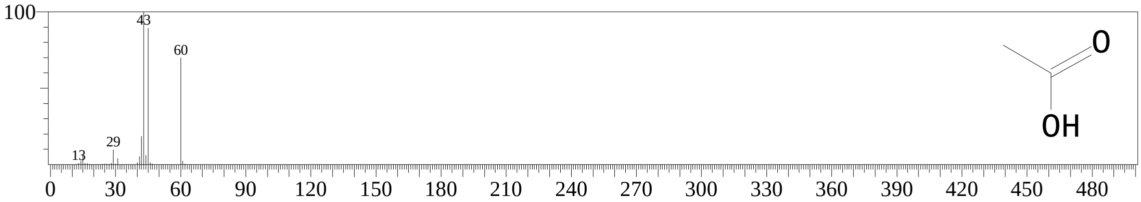

Hit#:5 Entry:133 Library:NIST05.LIB
SI:97 Formula:C2H4O2 CAS:64-19-7 MolWeight:60 RetIndex:576
CompName:Acetic acid $$ Ethanoic acid $$ Ethylic acid $$ Glacial acetic acid $$ Methanecarboxylic acid $$ Vinegar acid $$ CH3

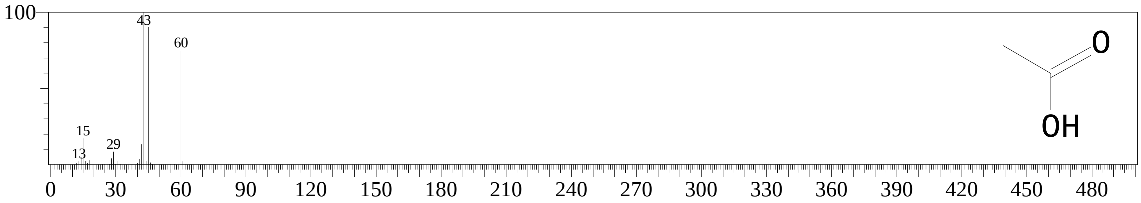

<< Target >>
Line#:3 R.Time:3.542(Scan#:66) MassPeaks:248 BasePeak:43.00(182256)
RawMode:Averaged 3.533-3.550(65-67) BG Mode:Calc. from Peak

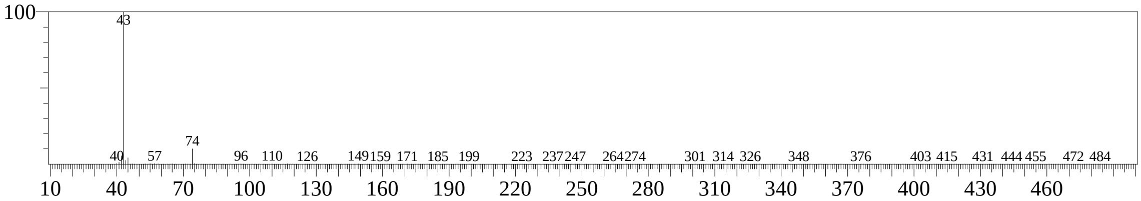

Hit#:1 Entry:415 Library:NIST05s.LIB
SI:98 Formula:C3H6O2 CAS:116-9-6 MolWeight:74 RetIndex:698
CompName:2-Propanone, 1-hydroxy- $$ Acetol $$ CH3C(O)CH2OH $$ Hydroxyacetone $$ Acetone alcohol $$ Acetylcarbinol $$

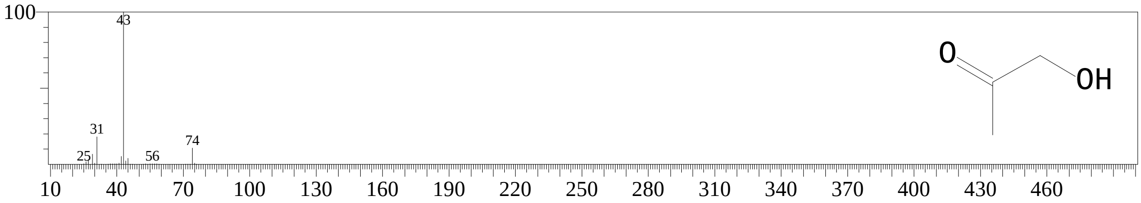

Hit#:2 Entry:368 Library:NIST05.LIB
SI:98 Formula:C3H6O2 CAS:116-9-6 MolWeight:74 RetIndex:698
CompName:2-Propanone, 1-hydroxy- $$ Acetol $$ CH3C(O)CH2OH $$ Hydroxyacetone $$ Acetone alcohol $$ Acetylcarbinol $$

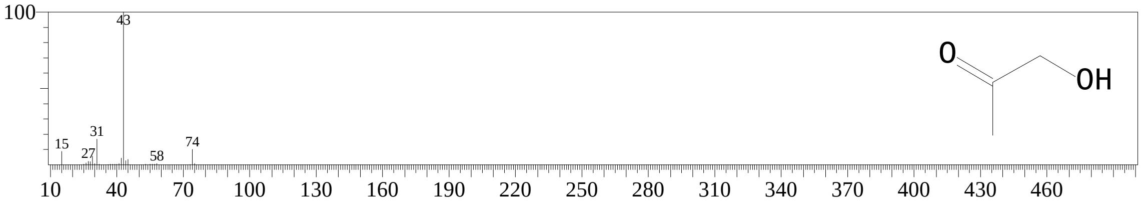

Hit#:3 Entry:414 Library:NIST05s.LIB
SI:96 Formula:C3H6O2 CAS:116-9-6 MolWeight:74 RetIndex:698
CompName:2-Propanone, 1-hydroxy- $$ Acetol $$ CH3C(O)CH2OH $$ Hydroxyacetone $$ Acetone alcohol $$ Acetylcarbinol $$

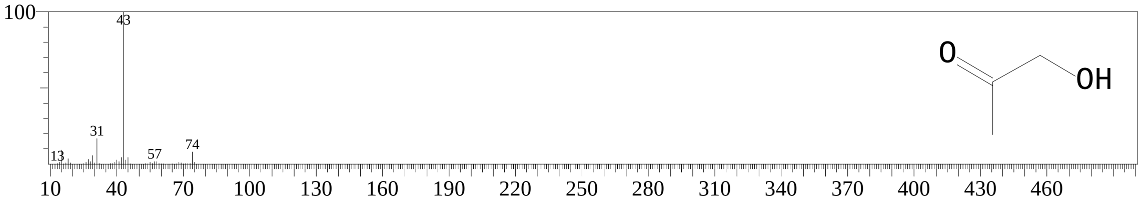

Hit#:4 Entry:369 Library:NIST05.LIB
SI:94 Formula:C3H6O2 CAS:79-20-9 MolWeight:74 RetIndex:487
CompName:Acetic acid, methyl ester $$ Methyl acetate $$ Devoton $$ Tereton $$ CH3COOCH3 $$ Methyl ethanoate $$ Acetate c

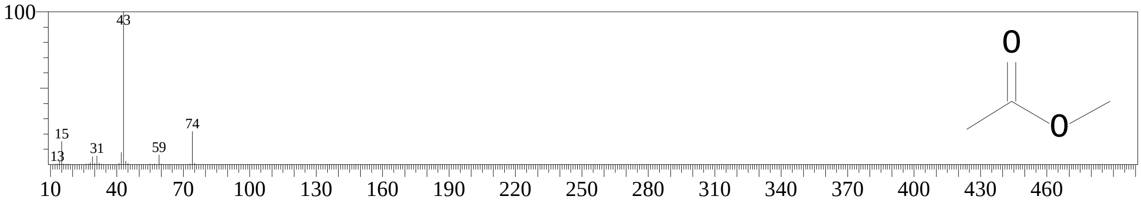

Hit#:5 Entry:4395 Library:NIST05.LIB
SI:93 Formula:C5H8O3 CAS:617-35-6 MolWeight:116 RetIndex:822
CompName:Propanoic acid, 2-oxo-, ethyl ester $$ Pyruvic acid, ethyl ester $$ Ethyl pyruvate $$ Ethyl 2-oxopropanoate $$ Ethyl 2-

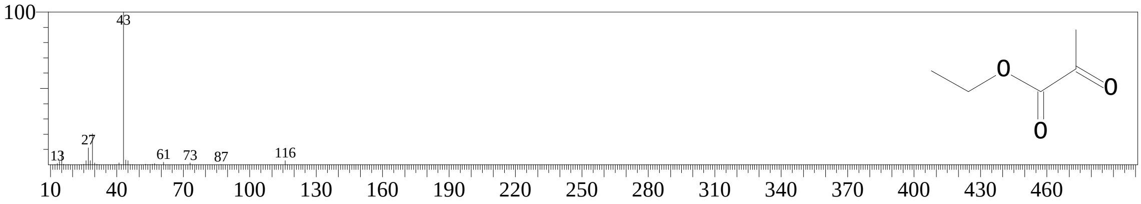

<< Target >>
Line#:4 R.Time:6.983(Scan#:479) MassPeaks:305 BasePeak:133.00(703371)
RawMode:Averaged 6.975-6.992(478-480) BG Mode:Calc. from Peak

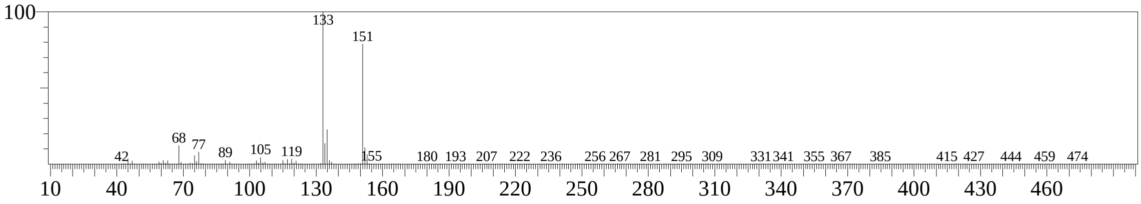

Hit#:1 Entry:15209 Library:NIST05.LIB
SI:85 Formula:C8H9NO2 CAS:0-0-0 MolWeight:151 RetIndex:1301
CompName:Oxime-, methoxy-phenyl-_

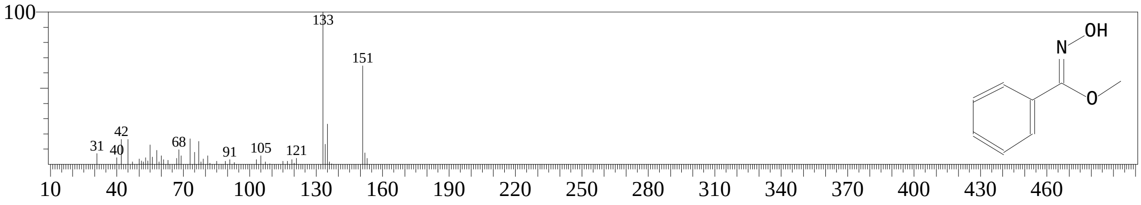

Hit#:2 Entry:15206 Library:NIST05.LIB
SI:79 Formula:C8H9NO2 CAS:2941-78-8 MolWeight:151 RetIndex:1575
CompName:2-Amino-5-methylbenzoic acid $$ 5-Methylanthranilic acid $$ Benzoic acid, 2-amino-5-methyl- $$ m-Toluic acid, 6-a

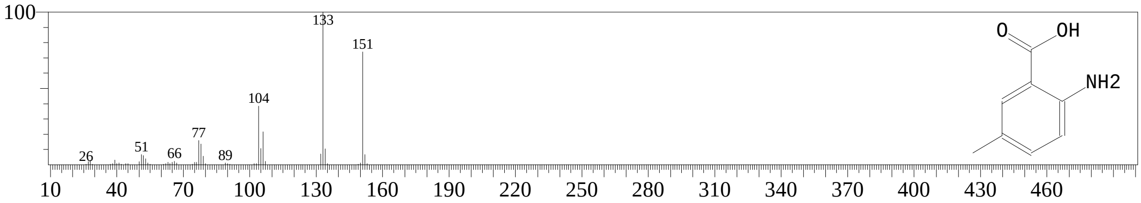

Hit#:3 Entry:51823 Library:NIST05.LIB
SI:79 Formula:C14H18O2 CAS:0-0-0 MolWeight:218 RetIndex:1714
CompName:4-Ethylbenzoic acid, cyclopentyl ester

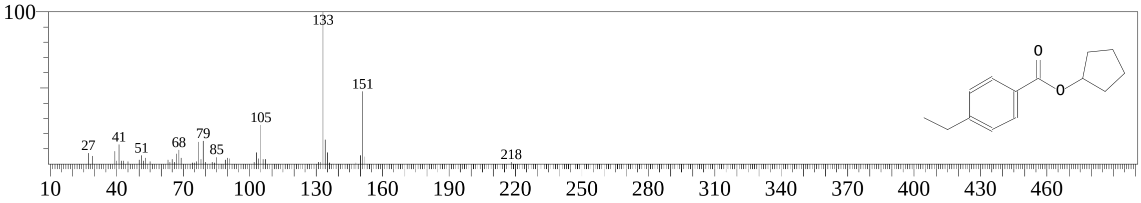

Hit#:4 Entry:61585 Library:NIST05.LIB
SI:78 Formula:C15H22O2 CAS:0-0-0 MolWeight:234 RetIndex:1770
CompName:4-Ethylbenzoic acid, hexyl ester

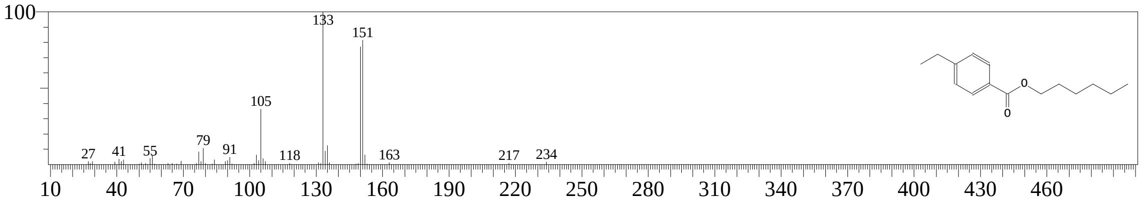

Hit#:5 Entry:15207 Library:NIST05.LIB
SI:76 Formula:C8H9NO2 CAS:2305-36-4 MolWeight:151 RetIndex:1575
CompName:Benzoic acid, 2-amino-4-methyl- $$ p-Toluic acid, 2-amino- $$ 2-Amino-4-methylbenzoic acid # $$

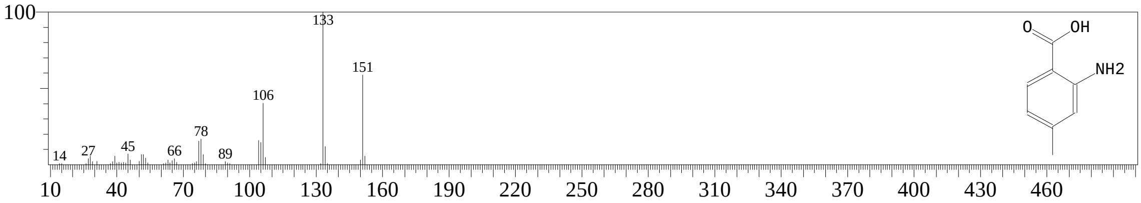

<< Target >>
Line#:5 R.Time:11.708(Scan#:1046) MassPeaks:229 BasePeak:43.00(19194)
RawMode:Averaged 11.700-11.717(1045-1047) BG Mode:Calc. from Peak

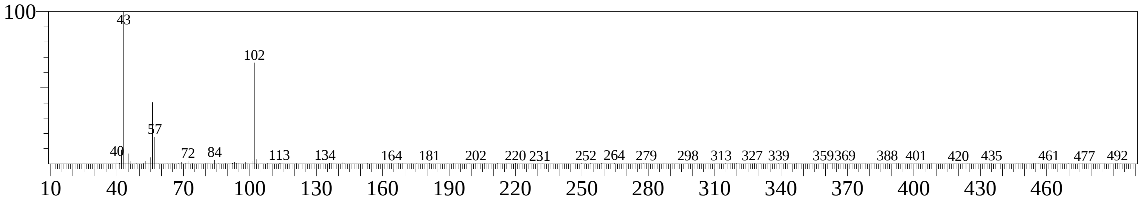

Hit#:1 Entry:3404 Library:NIST05s.LIB
SI:80 Formula:C5H8O3 CAS:123-76-2 MolWeight:116 RetIndex:1011
CompName:Pentanoic acid, 4-oxo- $$ Levulinic acid $$ .beta.-Acetylpropionic acid $$ .gamma.-Ketovaleric acid $$ Laevulinic aci

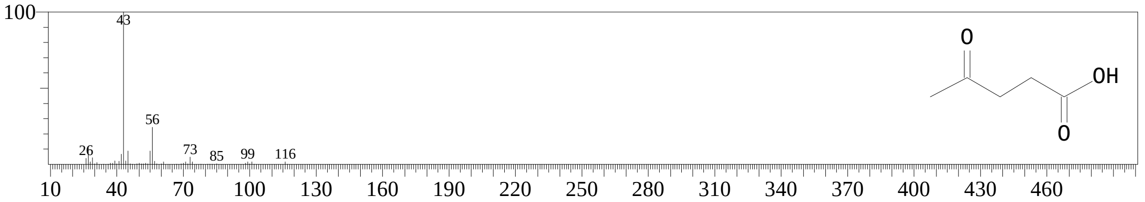

Hit#:2 Entry:4401 Library:NIST05.LIB
SI:80 Formula:C5H8O3 CAS:123-76-2 MolWeight:116 RetIndex:1011
CompName:Pentanoic acid, 4-oxo- $$ Levulinic acid $$ .beta.-Acetylpropionic acid $$ .gamma.-Ketovaleric acid $$ Laevulinic aci

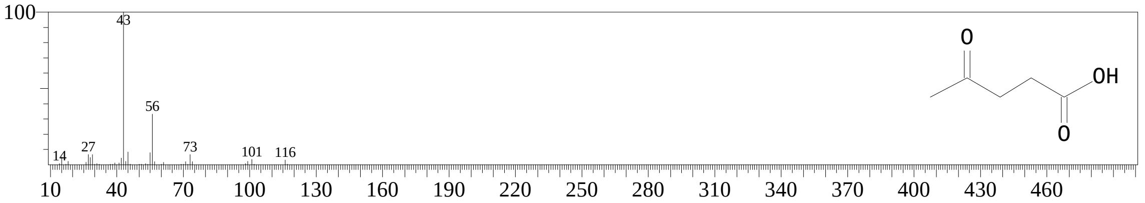

Hit#:3 Entry:2065 Library:NIST05.LIB
SI:80 Formula:C4H7NO2 CAS:1192-20-7 MolWeight:101 RetIndex:1030
CompName:Alpha-amino-gamma-butyrolactone $$ 3-Aminodihydro-2(3H)-furanone # $$

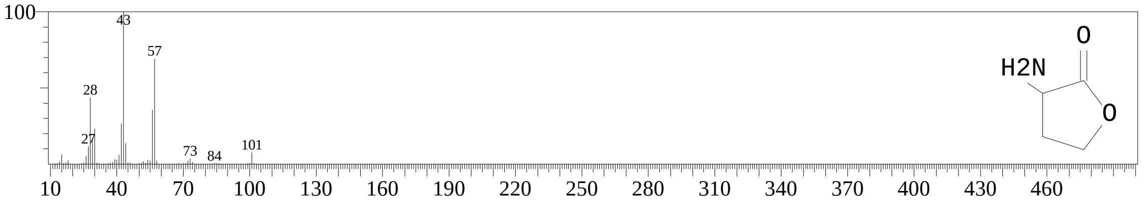

Hit#:4 Entry:3405 Library:NIST05s.LIB
SI:79 Formula:C5H8O3 CAS:123-76-2 MolWeight:116 RetIndex:1011
CompName:Pentanoic acid, 4-oxo- $$ Levulinic acid $$ .beta.-Acetylpropionic acid $$ .gamma.-Ketovaleric acid $$ Laevulinic aci

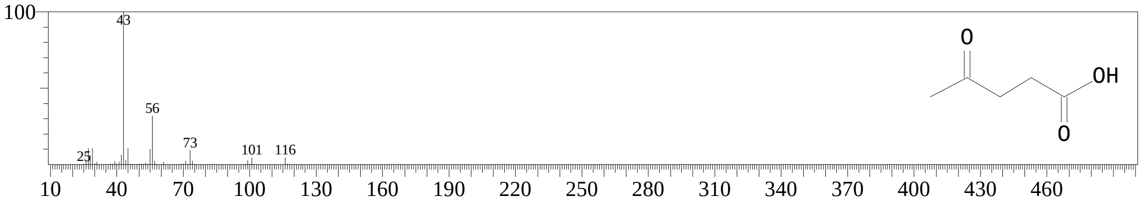

Hit#:5 Entry:2172 Library:NIST05.LIB
SI:79 Formula:C4H6O3 CAS:600-22-6 MolWeight:102 RetIndex:722
CompName:Propanoic acid, 2-oxo-, methyl ester $$ Pyruvic acid, methyl ester $$ Methyl pyruvate $$ Methylglyoxylic acid methyl

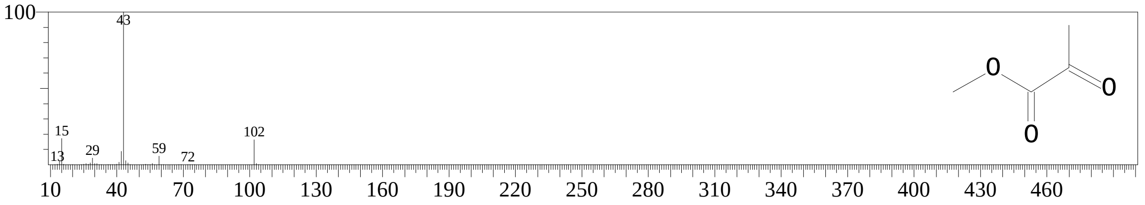

<< Target >>
Line#:6 R.Time:11.842(Scan#:1062) MassPeaks:233 BasePeak:43.00(75804)
RawMode:Averaged 11.833-11.850(1061-1063) BG Mode:Calc. from Peak

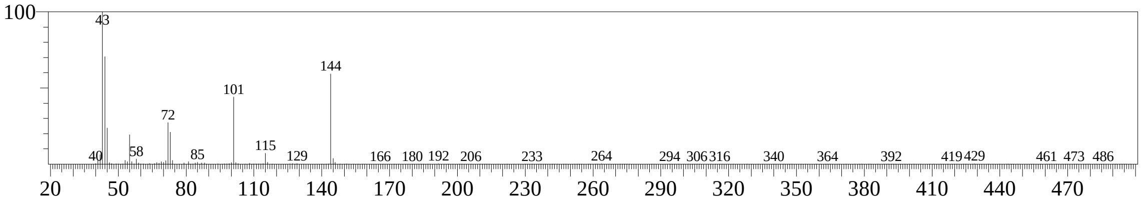

Hit#:1 Entry:7403 Library:NIST05s.LIB
SI:96 Formula:C6H8O4 CAS:28564-83-2 MolWeight:144 RetIndex:1269
CompName:4H-Pyran-4-one, 2,3-dihydro-3,5-dihydroxy-6-methyl- $$ 3,5-Dihydroxy-6-methyl-2,3-dihydro-4H-pyran-4-one # $$

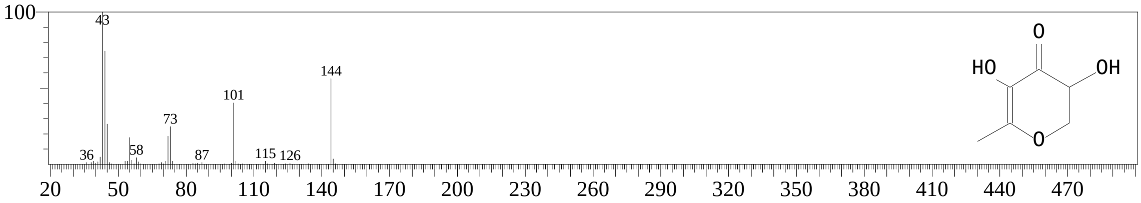

Hit#:2 Entry:12367 Library:NIST05.LIB
SI:92 Formula:C6H8O4 CAS:28564-83-2 MolWeight:144 RetIndex:1269
CompName:4H-Pyran-4-one, 2,3-dihydro-3,5-dihydroxy-6-methyl- $$ 3,5-Dihydroxy-6-methyl-2,3-dihydro-4H-pyran-4-one # $$

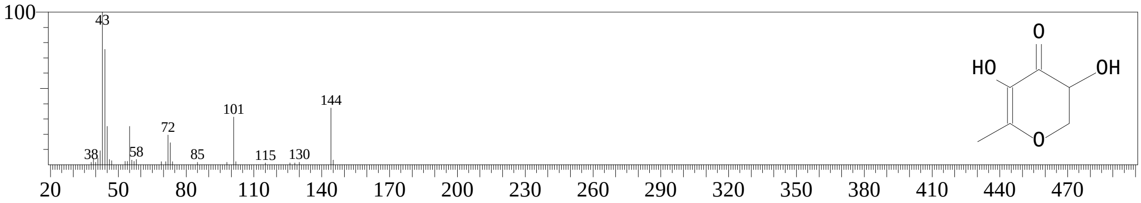

Hit#:3 Entry:4455 Library:NIST05.LIB
SI:82 Formula:C6H12O2 CAS:3299-32-9 MolWeight:116 RetIndex:761
CompName:1,3-Dioxolane, 2,4,5-trimethyl- $$ 2,4,5-Trimethyl-1,3-dioxolane # $$

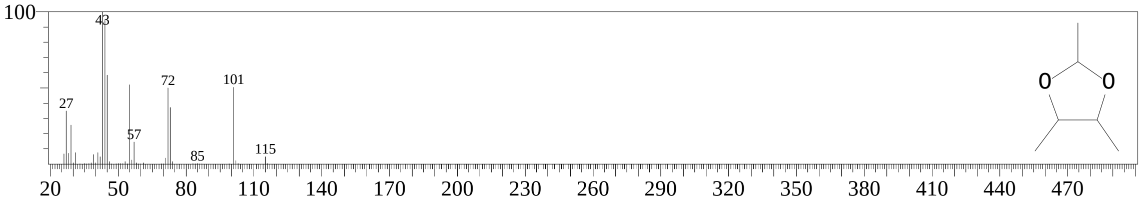

Hit#:4 Entry:12371 Library:NIST05.LIB
SI:80 Formula:C6H8O4 CAS:10230-62-3 MolWeight:144 RetIndex:1173
CompName:2,4-Dihydroxy-2,5-dimethyl-3(2H)-furan-3-one $$ 2,4-Dihydroxy-2,5-dimethyl-3(2H)-furanone # $$

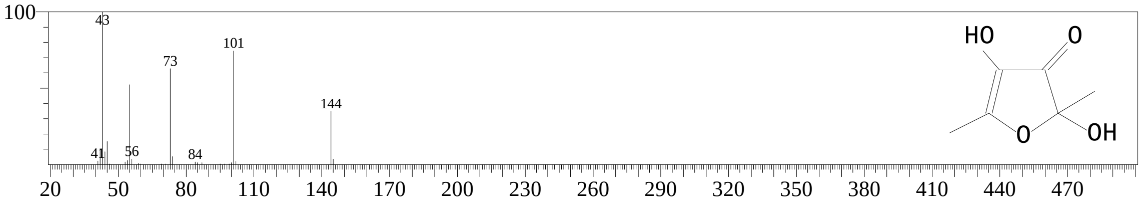

Hit#:5 Entry:12589 Library:NIST05.LIB
SI:78 Formula:C8H16O2 CAS:0-0-0 MolWeight:144 RetIndex:1156
CompName:2-Propyl-tetrahydropyran-3-ol

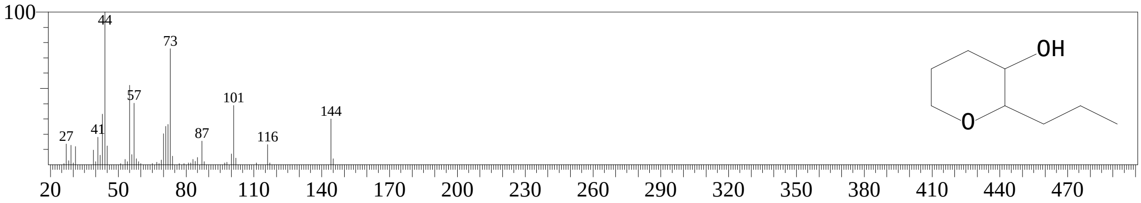

<< Target >>
Line#:7   R.Time:12.942(Scan#:1194) MassPeaks:251   BasePeak:71.95(19975)
RawMode:Averaged 12.933-12.950(1193-1195)   BG Mode:Calc. from Peak

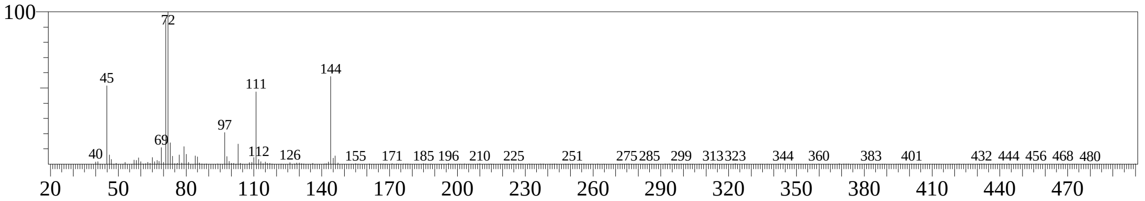

Hit#:1 Entry:12396 Library:NIST05.LIB
SI:93 Formula:C6H8S2 CAS:62488-53-3 MolWeight:144 RetIndex:1134
CompName:3-Vinyl-1,2-dithiacyclohex-5-ene $$ 3-Vinyl-3,4-dihydro-1,2-dithiine # $$

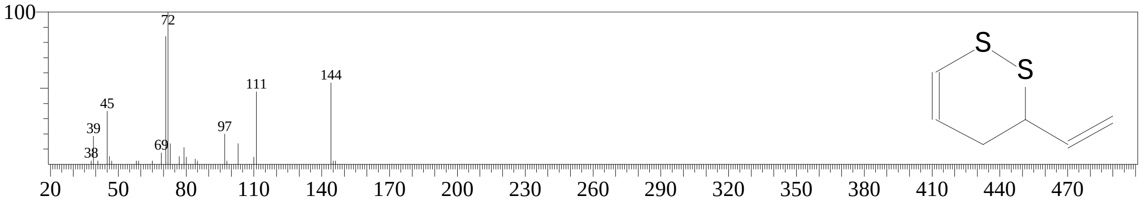

Hit#:2 Entry:12397 Library:NIST05.LIB
SI:75 Formula:C6H8S2 CAS:62488-52-2 MolWeight:144 RetIndex:1134
CompName:3-Vinyl-1,2-dithiacyclohex-4-ene $$ 3-Vinyl-3,6-dihydro-1,2-dithiine # $$

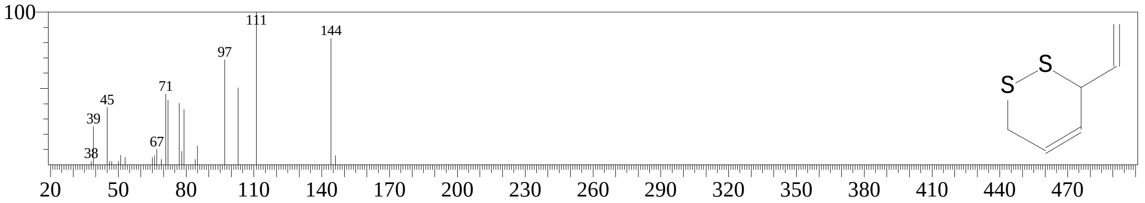

Hit#:3 Entry:12639 Library:NIST05.LIB
SI:71 Formula:C8H16O2 CAS:29887-60-3 MolWeight:144 RetIndex:993
CompName:Cyclohexane, 1,2-dimethoxy-, trans- $$ trans-1,2-Dimethoxycyclohexane $$ 1,2-Dimethoxycyclohexane # $$

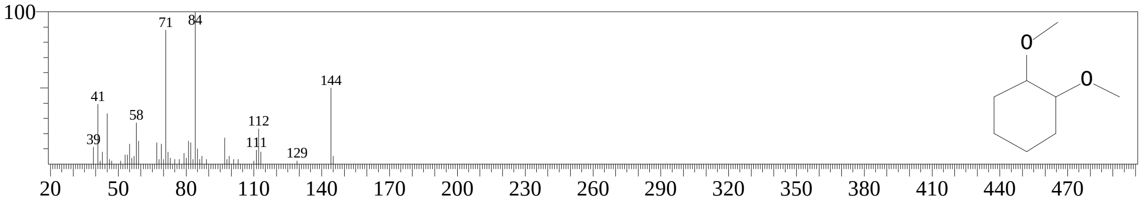

Hit#:4 Entry:12638 Library:NIST05.LIB
SI:70 Formula:C8H16O2 CAS:30363-80-5 MolWeight:144 RetIndex:993
CompName:Cyclohexane, 1,2-dimethoxy-, cis- $$ cis-1,2-Dimethoxycyclohexane $$ 1,2-Dimethoxycyclohexane # $$

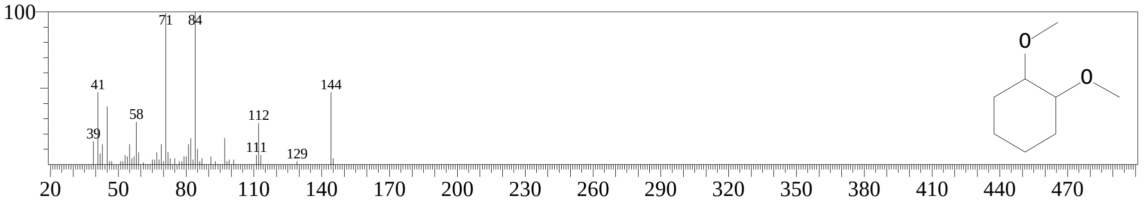

Hit#:5 Entry:3409 Library:NIST05.LIB
SI:70 Formula:C6H8S CAS:89533-93-7 MolWeight:112 RetIndex:894
CompName:Sulfide,1- propenyl 1-propynyl $$ (1E)-1-(1-Propynylsulfanyl)-1-propene # $$

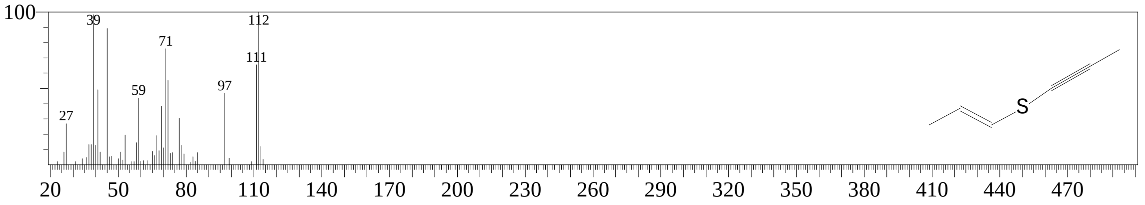

<< Target >>
Line#:8   R.Time:14.425(Scan#:1372) MassPeaks:241   BasePeak:41.00(14100)
RawMode:Averaged 14.417-14.433(1371-1373)   BG Mode:Calc. from Peak

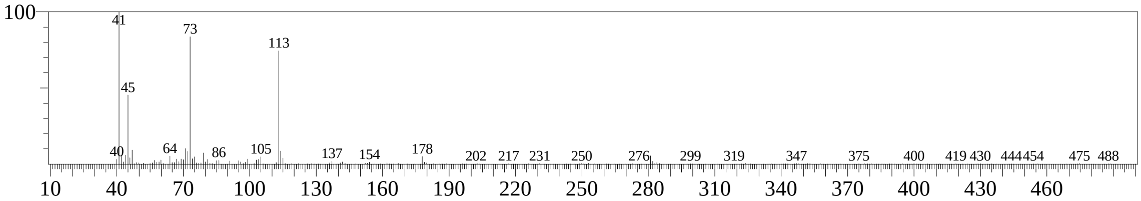

Hit#:1 Entry:28371 Library:NIST05.LIB
SI:91 Formula:C6H10S3 CAS:2050-87-5 MolWeight:178 RetIndex:1350
CompName:Trisulfide, di-2-propenyl $$ Allyl trisulfide $$ Diallyl trisulfide $$ 1,3-Diallyltrisulfane # $$

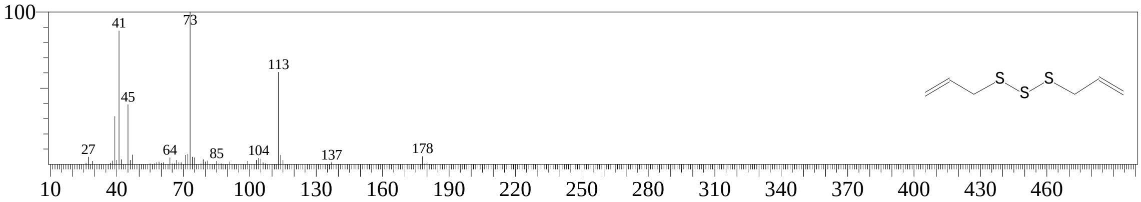

Hit#:2 Entry:12960 Library:NIST05s.LIB
SI:87 Formula:C6H10S3 CAS:2050-87-5 MolWeight:178 RetIndex:1350
CompName:Trisulfide, di-2-propenyl $$ Allyl trisulfide $$ Diallyl trisulfide $$ 1,3-Diallyltrisulfane # $$

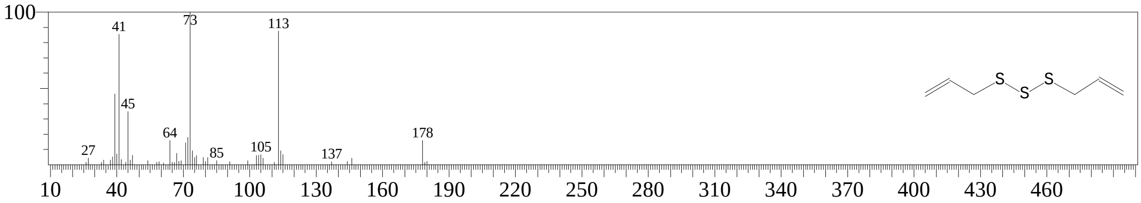

Hit#:3 Entry:3712 Library:NIST05.LIB
SI:84 Formula:C5H7NS CAS:105643-80-9 MolWeight:113 RetIndex:1004
CompName:2-Propenylthioacetonitrile $$ 2-Propenylthioacetonitrile $$ (Allylsulfanyl)acetonitrile # $$

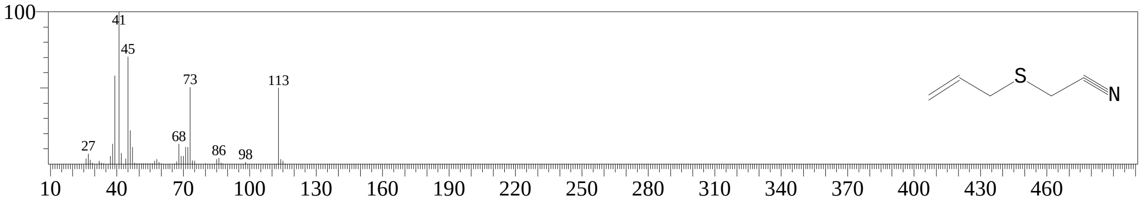

Hit#:4 Entry:13170 Library:NIST05.LIB
SI:77 Formula:C6H10S2 CAS:2179-57-9 MolWeight:146 RetIndex:1099
CompName:Diallyl disulphide $$ Allyl disulfide $$ Diallyl disulfide $$ Disulfide, di-2-propenyl $$ Allyl disulphide $$ 4,5-Dithia-

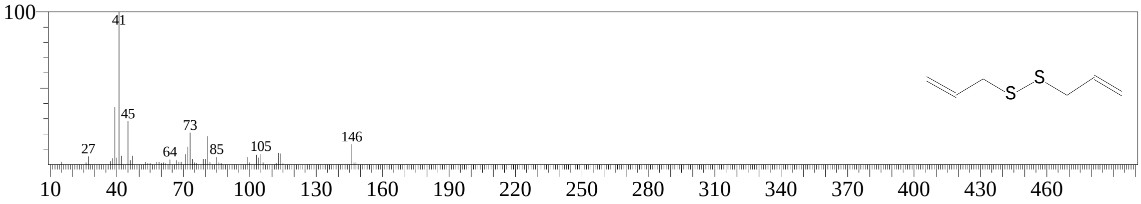

Hit#:5 Entry:3168 Library:NIST05s.LIB
SI:76 Formula:C6H10S CAS:592-88-1 MolWeight:114 RetIndex:849
CompName:1-Propene, 3,3'-thiobis- $$ Allyl sulfide $$ Allyl monosulfide $$ Diallyl monosulfide $$ Diallyl sulfide $$ Diallyl thioa

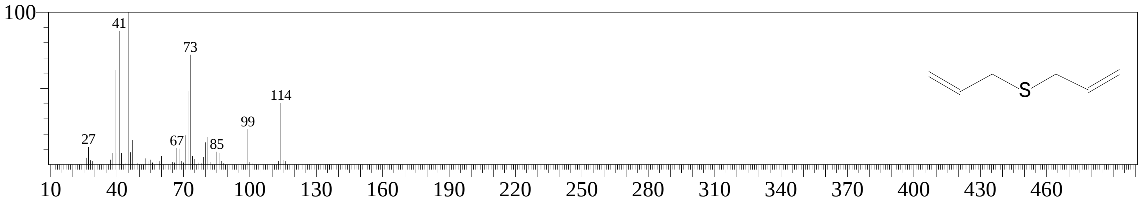

<< Target >>
Line#:9   R.Time:15.925(Scan#:1552) MassPeaks:258   BasePeak:57.00(5047)
RawMode:Averaged 15.917-15.933(1551-1553)   BG Mode:Calc. from Peak

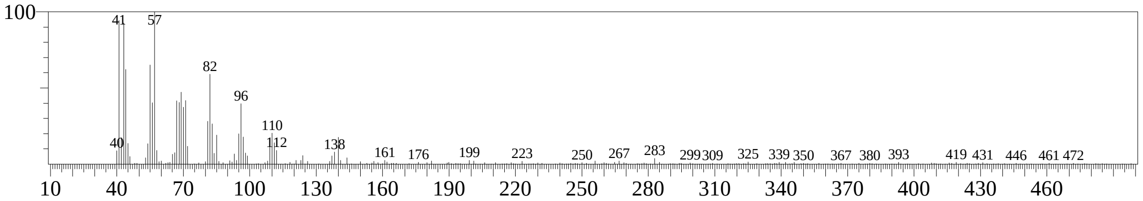

Hit#:1 Entry:14087 Library:NIST05s.LIB
SI:93 Formula:C12H24O CAS:112-54-9 MolWeight:184 RetIndex:1402
CompName:Dodecanal $$ Lauraldehyde $$ n-Dodecanal $$ n-Dodecyl aldehyde $$ n-Dodecylic aldehyde $$ n-Lauraldehyde $$ A

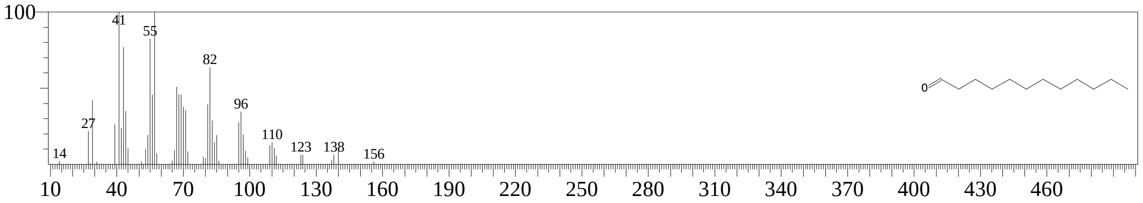

Hit#:2 Entry:14089 Library:NIST05s.LIB
SI:93 Formula:C12H24O CAS:112-54-9 MolWeight:184 RetIndex:1402
CompName:Dodecanal $$ Lauraldehyde $$ n-Dodecanal $$ n-Dodecyl aldehyde $$ n-Dodecylic aldehyde $$ n-Lauraldehyde $$ A

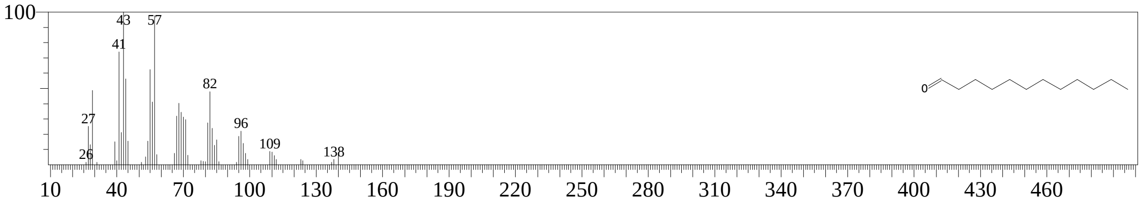

Hit#:3 Entry:17684 Library:NIST05s.LIB
SI:92 Formula:C14H28O CAS:124-25-4 MolWeight:212 RetIndex:1601
CompName:Tetradecanal $$ Myristaldehyde $$ Myristylaldehyde $$ Tetradecylaldehyde $$ n-Tetradecanal $$ Aldehyde C-14 $$ A

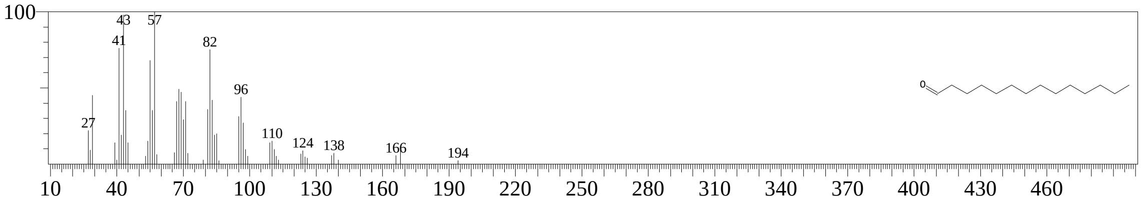

Hit#:4 Entry:17683 Library:NIST05s.LIB
SI:91 Formula:C14H28O CAS:124-25-4 MolWeight:212 RetIndex:1601
CompName:Tetradecanal $$ Myristaldehyde $$ Myristylaldehyde $$ Tetradecylaldehyde $$ n-Tetradecanal $$ Aldehyde C-14 $$ A

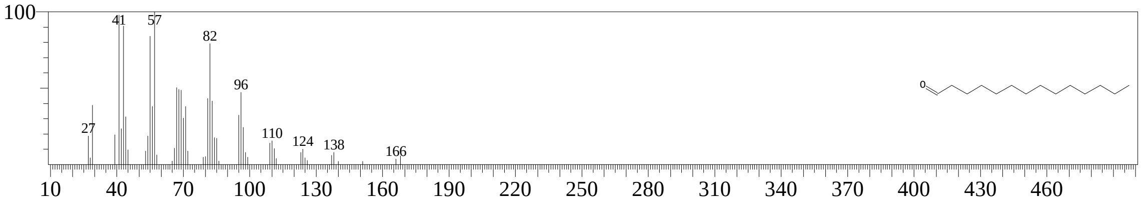

Hit#:5 Entry:40204 Library:NIST05.LIB
SI:91 Formula:C13H26O CAS:10486-19-8 MolWeight:198 RetIndex:1502
CompName:Tridecanal $$ n-Tridecylaldehyde $$ Tridecanaldehyde $$ Tridecyl aldehyde $$ 1-Tridecanal $$ Tridecane aldehyde $$

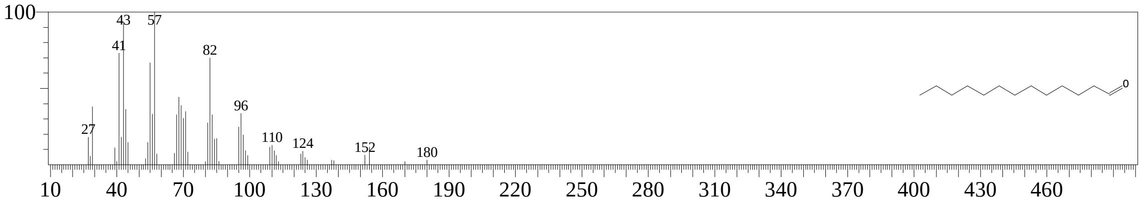

<< Target >>
Line#:10 R.Time:16.625(Scan#:1636) MassPeaks:304 BasePeak:57.00(110691)
RawMode:Averaged 16.617-16.633(1635-1637) BG Mode:Calc. from Peak

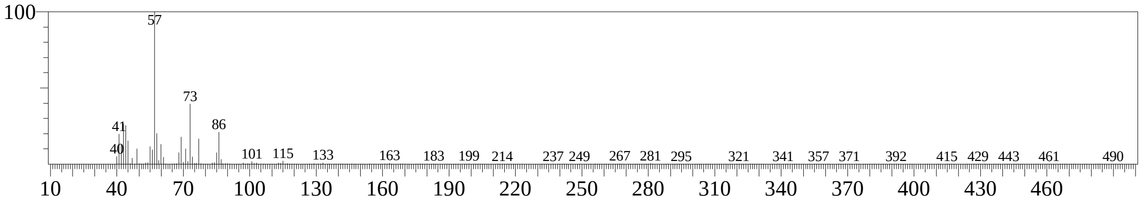

Hit#:1 Entry:123174 Library:NIST05.LIB
SI:83 Formula:C12H22O11 CAS:57-50-1 MolWeight:342 RetIndex:3139
CompName:Sucrose $$ .alpha.-D-Glucopyranoside, .beta.-D-fructofuranosyl $$ .beta.-D-Fructofuranosyl .alpha.-D-glucopyranoside

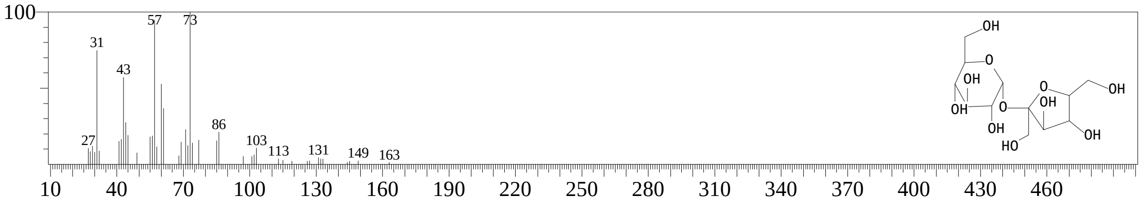

Hit#:2 Entry:8563 Library:NIST05s.LIB
SI:82 Formula:C4H9NO5 CAS:126-11-4 MolWeight:151 RetIndex:1444
CompName:1,3-Propanediol, 2-(hydroxymethyl)-2-nitro- $$ Isobutylglycerol, nitro- $$ Methane, trimethylolnitro- $$ Nitroisobutylg

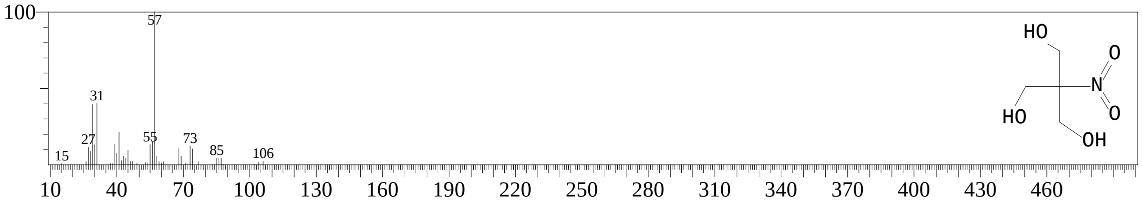

Hit#:3 Entry:5797 Library:NIST05s.LIB
SI:82 Formula:C6H14O3 CAS:77-99-6 MolWeight:134 RetIndex:1261
CompName:1,3-Propanediol, 2-ethyl-2-(hydroxymethyl)- $$ Ethriol $$ Ethyltrimethylolmethane $$ Etriol $$ Ettriol $$ Hexaglyceri

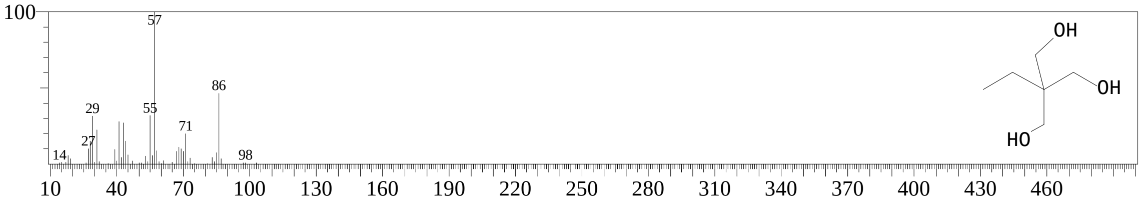

Hit#:4 Entry:15081 Library:NIST05.LIB
SI:81 Formula:C4H9NO5 CAS:126-11-4 MolWeight:151 RetIndex:1444
CompName:1,3-Propanediol, 2-(hydroxymethyl)-2-nitro- $$ Isobutylglycerol, nitro- $$ Methane, trimethylolnitro- $$ Nitroisobutylg

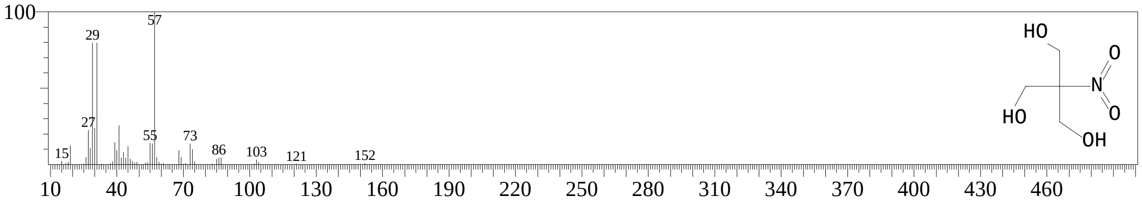

Hit#:5 Entry:1952 Library:NIST05s.LIB
SI:80 Formula:C4H6O3 CAS:108-32-7 MolWeight:102 RetIndex:875
CompName:Propylene Carbonate $$ 1,3-Dioxolan-2-one, 4-methyl- $$ Carbonic acid, cyclic propylene ester $$ Cyclic methylethyl

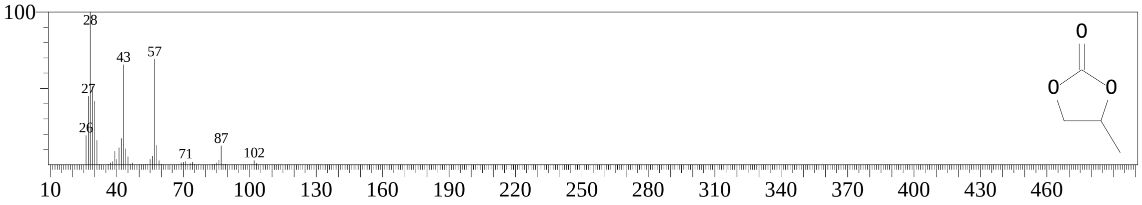

<< Target >>
Line#:11   R.Time:16.825(Scan#:1660) MassPeaks:261   BasePeak:41.00(4409)
RawMode:Averaged 16.817-16.833(1659-1661)   BG Mode:Calc. from Peak

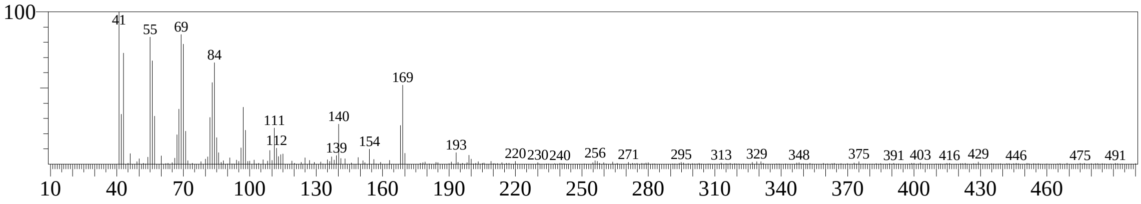

Hit#:1 Entry:23837 Library:NIST05.LIB
SI:86 Formula:C12H24 CAS:294-62-2 MolWeight:168 RetIndex:1439
CompName:Cyclododecane

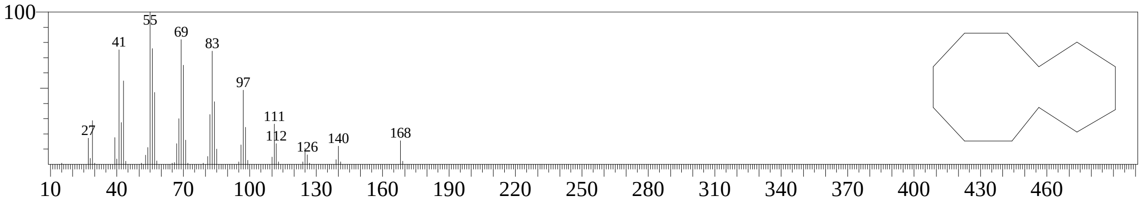

Hit#:2 Entry:11686 Library:NIST05s.LIB
SI:86 Formula:C12H24 CAS:294-62-2 MolWeight:168 RetIndex:1439
CompName:Cyclododecane

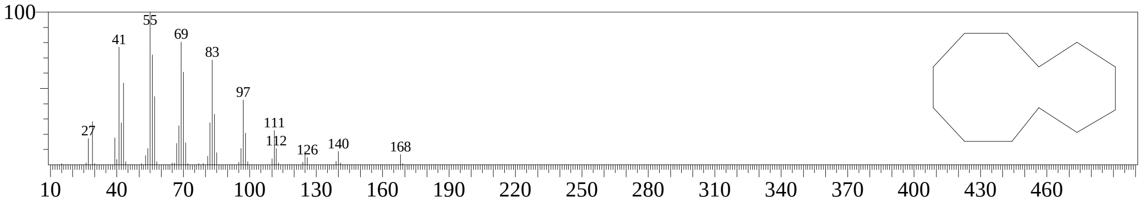

Hit#:3 Entry:14421 Library:NIST05s.LIB
SI:86 Formula:C12H26O CAS:112-53-8 MolWeight:186 RetIndex:1457
CompName:1-Dodecanol $$ Dodecyl alcohol $$ n-Dodecan-1-ol $$ n-Dodecanol $$ n-Dodecyl alcohol $$ Alcohol C-12 $$ Alfol 1

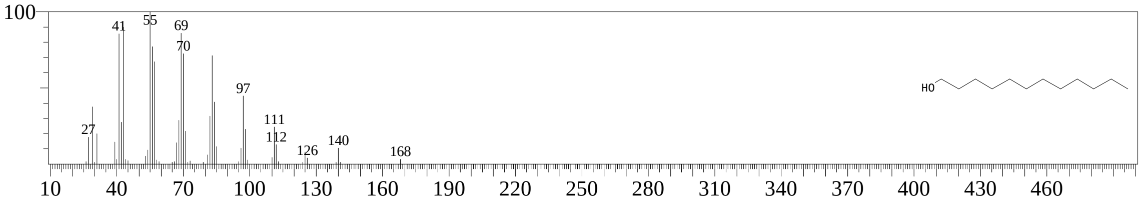

Hit#:4 Entry:33451 Library:NIST05.LIB
SI:85 Formula:C12H26O CAS:112-53-8 MolWeight:186 RetIndex:1457
CompName:1-Dodecanol $$ Dodecyl alcohol $$ n-Dodecan-1-ol $$ n-Dodecanol $$ n-Dodecyl alcohol $$ Alcohol C-12 $$ Alfol 1

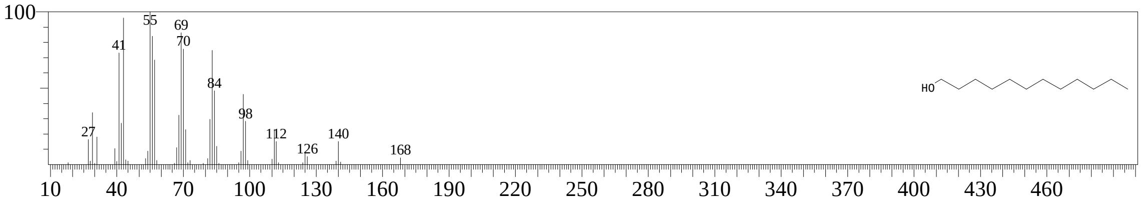

Hit#:5 Entry:17937 Library:NIST05s.LIB
SI:85 Formula:C14H30O CAS:112-72-1 MolWeight:214 RetIndex:1656
CompName:1-Tetradecanol $$ n-Tetradecan-1-ol $$ n-Tetradecanol $$ n-Tetradecyl alcohol $$ Alfol 14 $$ Lanette K $$ Lanette W

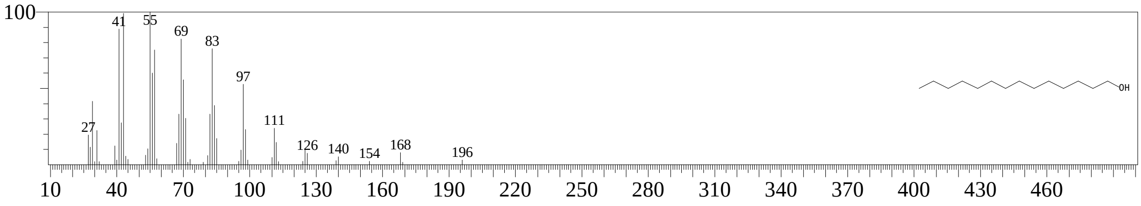

<< Target >>
Line#:12 R.Time:18.825(Scan#:1900) MassPeaks:248 BasePeak:170.05(12842)
RawMode:Averaged 18.817-18.833(1899-1901) BG Mode:Calc. from Peak

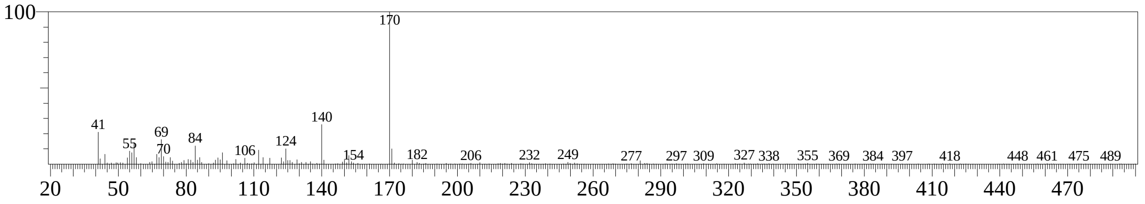

Hit#:1 Entry:139920 Library:NIST05.LIB
SI:74 Formula:C22H41NO4 CAS:0-0-0 MolWeight:383 RetIndex:2603
CompName:l-Isoleucine, N-allyloxycarbonyl-, dodecyl ester

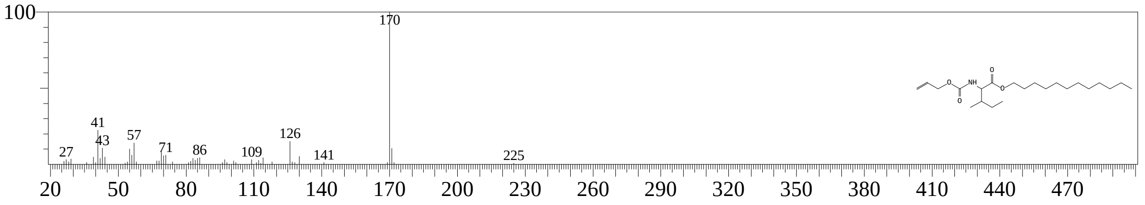

Hit#:2 Entry:115885 Library:NIST05.LIB
SI:73 Formula:C18H33NO4 CAS:0-0-0 MolWeight:327 RetIndex:2205
CompName:l-Isoleucine, N-allyloxycarbonyl-, octyl ester

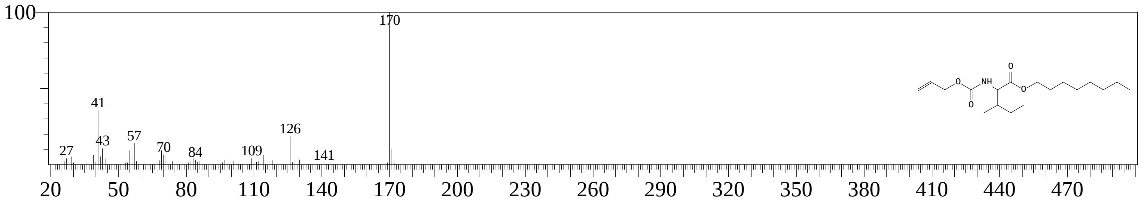

Hit#:3 Entry:66855 Library:NIST05.LIB
SI:73 Formula:C12H21NO4 CAS:0-0-0 MolWeight:243 RetIndex:1609
CompName:l-Isoleucine, N-allyloxycarbonyl-, ethyl ester

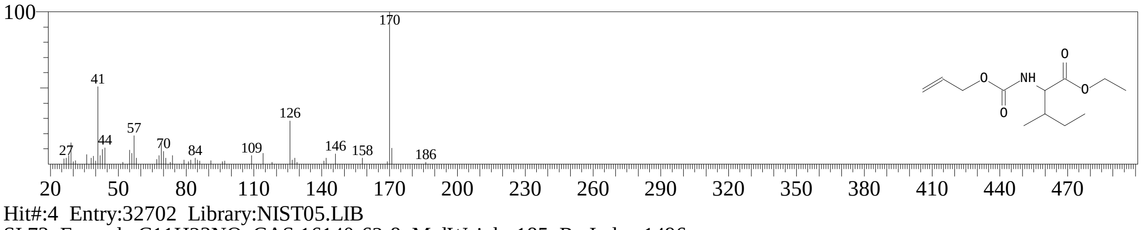

Hit#:4 Entry:32702 Library:NIST05.LIB
SI:73 Formula:C11H23NO CAS:16140-62-8 MolWeight:185 RetIndex:1496
CompName:3-(5-Ethyl-2-methylpiperidino)-1-propanol $$ 3-(5-Ethyl-2-methyl-1-piperidinyl)-1-propanol # $$

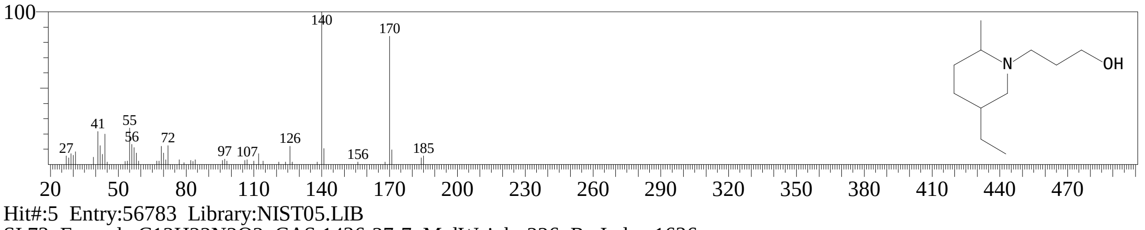

Hit#:5 Entry:56783 Library:NIST05.LIB
SI:73 Formula:C12H22N2O2 CAS:1436-27-7 MolWeight:226 RetIndex:1636
CompName:2,5-Piperazinedione, 3,6-bis(2-methylpropyl)- $$ 3,6-Diisobutyl-2,5-piperazinedione # $$

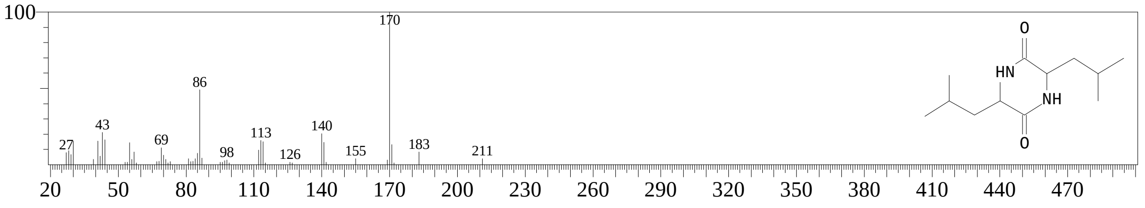

<< Target >>
Line#:13   R.Time:19.525(Scan#:1984) MassPeaks:255   BasePeak:144.00(58611)
RawMode:Averaged 19.517-19.533(1983-1985)   BG Mode:Calc. from Peak

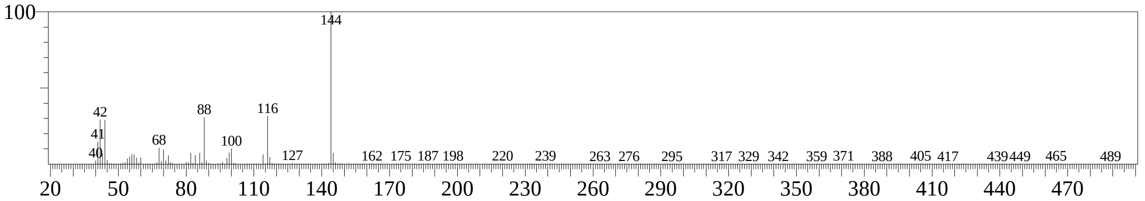

Hit#:1 Entry:67958 Library:NIST05.LIB
SI:81 Formula:C12H23NO4 CAS:0-0-0 MolWeight:245 RetIndex:1619
CompName:l-Alanine, N-butoxycarbonyl-, isobutyl ester

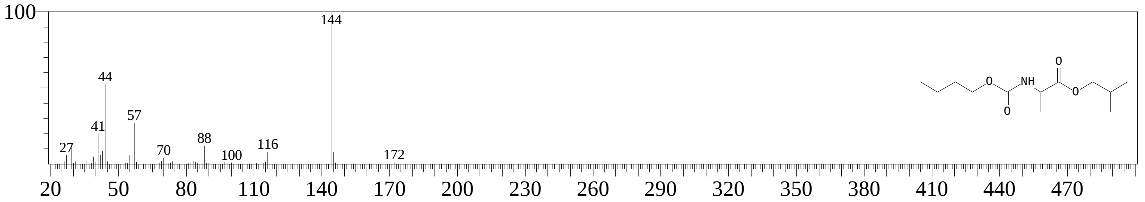

Hit#:2 Entry:109550 Library:NIST05.LIB
SI:81 Formula:C17H33NO4 CAS:0-0-0 MolWeight:315 RetIndex:2180
CompName:l-Alanine, N-butoxycarbonyl-, nonyl ester

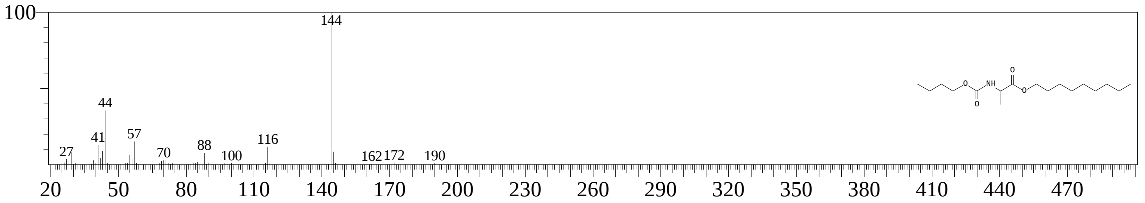

Hit#:3 Entry:93408 Library:NIST05.LIB
SI:81 Formula:C15H29NO4 CAS:0-0-0 MolWeight:287 RetIndex:1981
CompName:l-Alanine, N-butoxycarbonyl-, heptyl ester

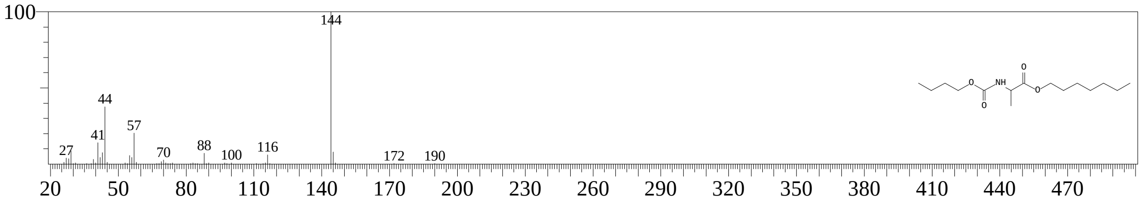

Hit#:4 Entry:34681 Library:NIST05.LIB
SI:81 Formula:C10H23NO2 CAS:0-0-0 MolWeight:189 RetIndex:1389
CompName:Butanamine, N,N-bis(2-hydroxypropyl)-

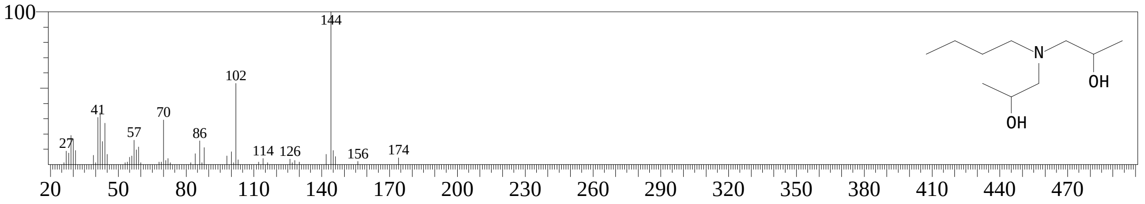

Hit#:5 Entry:76475 Library:NIST05.LIB
SI:80 Formula:C13H25NO4 CAS:0-0-0 MolWeight:259 RetIndex:1782
CompName:l-Alanine, N-butoxycarbonyl-, pentyl ester

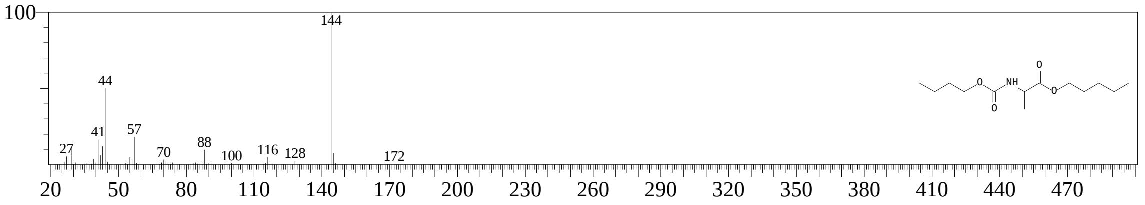

<< Target >>
Line#:14 R.Time:20.383(Scan#:2087) MassPeaks:275 BasePeak:73.00(9342)
RawMode:Averaged 20.375-20.392(2086-2088) BG Mode:Calc. from Peak

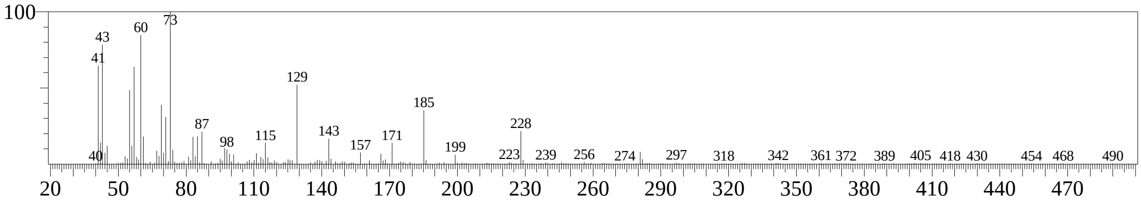

Hit#:1 Entry:19252 Library:NIST05s.LIB
SI:94 Formula:C14H28O2 CAS:544-63-8 MolWeight:228 RetIndex:1769
CompName:Tetradecanoic acid $$ Myristic acid $$ n-Tetradecanoic acid $$ n-Tetradecoic acid $$ Neo-Fat 14 $$ Univol U 316S $

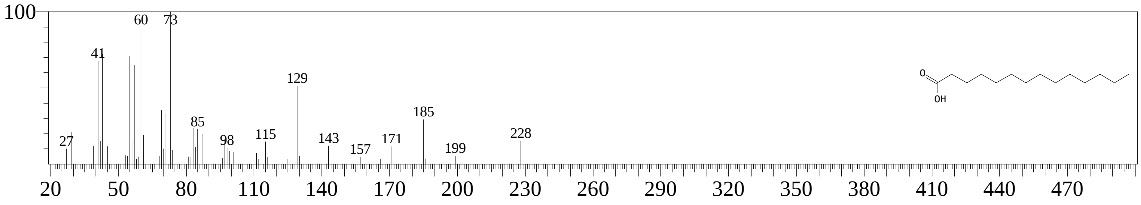

Hit#:2 Entry:19251 Library:NIST05s.LIB
SI:92 Formula:C14H28O2 CAS:544-63-8 MolWeight:228 RetIndex:1769
CompName:Tetradecanoic acid $$ Myristic acid $$ n-Tetradecanoic acid $$ n-Tetradecoic acid $$ Neo-Fat 14 $$ Univol U 316S $

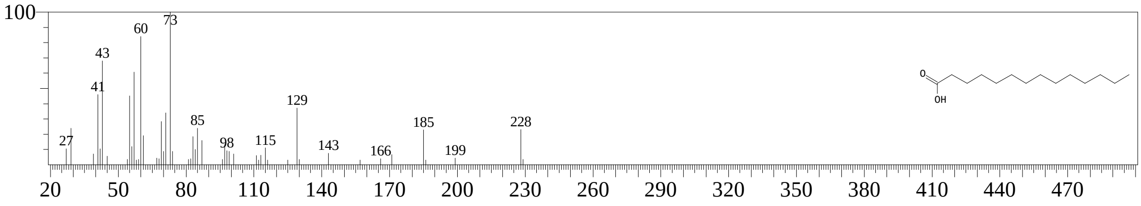

Hit#:3 Entry:58280 Library:NIST05.LIB
SI:92 Formula:C14H28O2 CAS:544-63-8 MolWeight:228 RetIndex:1769
CompName:Tetradecanoic acid $$ Myristic acid $$ n-Tetradecanoic acid $$ n-Tetradecoic acid $$ Neo-Fat 14 $$ Univol U 316S $

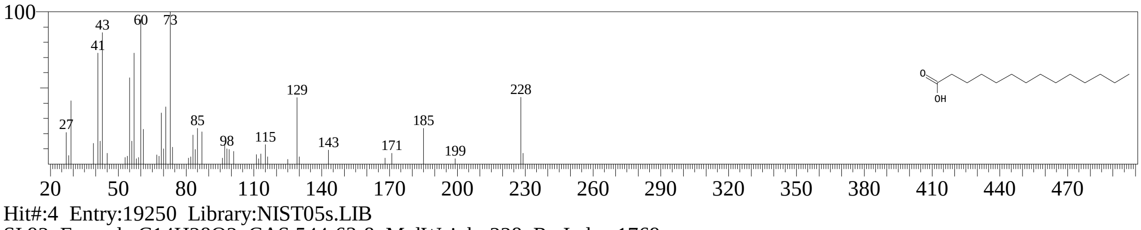

Hit#:4 Entry:19250 Library:NIST05s.LIB
SI:92 Formula:C14H28O2 CAS:544-63-8 MolWeight:228 RetIndex:1769
CompName:Tetradecanoic acid $$ Myristic acid $$ n-Tetradecanoic acid $$ n-Tetradecoic acid $$ Neo-Fat 14 $$ Univol U 316S $

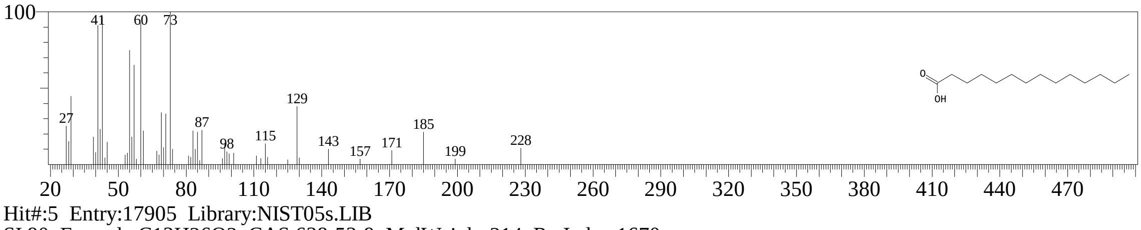

Hit#:5 Entry:17905 Library:NIST05s.LIB
SI:90 Formula:C13H26O2 CAS:638-53-9 MolWeight:214 RetIndex:1670
CompName:Tridecanoic acid $$ n-Tridecanoic acid $$ n-Tridecoic acid $$ Tridecylic acid $$

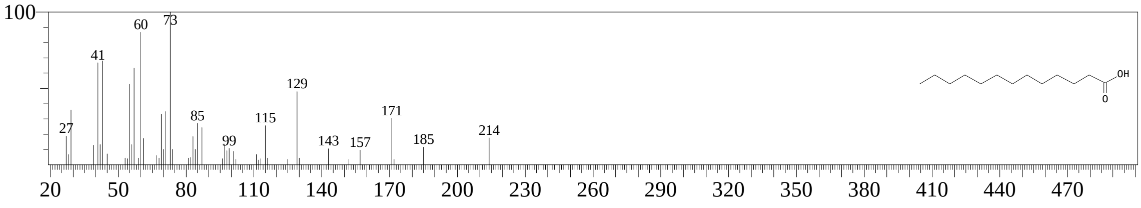

<< Target >>
Line#:15 R.Time:21.358(Scan#:2204) MassPeaks:269 BasePeak:69.00(10380)
RawMode:Averaged 21.350-21.367(2203-2205) BG Mode:Calc. from Peak

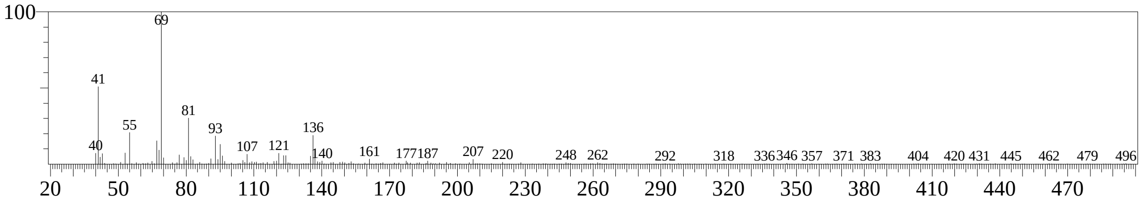

Hit#:1 Entry:70128 Library:NIST05.LIB
SI:92 Formula:C17H28O CAS:66408-55-7 MolWeight:248 RetIndex:1855
CompName:4,8,12-Tetradecatrienal, 5,9,13-trimethyl- $$ (4E,8E)-5,9,13-Trimethyl-4,8,12-tetradecatrienal # $$

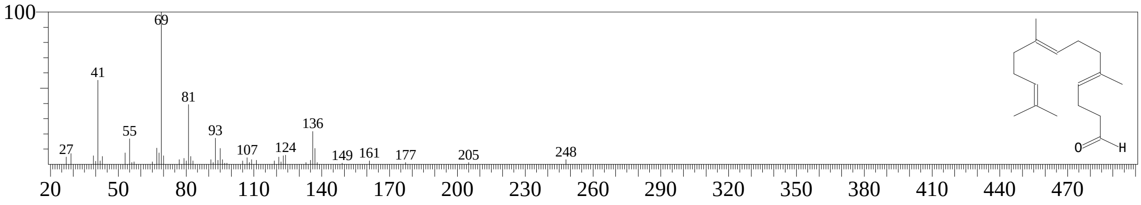

Hit#:2 Entry:141461 Library:NIST05.LIB
SI:88 Formula:C21H28N2O5 CAS:0-0-0 MolWeight:388 RetIndex:3007
CompName:1-(3,5-Dinitrophenoxy)-3,7,11-trimethyl-dodeca-2,6,10-triene

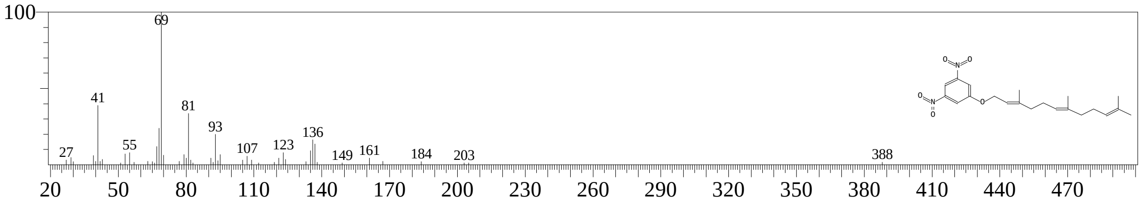

Hit#:3 Entry:18658 Library:NIST05s.LIB
SI:87 Formula:C15H26O CAS:106-28-5 MolWeight:222 RetIndex:1710
CompName:2,6,10-Dodecatrien-1-ol, 3,7,11-trimethyl-, (E,E)- $$ (E,E)-Farnesol $$ (2E,6E)-Farnesol $$ All-trans-Farnesol $$ trans

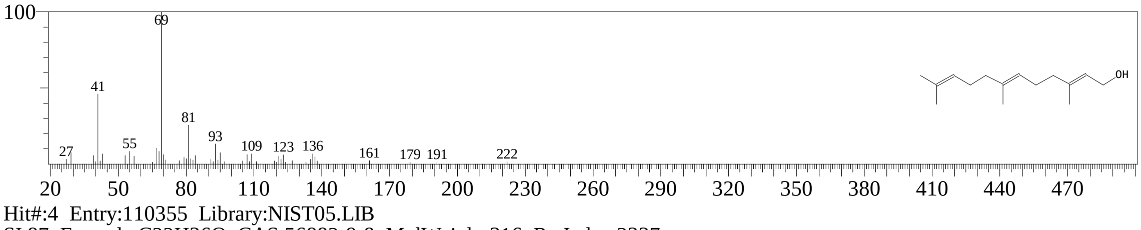

Hit#:4 Entry:110355 Library:NIST05.LIB
SI:87 Formula:C22H36O CAS:56882-9-8 MolWeight:316 RetIndex:2337
CompName:4,9,13,17-Tetramethyl-4,8,12,16-octadecatetraenal $$ (4E,8E,12E)-4,9,13,17-Tetramethyl-4,8,12,16-octadecatetraenal

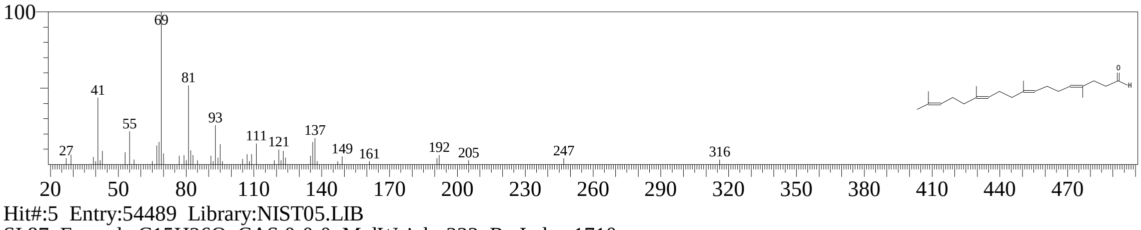

Hit#:5 Entry:54489 Library:NIST05.LIB
SI:87 Formula:C15H26O CAS:0-0-0 MolWeight:222 RetIndex:1710
CompName:Farnesol isomer a

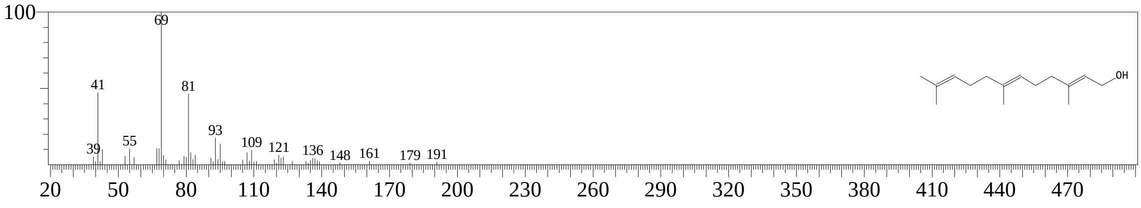

<< Target >>
Line#:16   R.Time:22.217(Scan#:2307) MassPeaks:281   BasePeak:74.00(14218)
RawMode:Averaged 22.208-22.225(2306-2308)   BG Mode:Calc. from Peak

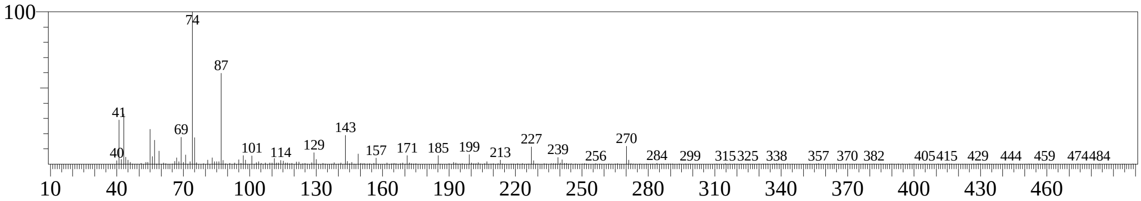

Hit#:1 Entry:83491 Library:NIST05.LIB
SI:93 Formula:C17H34O2 CAS:112-39-0 MolWeight:270 RetIndex:1878
CompName:Hexadecanoic acid, methyl ester $$ Palmitic acid, methyl ester $$ n-Hexadecanoic acid methyl ester $$ Metholene 221

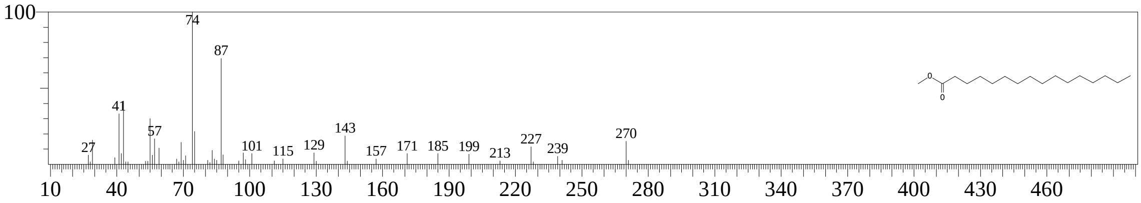

Hit#:2 Entry:22219 Library:NIST05s.LIB
SI:92 Formula:C17H34O2 CAS:112-39-0 MolWeight:270 RetIndex:1878
CompName:Hexadecanoic acid, methyl ester $$ Palmitic acid, methyl ester $$ n-Hexadecanoic acid methyl ester $$ Metholene 221

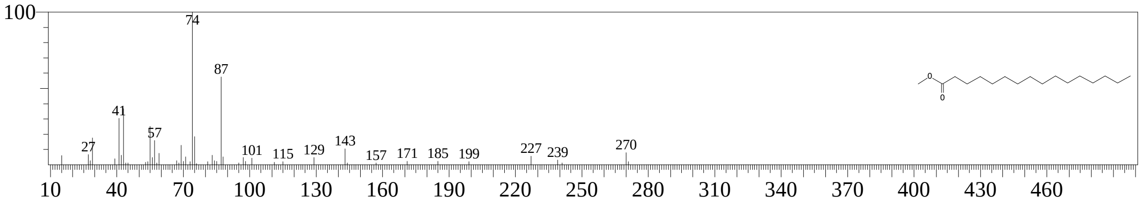

Hit#:3 Entry:22220 Library:NIST05s.LIB
SI:91 Formula:C17H34O2 CAS:112-39-0 MolWeight:270 RetIndex:1878
CompName:Hexadecanoic acid, methyl ester $$ Palmitic acid, methyl ester $$ n-Hexadecanoic acid methyl ester $$ Metholene 221

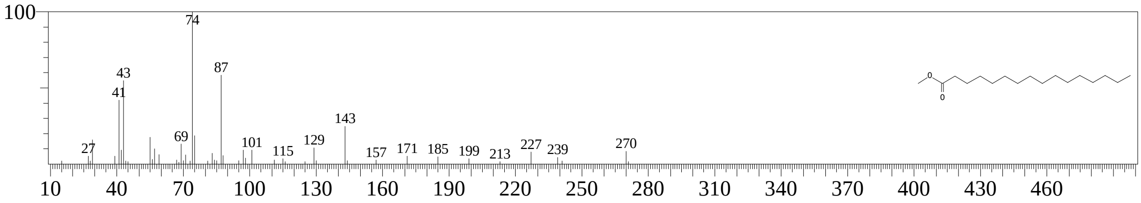

Hit#:4 Entry:22222 Library:NIST05s.LIB
SI:90 Formula:C17H34O2 CAS:5129-60-2 MolWeight:270 RetIndex:1814
CompName:Pentadecanoic acid, 14-methyl-, methyl ester $$ Methyl 14-methylpentadecanoate # $$

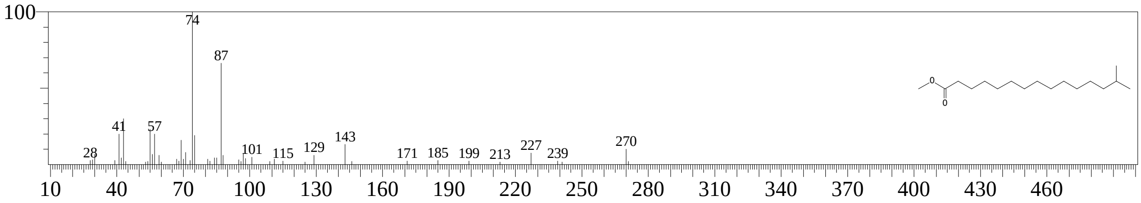

Hit#:5 Entry:83493 Library:NIST05.LIB
SI:90 Formula:C17H34O2 CAS:5129-60-2 MolWeight:270 RetIndex:1814
CompName:Pentadecanoic acid, 14-methyl-, methyl ester $$ Methyl 14-methylpentadecanoate # $$

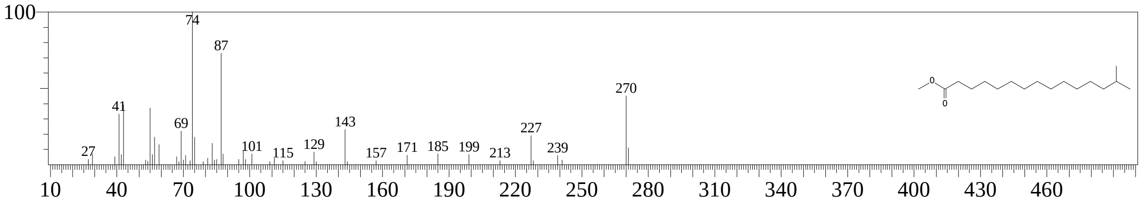

<< Target >>
Line#:17 R.Time:22.392(Scan#:2328) MassPeaks:288 BasePeak:41.00(8607)
RawMode:Averaged 22.383-22.400(2327-2329) BG Mode:Calc. from Peak

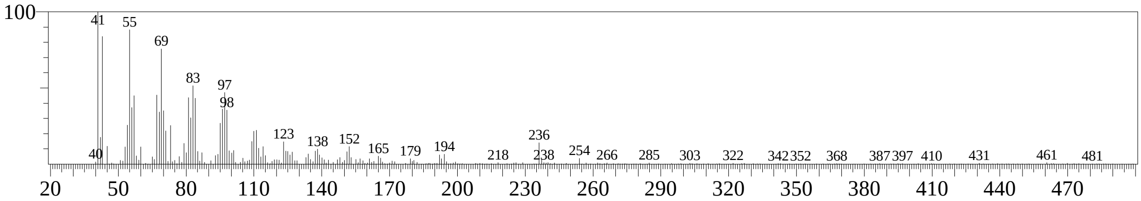

Hit#:1 Entry:90568 Library:NIST05.LIB
SI:92 Formula:C18H34O2 CAS:593-39-5 MolWeight:282 RetIndex:2175
CompName:6-Octadecenoic acid, (Z)- $$ (6Z)-6-Octadecenoic acid # $$

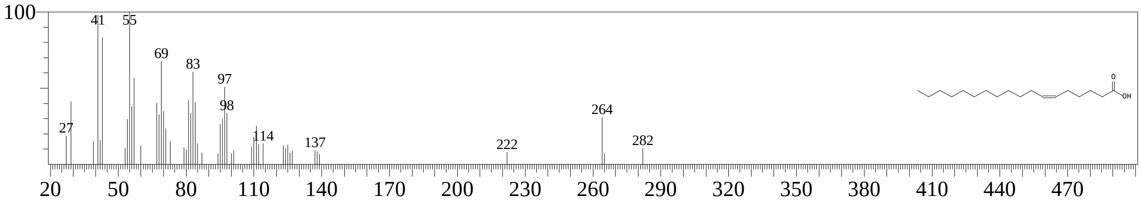

Hit#:2 Entry:65295 Library:NIST05.LIB
SI:92 Formula:C15H28O2 CAS:4727-18-8 MolWeight:240 RetIndex:2158
CompName:Cyclopentadecanone, 2-hydroxy- $$ 2-Hydroxycyclopentadecanone # $$

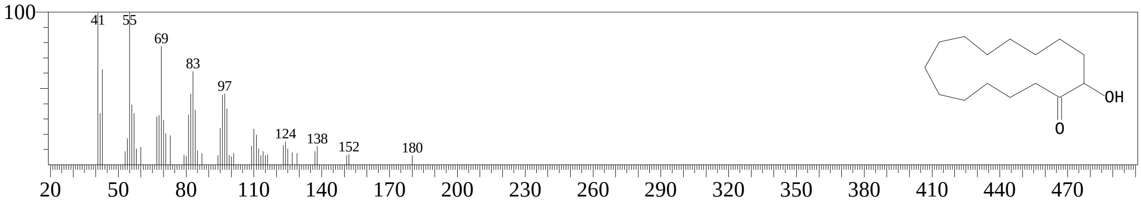

Hit#:3 Entry:90577 Library:NIST05.LIB
SI:91 Formula:C18H34O2 CAS:112-80-1 MolWeight:282 RetIndex:2175
CompName:Oleic Acid $$ 9-Octadecenoic acid (Z)- $$ .delta.(Sup9)-cis-Oleic acid $$ cis-.delta.(Sup9)-Octadecenoic acid $$ cis-C

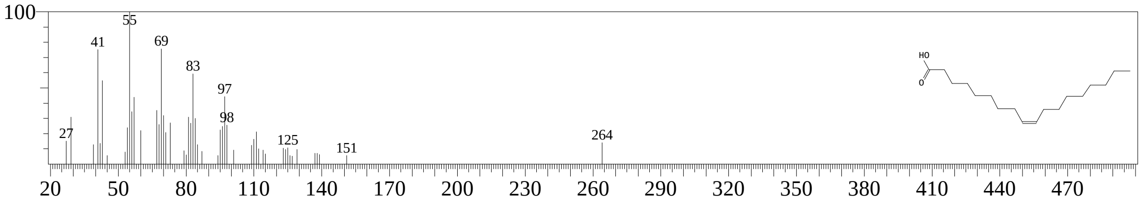

Hit#:4 Entry:73680 Library:NIST05.LIB
SI:89 Formula:C16H30O2 CAS:0-0-0 MolWeight:254 RetIndex:1976
CompName:Z-7-Hexadecenoic acid

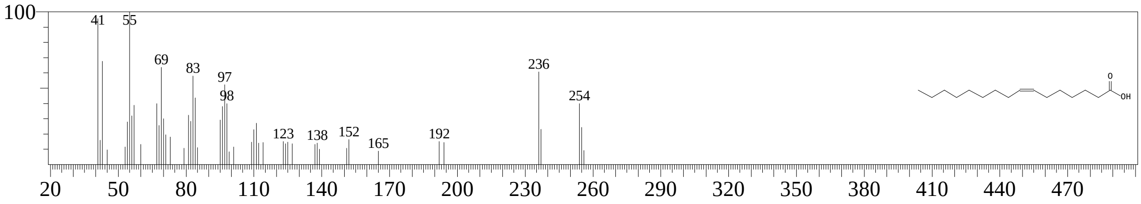

Hit#:5 Entry:73685 Library:NIST05.LIB
SI:89 Formula:C16H30O2 CAS:2091-29-4 MolWeight:254 RetIndex:1976
CompName:9-Hexadecenoic acid $$ (9E)-9-Hexadecenoic acid # $$

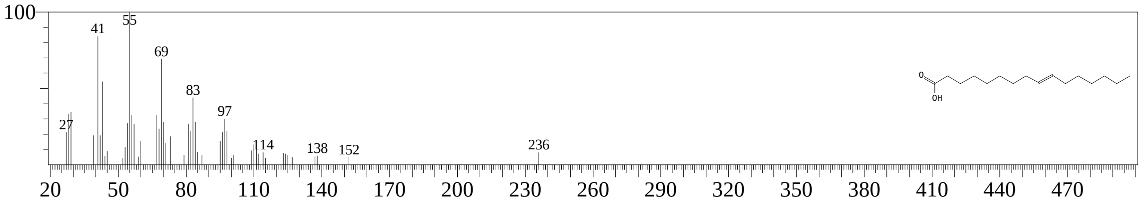

<< Target >>
Line#:18   R.Time:22.583(Scan#:2351) MassPeaks:308   BasePeak:43.05(45726)
RawMode:Averaged 22.575-22.592(2350-2352)   BG Mode:Calc. from Peak

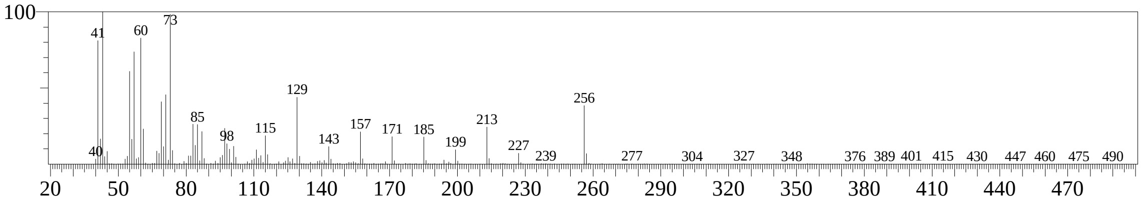

Hit#:1 Entry:21331 Library:NIST05s.LIB
SI:95 Formula:C16H32O2 CAS:57-10-3 MolWeight:256 RetIndex:1968
CompName:n-Hexadecanoic acid $$ Hexadecanoic acid $$ n-Hexadecoic acid $$ Palmitic acid $$ Pentadecanecarboxylic acid $$ 1

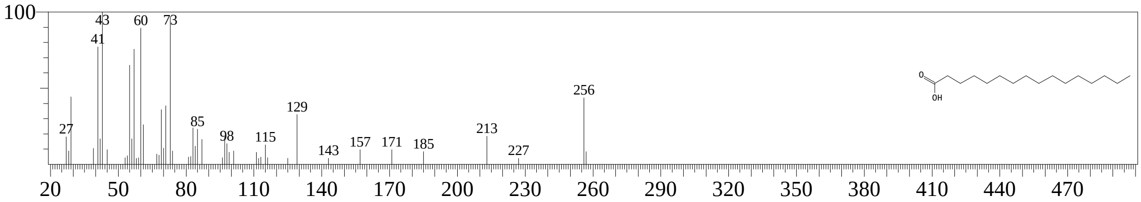

Hit#:2 Entry:20371 Library:NIST05s.LIB
SI:92 Formula:C15H30O2 CAS:1002-84-2 MolWeight:242 RetIndex:1869
CompName:Pentadecanoic acid $$ Pentadecylic acid $$ n-Pentadecanoic acid $$ n-Pentadecylic acid $$

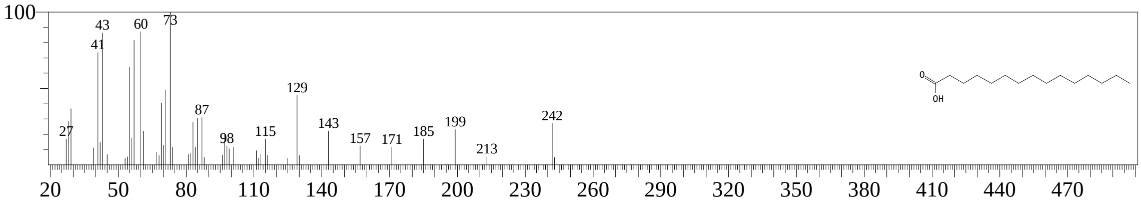

Hit#:3 Entry:161860 Library:NIST05.LIB
SI:92 Formula:C38H68O8 CAS:28474-90-0 MolWeight:652 RetIndex:4765
CompName:l-(+)-Ascorbic acid 2,6-dihexadecanoate

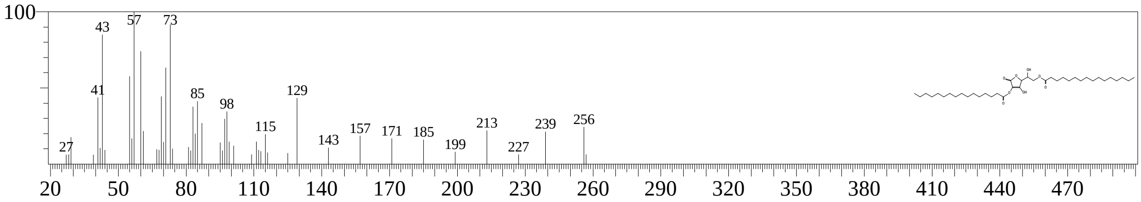

Hit#:4 Entry:74999 Library:NIST05.LIB
SI:91 Formula:C16H32O2 CAS:57-10-3 MolWeight:256 RetIndex:1968
CompName:n-Hexadecanoic acid $$ Hexadecanoic acid $$ n-Hexadecoic acid $$ Palmitic acid $$ Pentadecanecarboxylic acid $$ 1

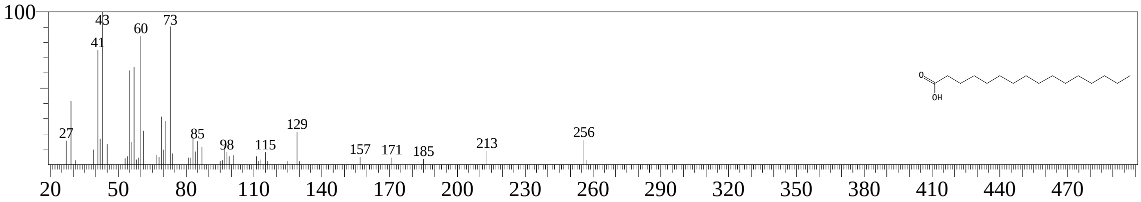

Hit#:5 Entry:22979 Library:NIST05s.LIB
SI:90 Formula:C18H36O2 CAS:57-11-4 MolWeight:284 RetIndex:2167
CompName:Octadecanoic acid $$ Stearic acid $$ n-Octadecanoic acid $$ Humko Industrene R $$ Hydrofol Acid 150 $$ Hystrene

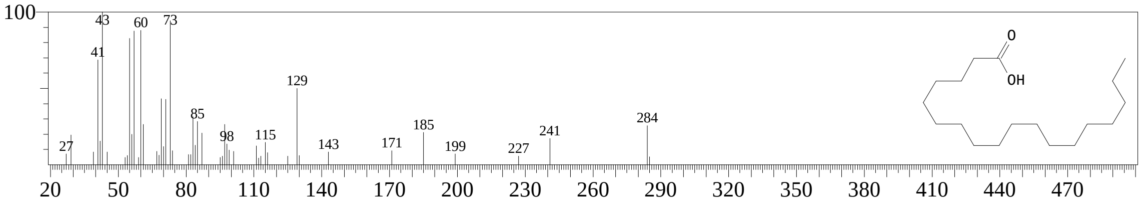

<< Target >>
Line#:19   R.Time:24.400(Scan#:2569) MassPeaks:330   BasePeak:174.00(20413)
RawMode:Averaged 24.392-24.408(2568-2570)   BG Mode:Calc. from Peak

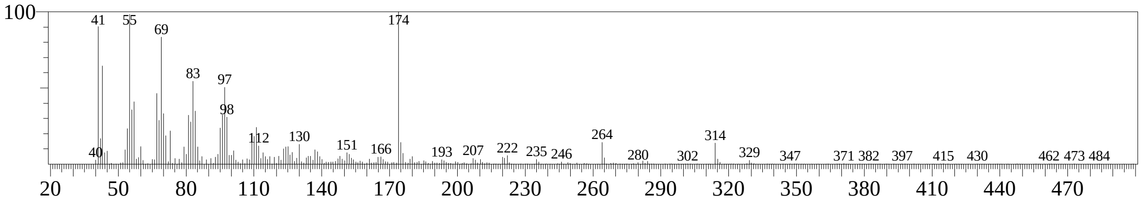

Hit#:1 Entry:90577 Library:NIST05.LIB
SI:89 Formula:C18H34O2 CAS:112-80-1 MolWeight:282 RetIndex:2175
CompName:Oleic Acid $$ 9-Octadecenoic acid (Z)- $$ .delta.(Sup9)-cis-Oleic acid $$ cis-.delta.(Sup9)-Octadecenoic acid $$ cis-C

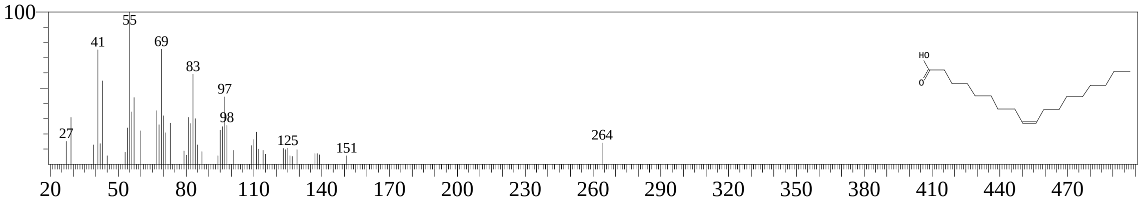

Hit#:2 Entry:90568 Library:NIST05.LIB
SI:88 Formula:C18H34O2 CAS:593-39-5 MolWeight:282 RetIndex:2175
CompName:6-Octadecenoic acid, (Z)- $$ (6Z)-6-Octadecenoic acid # $$

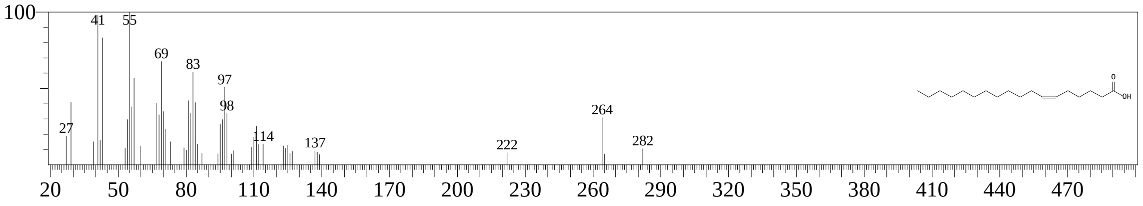

Hit#:3 Entry:65295 Library:NIST05.LIB
SI:87 Formula:C15H28O2 CAS:4727-18-8 MolWeight:240 RetIndex:2158
CompName:Cyclopentadecanone, 2-hydroxy- $$ 2-Hydroxycyclopentadecanone # $$

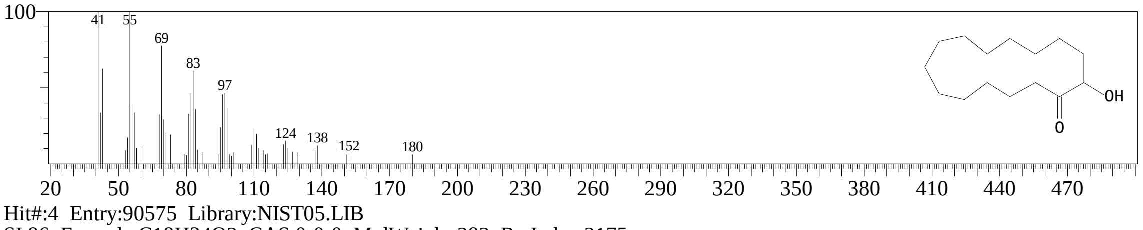

Hit#:4 Entry:90575 Library:NIST05.LIB
SI:86 Formula:C18H34O2 CAS:0-0-0 MolWeight:282 RetIndex:2175
CompName:Octadec-9-enoic acid

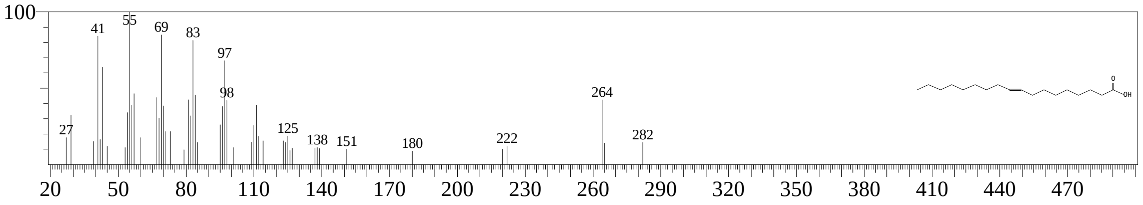

Hit#:5 Entry:22872 Library:NIST05s.LIB
SI:86 Formula:C18H34O2 CAS:112-79-8 MolWeight:282 RetIndex:2175
CompName:9-Octadecenoic acid, (E)- $$ trans-.delta.(sup 9)-Octadecenoic acid $$ trans-.delta.9-Octadecenoic acid $$ trans-Octad

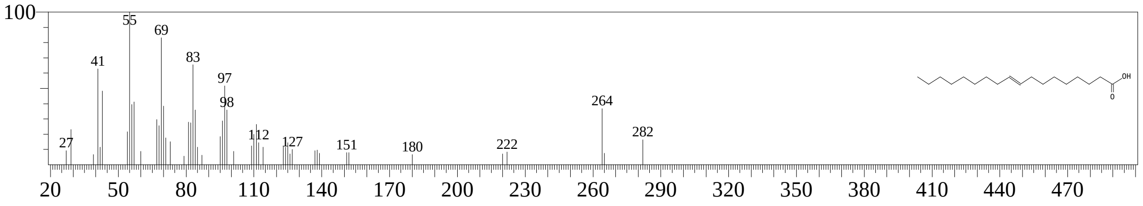

<< Target >>
Line#:20   R.Time:24.592(Scan#:2592) MassPeaks:279   BasePeak:73.00(4647)
RawMode:Averaged 24.583-24.600(2591-2593)   BG Mode:Calc. from Peak

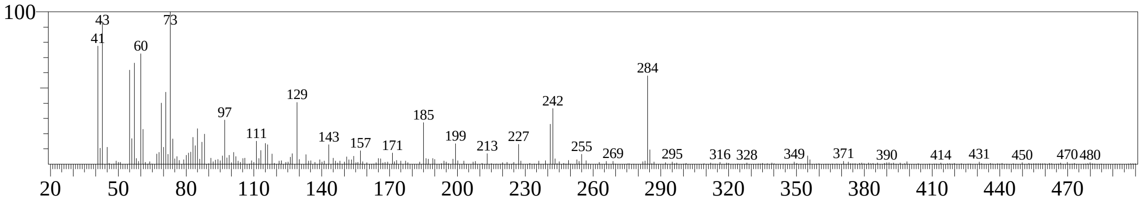

Hit#:1 Entry:22979 Library:NIST05s.LIB
SI:88 Formula:C18H36O2 CAS:57-11-4 MolWeight:284 RetIndex:2167
CompName:Octadecanoic acid $$ Stearic acid $$ n-Octadecanoic acid $$ Humko Industrene R $$ Hydrofol Acid 150 $$ Hystrene

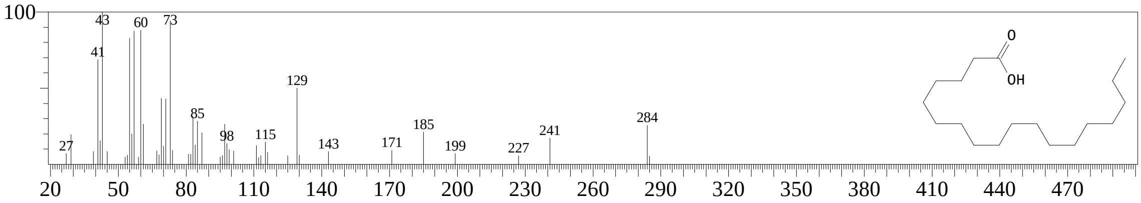

Hit#:2 Entry:91895 Library:NIST05.LIB
SI:87 Formula:C18H36O2 CAS:57-11-4 MolWeight:284 RetIndex:2167
CompName:Octadecanoic acid $$ Stearic acid $$ n-Octadecanoic acid $$ Humko Industrene R $$ Hydrofol Acid 150 $$ Hystrene

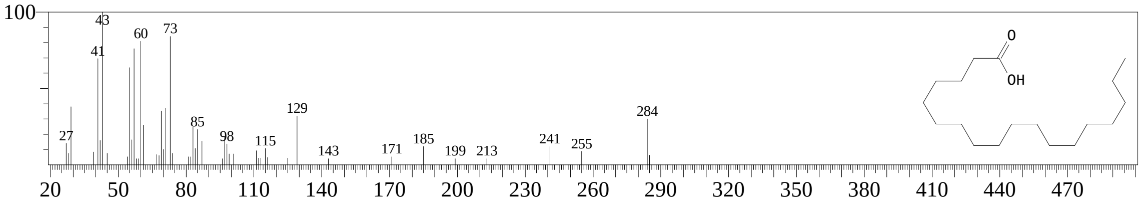

Hit#:3 Entry:20371 Library:NIST05s.LIB
SI:87 Formula:C15H30O2 CAS:1002-84-2 MolWeight:242 RetIndex:1869
CompName:Pentadecanoic acid $$ Pentadecylic acid $$ n-Pentadecanoic acid $$ n-Pentadecylic acid $$

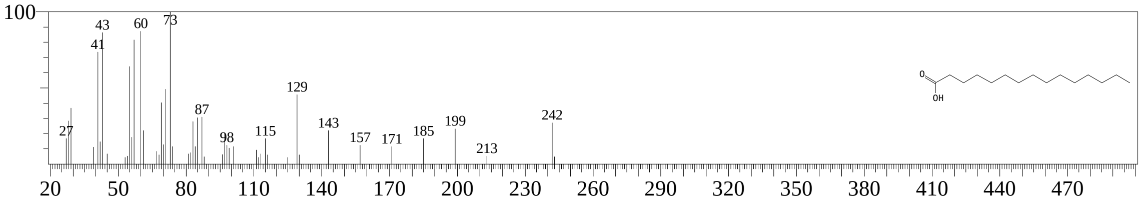

Hit#:4 Entry:22978 Library:NIST05s.LIB
SI:86 Formula:C18H36O2 CAS:57-11-4 MolWeight:284 RetIndex:2167
CompName:Octadecanoic acid $$ Stearic acid $$ n-Octadecanoic acid $$ Humko Industrene R $$ Hydrofol Acid 150 $$ Hystrene

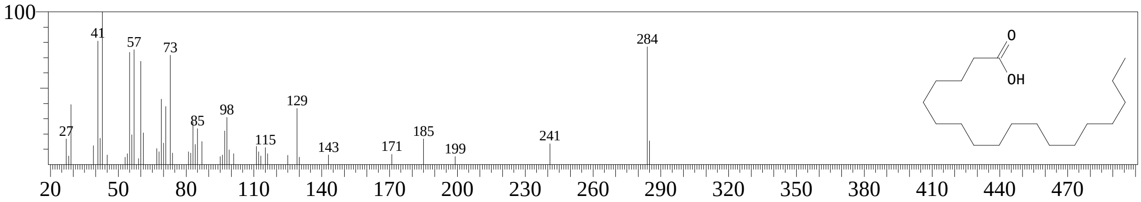

Hit#:5 Entry:22977 Library:NIST05s.LIB
SI:86 Formula:C18H36O2 CAS:57-11-4 MolWeight:284 RetIndex:2167
CompName:Octadecanoic acid $$ Stearic acid $$ n-Octadecanoic acid $$ Humko Industrene R $$ Hydrofol Acid 150 $$ Hystrene

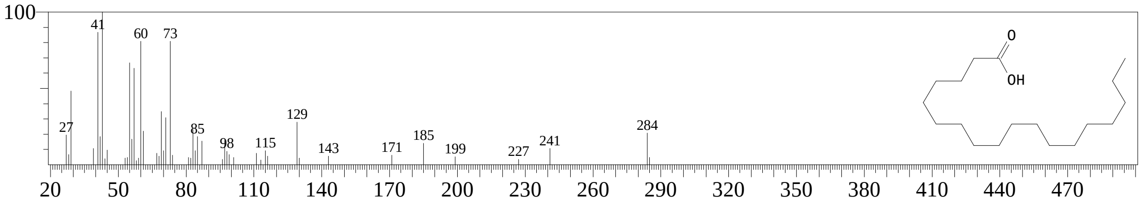

Supplement: Supplemental Information 2 [file peerj-07-6442-s002.pdf]
