# Supplementary material for: Comparative study on the composition of four different varieties of garlic
Source: PeerJ. 2019 Feb 21;7:e6442. doi: 10.7717/peerj.6442 (PMC6387757; doi:10.7717/peerj.6442)

Sample Information

Analyzed by : Admin
Analyzed : 2018-5-7 23:19:23
Sample Type : Unknown
Level # : 1
Sample Name : 2018-5-7-4-2
Sample ID :
IS Amount : [1]=1.000
Sample Amount : 1.000
Dilution Factor : 1.000
Vial # : 1
Injection Volume : 1.000
Data File : E:\陈存\GC-MS硫化物\2018-5-7-4-2.qgd
Org Data File : E:\刘春宏\2018-5-7-4-2.qgd
Method File : E:\刘春宏\2018-5-7-1.qgm
Org Method File : E:\刘春宏\2018-5-7-1.qgm
Report File :
Tuning File : C:\GCMSsolution\System\Tune1\2017-3-21.qgt
Modified by : Admin
Modified : 2018-5-7 23:48:23

Chromatogram 2018-5-7-4-2 E:\陈存\GC-MS硫化物\2018-5-7-4-2.qgd

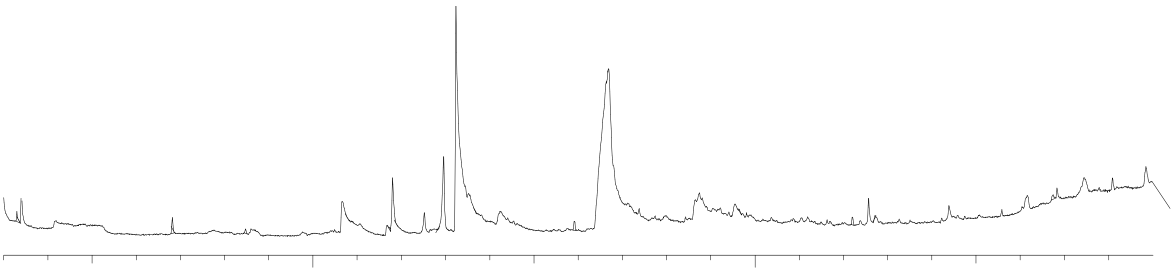

Peak Report TIC

Peak#	R.Time	I.Time	F.Time	Area	Area%	Height	Height%	A/H	Mark	Name
1	3.300	3.275	3.383	106994	0.37	55828	1.34	1.91		
2	6.817	6.783	6.867	172277	0.59	89051	2.14	1.93		
3	8.472	8.442	8.533	45330	0.16	24307	0.59	1.86		
4	10.662	10.608	10.808	917429	3.16	156361	3.76	5.86		
5	11.667	11.633	11.758	228135	0.79	50278	1.21	4.53		
6	11.800	11.758	11.917	958915	3.31	293235	7.06	3.27	V	
7	12.520	12.467	12.600	283801	0.98	103462	2.49	2.74		
8	12.954	12.858	13.017	1291975	4.46	397258	9.56	3.25		
9	13.234	13.192	13.492	7765811	26.78	1248326	30.05	6.22		
10	13.550	13.492	13.708	1013166	3.49	134574	3.24	7.52	V	
11	14.422	14.397	14.472	475092	1.64	55073	1.33	8.62		
12	15.915	15.875	15.950	105762	0.36	51888	1.25	2.03		
13	16.686	16.350	16.883	12993449	44.81	818285	19.70	15.87		
14	18.642	18.575	18.700	539470	1.86	98398	2.37	5.48		
15	18.741	18.700	18.792	582071	2.01	127829	3.08	4.55	V	
16	18.808	18.792	18.883	322597	1.11	89371	2.15	3.60	V	
17	18.917	18.883	18.950	67644	0.23	23736	0.57	2.84	V	
18	19.548	19.483	19.692	416238	1.44	61793	1.49	6.73		
19	22.203	22.175	22.242	81481	0.28	43909	1.06	1.85		
20	22.570	22.525	22.658	334072	1.15	139636	3.36	2.39		
21	24.387	24.342	24.458	209896	0.72	68997	1.66	3.04		
22	26.733	26.700	26.808	82626	0.28	22239	0.54	3.71		
				28994231	100.00	4153834	100.00			

Library

<< Target >>
Line#:1   R.Time:3.300(Scan#:37) MassPeaks:226   BasePeak:42.95(37650)
RawMode:Averaged 3.292-3.308(36-38)   BG Mode:Calc. from Peak

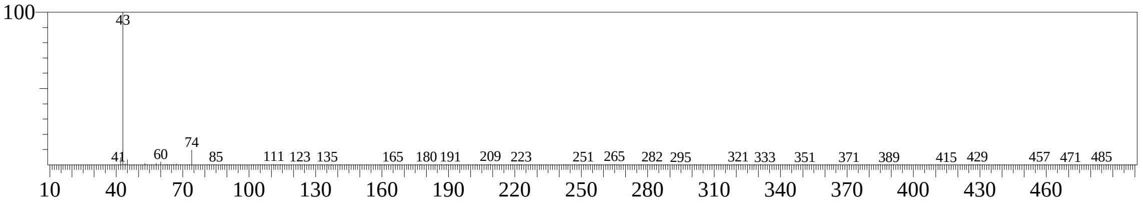

Hit#:1 Entry:368 Library:NIST05.LIB
SI:95 Formula:C3H6O2 CAS:116-9-6 MolWeight:74 RetIndex:698
CompName:2-Propanone, 1-hydroxy- $$ Acetol $$ CH3C(O)CH2OH $$ Hydroxyacetone $$ Acetone alcohol $$ Acetylcarbinol $$

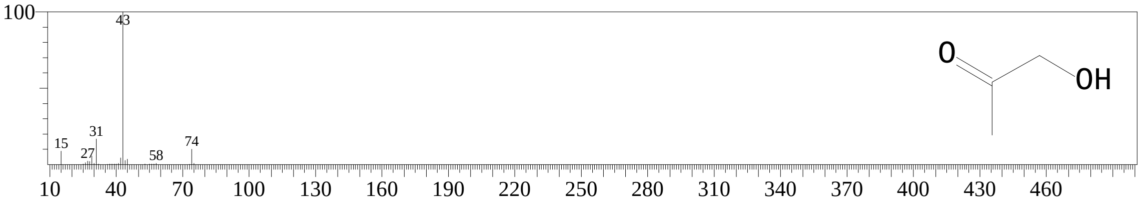

Hit#:2 Entry:415 Library:NIST05s.LIB
SI:94 Formula:C3H6O2 CAS:116-9-6 MolWeight:74 RetIndex:698
CompName:2-Propanone, 1-hydroxy- $$ Acetol $$ CH3C(O)CH2OH $$ Hydroxyacetone $$ Acetone alcohol $$ Acetylcarbinol $$

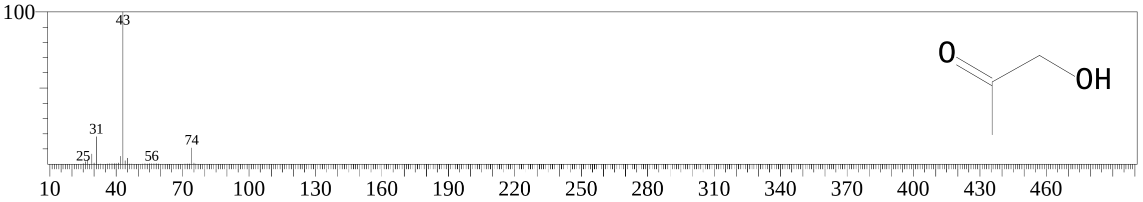

Hit#:3 Entry:414 Library:NIST05s.LIB
SI:93 Formula:C3H6O2 CAS:116-9-6 MolWeight:74 RetIndex:698
CompName:2-Propanone, 1-hydroxy- $$ Acetol $$ CH3C(O)CH2OH $$ Hydroxyacetone $$ Acetone alcohol $$ Acetylcarbinol $$

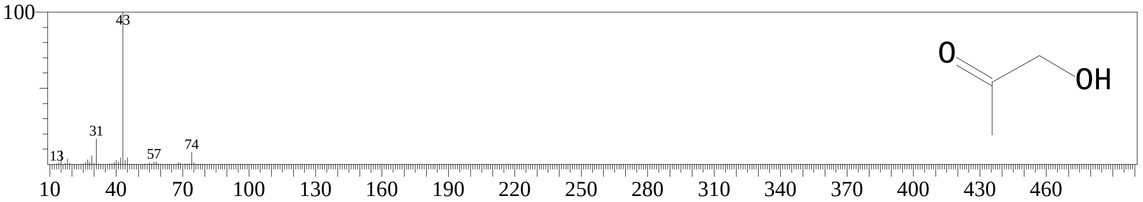

Hit#:4 Entry:1954 Library:NIST05s.LIB
SI:91 Formula:C4H6O3 CAS:108-24-7 MolWeight:102 RetIndex:722
CompName:Acetic anhydride $$ Acetic acid, anhydride $$ Acetic oxide $$ Acetyl anhydride $$ Acetyl ether $$ Acetyl oxide $$ Et

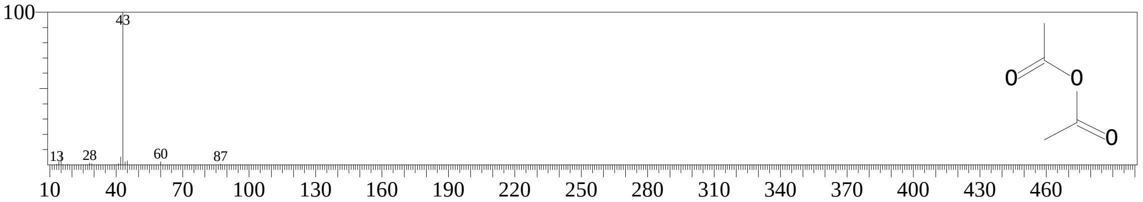

Hit#:5 Entry:1953 Library:NIST05s.LIB
SI:91 Formula:C4H6O3 CAS:108-24-7 MolWeight:102 RetIndex:722
CompName:Acetic anhydride $$ Acetic acid, anhydride $$ Acetic oxide $$ Acetyl anhydride $$ Acetyl ether $$ Acetyl oxide $$ Et

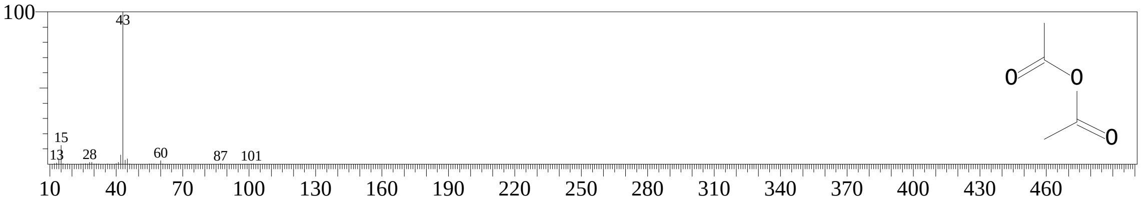

<< Target >>
Line#:2   R.Time:6.817(Scan#:459) MassPeaks:232   BasePeak:132.95(25200)
RawMode:Averaged 6.808-6.825(458-460)   BG Mode:Calc. from Peak

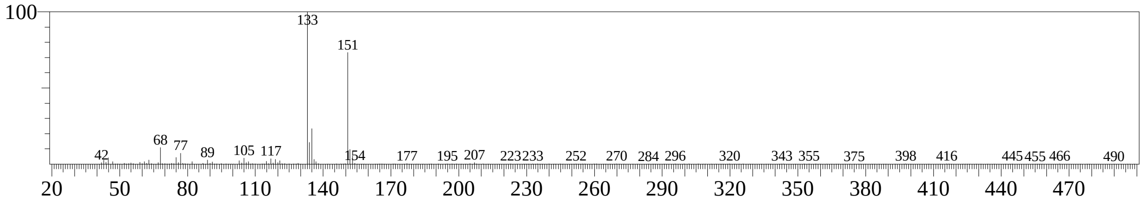

Hit#:1 Entry:15209 Library:NIST05.LIB
SI:85 Formula:C8H9NO2 CAS:0-0-0 MolWeight:151 RetIndex:1301
CompName:Oxime-, methoxy-phenyl-_

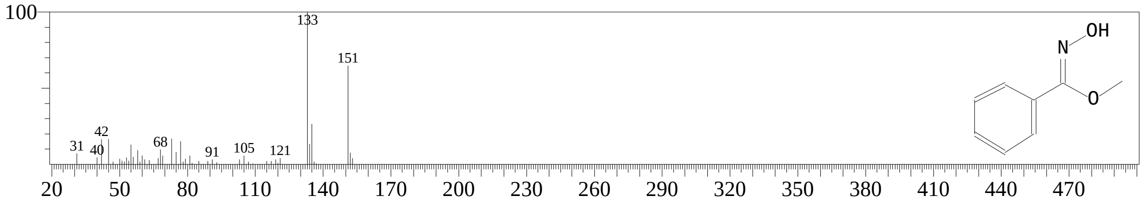

Hit#:2 Entry:51823 Library:NIST05.LIB
SI:79 Formula:C14H18O2 CAS:0-0-0 MolWeight:218 RetIndex:1714
CompName:4-Ethylbenzoic acid, cyclopentyl ester

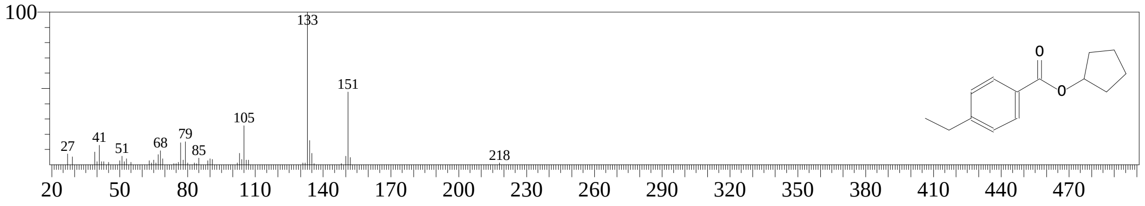

Hit#:3 Entry:15206 Library:NIST05.LIB
SI:79 Formula:C8H9NO2 CAS:2941-78-8 MolWeight:151 RetIndex:1575
CompName:2-Amino-5-methylbenzoic acid $$ 5-Methylanthranilic acid $$ Benzoic acid, 2-amino-5-methyl- $$ m-Toluic acid, 6-a

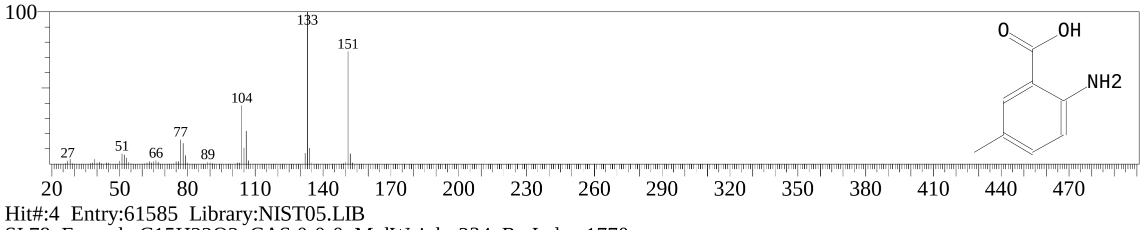

Hit#:4 Entry:61585 Library:NIST05.LIB
SI:78 Formula:C15H22O2 CAS:0-0-0 MolWeight:234 RetIndex:1770
CompName:4-Ethylbenzoic acid, hexyl ester

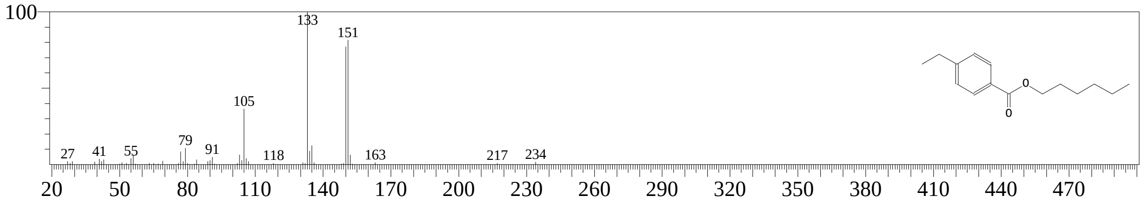

Hit#:5 Entry:44608 Library:NIST05.LIB
SI:77 Formula:C13H18O2 CAS:0-0-0 MolWeight:206 RetIndex:1507
CompName:4-Ethylbenzoic acid, 2-butyl ester

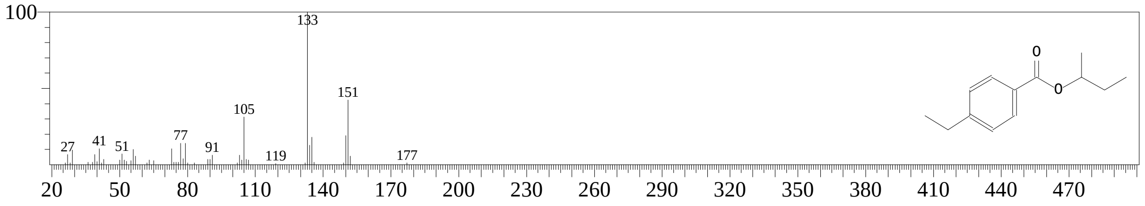

<< Target >>
Line#:3 R.Time:8.475(Scan#:658) MassPeaks:241 BasePeak:43.00(5010)
RawMode:Averaged 8.467-8.483(657-659) BG Mode:Calc. from Peak

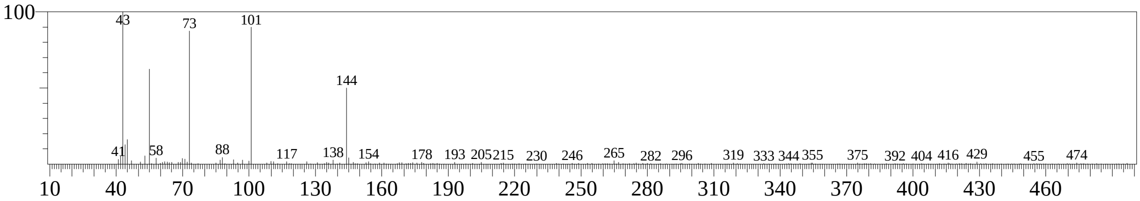

Hit#:1 Entry:12371 Library:NIST05.LIB
SI:86 Formula:C6H8O4 CAS:10230-62-3 MolWeight:144 RetIndex:1173
CompName:2,4-Dihydroxy-2,5-dimethyl-3(2H)-furan-3-one $$ 2,4-Dihydroxy-2,5-dimethyl-3(2H)-furanone # $$

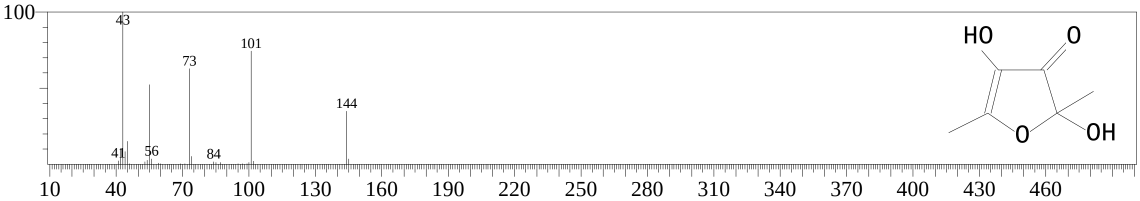

Hit#:2 Entry:7403 Library:NIST05s.LIB
SI:78 Formula:C6H8O4 CAS:28564-83-2 MolWeight:144 RetIndex:1269
CompName:4H-Pyran-4-one, 2,3-dihydro-3,5-dihydroxy-6-methyl- $$ 3,5-Dihydroxy-6-methyl-2,3-dihydro-4H-pyran-4-one # $$

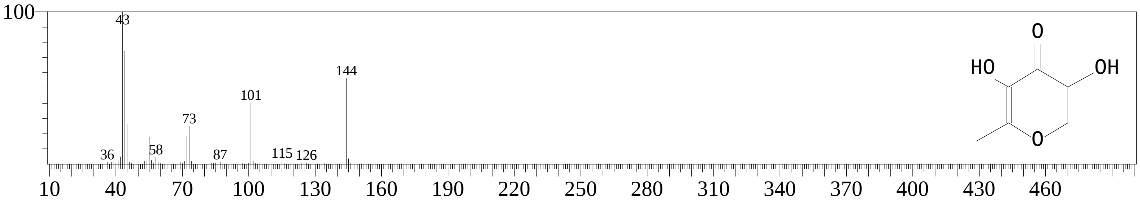

Hit#:3 Entry:12367 Library:NIST05.LIB
SI:75 Formula:C6H8O4 CAS:28564-83-2 MolWeight:144 RetIndex:1269
CompName:4H-Pyran-4-one, 2,3-dihydro-3,5-dihydroxy-6-methyl- $$ 3,5-Dihydroxy-6-methyl-2,3-dihydro-4H-pyran-4-one # $$

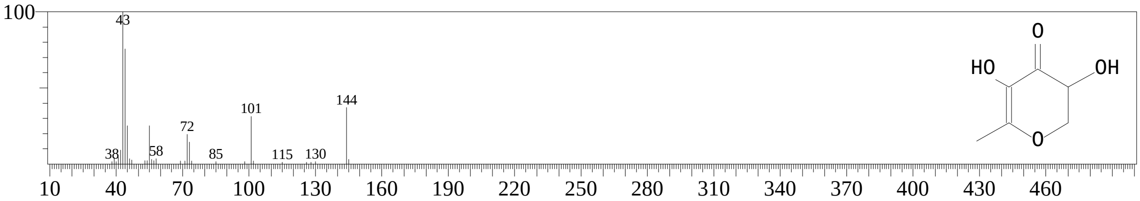

Hit#:4 Entry:33167 Library:NIST05.LIB
SI:74 Formula:C10H18O3 CAS:1522-34-5 MolWeight:186 RetIndex:1190
CompName:Pentanoic acid, 2-acetyl-4-methyl-, ethyl ester $$ Valeric acid, 2-acetyl-4-methyl-, ethyl ester $$ Ethyl 2-acetyl-4-methy

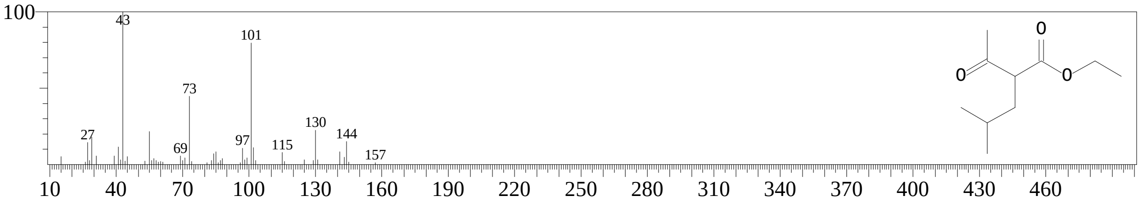

Hit#:5 Entry:33168 Library:NIST05.LIB
SI:73 Formula:C10H18O3 CAS:1540-29-0 MolWeight:186 RetIndex:1254
CompName:Hexanoic acid, 2-acetyl-, ethyl ester $$ Ethyl .alpha.-butylacetoacetate $$ Ethyl 2-acetylhexanoate $$ Ethyl 2-butylacet

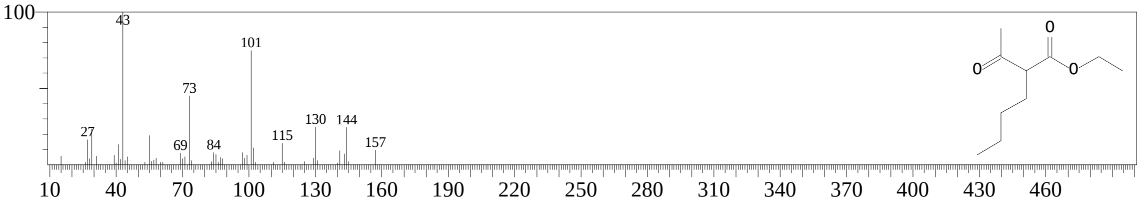

<< Target >>
Line#:4   R.Time:10.658(Scan#:920) MassPeaks:269   BasePeak:43.00(51947)
RawMode:Averaged 10.650-10.667(919-921)   BG Mode:Calc. from Peak

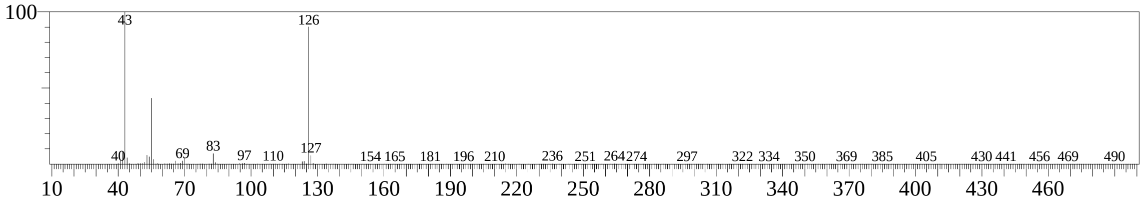

Hit#:1 Entry:4475 Library:NIST05s.LIB
SI:85 Formula:C3H6N6 CAS:108-78-1 MolWeight:126 RetIndex:1597
CompName:1,3,5-Triazine-2,4,6-triamine $$ Melamine $$ S-Triazinetriamine $$ Cyanuramide $$ Cyanurotriamide $$ Cyanurotria

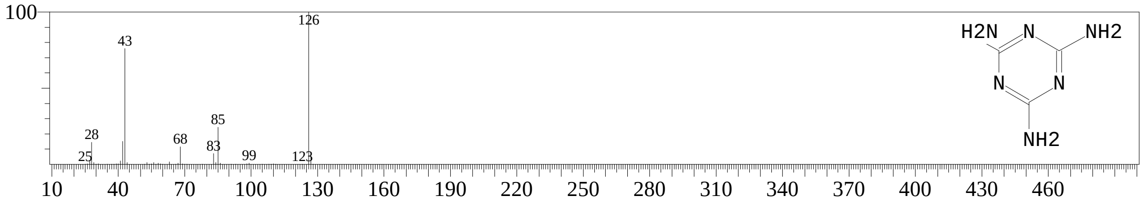

Hit#:2 Entry:6245 Library:NIST05.LIB
SI:82 Formula:C6H6O3 CAS:118-71-8 MolWeight:126 RetIndex:1063
CompName:Maltol $$ 4H-Pyran-4-one, 3-hydroxy-2-methyl- $$ Larixic acid $$ Larixinic acid $$ Palatone $$ 2-Methyl-3-hydroxyp

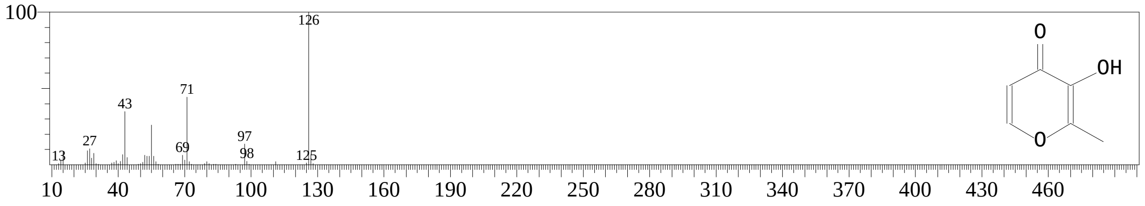

Hit#:3 Entry:4524 Library:NIST05s.LIB
SI:82 Formula:C6H6O3 CAS:118-71-8 MolWeight:126 RetIndex:1063
CompName:Maltol $$ 4H-Pyran-4-one, 3-hydroxy-2-methyl- $$ Larixic acid $$ Larixinic acid $$ Palatone $$ 2-Methyl-3-hydroxyp

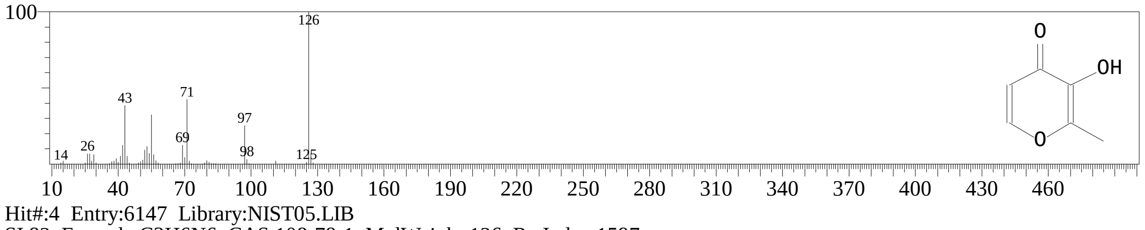

Hit#:4 Entry:6147 Library:NIST05.LIB
SI:82 Formula:C3H6N6 CAS:108-78-1 MolWeight:126 RetIndex:1597
CompName:1,3,5-Triazine-2,4,6-triamine $$ Melamine $$ S-Triazinetriamine $$ Cyanuramide $$ Cyanurotriamide $$ Cyanurotria

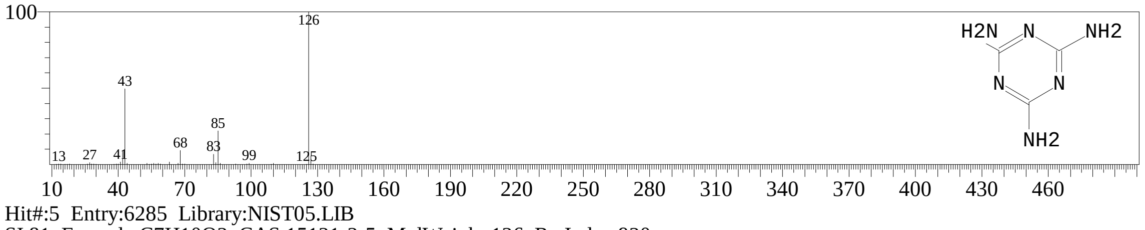

Hit#:5 Entry:6285 Library:NIST05.LIB
SI:81 Formula:C7H10O2 CAS:15121-2-5 MolWeight:126 RetIndex:920
CompName:Cyclopentane, 1-acetyl-1,2-epoxy- $$ 1-(6-Oxabicyclo[3.1.0]hex-1-yl)ethanone # $$

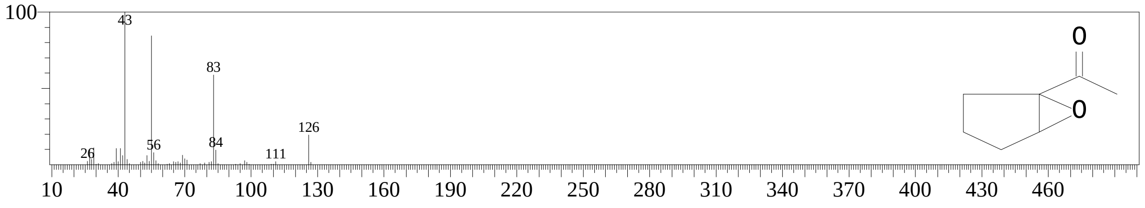

<< Target >>
Line#:5   R.Time:11.667(Scan#:1041) MassPeaks:252   BasePeak:43.00(14725)
RawMode:Averaged 11.658-11.675(1040-1042)   BG Mode:Calc. from Peak

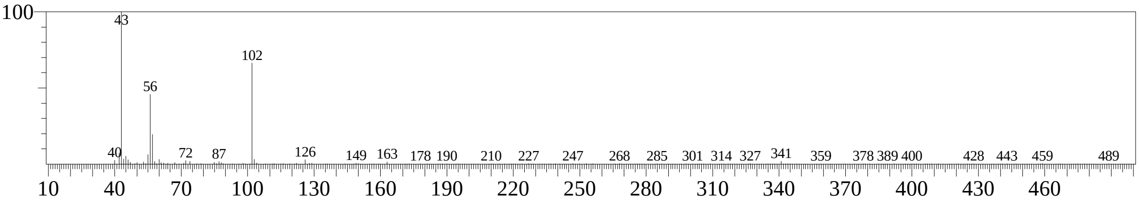

Hit#:1 Entry:3404 Library:NIST05s.LIB
SI:79 Formula:C5H8O3 CAS:123-76-2 MolWeight:116 RetIndex:1011
CompName:Pentanoic acid, 4-oxo- $$ Levulinic acid $$ .beta.-Acetylpropionic acid $$ .gamma.-Ketovaleric acid $$ Laevulinic aci

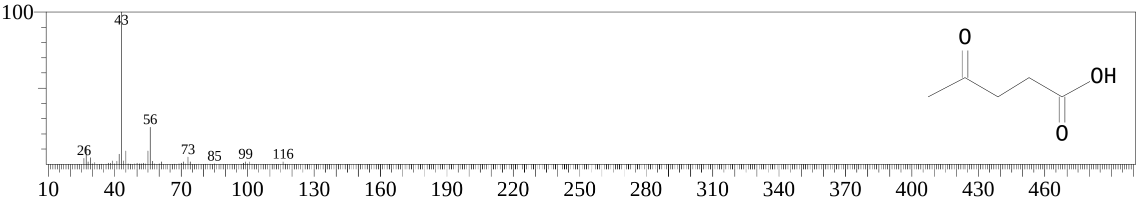

Hit#:2 Entry:4401 Library:NIST05.LIB
SI:79 Formula:C5H8O3 CAS:123-76-2 MolWeight:116 RetIndex:1011
CompName:Pentanoic acid, 4-oxo- $$ Levulinic acid $$ .beta.-Acetylpropionic acid $$ .gamma.-Ketovaleric acid $$ Laevulinic aci

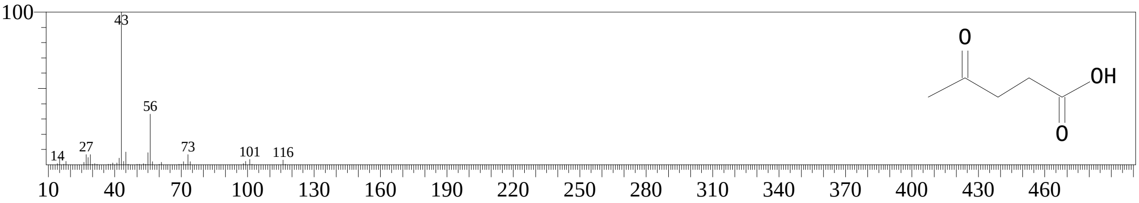

Hit#:3 Entry:3405 Library:NIST05s.LIB
SI:79 Formula:C5H8O3 CAS:123-76-2 MolWeight:116 RetIndex:1011
CompName:Pentanoic acid, 4-oxo- $$ Levulinic acid $$ .beta.-Acetylpropionic acid $$ .gamma.-Ketovaleric acid $$ Laevulinic aci

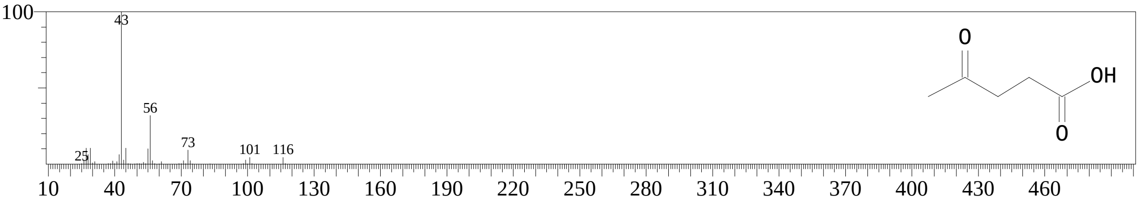

Hit#:4 Entry:7443 Library:NIST05s.LIB
SI:78 Formula:C7H12O3 CAS:609-14-3 MolWeight:144 RetIndex:956
CompName:Butanoic acid, 2-methyl-3-oxo-, ethyl ester $$ Acetoacetic acid, 2-methyl-, ethyl ester $$ .alpha.-Methylacetoacetic este

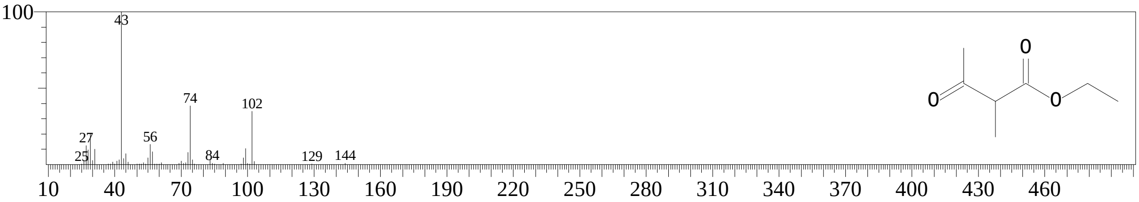

Hit#:5 Entry:7446 Library:NIST05s.LIB
SI:78 Formula:C7H12O3 CAS:609-14-3 MolWeight:144 RetIndex:956
CompName:Butanoic acid, 2-methyl-3-oxo-, ethyl ester $$ Acetoacetic acid, 2-methyl-, ethyl ester $$ .alpha.-Methylacetoacetic este

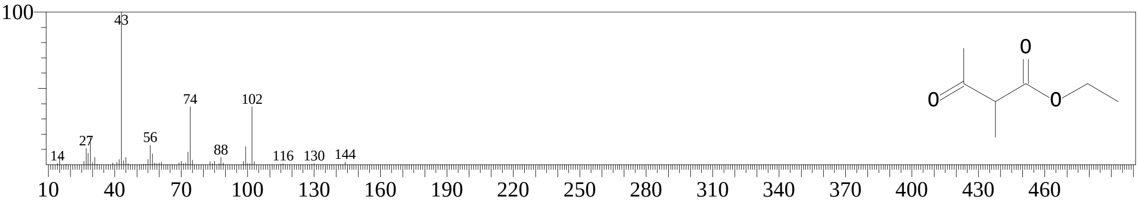

<< Target >>
Line#:6   R.Time:11.800(Scan#:1057) MassPeaks:314   BasePeak:43.00(46845)
RawMode:Averaged 11.792-11.808(1056-1058)   BG Mode:Calc. from Peak

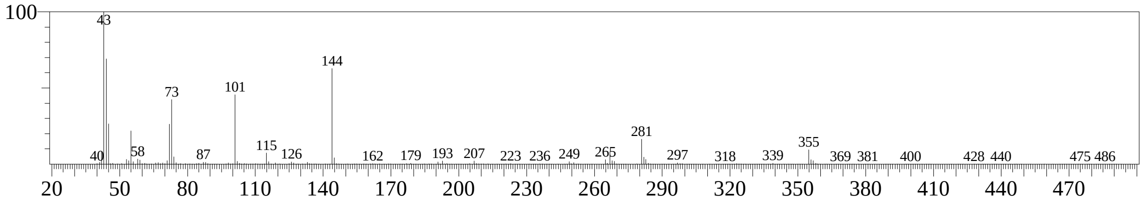

Hit#:1 Entry:7403 Library:NIST05s.LIB
SI:88 Formula:C6H8O4 CAS:28564-83-2 MolWeight:144 RetIndex:1269
CompName:4H-Pyran-4-one, 2,3-dihydro-3,5-dihydroxy-6-methyl- $$ 3,5-Dihydroxy-6-methyl-2,3-dihydro-4H-pyran-4-one # $$

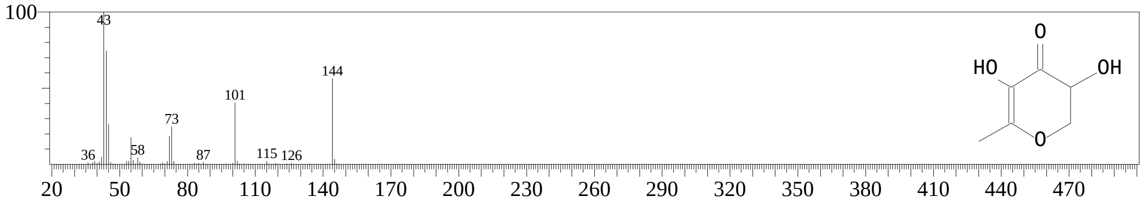

Hit#:2 Entry:12367 Library:NIST05.LIB
SI:84 Formula:C6H8O4 CAS:28564-83-2 MolWeight:144 RetIndex:1269
CompName:4H-Pyran-4-one, 2,3-dihydro-3,5-dihydroxy-6-methyl- $$ 3,5-Dihydroxy-6-methyl-2,3-dihydro-4H-pyran-4-one # $$

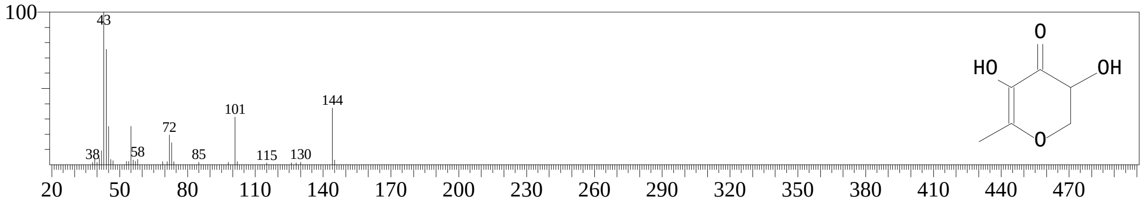

Hit#:3 Entry:4455 Library:NIST05.LIB
SI:77 Formula:C6H12O2 CAS:3299-32-9 MolWeight:116 RetIndex:761
CompName:1,3-Dioxolane, 2,4,5-trimethyl- $$ 2,4,5-Trimethyl-1,3-dioxolane # $$

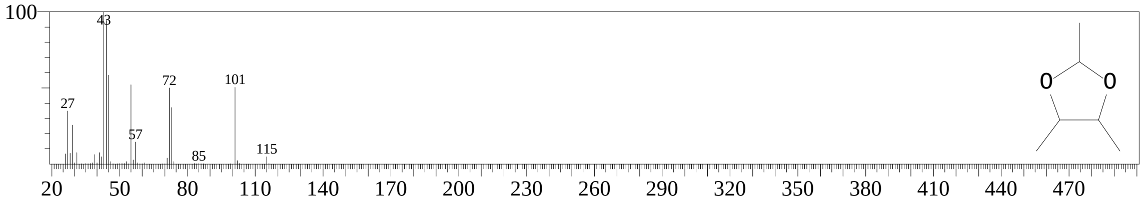

Hit#:4 Entry:12371 Library:NIST05.LIB
SI:76 Formula:C6H8O4 CAS:10230-62-3 MolWeight:144 RetIndex:1173
CompName:2,4-Dihydroxy-2,5-dimethyl-3(2H)-furan-3-one $$ 2,4-Dihydroxy-2,5-dimethyl-3(2H)-furanone # $$

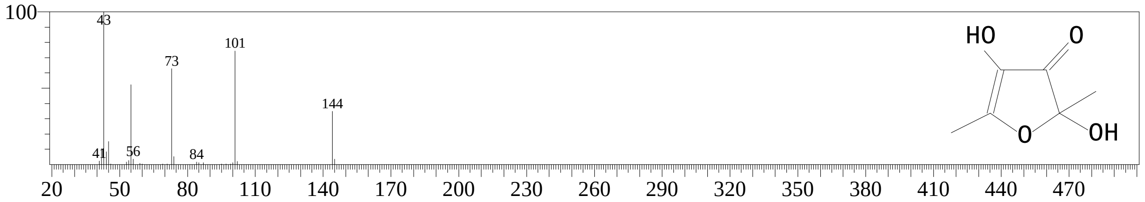

Hit#:5 Entry:12589 Library:NIST05.LIB
SI:72 Formula:C8H16O2 CAS:0-0-0 MolWeight:144 RetIndex:1156
CompName:2-Propyl-tetrahydropyran-3-ol

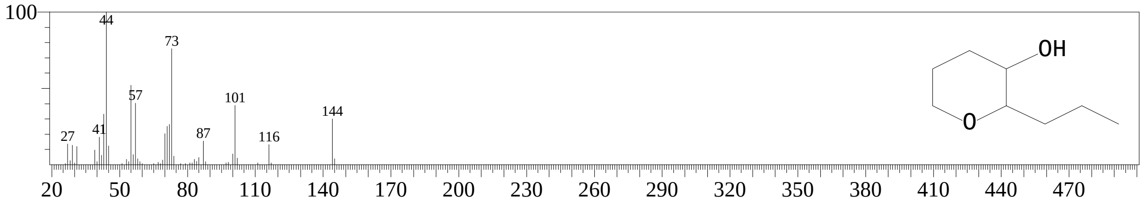

<< Target >>
Line#:7   R.Time:12.517(Scan#:1143) MassPeaks:250   BasePeak:110.95(11691)
RawMode:Averaged 12.508-12.525(1142-1144)   BG Mode:Calc. from Peak

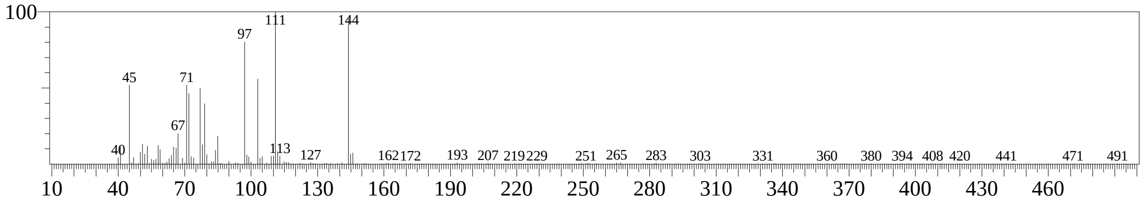

Hit#:1 Entry:12397 Library:NIST05.LIB
SI:86 Formula:C6H8S2 CAS:62488-52-2 MolWeight:144 RetIndex:1134
CompName:3-Vinyl-1,2-dithiacyclohex-4-ene $$ 3-Vinyl-3,6-dihydro-1,2-dithiine # $$

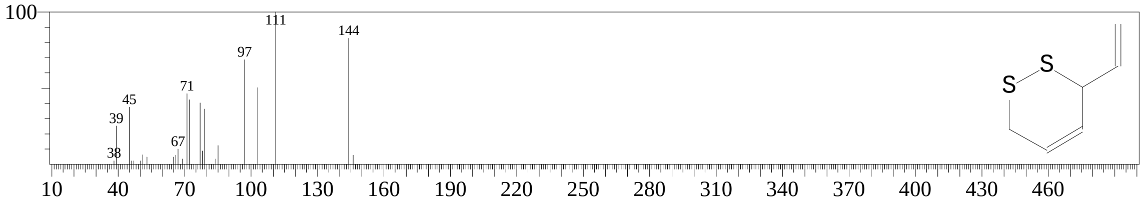

Hit#:2 Entry:3400 Library:NIST05.LIB
SI:72 Formula:C6H8S CAS:0-0-0 MolWeight:112 RetIndex:785
CompName:5-Thiatricyclo[4.1.0.0(2,4)]heptane

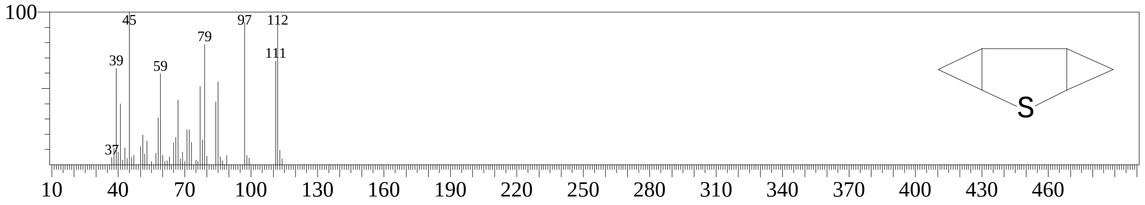

Hit#:3 Entry:3409 Library:NIST05.LIB
SI:71 Formula:C6H8S CAS:89533-93-7 MolWeight:112 RetIndex:894
CompName:Sulfide,1- propenyl 1-propynyl $$ (1E)-1-(1-Propynylsulfanyl)-1-propene # $$

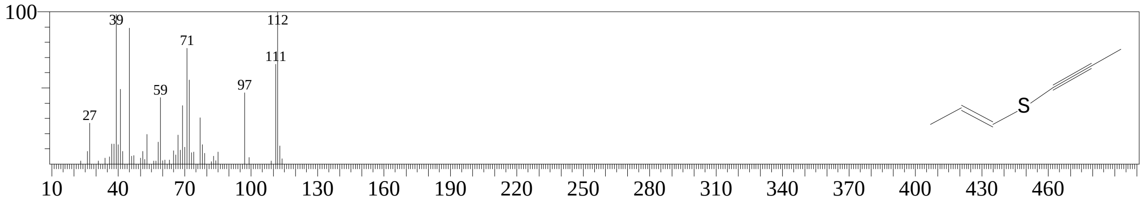

Hit#:4 Entry:12396 Library:NIST05.LIB
SI:70 Formula:C6H8S2 CAS:62488-53-3 MolWeight:144 RetIndex:1134
CompName:3-Vinyl-1,2-dithiacyclohex-5-ene $$ 3-Vinyl-3,4-dihydro-1,2-dithiine # $$

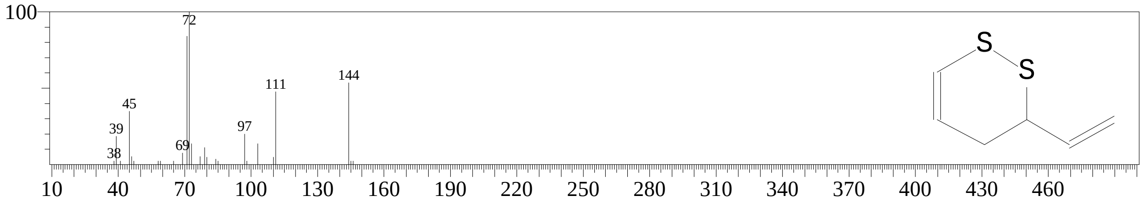

Hit#:5 Entry:3407 Library:NIST05.LIB
SI:67 Formula:C6H8S CAS:638-2-8 MolWeight:112 RetIndex:884
CompName:Thiophene, 2,5-dimethyl- $$ 2,5-Dimethylthiophene $$

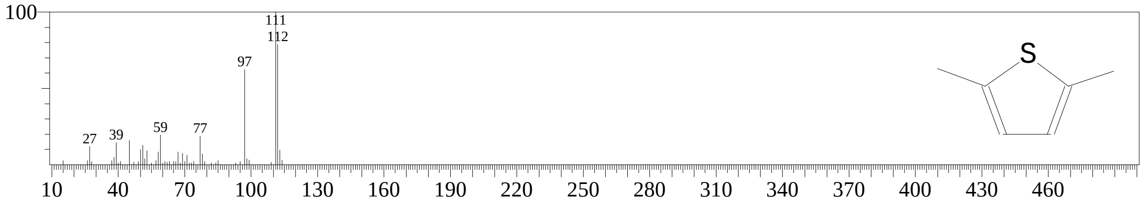

<< Target >>
Line#:8   R.Time:12.950(Scan#:1195) MassPeaks:275   BasePeak:71.95(61038)
RawMode:Averaged 12.942-12.958(1194-1196)   BG Mode:Calc. from Peak

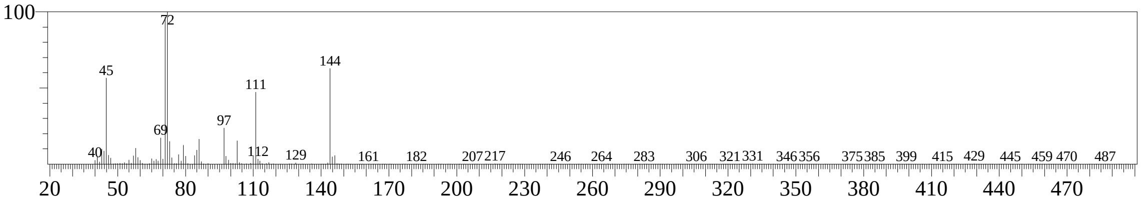

Hit#:1 Entry:12396 Library:NIST05.LIB
SI:87 Formula:C6H8S2 CAS:62488-53-3 MolWeight:144 RetIndex:1134
CompName:3-Vinyl-1,2-dithiacyclohex-5-ene $$ 3-Vinyl-3,4-dihydro-1,2-dithiine # $$

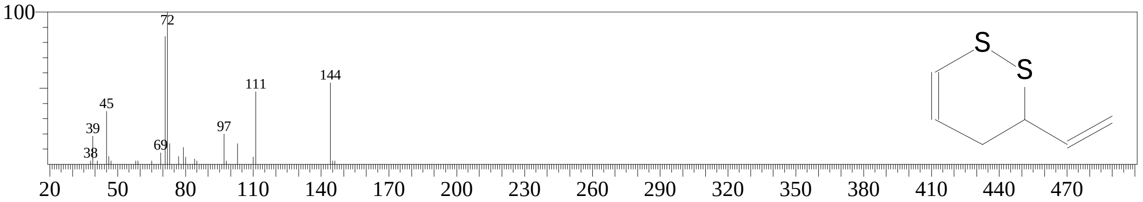

Hit#:2 Entry:12639 Library:NIST05.LIB
SI:74 Formula:C8H16O2 CAS:29887-60-3 MolWeight:144 RetIndex:993
CompName:Cyclohexane, 1,2-dimethoxy-, trans- $$ trans-1,2-Dimethoxycyclohexane $$ 1,2-Dimethoxycyclohexane # $$

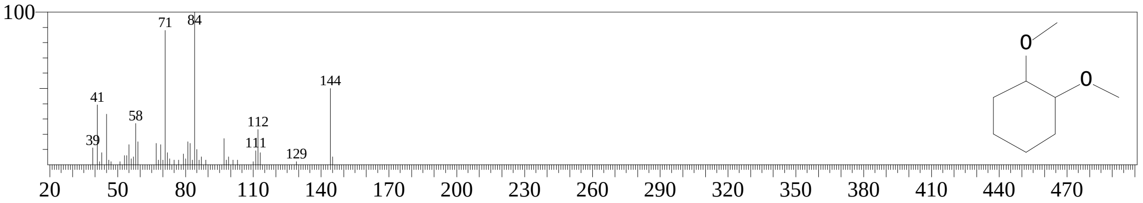

Hit#:3 Entry:12638 Library:NIST05.LIB
SI:73 Formula:C8H16O2 CAS:30363-80-5 MolWeight:144 RetIndex:993
CompName:Cyclohexane, 1,2-dimethoxy-, cis- $$ cis-1,2-Dimethoxycyclohexane $$ 1,2-Dimethoxycyclohexane # $$

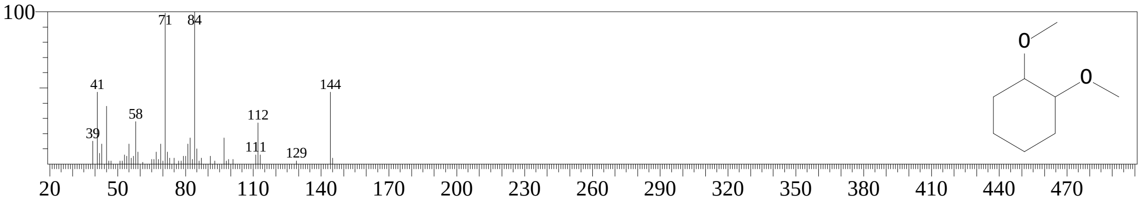

Hit#:4 Entry:12397 Library:NIST05.LIB
SI:72 Formula:C6H8S2 CAS:62488-52-2 MolWeight:144 RetIndex:1134
CompName:3-Vinyl-1,2-dithiacyclohex-4-ene $$ 3-Vinyl-3,6-dihydro-1,2-dithiine # $$

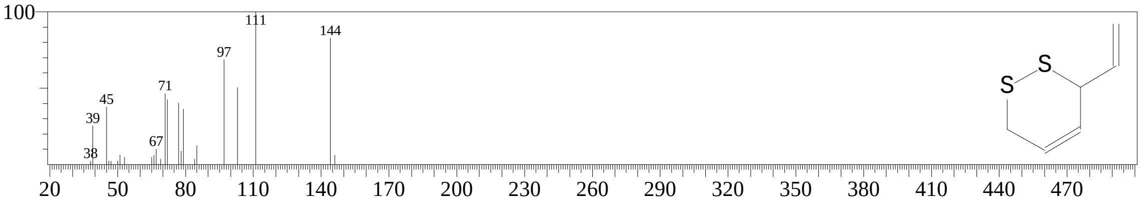

Hit#:5 Entry:3409 Library:NIST05.LIB
SI:70 Formula:C6H8S CAS:89533-93-7 MolWeight:112 RetIndex:894
CompName:Sulfide,1- propenyl 1-propynyl $$ (1E)-1-(1-Propynylsulfanyl)-1-propene # $$

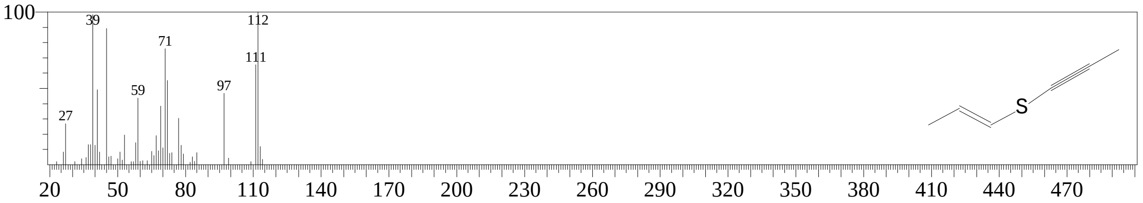

<< Target >>
Line#:9 R.Time:13.233(Scan#:1229) MassPeaks:275 BasePeak:97.00(281850)
RawMode:Averaged 13.225-13.242(1228-1230) BG Mode:Calc. from Peak

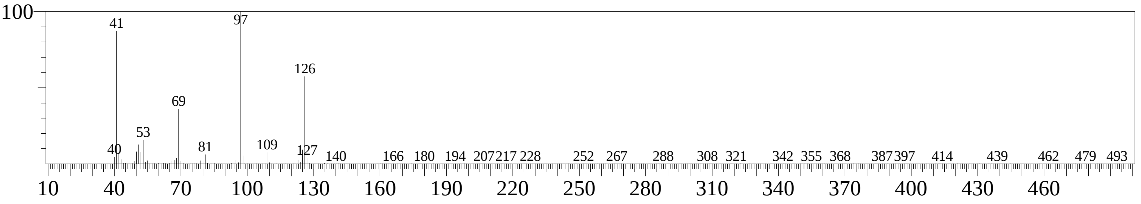

Hit#:1 Entry:6237 Library:NIST05.LIB
SI:95 Formula:C6H6O3 CAS:67-47-0 MolWeight:126 RetIndex:1163
CompName:2-Furancarboxaldehyde, 5-(hydroxymethyl)- $$ 2-Furaldehyde, 5-(hydroxymethyl)- $$ 5-Hydrxoymethylfurfural $$ Hy

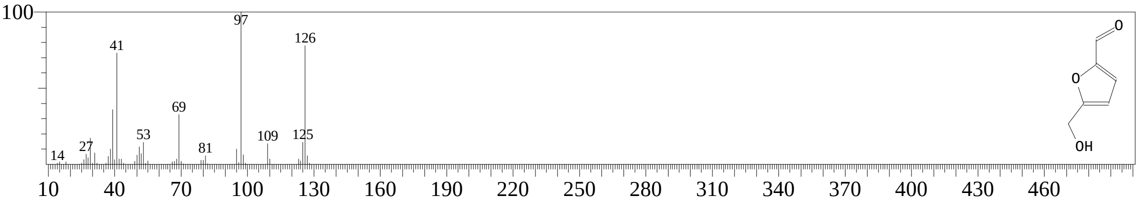

Hit#:2 Entry:4517 Library:NIST05s.LIB
SI:94 Formula:C6H6O3 CAS:67-47-0 MolWeight:126 RetIndex:1163
CompName:2-Furancarboxaldehyde, 5-(hydroxymethyl)- $$ 2-Furaldehyde, 5-(hydroxymethyl)- $$ 5-Hydrxoymethylfurfural $$ Hy

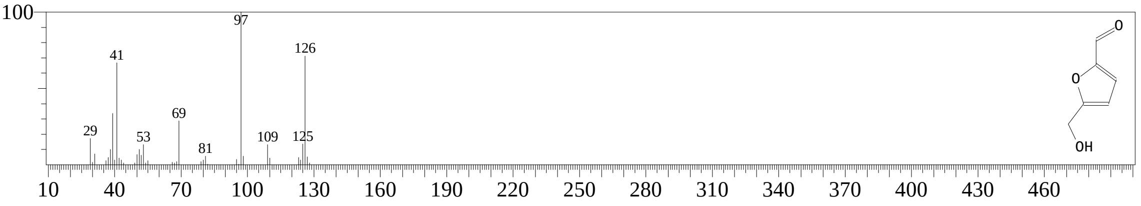

Hit#:3 Entry:6495 Library:NIST05.LIB
SI:85 Formula:C8H14O CAS:1447-26-3 MolWeight:126 RetIndex:938
CompName:4-Hepten-3-one, 5-methyl- $$ (4E)-5-Methyl-4-hepten-3-one # $$

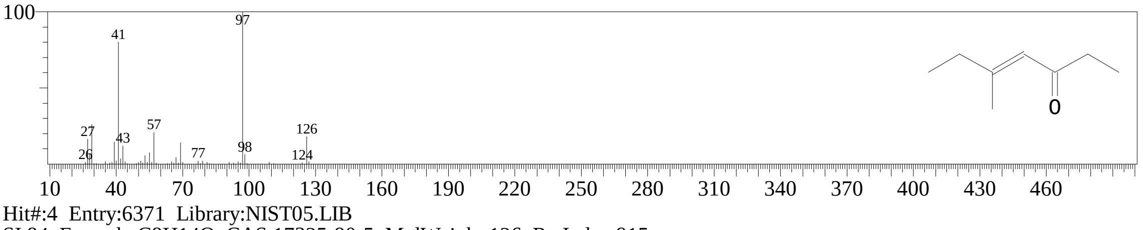

Hit#:4 Entry:6371 Library:NIST05.LIB
SI:84 Formula:C8H14O CAS:17325-90-5 MolWeight:126 RetIndex:915
CompName:4-Hexen-3-one, 4,5-dimethyl- $$ 4,5-Dimethyl-4-hexen-3-one # $$

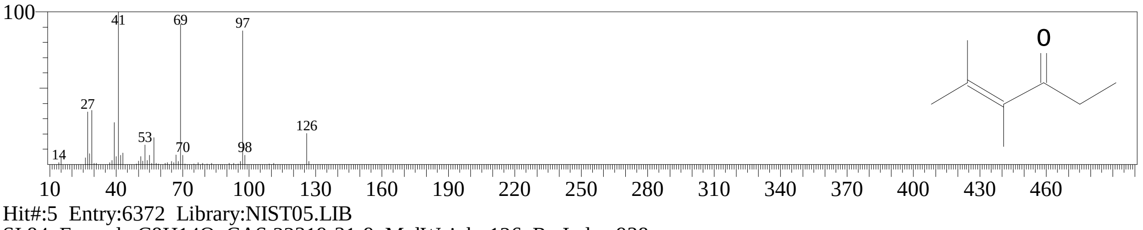

Hit#:5 Entry:6372 Library:NIST05.LIB
SI:84 Formula:C8H14O CAS:22319-31-9 MolWeight:126 RetIndex:938
CompName:4-Hepten-3-one, 4-methyl- $$ (4E)-4-Methyl-4-hepten-3-one # $$

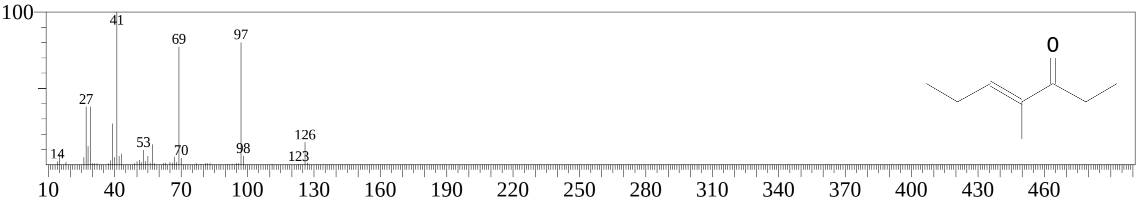

<< Target >>
Line#:10   R.Time:13.550(Scan#:1267) MassPeaks:232   BasePeak:43.00(27511)
RawMode:Averaged 13.542-13.558(1266-1268)   BG Mode:Calc. from Peak

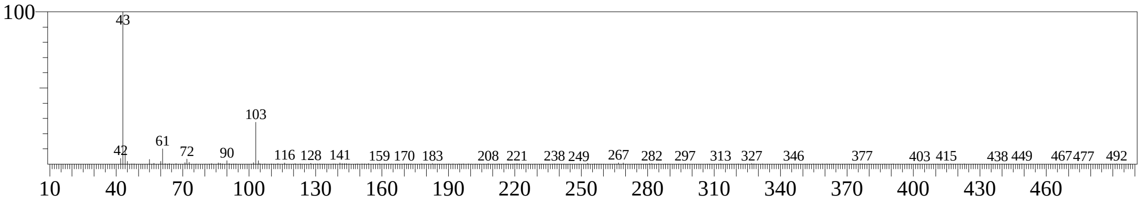

Hit#:1 Entry:5769 Library:NIST05s.LIB
SI:89 Formula:C5H10O4 CAS:26446-35-5 MolWeight:134 RetIndex:1091
CompName:1,2,3-Propanetriol, monoacetate $$ Acetin, mono- $$ Acetin $$ Acetoglyceride $$ Acetyl monoglyceride $$ Glycerin m

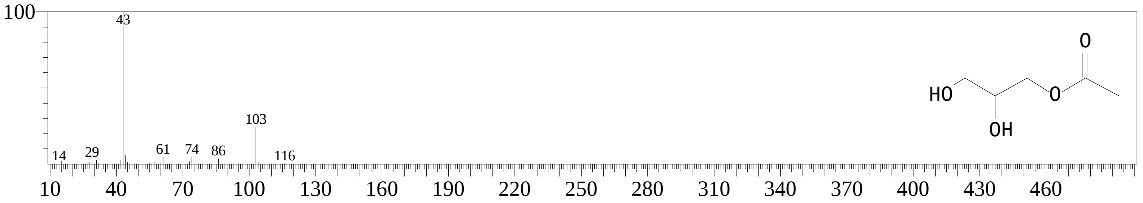

Hit#:2 Entry:8646 Library:NIST05.LIB
SI:89 Formula:C5H10O4 CAS:106-61-6 MolWeight:134 RetIndex:1091
CompName:1,2,3-Propanetriol, 1-acetate $$ Acetin, 1-mono- $$ .alpha.-Monoacetin $$ Glycerol .alpha.-monoacetate $$ 1-Monoac

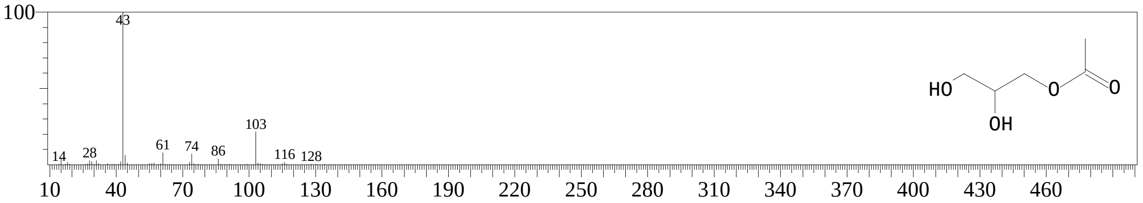

Hit#:3 Entry:8645 Library:NIST05.LIB
SI:88 Formula:C5H10O4 CAS:26446-35-5 MolWeight:134 RetIndex:1091
CompName:1,2,3-Propanetriol, monoacetate $$ Acetin, mono- $$ Acetin $$ Acetoglyceride $$ Acetyl monoglyceride $$ Glycerin m

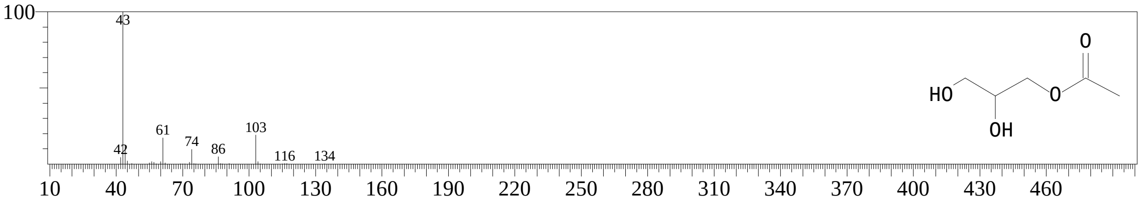

Hit#:4 Entry:42842 Library:NIST05.LIB
SI:85 Formula:C8H12O6 CAS:2983-35-9 MolWeight:204 RetIndex:1254
CompName:1,1,2-Triacetoxyethane $$ 1,1,2-Ethanetriol, triacetate $$ 1,2-Bis(acetyloxy)ethyl acetate # $$

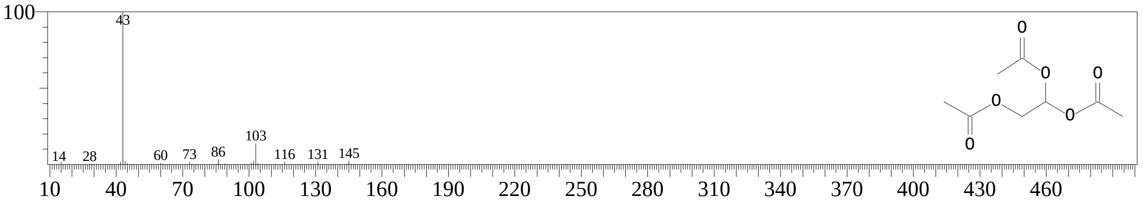

Hit#:5 Entry:12726 Library:NIST05s.LIB
SI:84 Formula:C7H12O5 CAS:25395-31-7 MolWeight:176 RetIndex:1230
CompName:1,2,3-Propanetriol, diacetate $$ Acetin, di- $$ Diacetin $$ Diacetylglycerol $$ Glycerin diacetate $$ Glycerine diacetat

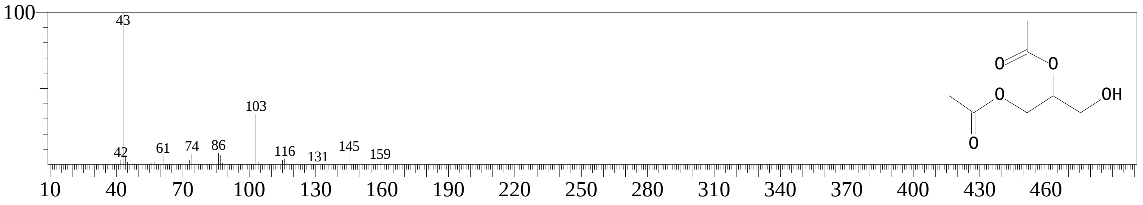

<< Target >>
Line#:11   R.Time:14.225(Scan#:1348) MassPeaks:262   BasePeak:43.00(17281)
RawMode:Averaged 14.217-14.233(1347-1349)   BG Mode:Calc. from Peak

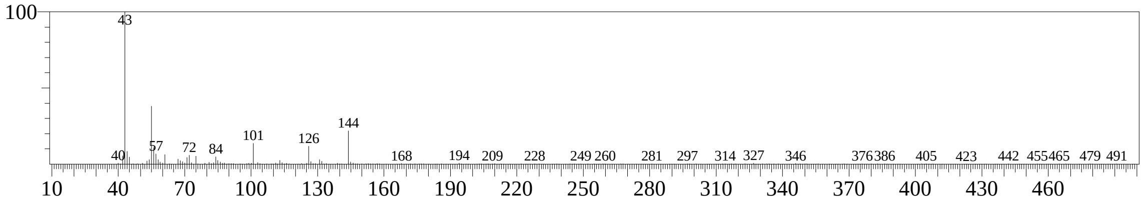

Hit#:1 Entry:12478 Library:NIST05.LIB
SI:79 Formula:C7H12O3 CAS:3128-7-2 MolWeight:144 RetIndex:1209
CompName:Heptanoic acid, 6-oxo- $$ 6-Oxoheptanoic acid # $$

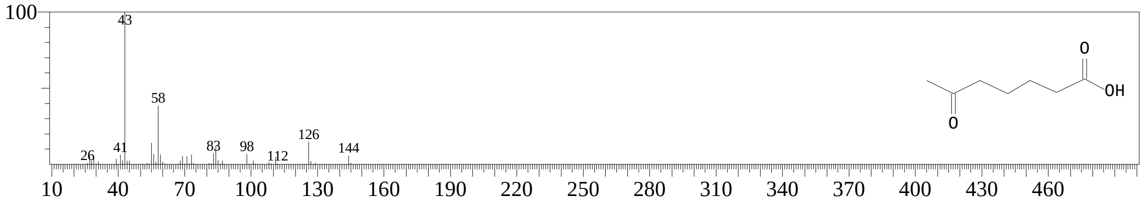

Hit#:2 Entry:68402 Library:NIST05.LIB
SI:76 Formula:C11H18O6 CAS:13431-59-9 MolWeight:246 RetIndex:1532
CompName:1,3-Propanediol, 2-hydroxymethyl-2-methyl-, triacetate $$ 3-(Acetyloxy)-2-[(acetyloxy)methyl]-2-methylpropyl acetate

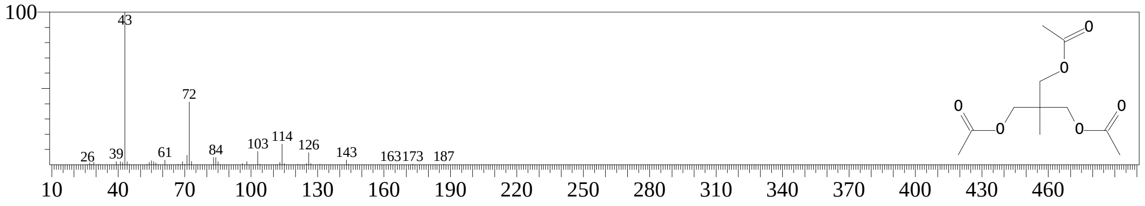

Hit#:3 Entry:12345 Library:NIST05.LIB
SI:76 Formula:C5H8N2O3 CAS:0-0-0 MolWeight:144 RetIndex:1313
CompName:N-Acetylcycloserine

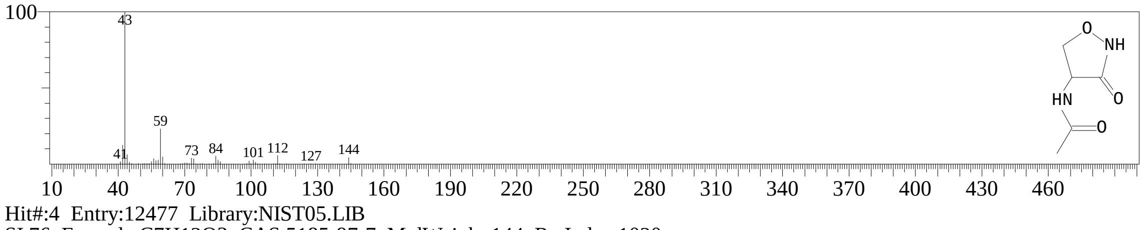

Hit#:4 Entry:12477 Library:NIST05.LIB
SI:76 Formula:C7H12O3 CAS:5185-97-7 MolWeight:144 RetIndex:1020
CompName:2-Pentanone, 5-(acetyloxy)- $$ 2-Pentanone, 5-hydroxy-, acetate $$ .gamma.-Acetylpropyl acetate $$ Acetopropyl acet

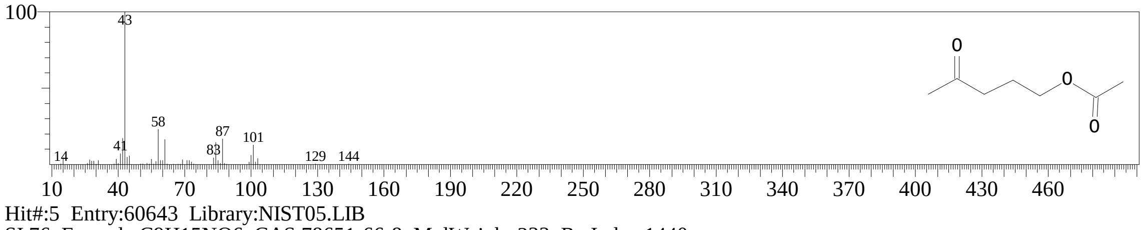

Hit#:5 Entry:60643 Library:NIST05.LIB
SI:76 Formula:C9H15NO6 CAS:78651-66-8 MolWeight:233 RetIndex:1440
CompName:2,4-Pentanediol, 3-nitro-, diacetate $$ 2,4-Di-O-acetyl-1,3,5-trideoxy-3-nitropentitol # $$

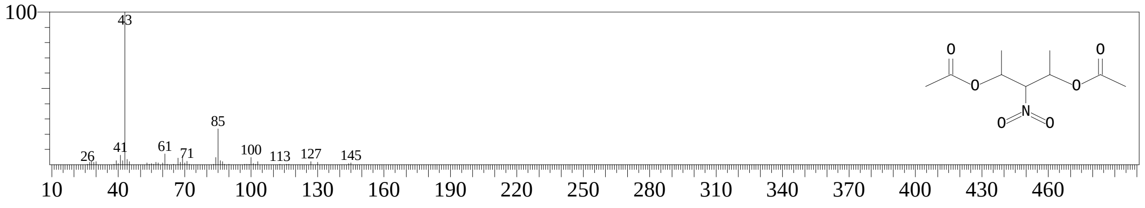

<< Target >>
Line#:12   R.Time:15.917(Scan#:1551) MassPeaks:273   BasePeak:41.00(4611)
RawMode:Averaged 15.908-15.925(1550-1552)   BG Mode:Calc. from Peak

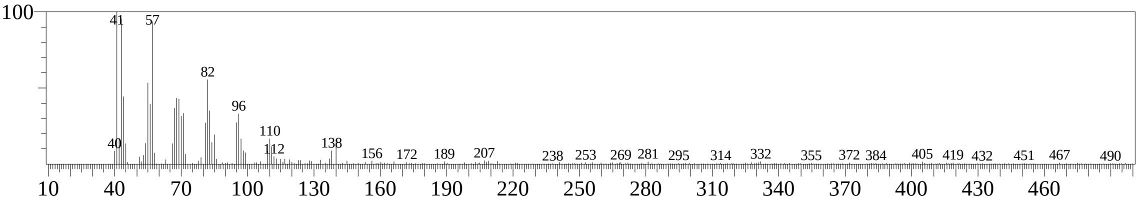

Hit#:1 Entry:14089 Library:NIST05s.LIB
SI:94 Formula:C12H24O CAS:112-54-9 MolWeight:184 RetIndex:1402
CompName:Dodecanal $$ Lauraldehyde $$ n-Dodecanal $$ n-Dodecyl aldehyde $$ n-Dodecylic aldehyde $$ n-Lauraldehyde $$ A

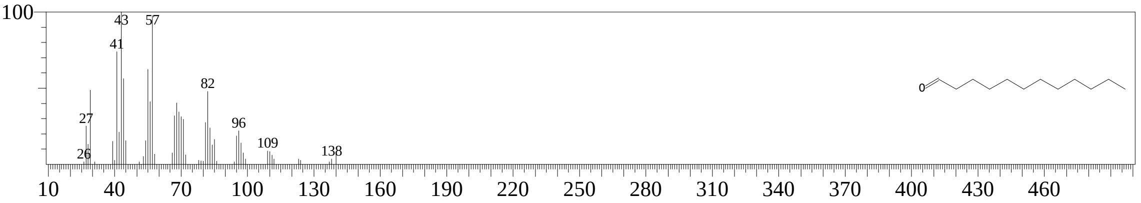

Hit#:2 Entry:14087 Library:NIST05s.LIB
SI:94 Formula:C12H24O CAS:112-54-9 MolWeight:184 RetIndex:1402
CompName:Dodecanal $$ Lauraldehyde $$ n-Dodecanal $$ n-Dodecyl aldehyde $$ n-Dodecylic aldehyde $$ n-Lauraldehyde $$ A

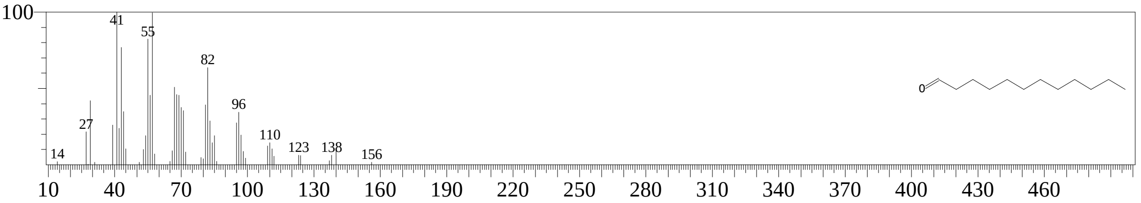

Hit#:3 Entry:17684 Library:NIST05s.LIB
SI:93 Formula:C14H28O CAS:124-25-4 MolWeight:212 RetIndex:1601
CompName:Tetradecanal $$ Myristaldehyde $$ Myristylaldehyde $$ Tetradecylaldehyde $$ n-Tetradecanal $$ Aldehyde C-14 $$ A

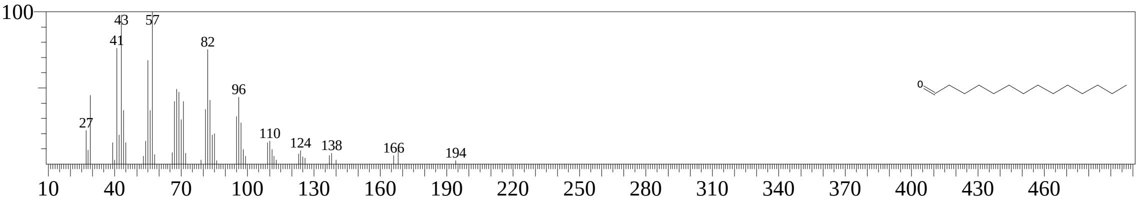

Hit#:4 Entry:40204 Library:NIST05.LIB
SI:93 Formula:C13H26O CAS:10486-19-8 MolWeight:198 RetIndex:1502
CompName:Tridecanal $$ n-Tridecylaldehyde $$ Tridecanaldehyde $$ Tridecyl aldehyde $$ 1-Tridecanal $$ Tridecane aldehyde $

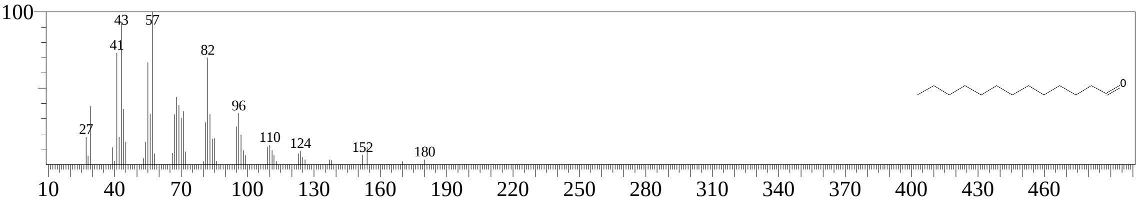

Hit#:5 Entry:65429 Library:NIST05.LIB
SI:92 Formula:C16H32O CAS:629-80-1 MolWeight:240 RetIndex:1800
CompName:Hexadecanal $$ Palmitaldehyde $$

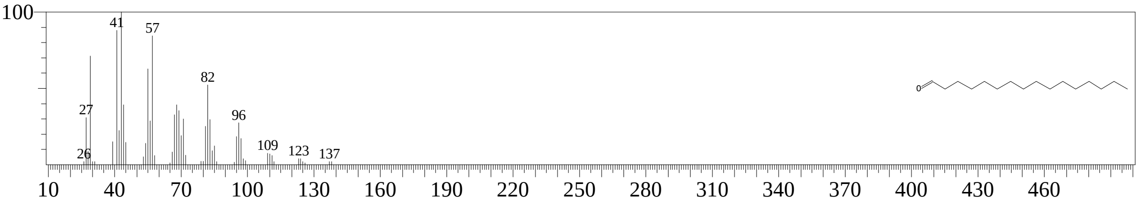

<< Target >>
Line#:13   R.Time:16.683(Scan#:1643) MassPeaks:272   BasePeak:57.00(180019)
RawMode:Averaged 16.675-16.692(1642-1644)   BG Mode:Calc. from Peak

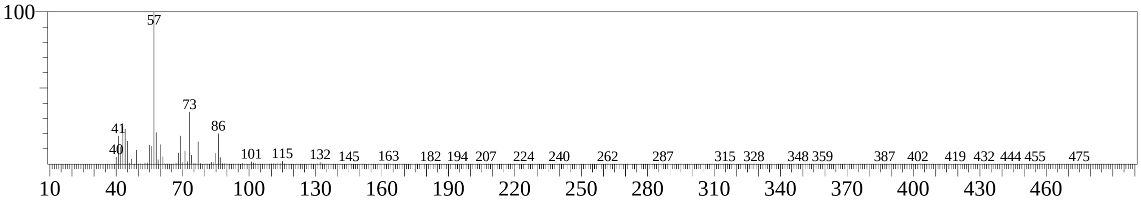

Hit#:1 Entry:8563 Library:NIST05s.LIB
SI:84 Formula:C4H9NO5 CAS:126-11-4 MolWeight:151 RetIndex:1444
CompName:1,3-Propanediol, 2-(hydroxymethyl)-2-nitro- $$ Isobutylglycerol, nitro- $$ Methane, trimethylolnitro- $$ Nitroisobutylg

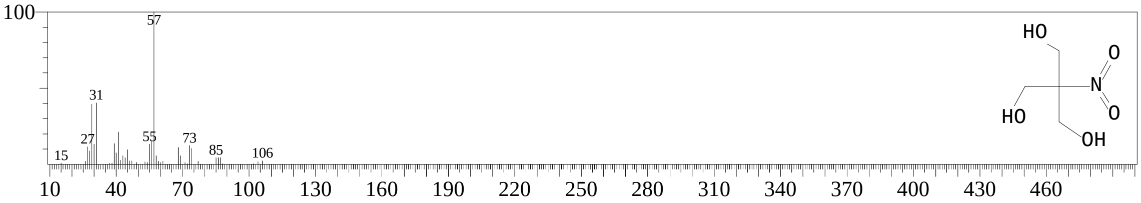

Hit#:2 Entry:15081 Library:NIST05.LIB
SI:82 Formula:C4H9NO5 CAS:126-11-4 MolWeight:151 RetIndex:1444
CompName:1,3-Propanediol, 2-(hydroxymethyl)-2-nitro- $$ Isobutylglycerol, nitro- $$ Methane, trimethylolnitro- $$ Nitroisobutylg

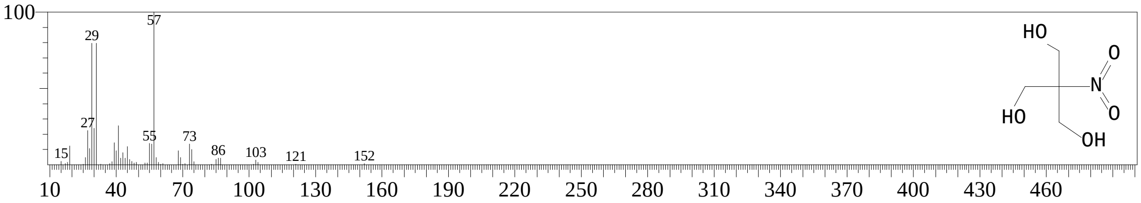

Hit#:3 Entry:123174 Library:NIST05.LIB
SI:82 Formula:C12H22O11 CAS:57-50-1 MolWeight:342 RetIndex:3139
CompName:Sucrose $$ .alpha.-D-Glucopyranoside, .beta.-D-fructofuranosyl $$ .beta.-D-Fructofuranosyl .alpha.-D-glucopyranosid

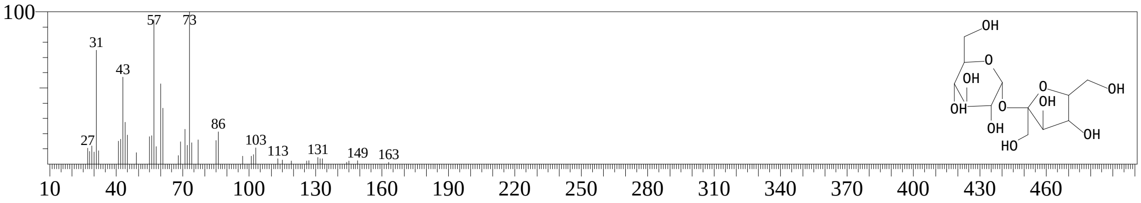

Hit#:4 Entry:5797 Library:NIST05s.LIB
SI:82 Formula:C6H14O3 CAS:77-99-6 MolWeight:134 RetIndex:1261
CompName:1,3-Propanediol, 2-ethyl-2-(hydroxymethyl)- $$ Ethriol $$ Ethyltrimethylolmethane $$ Etriol $$ Ettriol $$ Hexaglyceri

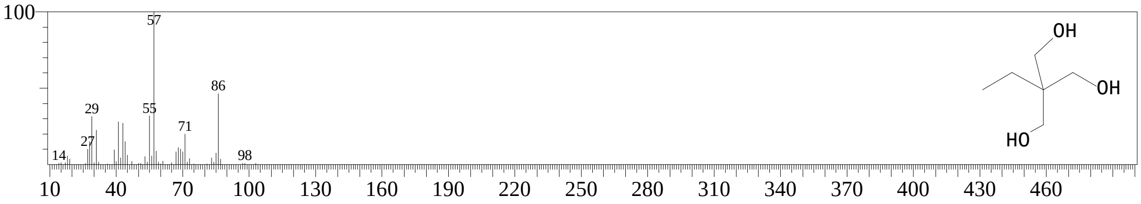

Hit#:5 Entry:13359 Library:NIST05.LIB
SI:81 Formula:C8H18O2 CAS:0-0-0 MolWeight:146 RetIndex:840
CompName:1-Isobutoxy-1-methoxypropane

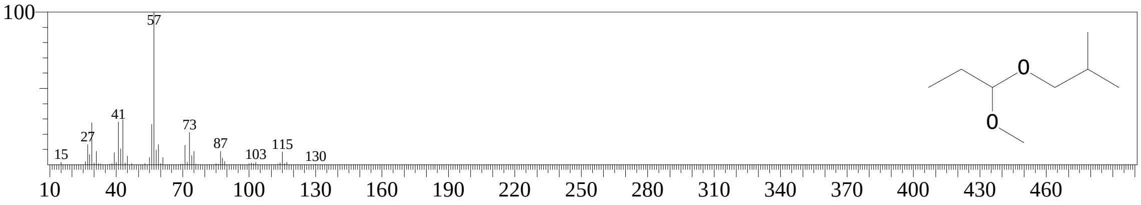

<< Target >>
Line#:14 R.Time:18.642(Scan#:1878) MassPeaks:257 BasePeak:57.00(5350)
RawMode:Averaged 18.633-18.650(1877-1879) BG Mode:Calc. from Peak

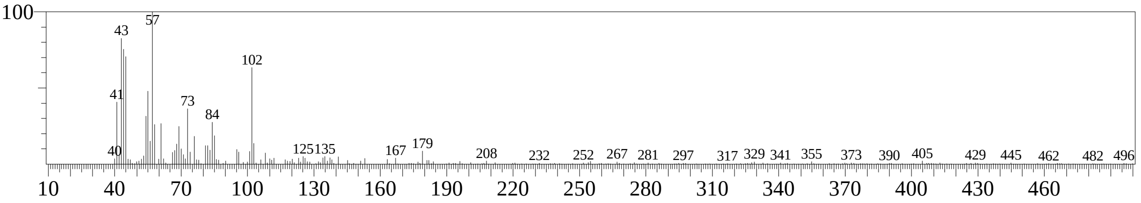

Hit#:1 Entry:20146 Library:NIST05.LIB
SI:83 Formula:C6H10O5 CAS:0-0-0 MolWeight:162 RetIndex:1625
CompName:3-Deoxy-d-mannoic lactone

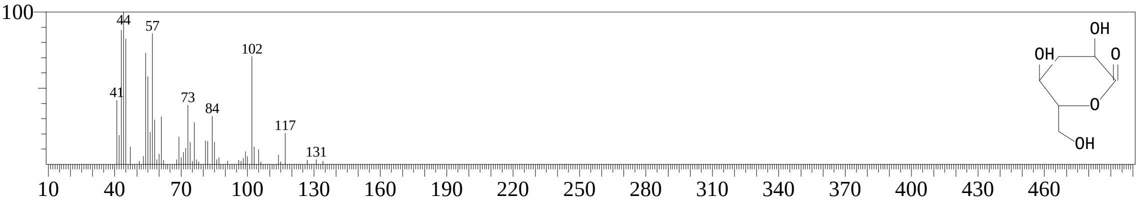

Hit#:2 Entry:29440 Library:NIST05.LIB
SI:79 Formula:C6H12O6 CAS:0-0-0 MolWeight:180 RetIndex:1704
CompName:3-Deoxy-d-mannonic acid

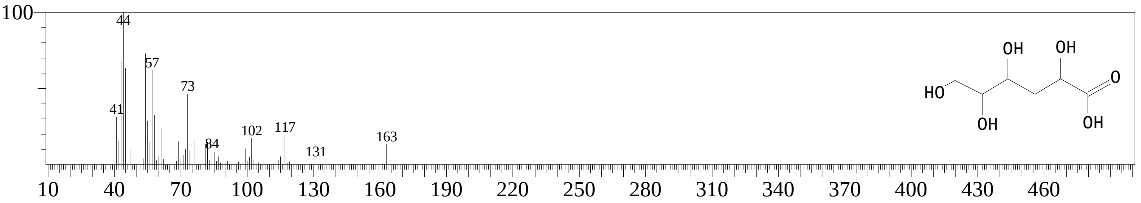

Hit#:3 Entry:7131 Library:NIST05.LIB
SI:74 Formula:C8H16O CAS:124-13-0 MolWeight:128 RetIndex:1005
CompName:Octanal $$ n-Caprylaldehyde $$ n-Octaldehyde $$ n-Octanal $$ n-Octylal $$ Antifoam-LF $$ Caprylaldehyde $$ Capı

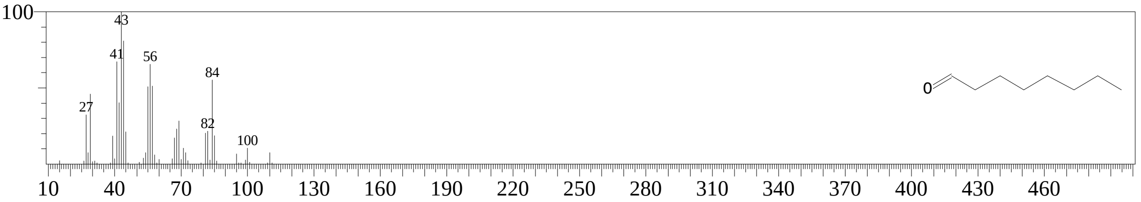

Hit#:4 Entry:4912 Library:NIST05s.LIB
SI:74 Formula:C8H16O CAS:124-13-0 MolWeight:128 RetIndex:1005
CompName:Octanal $$ n-Caprylaldehyde $$ n-Octaldehyde $$ n-Octanal $$ n-Octylal $$ Antifoam-LF $$ Caprylaldehyde $$ Capı

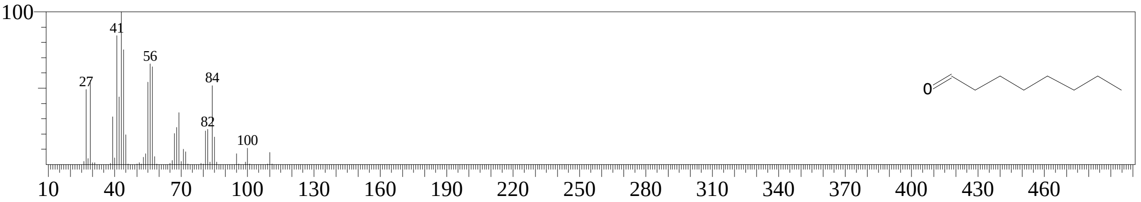

Hit#:5 Entry:7621 Library:NIST05.LIB
SI:74 Formula:C6H10O3 CAS:14241-58-8 MolWeight:130 RetIndex:1028
CompName:1,6-Anhydro-2,4-dideoxy-.beta.-D-arabo-hexopyranose

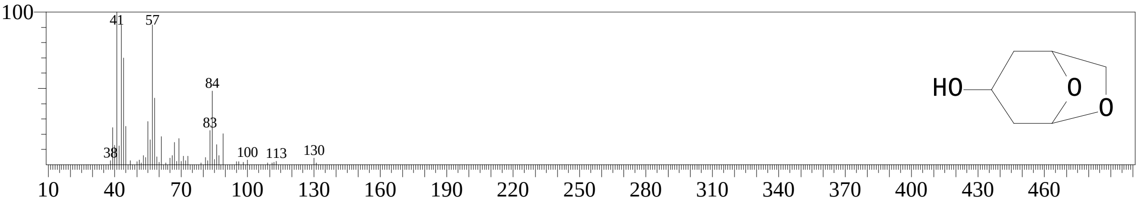

<< Target >>
Line#:15   R.Time:18.742(Scan#:1890) MassPeaks:245   BasePeak:156.00(12330)
RawMode:Averaged 18.733-18.750(1889-1891)   BG Mode:Calc. from Peak

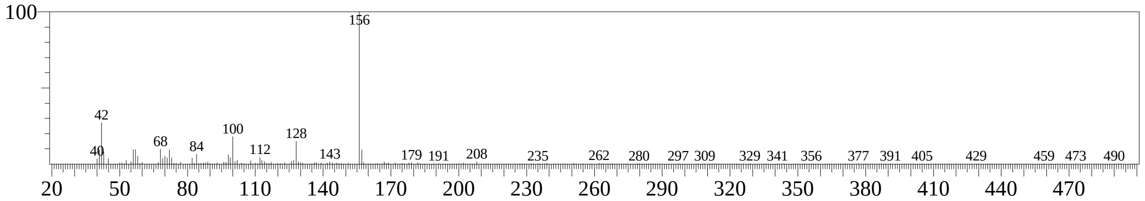

Hit#:1 Entry:25287 Library:NIST05.LIB
SI:78 Formula:$C_{10}H_{21}NO$ CAS:0-0-0 MolWeight:171 RetIndex:1278
CompName:Piperidin-4-ol, 2,5-dimethyl-1-(1-methylethyl)-

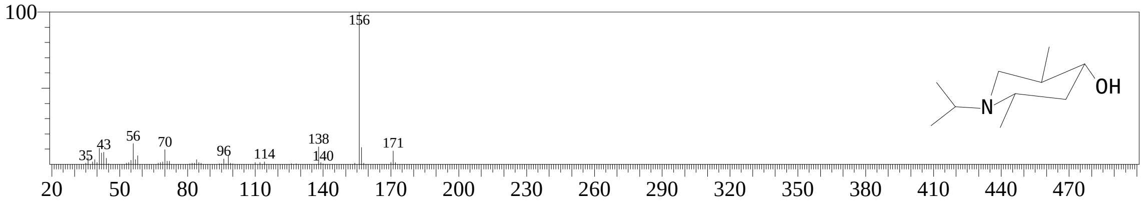

Hit#:2 Entry:9607 Library:NIST05s.LIB
SI:77 Formula:$C_6H_8N_2O_3$ CAS:769-42-6 MolWeight:156 RetIndex:1532
CompName:1,3-Dimethylbarbituric acid $$ 2,4,6(1H,3H,5H)-Pyrimidinetrione, 1,3-dimethyl- $$ Barbituric acid, 1,3-dimethyl- $$ [illegible]

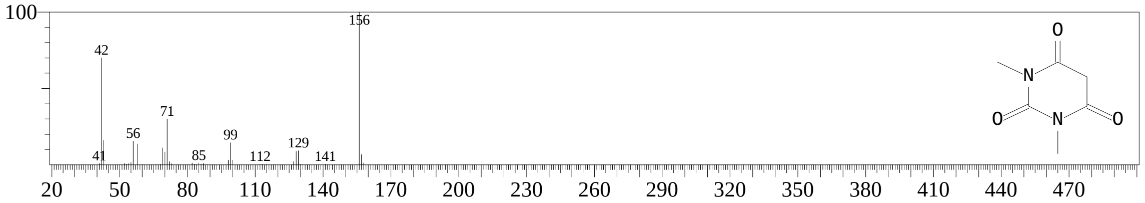

Hit#:3 Entry:49985 Library:NIST05.LIB
SI:76 Formula:$C_{12}H_{25}NO_2$ CAS:6141-46-4 MolWeight:215 RetIndex:1495
CompName:Norleucine, 2-butyl-N-methyl-, methyl ester $$ Methyl 2-butyl-2-(methylamino)hexanoate # $$

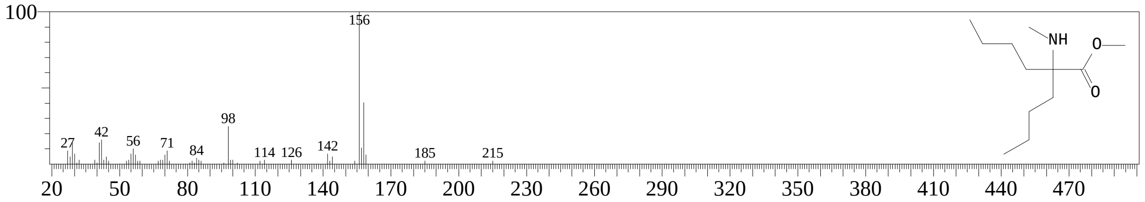

Hit#:4 Entry:21263 Library:NIST05s.LIB
SI:74 Formula:$C_{17}H_{37}N$ CAS:4455-26-9 MolWeight:255 RetIndex:1760
CompName:1-Octanamine, N-methyl-N-octyl- $$ Di(octyl)methylamine $$ N-Methyldioctylamine $$ N,N-Dioctylmethylamine $$

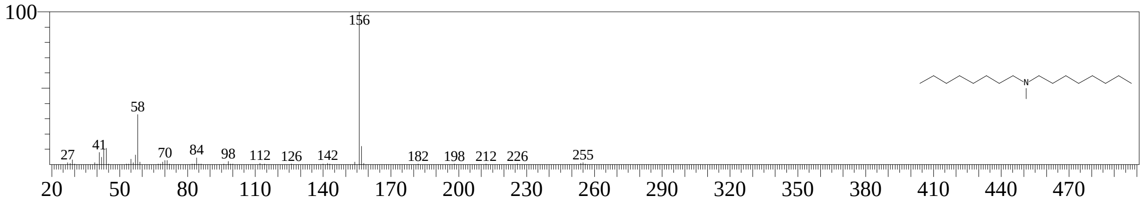

Hit#:5 Entry:21261 Library:NIST05s.LIB
SI:74 Formula:$C_{17}H_{37}N$ CAS:4455-26-9 MolWeight:255 RetIndex:1760
CompName:1-Octanamine, N-methyl-N-octyl- $$ Di(octyl)methylamine $$ N-Methyldioctylamine $$ N,N-Dioctylmethylamine $$

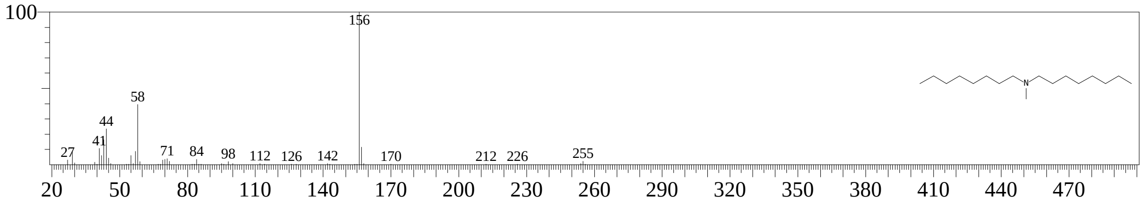

<< Target >>
Line#:16   R.Time:18.808(Scan#:1898) MassPeaks:257   BasePeak:170.05(2379)
RawMode:Averaged 18.800-18.817(1897-1899)   BG Mode:Calc. from Peak

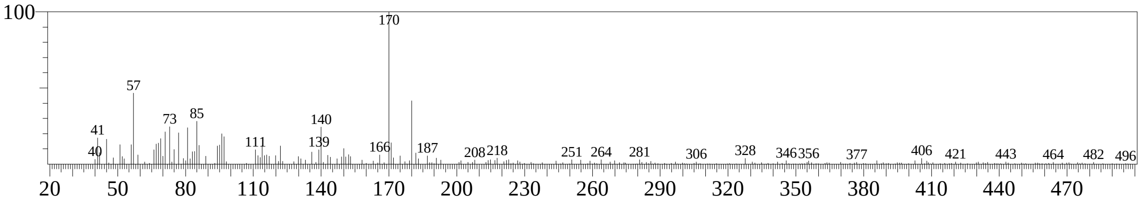

Hit#:1 Entry:101245 Library:NIST05.LIB
SI:58 Formula:C19H28N2O CAS:22311-11-1 MolWeight:300 RetIndex:2613
CompName:4-Piperidineethanol, .beta.-ethyl-1-(2-indol-3-ylethyl)- $$ 2-(1-[2-(1H-Indol-3-yl)ethyl]-4-piperidinyl)-1-butanol # $$

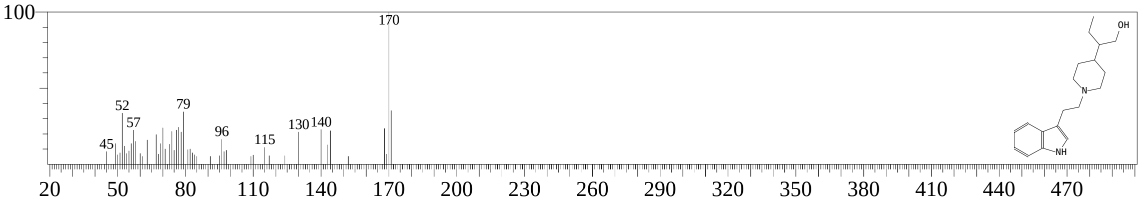

Hit#:2 Entry:66735 Library:NIST05.LIB
SI:57 Formula:C9H13N3O5 CAS:69-74-9 MolWeight:243 RetIndex:2385
CompName:Cytarabine Hydrochloride $$ 1-.beta.-D-Arabinofuranosylcytosine hydrochloride $$ Cytosine-.beta.-D-arabinofuranosi

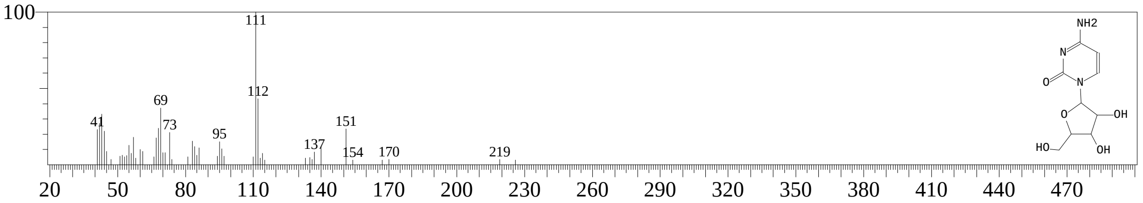

Hit#:3 Entry:98826 Library:NIST05.LIB
SI:56 Formula:C19H36O2 CAS:0-0-0 MolWeight:296 RetIndex:2120
CompName:Cyclopentanecarboxylic acid, tridecyl ester

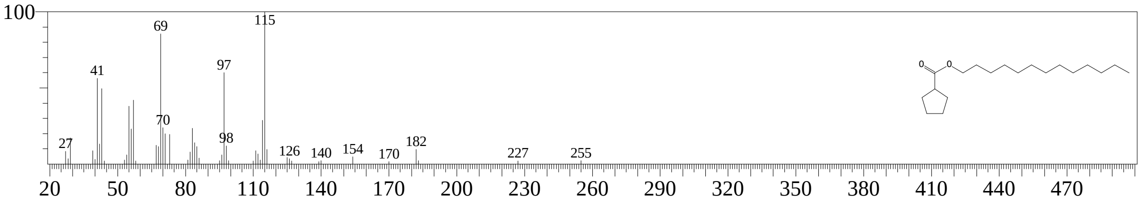

Hit#:4 Entry:73737 Library:NIST05.LIB
SI:56 Formula:C16H30O2 CAS:0-0-0 MolWeight:254 RetIndex:1822
CompName:Cyclopentanecarboxylic acid, decyl ester

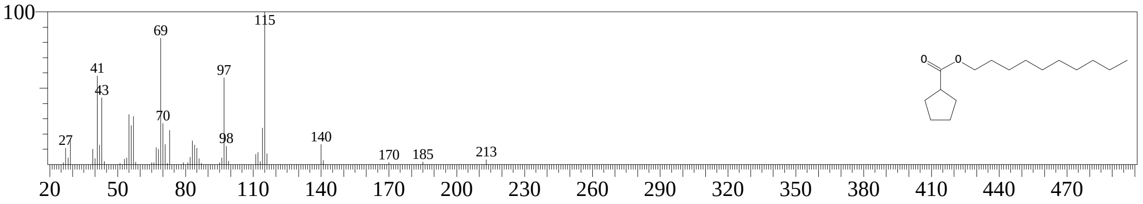

Hit#:5 Entry:56783 Library:NIST05.LIB
SI:55 Formula:C12H22N2O2 CAS:1436-27-7 MolWeight:226 RetIndex:1636
CompName:2,5-Piperazinedione, 3,6-bis(2-methylpropyl)- $$ 3,6-Diisobutyl-2,5-piperazinedione # $$

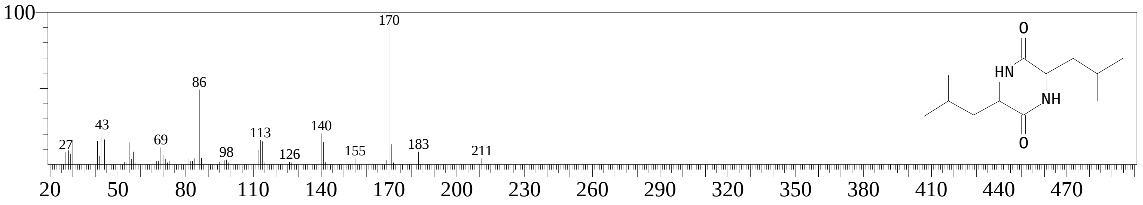

<< Target >>
Line#:17 R.Time:18.917(Scan#:1911) MassPeaks:233 BasePeak:156.00(1261)
RawMode:Averaged 18.908-18.925(1910-1912) BG Mode:Calc. from Peak

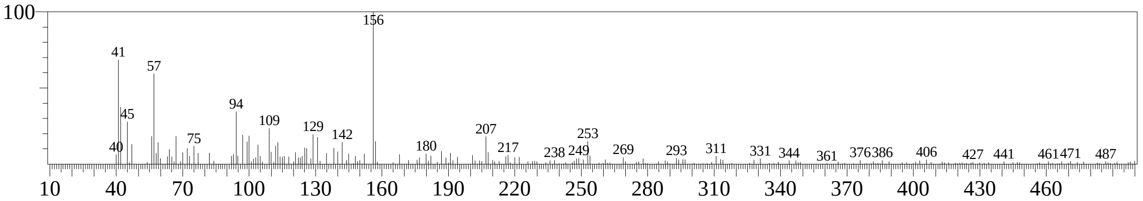

Hit#:1 Entry:128272 Library:NIST05.LIB
SI:53 Formula:C12H20INO3 CAS:0-0-0 MolWeight:353 RetIndex:1922
CompName:Carbamic acid, N-methyl-N-[6-iodo-9-oxabicyclo[3.3.1]nonan-2-yl]-, ethyl ester

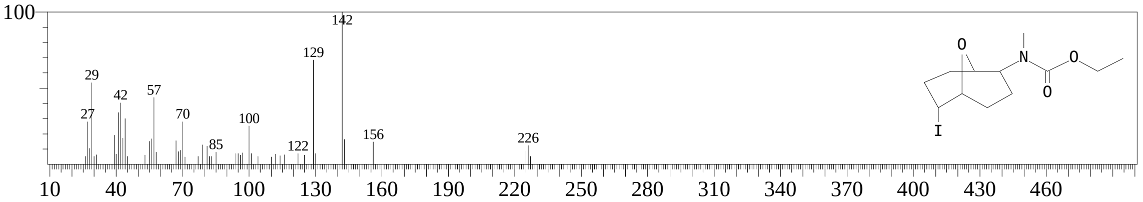

Hit#:2 Entry:40799 Library:NIST05.LIB
SI:53 Formula:C8H16N4O2 CAS:0-0-0 MolWeight:200 RetIndex:1958
CompName:1,2,4-Triazole-3,5-dipropanol, 4-amino-

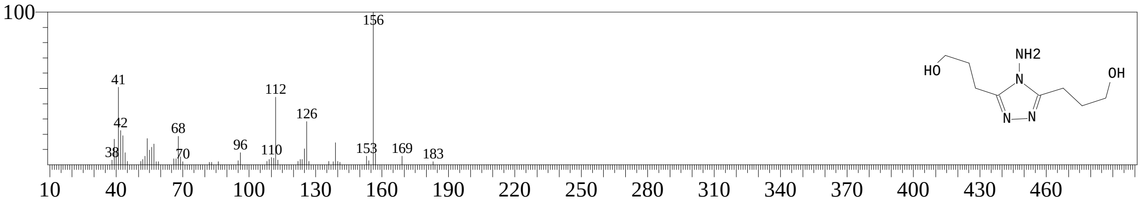

Hit#:3 Entry:66120 Library:NIST05.LIB
SI:53 Formula:C11H18N2O2S CAS:15182-54-4 MolWeight:242 RetIndex:2115
CompName:3-Morpholino-thioacrylomorpholide $$ 4-[(1E)-3-(4-Morpholinyl)-3-thioxo-1-propenyl]morpholine # $$

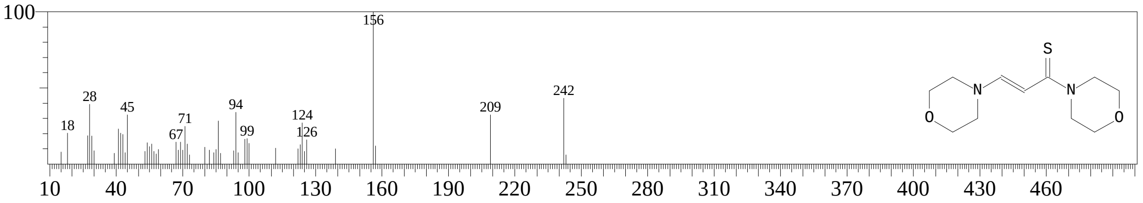

Hit#:4 Entry:17631 Library:NIST05.LIB
SI:53 Formula:C7H12N2S CAS:6601-20-3 MolWeight:156 RetIndex:1480
CompName:Thiourea, N,N'-di-2-propenyl- $$ Bisallylthiocarbamide $$ N,N'-Bis-allylthiourea $$ N,N'-Diallylthiourea $$ 1,3-Diall

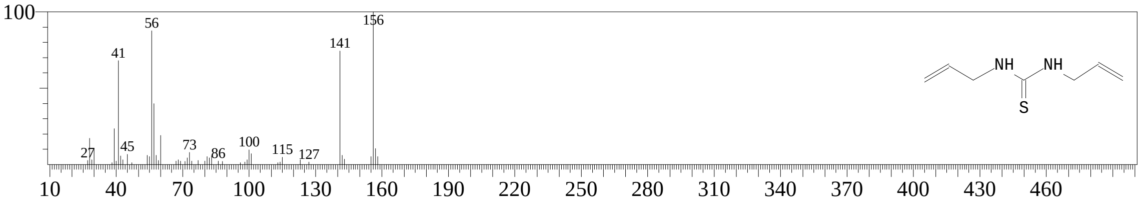

Hit#:5 Entry:72898 Library:NIST05.LIB
SI:52 Formula:C14H23NOS CAS:0-0-0 MolWeight:253 RetIndex:1890
CompName:2-Thiopheneacetamide, N,N-dibutyl-

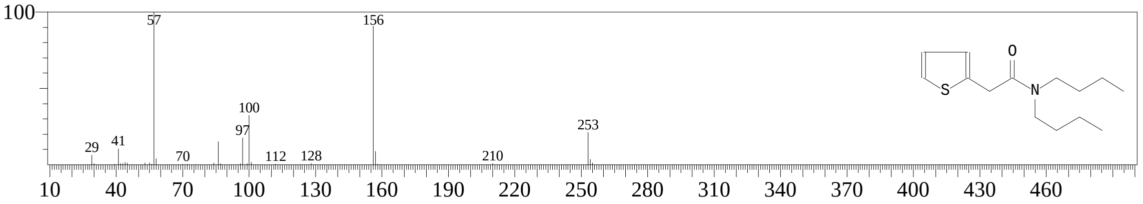

<< Target >>
Line#:18   R.Time:19.550(Scan#:1987) MassPeaks:243   BasePeak:144.00(17054)
RawMode:Averaged 19.542-19.558(1986-1988)   BG Mode:Calc. from Peak

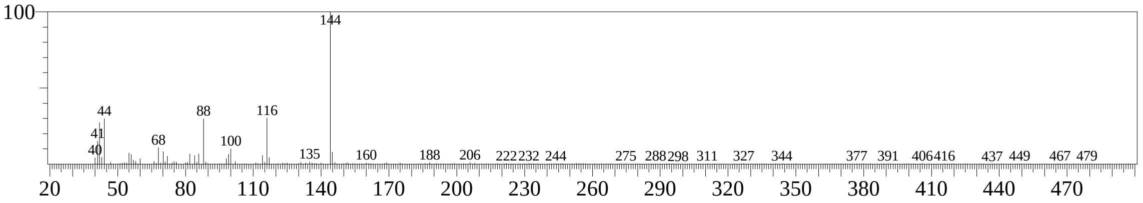

Hit#:1 Entry:109550 Library:NIST05.LIB
SI:79 Formula:C17H33NO4 CAS:0-0-0 MolWeight:315 RetIndex:2180
CompName:l-Alanine, N-butoxycarbonyl-, nonyl ester

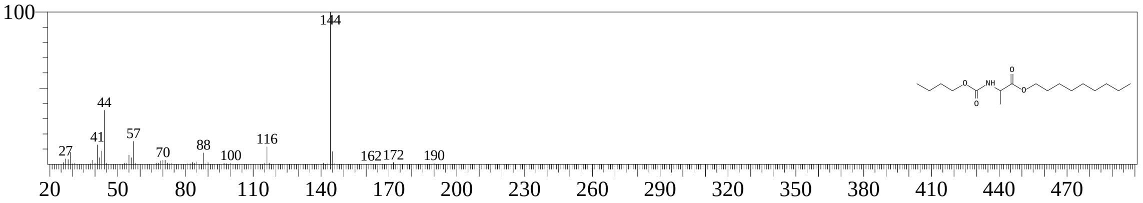

Hit#:2 Entry:67958 Library:NIST05.LIB
SI:79 Formula:C12H23NO4 CAS:0-0-0 MolWeight:245 RetIndex:1619
CompName:l-Alanine, N-butoxycarbonyl-, isobutyl ester

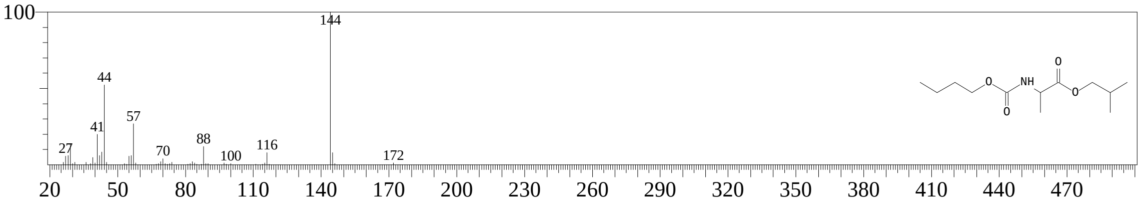

Hit#:3 Entry:93408 Library:NIST05.LIB
SI:79 Formula:C15H29NO4 CAS:0-0-0 MolWeight:287 RetIndex:1981
CompName:l-Alanine, N-butoxycarbonyl-, heptyl ester

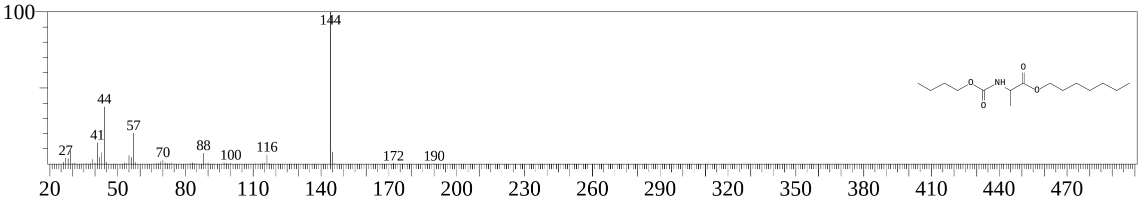

Hit#:4 Entry:76475 Library:NIST05.LIB
SI:78 Formula:C13H25NO4 CAS:0-0-0 MolWeight:259 RetIndex:1782
CompName:l-Alanine, N-butoxycarbonyl-, pentyl ester

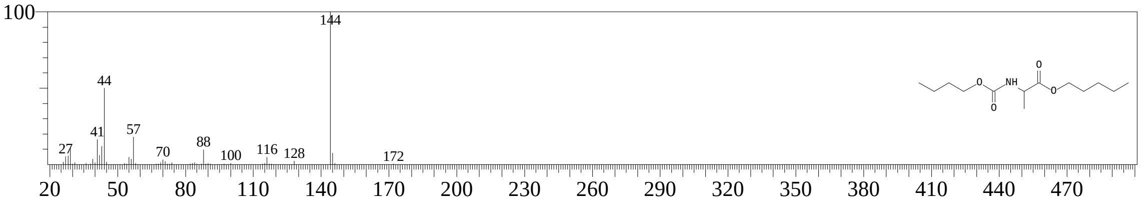

Hit#:5 Entry:101588 Library:NIST05.LIB
SI:78 Formula:C16H31NO4 CAS:0-0-0 MolWeight:301 RetIndex:2081
CompName:l-Alanine, N-butoxycarbonyl-, octyl ester

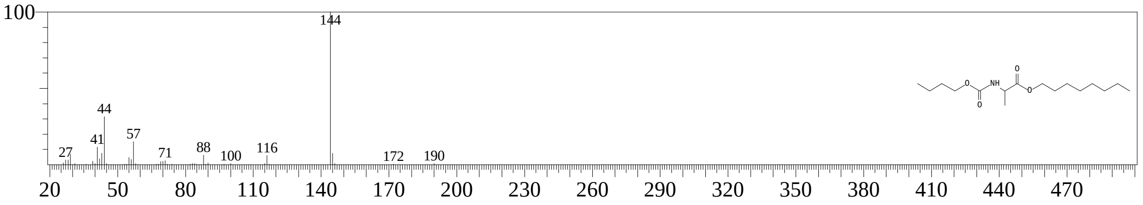

<< Target >>
Line#:19   R.Time:22.200(Scan#:2305) MassPeaks:244   BasePeak:74.00(7098)
RawMode:Averaged 22.192-22.208(2304-2306)   BG Mode:Calc. from Peak

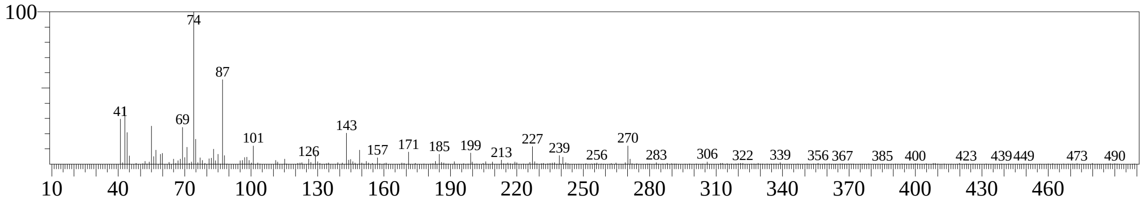

Hit#:1 Entry:83491 Library:NIST05.LIB
SI:90 Formula:C17H34O2 CAS:112-39-0 MolWeight:270 RetIndex:1878
CompName:Hexadecanoic acid, methyl ester $$ Palmitic acid, methyl ester $$ n-Hexadecanoic acid methyl ester $$ Metholene 221

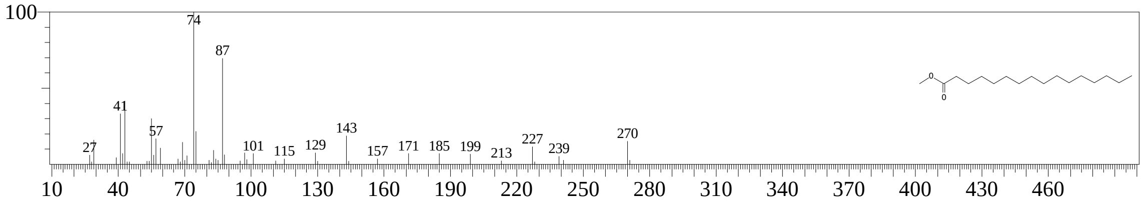

Hit#:2 Entry:22220 Library:NIST05s.LIB
SI:88 Formula:C17H34O2 CAS:112-39-0 MolWeight:270 RetIndex:1878
CompName:Hexadecanoic acid, methyl ester $$ Palmitic acid, methyl ester $$ n-Hexadecanoic acid methyl ester $$ Metholene 221

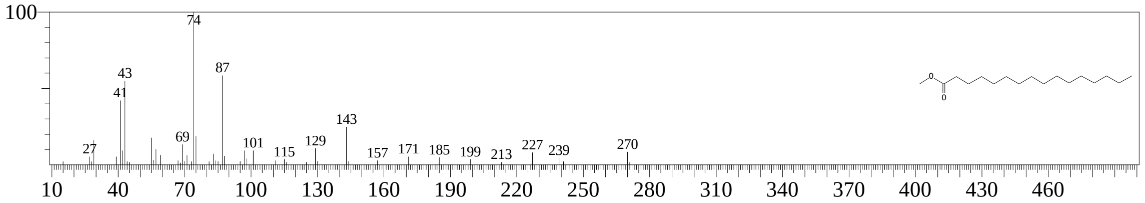

Hit#:3 Entry:22219 Library:NIST05s.LIB
SI:87 Formula:C17H34O2 CAS:112-39-0 MolWeight:270 RetIndex:1878
CompName:Hexadecanoic acid, methyl ester $$ Palmitic acid, methyl ester $$ n-Hexadecanoic acid methyl ester $$ Metholene 221

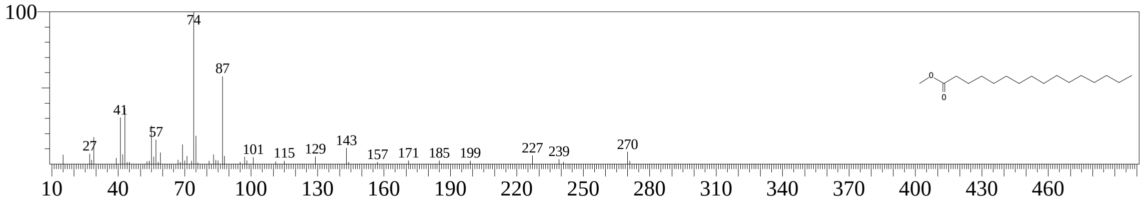

Hit#:4 Entry:22222 Library:NIST05s.LIB
SI:86 Formula:C17H34O2 CAS:5129-60-2 MolWeight:270 RetIndex:1814
CompName:Pentadecanoic acid, 14-methyl-, methyl ester $$ Methyl 14-methylpentadecanoate # $$

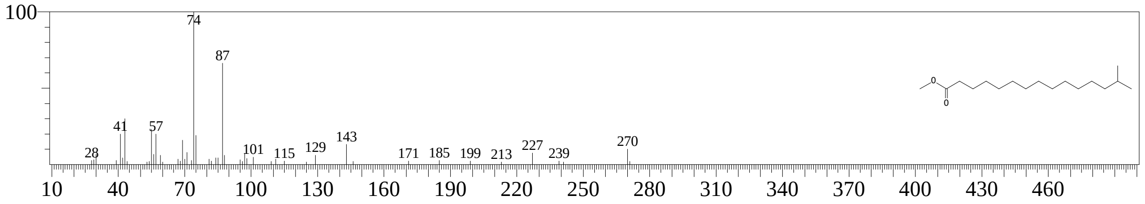

Hit#:5 Entry:22221 Library:NIST05s.LIB
SI:86 Formula:C17H34O2 CAS:112-39-0 MolWeight:270 RetIndex:1878
CompName:Hexadecanoic acid, methyl ester $$ Palmitic acid, methyl ester $$ n-Hexadecanoic acid methyl ester $$ Metholene 221

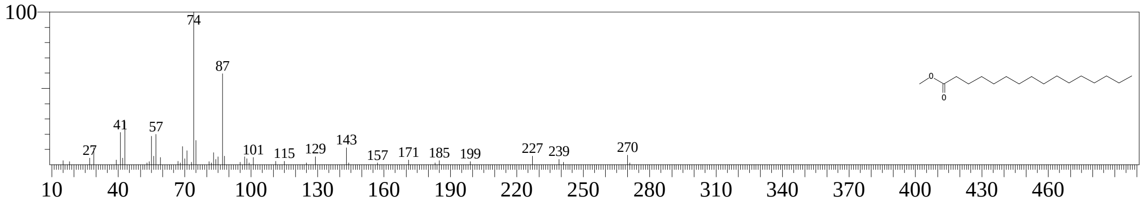

<< Target >>
Line#:20   R.Time:22.567(Scan#:2349) MassPeaks:282   BasePeak:43.00(9684)
RawMode:Averaged 22.558-22.575(2348-2350)   BG Mode:Calc. from Peak

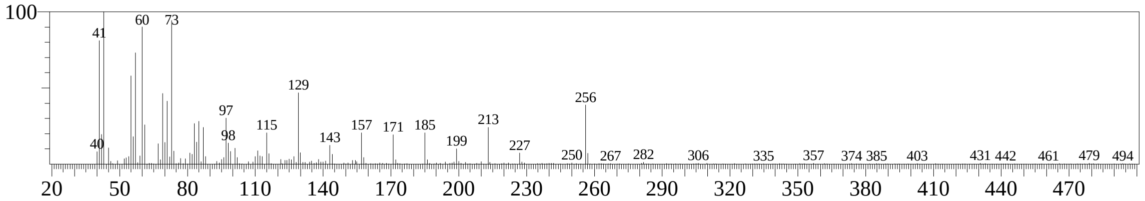

Hit#:1 Entry:21331 Library:NIST05s.LIB
SI:93 Formula:C16H32O2 CAS:57-10-3 MolWeight:256 RetIndex:1968
CompName:n-Hexadecanoic acid $$ Hexadecanoic acid $$ n-Hexadecoic acid $$ Palmitic acid $$ Pentadecanecarboxylic acid $$ 1

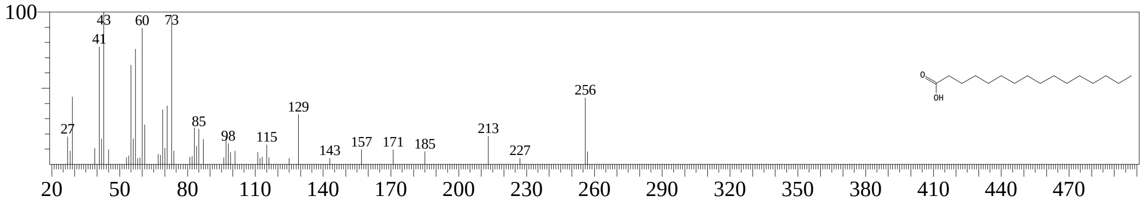

Hit#:2 Entry:20371 Library:NIST05s.LIB
SI:90 Formula:C15H30O2 CAS:1002-84-2 MolWeight:242 RetIndex:1869
CompName:Pentadecanoic acid $$ Pentadecylic acid $$ n-Pentadecanoic acid $$ n-Pentadecylic acid $$

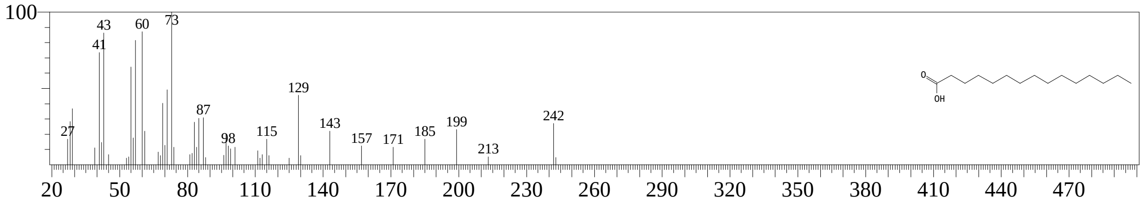

Hit#:3 Entry:161860 Library:NIST05.LIB
SI:88 Formula:C38H68O8 CAS:28474-90-0 MolWeight:652 RetIndex:4765
CompName:l-(+)-Ascorbic acid 2,6-dihexadecanoate

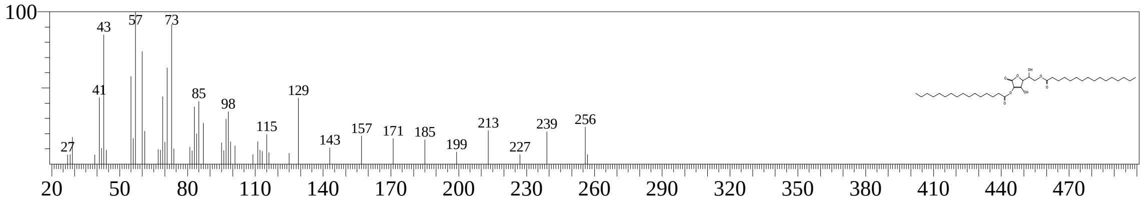

Hit#:4 Entry:22979 Library:NIST05s.LIB
SI:88 Formula:C18H36O2 CAS:57-11-4 MolWeight:284 RetIndex:2167
CompName:Octadecanoic acid $$ Stearic acid $$ n-Octadecanoic acid $$ Humko Industrene R $$ Hydrofol Acid 150 $$ Hystrene

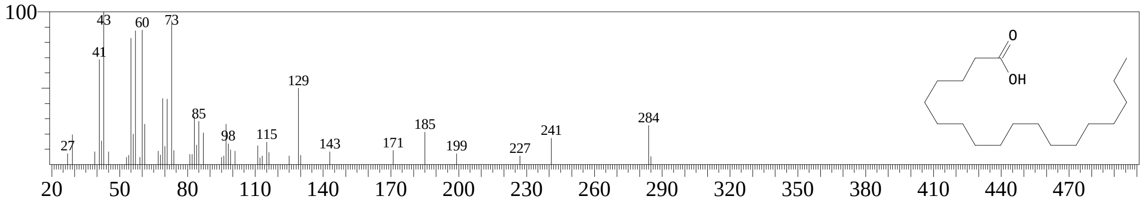

Hit#:5 Entry:19250 Library:NIST05s.LIB
SI:88 Formula:C14H28O2 CAS:544-63-8 MolWeight:228 RetIndex:1769
CompName:Tetradecanoic acid $$ Myristic acid $$ n-Tetradecanoic acid $$ n-Tetradecoic acid $$ Neo-Fat 14 $$ Univol U 316S $

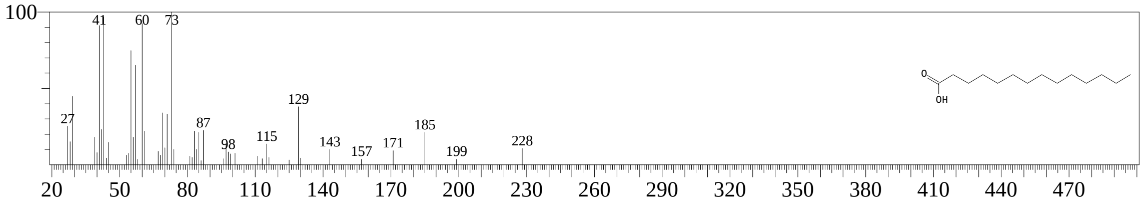

<< Target >>
Line#:21 R.Time:24.383(Scan#:2567) MassPeaks:298 BasePeak:55.00(4288)
RawMode:Averaged 24.375-24.392(2566-2568) BG Mode:Calc. from Peak

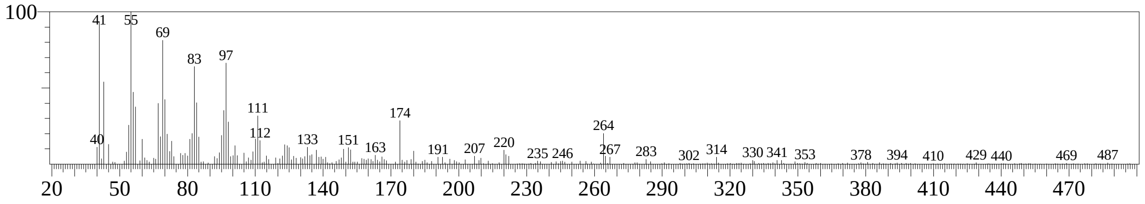

Hit#:1 Entry:90575 Library:NIST05.LIB
SI:87 Formula:C18H34O2 CAS:0-0-0 MolWeight:282 RetIndex:2175
CompName:Octadec-9-enoic acid

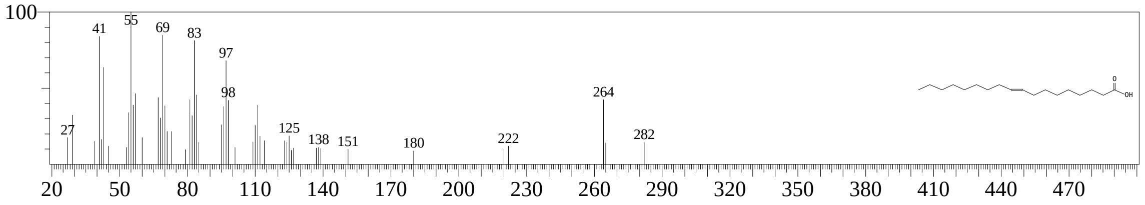

Hit#:2 Entry:90577 Library:NIST05.LIB
SI:86 Formula:C18H34O2 CAS:112-80-1 MolWeight:282 RetIndex:2175
CompName:Oleic Acid $$ 9-Octadecenoic acid (Z)- $$ .delta.(Sup9)-cis-Oleic acid $$ cis-.delta.(Sup9)-Octadecenoic acid $$ cis-C

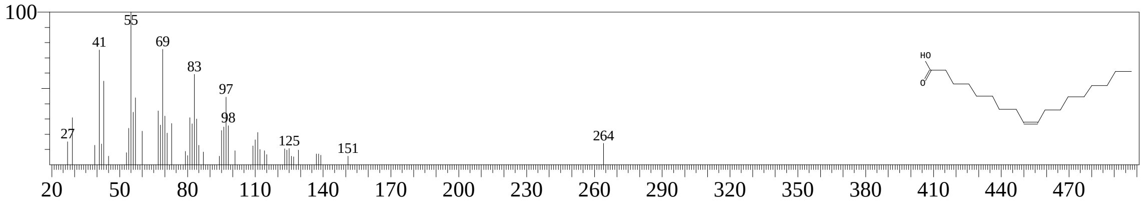

Hit#:3 Entry:22872 Library:NIST05s.LIB
SI:86 Formula:C18H34O2 CAS:112-79-8 MolWeight:282 RetIndex:2175
CompName:9-Octadecenoic acid, (E)- $$ trans-.delta.(sup 9)-Octadecenoic acid $$ trans-.delta.9-Octadecenoic acid $$ trans-Octad

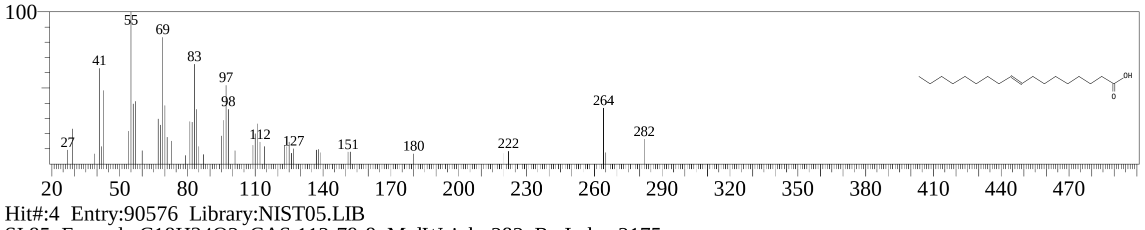

Hit#:4 Entry:90576 Library:NIST05.LIB
SI:85 Formula:C18H34O2 CAS:112-79-8 MolWeight:282 RetIndex:2175
CompName:9-Octadecenoic acid, (E)- $$ trans-.delta.(sup 9)-Octadecenoic acid $$ trans-.delta.9-Octadecenoic acid $$ trans-Octad

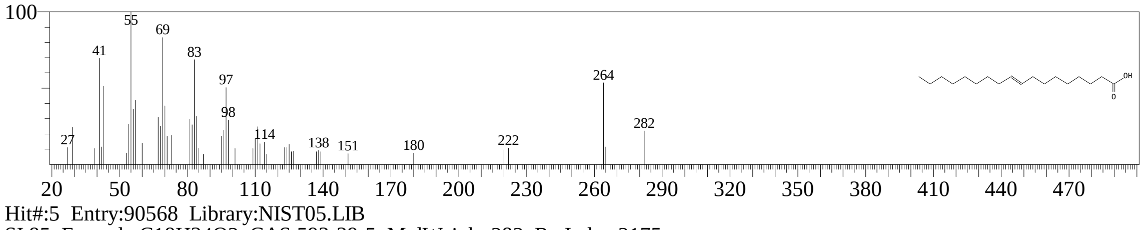

Hit#:5 Entry:90568 Library:NIST05.LIB
SI:85 Formula:C18H34O2 CAS:593-39-5 MolWeight:282 RetIndex:2175
CompName:6-Octadecenoic acid, (Z)- $$ (6Z)-6-Octadecenoic acid # $$

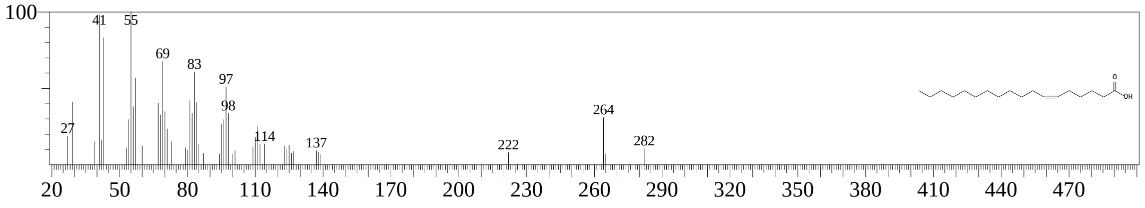

<< Target >>
Line#:22 R.Time:26.733(Scan#:2849) MassPeaks:252 BasePeak:251.95(5199)
RawMode:Averaged 26.725-26.742(2848-2850) BG Mode:Calc. from Peak

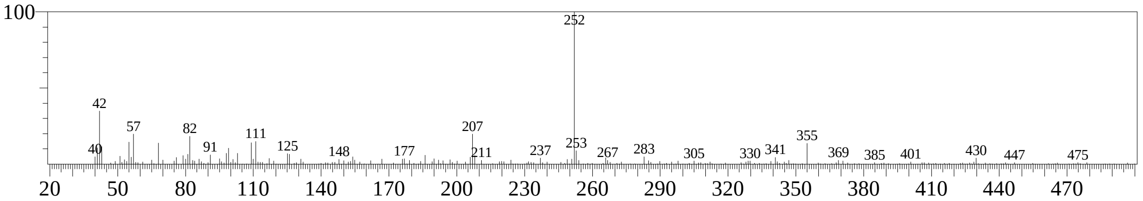

Hit#:1 Entry:139076 Library:NIST05.LIB
SI:57 Formula:C23H44N2O2 CAS:0-0-0 MolWeight:380 RetIndex:2606
CompName:Cyclohexane-1,2-dicarboxylic acid, butyl-propyl-amide) diisobutylamide

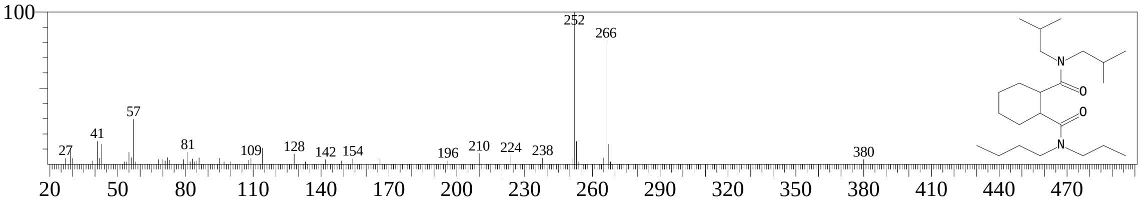

Hit#:2 Entry:71870 Library:NIST05.LIB
SI:55 Formula:C5H5IN2O2 CAS:6647-98-9 MolWeight:252 RetIndex:1565
CompName:Pyrazole-3-carboxylic acid, 4-iodo-1-methyl- $$ 4-Iodo-1-methyl-1H-pyrazole-3-carboxylic acid # $$

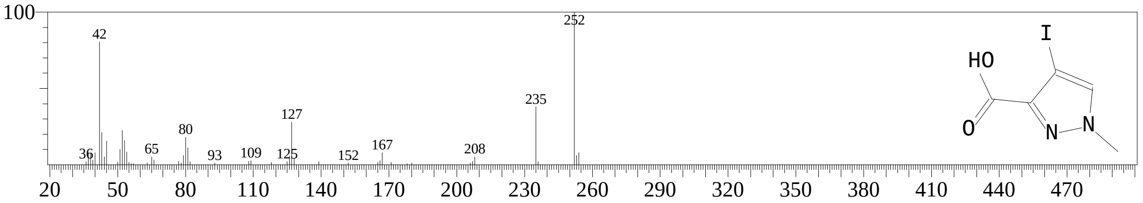

Hit#:3 Entry:89908 Library:NIST05.LIB
SI:54 Formula:C19H39N CAS:0-0-0 MolWeight:281 RetIndex:2160
CompName:2-Ethyl-5-tridecylpyrrolidine

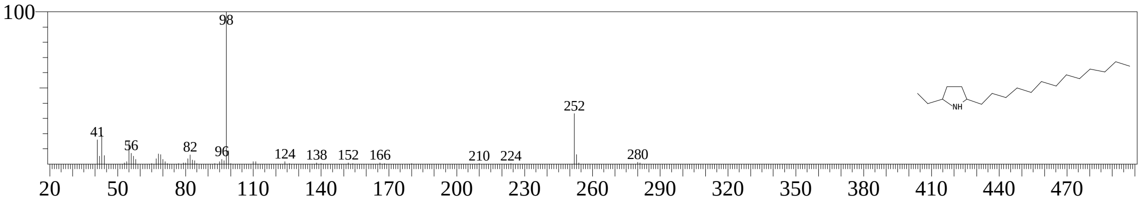

Hit#:4 Entry:72354 Library:NIST05.LIB
SI:54 Formula:C15H12N2O2 CAS:0-0-0 MolWeight:252 RetIndex:2466
CompName:2,9-Dimethyl-5-carboxy-1,10-phenanthroline

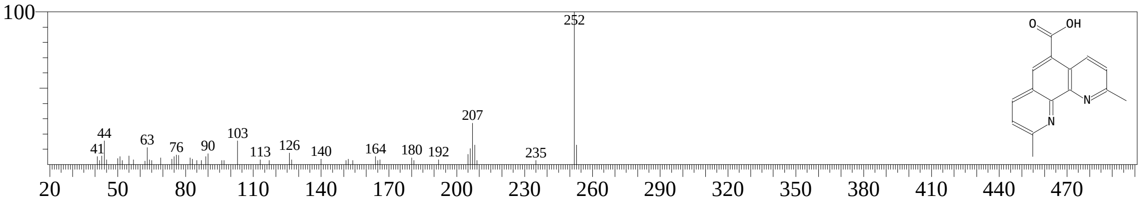

Hit#:5 Entry:97931 Library:NIST05.LIB
SI:53 Formula:C15H21NO3S CAS:318466-23-8 MolWeight:295 RetIndex:2483
CompName:Quinolin-4-ol, perhydro-1-phenylsulfonyl- $$ 1-(Phenylsulfonyl)decahydro-4-quinolinol # $$

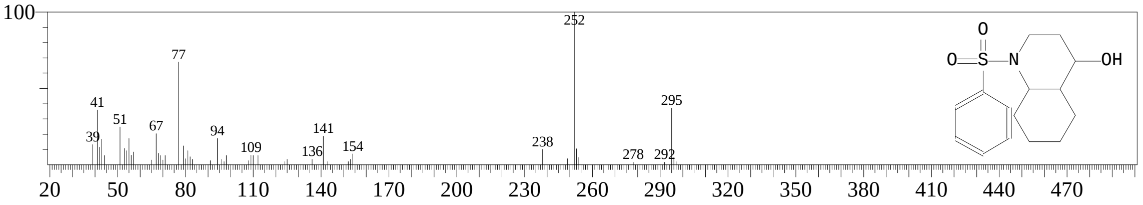

Supplement: Supplemental Information 3 [file peerj-07-6442-s003.pdf]
