# Supplementary material for: Comparative study on the composition of four different varieties of garlic
Source: PeerJ. 2019 Feb 21;7:e6442. doi: 10.7717/peerj.6442 (PMC6387757; doi:10.7717/peerj.6442)

Sample Information

Analyzed by	: Admin
Analyzed	: 2018-5-7 21:21:34
Sample Type	: Unknown
Level #	: 1
Sample Name	: 2018-5-7-3-2
Sample ID	:
IS Amount	: [1]=1.000
Sample Amount	: 1.000
Dilution Factor	: 1.000
Vial #	: 1
Injection Volume	: 1.000
Data File	: E:\陈存\GC-MS硫化物\2018-5-7-3-2.qgd
Org Data File	: E:\刘春宏\2018-5-7-3-2.qgd
Method File	: E:\刘春宏\2018-5-7-1.qgm
Org Method File	: E:\刘春宏\2018-5-7-1.qgm
Report File	:
Tuning File	: C:\GCMSsolution\System\Tune1\2017-3-21.qgt
Modified by	: Admin
Modified	: 2018-5-11 11:52:41

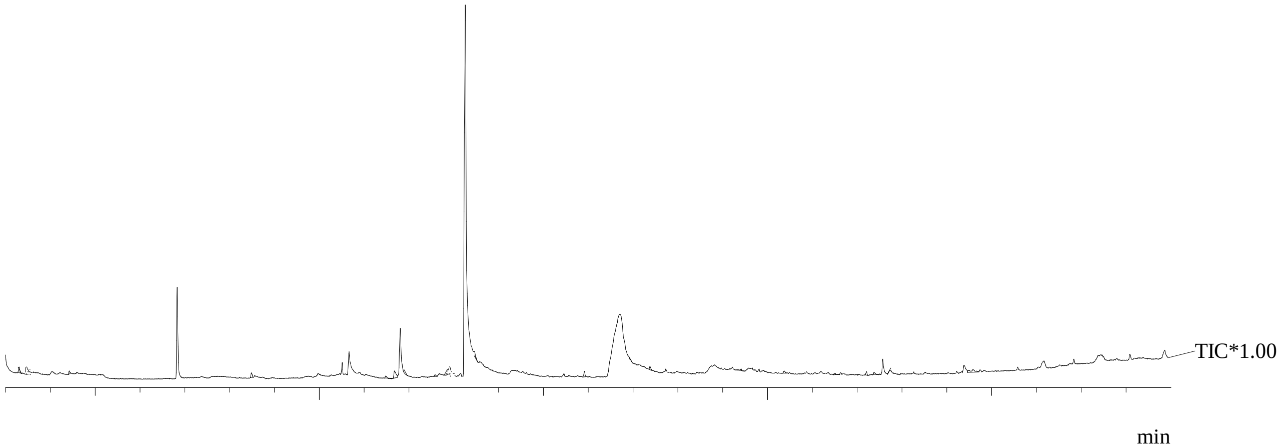

Peak Report TIC

Peak#	R.Time	I.Time	F.Time	Area	Area%	Height	Ieight%	A/H	Mark	Name
1	3.304	3.275	3.358	140258	0.35	78446	0.86	1.78		
2	4.424	4.400	4.458	73966	0.19	39609	0.43	1.86		
3	6.831	6.792	6.917	2379939	5.96	1248290	13.66	1.90		
4	8.491	8.458	8.525	112932	0.28	62973	0.69	1.79		
5	10.665	10.625	10.833	1172864	2.94	315173	3.45	3.72		
6	11.483	11.458	11.650	36737	0.09	29461	0.32	1.24		
7	11.683	11.650	11.750	329004	0.82	97512	1.07	3.37		
8	11.808	11.750	11.967	1661654	4.16	575760	6.30	2.89	V	
9	12.583	12.550	12.633	61916	0.16	20360	0.22	3.04		
10	12.974	12.933	13.067	591854	1.48	170838	1.87	3.46		
11	13.261	13.208	13.542	18799111	47.10	5064815	55.44	3.71	S	
12	15.918	15.883	15.958	146356	0.37	74754	0.82	1.95		
13	16.702	16.417	16.992	12375424	31.00	761434	8.33	16.25		
14	17.381	17.358	17.425	84058	0.21	44198	0.48	1.90		
15	19.413	19.383	19.450	42565	0.11	24279	0.27	1.75		
16	20.374	20.342	20.425	75538	0.19	32323	0.35	2.33		
17	21.492	21.475	21.608	34978	0.09	15230	0.17	2.29		
18	22.209	22.175	22.242	77123	0.19	46010	0.50	1.67		
19	22.375	22.350	22.542	87108	0.22	29622	0.32	2.94		
20	22.574	22.542	22.675	562465	1.41	214158	2.34	2.62	V	
21	22.717	22.675	22.950	382289	0.96	50296	0.55	7.60	V	
22	24.390	24.350	24.467	392209	0.98	101898	1.12	3.84	V	
23	24.583	24.467	24.725	294117	0.74	38266	0.42	7.68	V	
				39914465	100.00	9135705	100.00			

Library

<< Target >>
Line#:1   R.Time:3.300(Scan#:37) MassPeaks:230   BasePeak:43.00(50443)
RawMode:Averaged 3.292-3.308(36-38)   BG Mode:Calc. from Peak

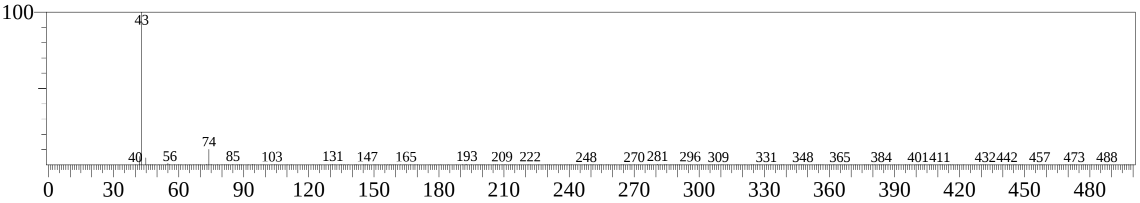

Hit#:1 Entry:415 Library:NIST05s.LIB
SI:95 Formula:C3H6O2 CAS:116-9-6 MolWeight:74 RetIndex:698
CompName:2-Propanone, 1-hydroxy- $$ Acetol $$ CH3C(O)CH2OH $$ Hydroxyacetone $$ Acetone alcohol $$ Acetylcarbinol $$

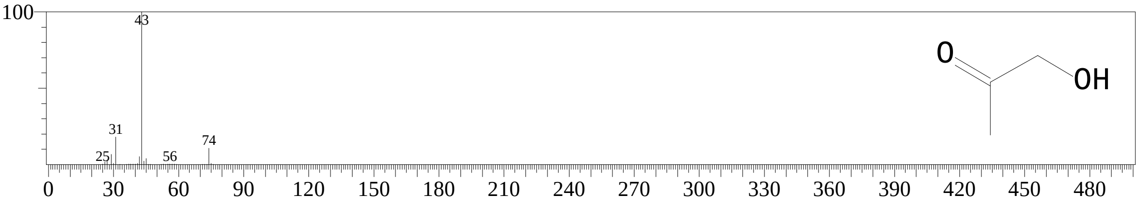

Hit#:2 Entry:368 Library:NIST05.LIB
SI:95 Formula:C3H6O2 CAS:116-9-6 MolWeight:74 RetIndex:698
CompName:2-Propanone, 1-hydroxy- $$ Acetol $$ CH3C(O)CH2OH $$ Hydroxyacetone $$ Acetone alcohol $$ Acetylcarbinol $$

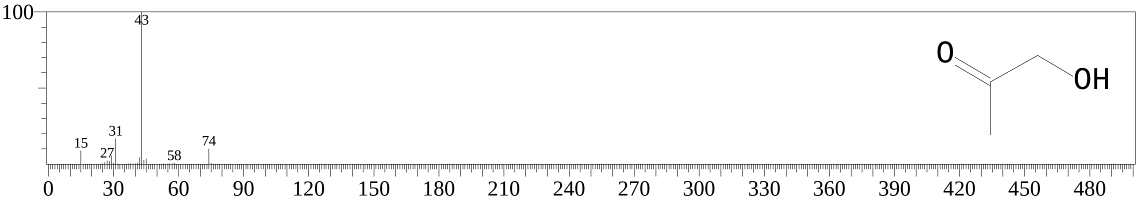

Hit#:3 Entry:414 Library:NIST05s.LIB
SI:93 Formula:C3H6O2 CAS:116-9-6 MolWeight:74 RetIndex:698
CompName:2-Propanone, 1-hydroxy- $$ Acetol $$ CH3C(O)CH2OH $$ Hydroxyacetone $$ Acetone alcohol $$ Acetylcarbinol $$

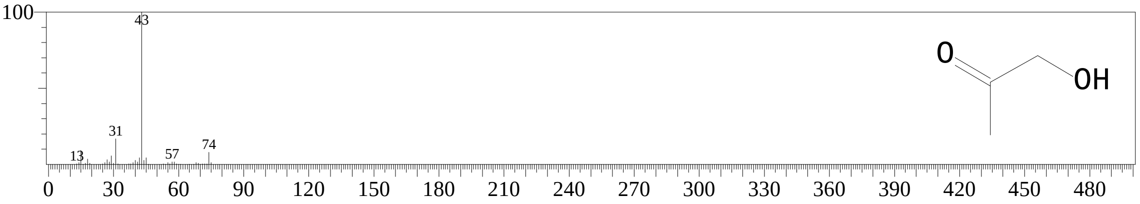

Hit#:4 Entry:1075 Library:NIST05s.LIB
SI:91 Formula:C3H7NO2 CAS:79-46-9 MolWeight:89 RetIndex:637
CompName:Propane, 2-nitro- $$ Dimethylnitromethane $$ Isonitropropane $$ 2-Nitropropane $$ i-C3H7NO2 $$ sec-Nitropropane

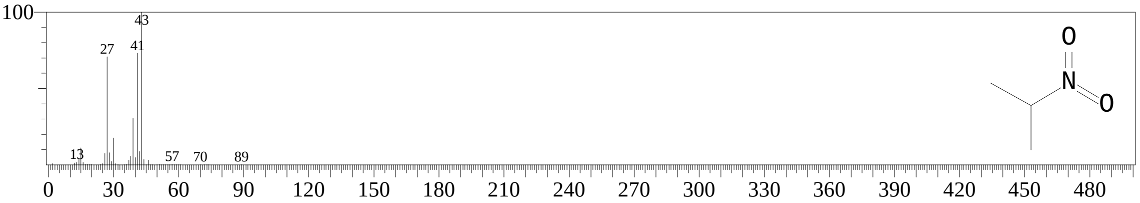

Hit#:5 Entry:1953 Library:NIST05s.LIB
SI:90 Formula:C4H6O3 CAS:108-24-7 MolWeight:102 RetIndex:722
CompName:Acetic anhydride $$ Acetic acid, anhydride $$ Acetic oxide $$ Acetyl anhydride $$ Acetyl ether $$ Acetyl oxide $$ Et

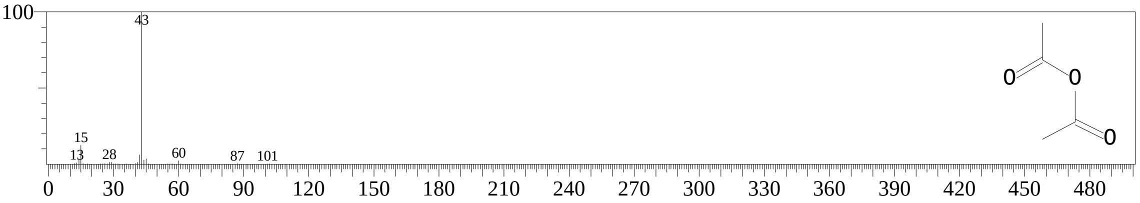

<< Target >>
Line#:2 R.Time:4.425(Scan#:172) MassPeaks:245 BasePeak:45.00(19852)
RawMode:Averaged 4.417-4.433(171-173) BG Mode:Calc. from Peak

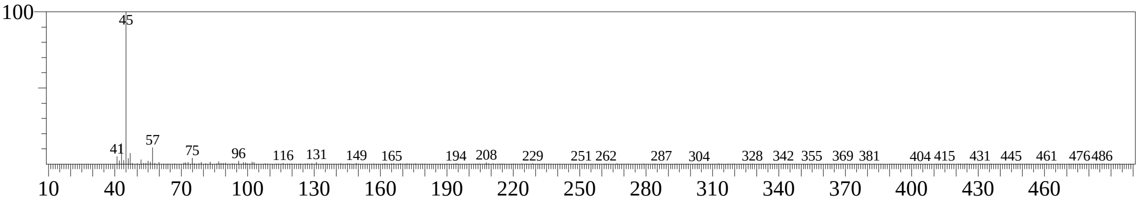

Hit#:1 Entry:1153 Library:NIST05s.LIB
SI:90 Formula:C4H10O2 CAS:513-85-9 MolWeight:90 RetIndex:743
CompName:2,3-Butanediol $$ Butane-2,3-diol $$ Dimethylethylene glycol $$ 2,3-Butylene glycol $$ 2,3-Dihydroxybutane $$ D-2,

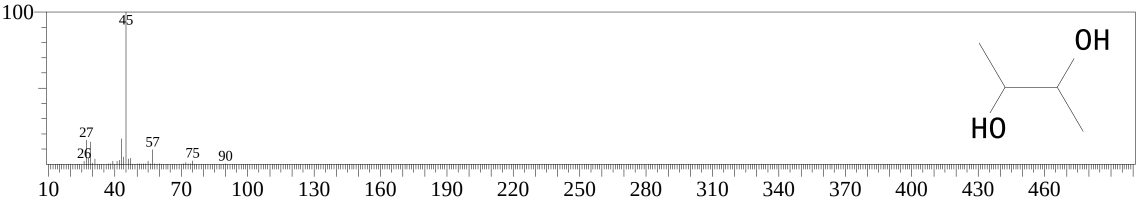

Hit#:2 Entry:1126 Library:NIST05.LIB
SI:90 Formula:C4H10O2 CAS:24347-58-8 MolWeight:90 RetIndex:743
CompName:2,3-Butanediol, [R-(R*,R*)]- $$ 2,3-Butanediol # $$

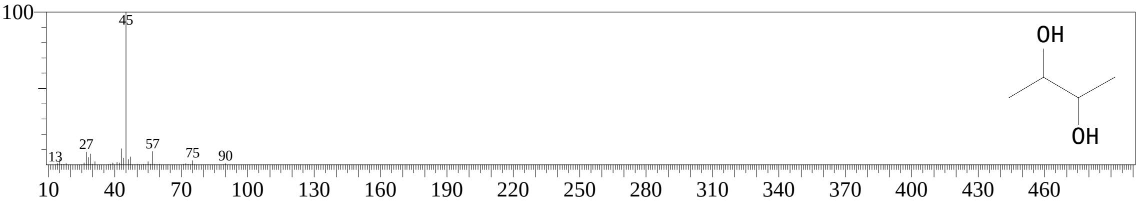

Hit#:3 Entry:1125 Library:NIST05.LIB
SI:89 Formula:C4H10O2 CAS:513-85-9 MolWeight:90 RetIndex:743
CompName:2,3-Butanediol $$ Butane-2,3-diol $$ Dimethylethylene glycol $$ 2,3-Butylene glycol $$ 2,3-Dihydroxybutane $$ D-2,

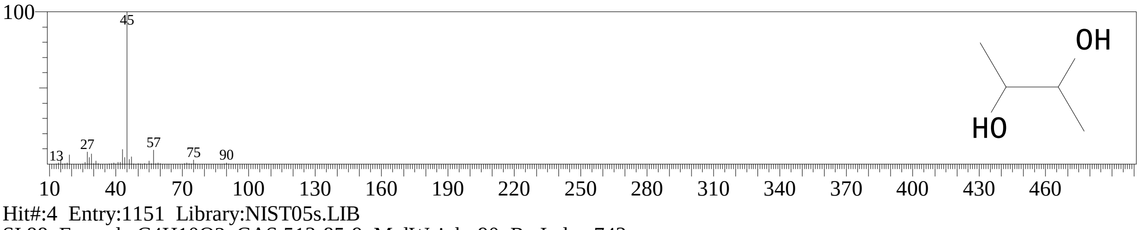

Hit#:4 Entry:1151 Library:NIST05s.LIB
SI:88 Formula:C4H10O2 CAS:513-85-9 MolWeight:90 RetIndex:743
CompName:2,3-Butanediol $$ Butane-2,3-diol $$ Dimethylethylene glycol $$ 2,3-Butylene glycol $$ 2,3-Dihydroxybutane $$ D-2,

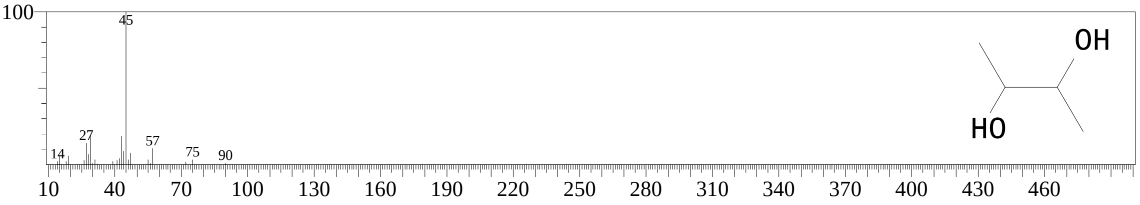

Hit#:5 Entry:1123 Library:NIST05.LIB
SI:88 Formula:C4H10O2 CAS:19132-6-0 MolWeight:90 RetIndex:743
CompName:2,3-Butanediol, [S-(R*,R*)]- $$ (2S,3S)-(+)-2,3-Butanediol $$ 2,3-Butanediol # $$

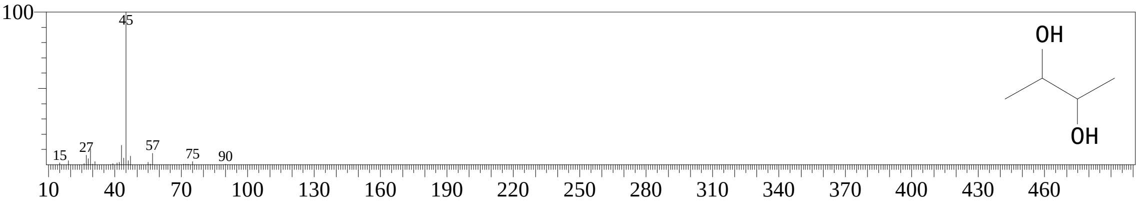

<< Target >>
Line#:3   R.Time:6.833(Scan#:461) MassPeaks:270   BasePeak:132.95(352509)
RawMode:Averaged 6.825-6.842(460-462)   BG Mode:Calc. from Peak

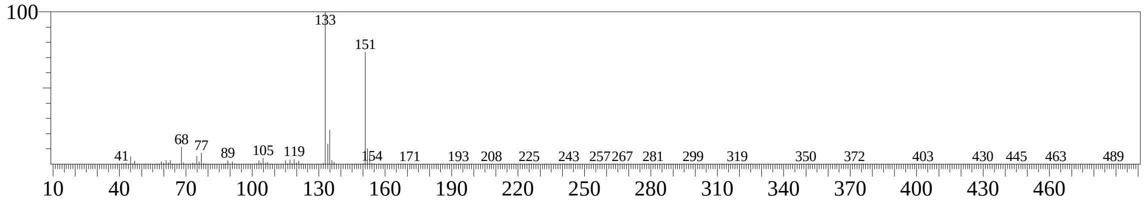

Hit#:1 Entry:15209 Library:NIST05.LIB
SI:85 Formula:C8H9NO2 CAS:0-0-0 MolWeight:151 RetIndex:1301
CompName:Oxime-, methoxy-phenyl-_

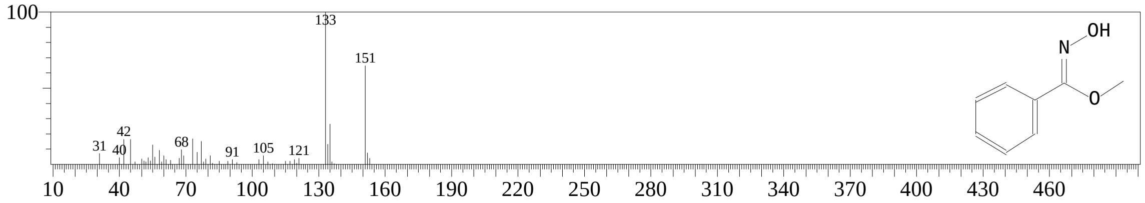

Hit#:2 Entry:15206 Library:NIST05.LIB
SI:80 Formula:C8H9NO2 CAS:2941-78-8 MolWeight:151 RetIndex:1575
CompName:2-Amino-5-methylbenzoic acid $$ 5-Methylanthranilic acid $$ Benzoic acid, 2-amino-5-methyl- $$ m-Toluic acid, 6-a

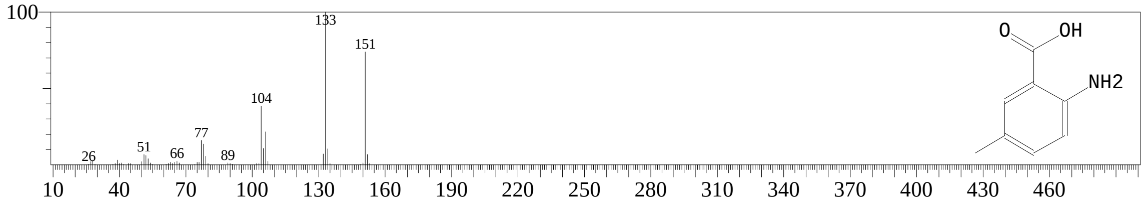

Hit#:3 Entry:51823 Library:NIST05.LIB
SI:79 Formula:C14H18O2 CAS:0-0-0 MolWeight:218 RetIndex:1714
CompName:4-Ethylbenzoic acid, cyclopentyl ester

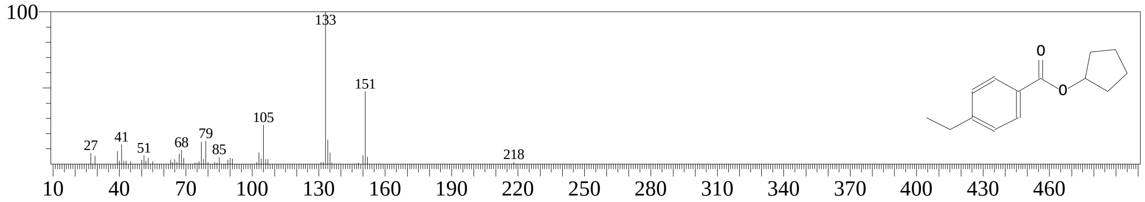

Hit#:4 Entry:61585 Library:NIST05.LIB
SI:78 Formula:C15H22O2 CAS:0-0-0 MolWeight:234 RetIndex:1770
CompName:4-Ethylbenzoic acid, hexyl ester

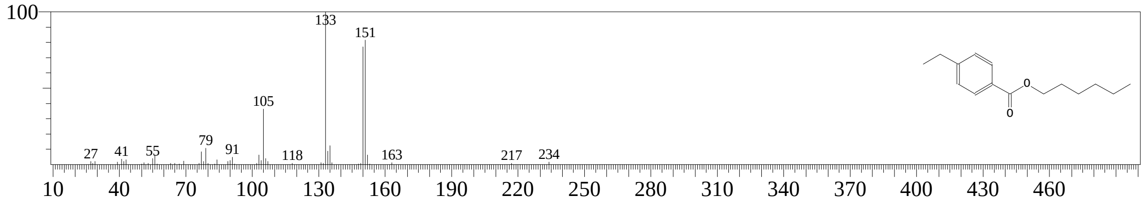

Hit#:5 Entry:15207 Library:NIST05.LIB
SI:77 Formula:C8H9NO2 CAS:2305-36-4 MolWeight:151 RetIndex:1575
CompName:Benzoic acid, 2-amino-4-methyl- $$ p-Toluic acid, 2-amino- $$ 2-Amino-4-methylbenzoic acid # $$

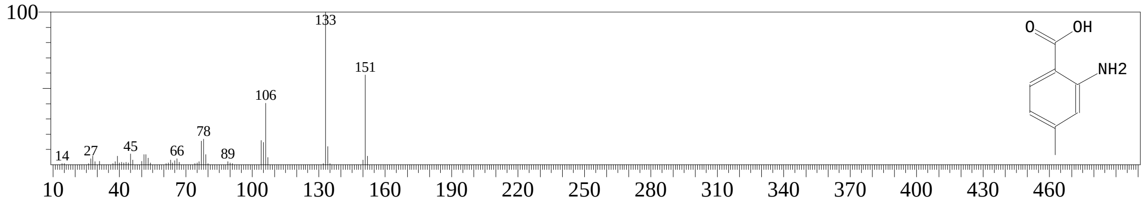

<< Target >>
Line#:4 R.Time:8.492(Scan#:660) MassPeaks:227 BasePeak:101.00(12321)
RawMode:Averaged 8.483-8.500(659-661) BG Mode:Calc. from Peak

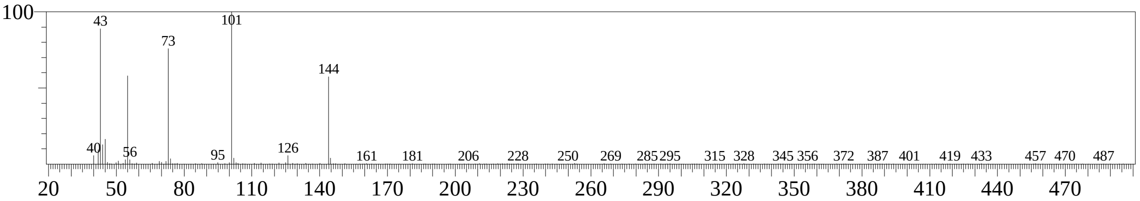

Hit#:1 Entry:12371 Library:NIST05.LIB
SI:90 Formula:C6H8O4 CAS:10230-62-3 MolWeight:144 RetIndex:1173
CompName:2,4-Dihydroxy-2,5-dimethyl-3(2H)-furan-3-one $$ 2,4-Dihydroxy-2,5-dimethyl-3(2H)-furanone # $$

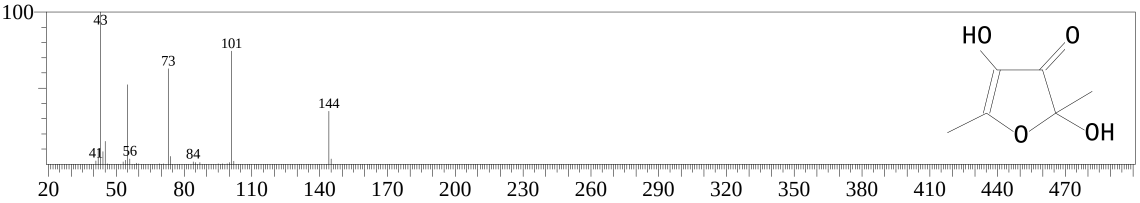

Hit#:2 Entry:7403 Library:NIST05s.LIB
SI:81 Formula:C6H8O4 CAS:28564-83-2 MolWeight:144 RetIndex:1269
CompName:4H-Pyran-4-one, 2,3-dihydro-3,5-dihydroxy-6-methyl- $$ 3,5-Dihydroxy-6-methyl-2,3-dihydro-4H-pyran-4-one # $$

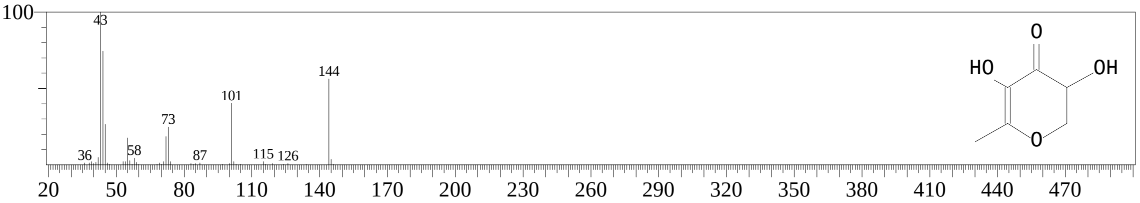

Hit#:3 Entry:12367 Library:NIST05.LIB
SI:78 Formula:C6H8O4 CAS:28564-83-2 MolWeight:144 RetIndex:1269
CompName:4H-Pyran-4-one, 2,3-dihydro-3,5-dihydroxy-6-methyl- $$ 3,5-Dihydroxy-6-methyl-2,3-dihydro-4H-pyran-4-one # $$

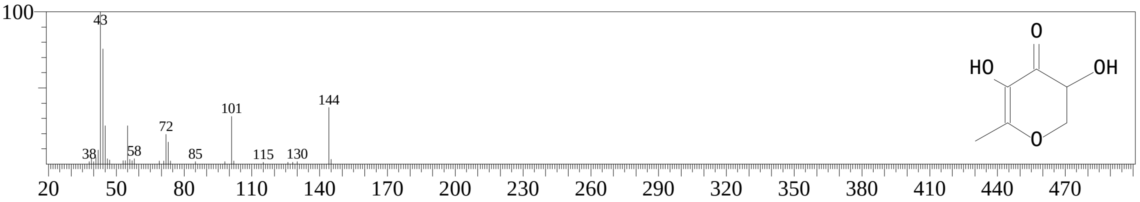

Hit#:4 Entry:13094 Library:NIST05.LIB
SI:76 Formula:C5H6O5 CAS:328-50-7 MolWeight:146 RetIndex:1367
CompName:2-Oxopentanedioic acid $$ 2-Ketoglutaric acid $$ 2-Oxoglutaric acid $$ .alpha.-Ketoglutaric acid $$ Pentanedioic acic

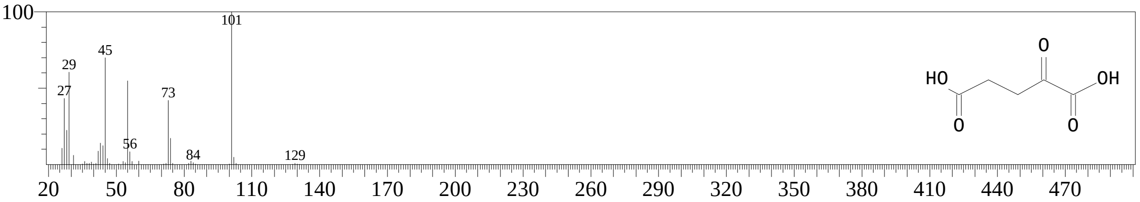

Hit#:5 Entry:4455 Library:NIST05.LIB
SI:72 Formula:C6H12O2 CAS:3299-32-9 MolWeight:116 RetIndex:761
CompName:1,3-Dioxolane, 2,4,5-trimethyl- $$ 2,4,5-Trimethyl-1,3-dioxolane # $$

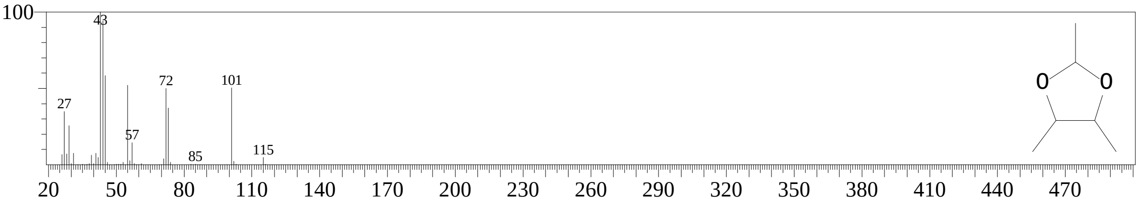

<< Target >>
Line#:5 R.Time:10.667(Scan#:921) MassPeaks:258 BasePeak:43.00(98359)
RawMode:Averaged 10.658-10.675(920-922) BG Mode:Calc. from Peak

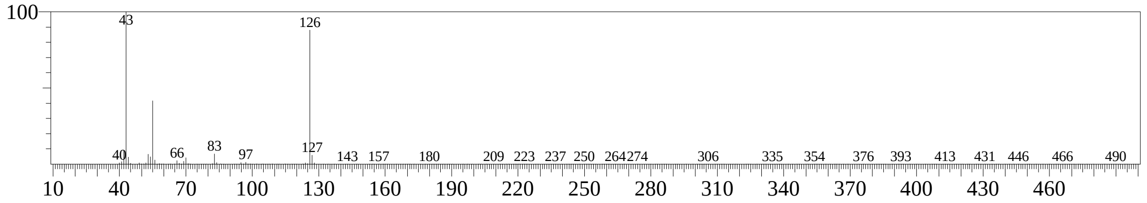

Hit#:1 Entry:4475 Library:NIST05s.LIB
SI:83 Formula:C3H6N6 CAS:108-78-1 MolWeight:126 RetIndex:1597
CompName:1,3,5-Triazine-2,4,6-triamine $$ Melamine $$ S-Triazinetriamine $$ Cyanuramide $$ Cyanurotriamide $$ Cyanurotria

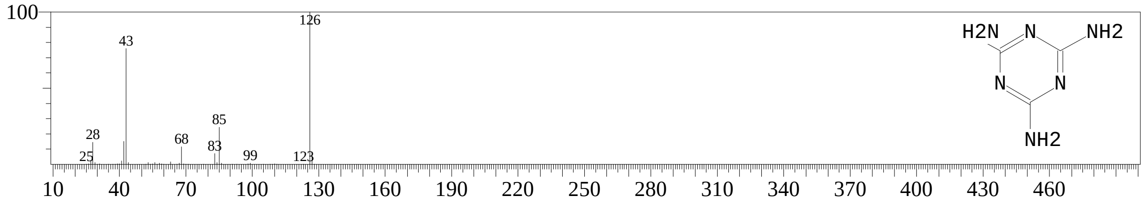

Hit#:2 Entry:6245 Library:NIST05.LIB
SI:82 Formula:C6H6O3 CAS:118-71-8 MolWeight:126 RetIndex:1063
CompName:Maltol $$ 4H-Pyran-4-one, 3-hydroxy-2-methyl- $$ Larixic acid $$ Larixinic acid $$ Palatone $$ 2-Methyl-3-hydroxyp

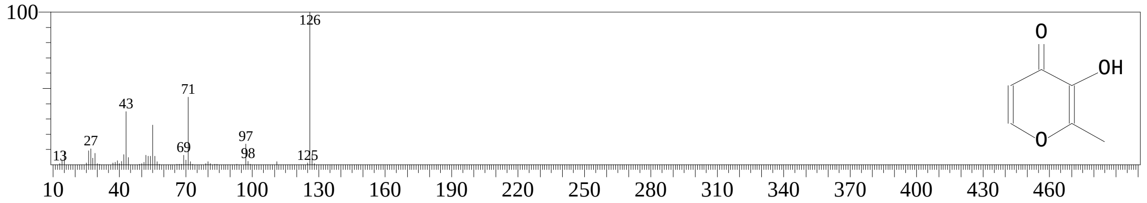

Hit#:3 Entry:4524 Library:NIST05s.LIB
SI:82 Formula:C6H6O3 CAS:118-71-8 MolWeight:126 RetIndex:1063
CompName:Maltol $$ 4H-Pyran-4-one, 3-hydroxy-2-methyl- $$ Larixic acid $$ Larixinic acid $$ Palatone $$ 2-Methyl-3-hydroxyp

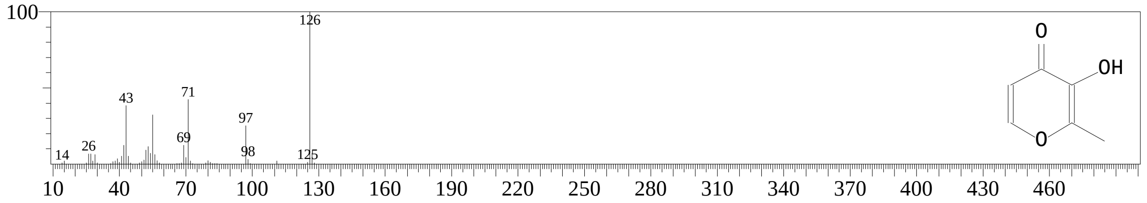

Hit#:4 Entry:4523 Library:NIST05s.LIB
SI:81 Formula:C6H6O3 CAS:118-71-8 MolWeight:126 RetIndex:1063
CompName:Maltol $$ 4H-Pyran-4-one, 3-hydroxy-2-methyl- $$ Larixic acid $$ Larixinic acid $$ Palatone $$ 2-Methyl-3-hydroxyp

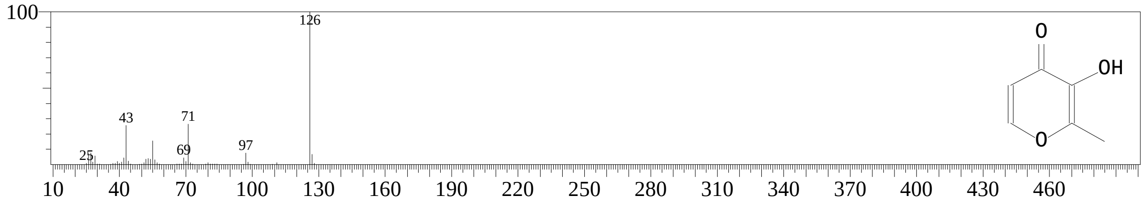

Hit#:5 Entry:6285 Library:NIST05.LIB
SI:81 Formula:C7H10O2 CAS:15121-2-5 MolWeight:126 RetIndex:920
CompName:Cyclopentane, 1-acetyl-1,2-epoxy- $$ 1-(6-Oxabicyclo[3.1.0]hex-1-yl)ethanone # $$

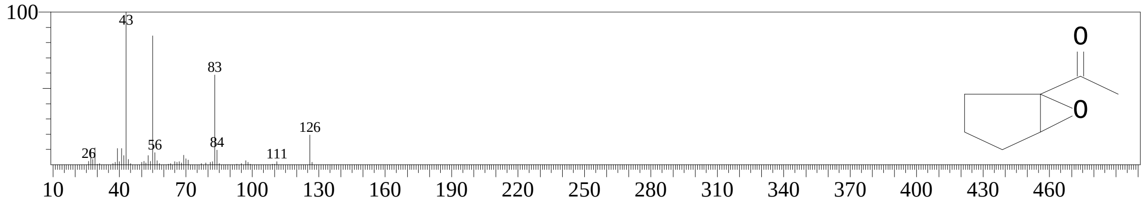

<< Target >>
Line#:6   R.Time:11.483(Scan#:1019) MassPeaks:252   BasePeak:56.95(8940)
RawMode:Averaged 11.475-11.492(1018-1020)   BG Mode:Calc. from Peak

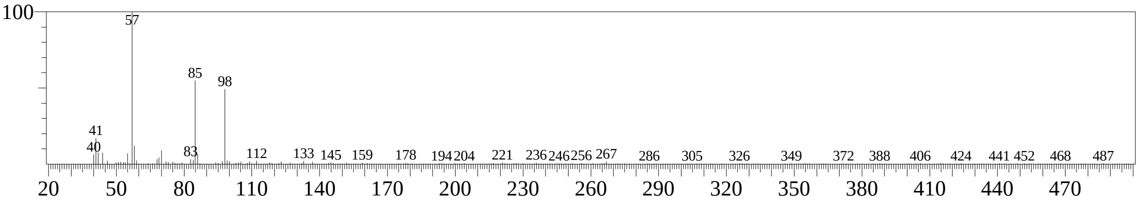

Hit#:1 Entry:11949 Library:NIST05.LIB
SI:78 Formula:C9H18O CAS:7492-38-8 MolWeight:142 RetIndex:988
CompName:4-Octanone, 2-methyl- $$ 2-Methyl-4-octanone $$

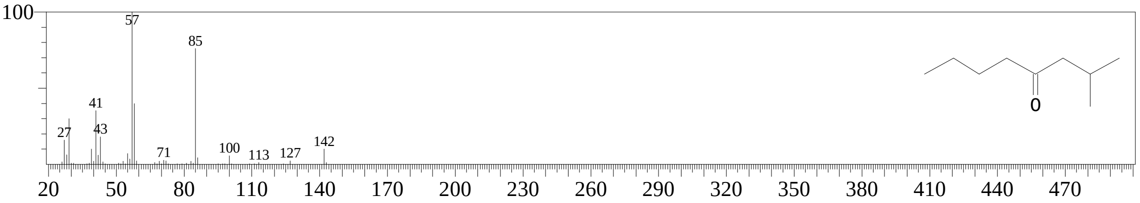

Hit#:2 Entry:4045 Library:NIST05.LIB
SI:78 Formula:C6H15Al CAS:97-93-8 MolWeight:114 RetIndex:0
CompName:Aluminum, triethyl- $$ Triethylaluminum $$ TEA $$ Triethylaluminium $$ UN 1102 $$

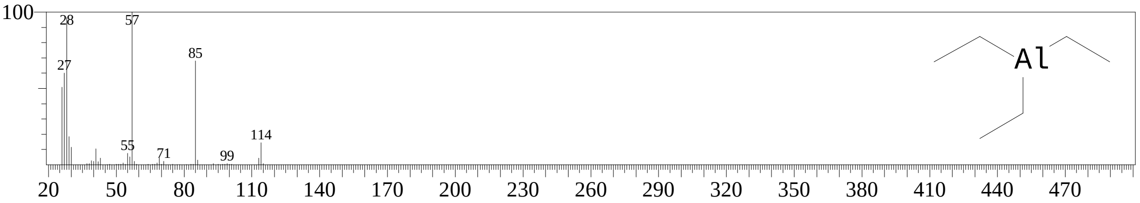

Hit#:3 Entry:12500 Library:NIST05.LIB
SI:76 Formula:C7H12O3 CAS:6395-83-1 MolWeight:144 RetIndex:1020
CompName:Hexanoic acid, 2-oxo-, methyl ester $$ Methyl 2-oxohexanoate # $$

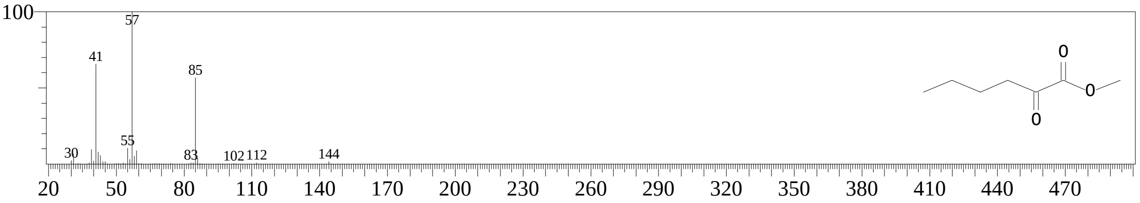

Hit#:4 Entry:40054 Library:NIST05.LIB
SI:76 Formula:C12H22O2 CAS:0-0-0 MolWeight:198 RetIndex:1273
CompName:3,5-Octanedione, 2,2,4,7-tetramethyl-

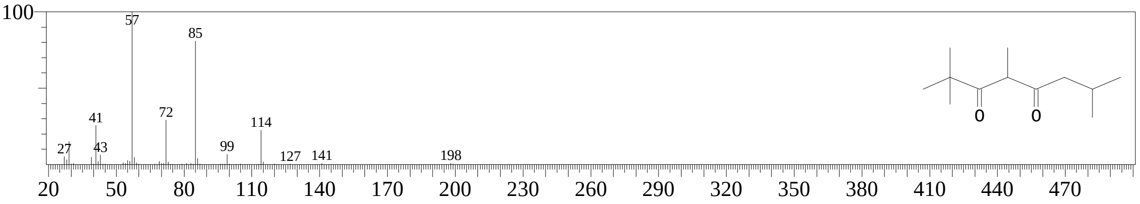

Hit#:5 Entry:23745 Library:NIST05.LIB
SI:76 Formula:C11H20O CAS:0-0-0 MolWeight:168 RetIndex:1174
CompName:2,2-Dimethylnon-5-en-3-one

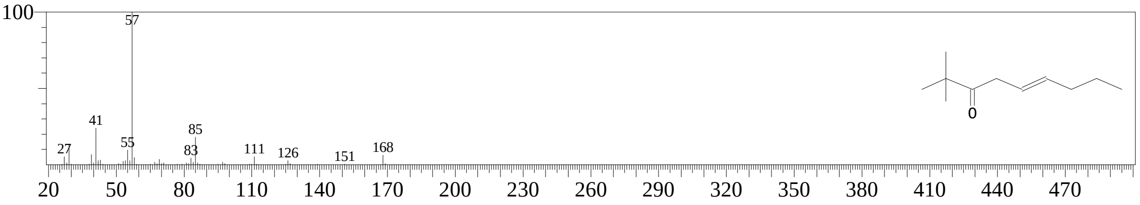

<< Target >>
Line#:7   R.Time:11.683(Scan#:1043) MassPeaks:247   BasePeak:43.00(29897)
RawMode:Averaged 11.675-11.692(1042-1044)   BG Mode:Calc. from Peak

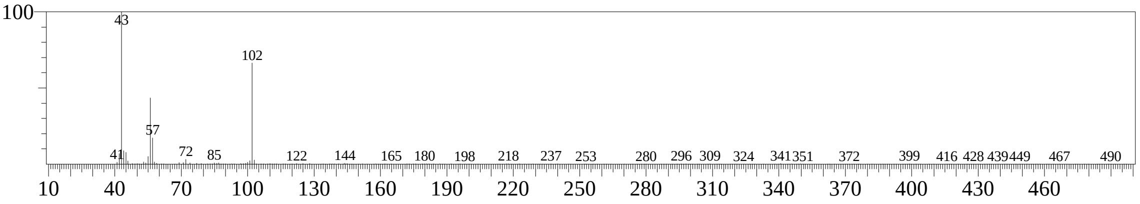

Hit#:1 Entry:3404 Library:NIST05s.LIB
SI:83 Formula:C5H8O3 CAS:123-76-2 MolWeight:116 RetIndex:1011
CompName:Pentanoic acid, 4-oxo- $$ Levulinic acid $$ .beta.-Acetylpropionic acid $$ .gamma.-Ketovaleric acid $$ Laevulinic aci

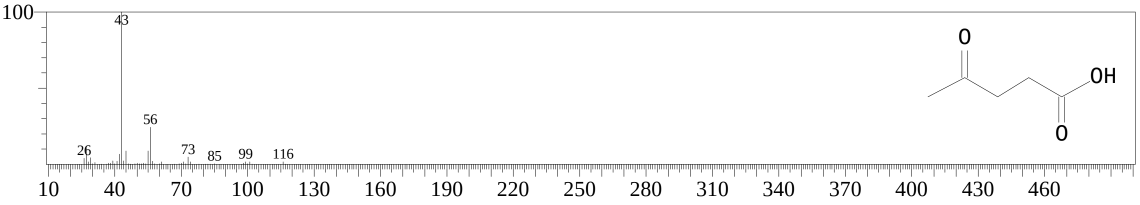

Hit#:2 Entry:4401 Library:NIST05.LIB
SI:83 Formula:C5H8O3 CAS:123-76-2 MolWeight:116 RetIndex:1011
CompName:Pentanoic acid, 4-oxo- $$ Levulinic acid $$ .beta.-Acetylpropionic acid $$ .gamma.-Ketovaleric acid $$ Laevulinic aci

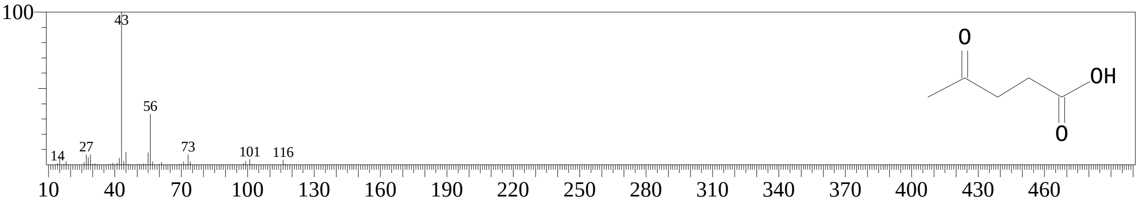

Hit#:3 Entry:3405 Library:NIST05s.LIB
SI:82 Formula:C5H8O3 CAS:123-76-2 MolWeight:116 RetIndex:1011
CompName:Pentanoic acid, 4-oxo- $$ Levulinic acid $$ .beta.-Acetylpropionic acid $$ .gamma.-Ketovaleric acid $$ Laevulinic aci

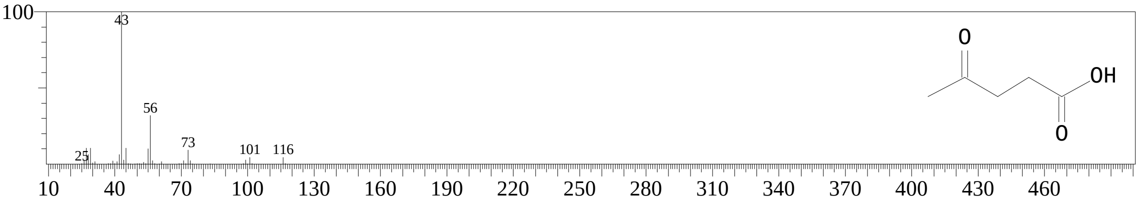

Hit#:4 Entry:7443 Library:NIST05s.LIB
SI:82 Formula:C7H12O3 CAS:609-14-3 MolWeight:144 RetIndex:956
CompName:Butanoic acid, 2-methyl-3-oxo-, ethyl ester $$ Acetoacetic acid, 2-methyl-, ethyl ester $$ .alpha.-Methylacetoacetic este

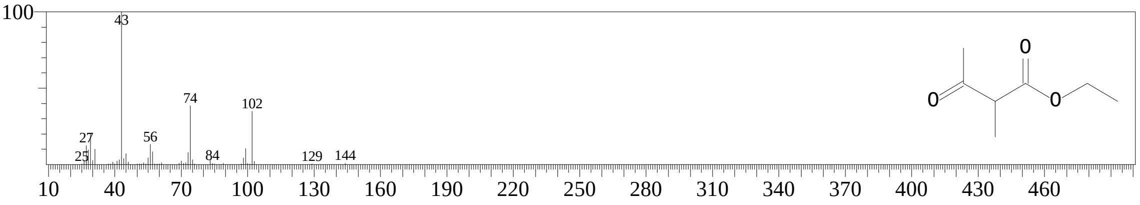

Hit#:5 Entry:7446 Library:NIST05s.LIB
SI:82 Formula:C7H12O3 CAS:609-14-3 MolWeight:144 RetIndex:956
CompName:Butanoic acid, 2-methyl-3-oxo-, ethyl ester $$ Acetoacetic acid, 2-methyl-, ethyl ester $$ .alpha.-Methylacetoacetic este

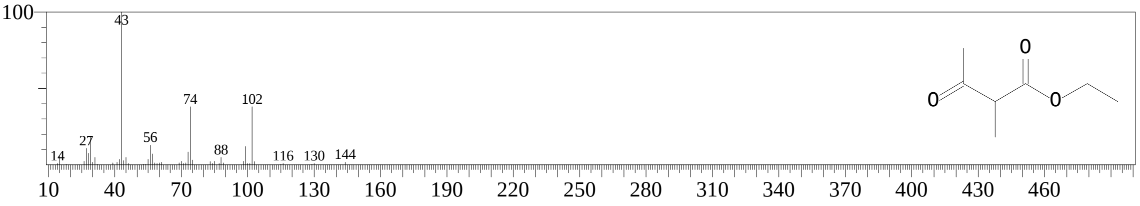

<< Target >>
Line#:8   R.Time:11.808(Scan#:1058) MassPeaks:303   BasePeak:43.00(125624)
RawMode:Averaged 11.800-11.817(1057-1059)   BG Mode:Calc. from Peak

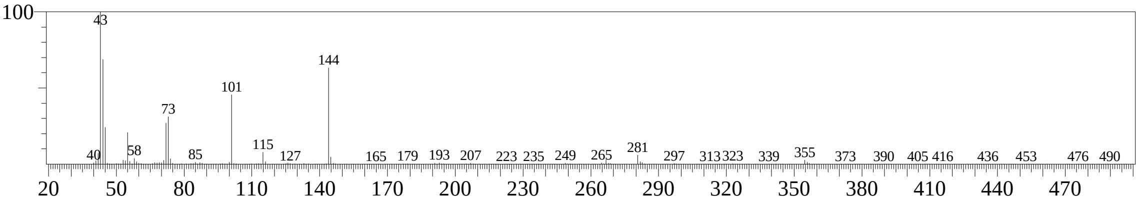

Hit#:1 Entry:7403 Library:NIST05s.LIB
SI:94 Formula:C6H8O4 CAS:28564-83-2 MolWeight:144 RetIndex:1269
CompName:4H-Pyran-4-one, 2,3-dihydro-3,5-dihydroxy-6-methyl- $$ 3,5-Dihydroxy-6-methyl-2,3-dihydro-4H-pyran-4-one # $$

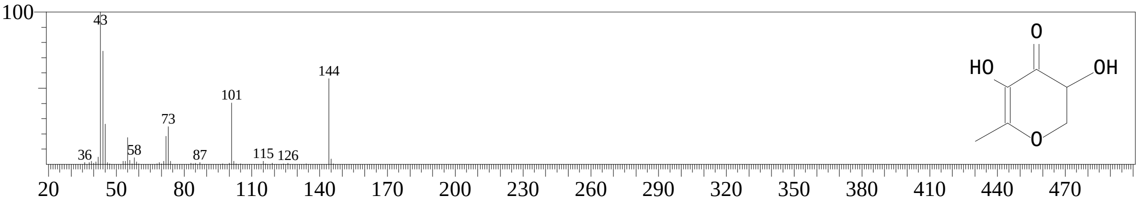

Hit#:2 Entry:12367 Library:NIST05.LIB
SI:89 Formula:C6H8O4 CAS:28564-83-2 MolWeight:144 RetIndex:1269
CompName:4H-Pyran-4-one, 2,3-dihydro-3,5-dihydroxy-6-methyl- $$ 3,5-Dihydroxy-6-methyl-2,3-dihydro-4H-pyran-4-one # $$

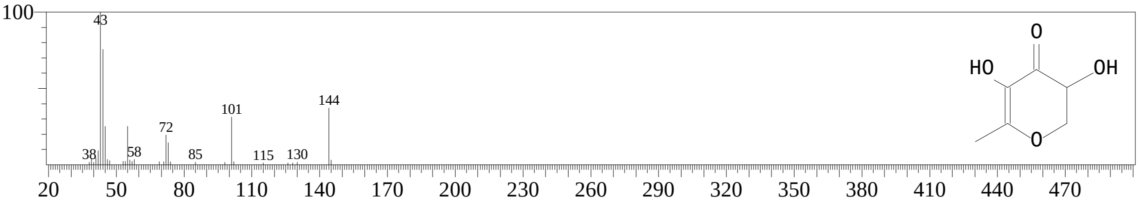

Hit#:3 Entry:4455 Library:NIST05.LIB
SI:80 Formula:C6H12O2 CAS:3299-32-9 MolWeight:116 RetIndex:761
CompName:1,3-Dioxolane, 2,4,5-trimethyl- $$ 2,4,5-Trimethyl-1,3-dioxolane # $$

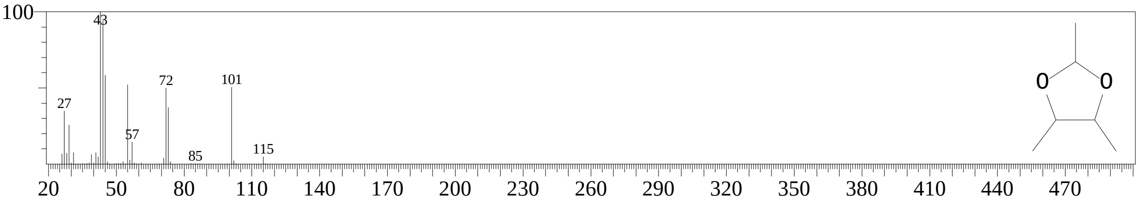

Hit#:4 Entry:12371 Library:NIST05.LIB
SI:80 Formula:C6H8O4 CAS:10230-62-3 MolWeight:144 RetIndex:1173
CompName:2,4-Dihydroxy-2,5-dimethyl-3(2H)-furan-3-one $$ 2,4-Dihydroxy-2,5-dimethyl-3(2H)-furanone # $$

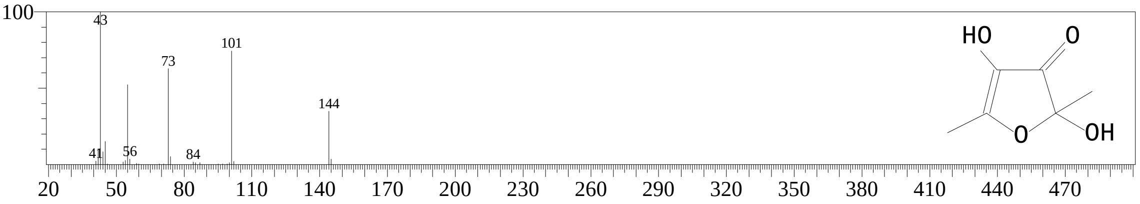

Hit#:5 Entry:12435 Library:NIST05.LIB
SI:76 Formula:C6H16N4 CAS:14866-81-0 MolWeight:144 RetIndex:886
CompName:2-Tetrazene, 1,1-diethyl-4,4-dimethyl- $$ 1,1-Dimethyl-4,4-diethyl-.DELTA.[2]-tetrazene $$ (2E)-1,1-Diethyl-4,4-dim

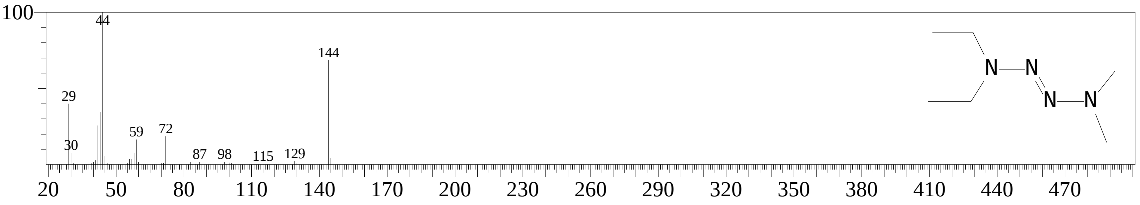

<< Target >>
Line#:9 R.Time:12.583(Scan#:1151) MassPeaks:249 BasePeak:141.95(4514)
RawMode:Averaged 12.575-12.592(1150-1152) BG Mode:Calc. from Peak

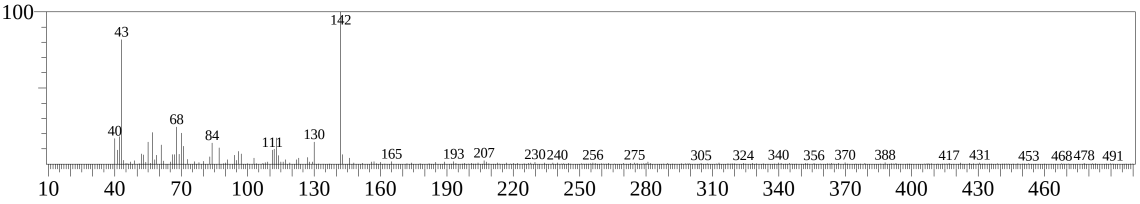

Hit#:1 Entry:11541 Library:NIST05.LIB
SI:76 Formula:C6H6O4 CAS:1073-96-7 MolWeight:142 RetIndex:1193
CompName:4H-Pyran-4-one, 3,5-dihydroxy-2-methyl- $$ 3,5-Dihydroxy-2-methyl-4-pyrone $$ 3,5-Dihydroxy-2-methyl-4H-pyran-

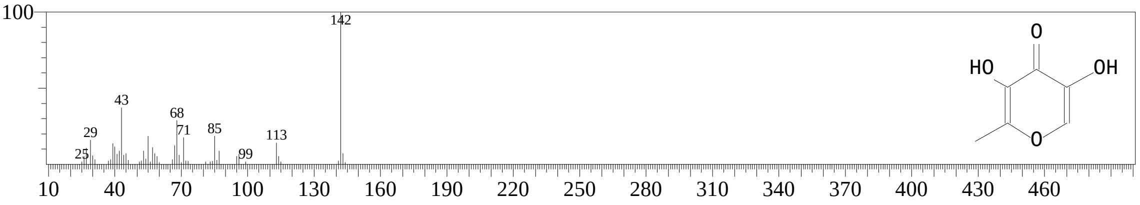

Hit#:2 Entry:7093 Library:NIST05s.LIB
SI:73 Formula:C6H6O4 CAS:1073-96-7 MolWeight:142 RetIndex:1193
CompName:4H-Pyran-4-one, 3,5-dihydroxy-2-methyl- $$ 3,5-Dihydroxy-2-methyl-4-pyrone $$ 3,5-Dihydroxy-2-methyl-4H-pyran-

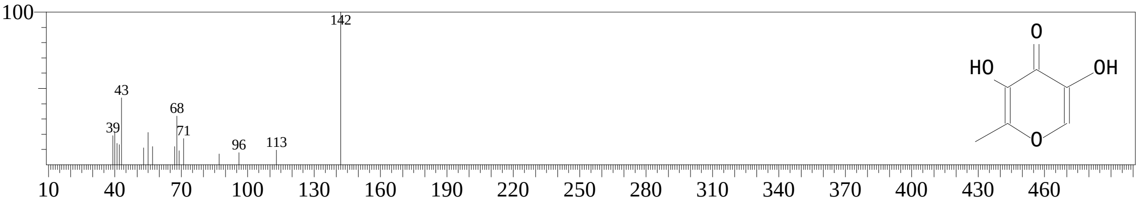

Hit#:3 Entry:11542 Library:NIST05.LIB
SI:72 Formula:C6H6O4 CAS:501-30-4 MolWeight:142 RetIndex:1306
CompName:4H-Pyran-4-one, 5-hydroxy-2-(hydroxymethyl)- $$ Kojic acid $$ 5-Hydroxy-2-(hydroxymethyl)-4-pyrone $$ 5-Hydrox

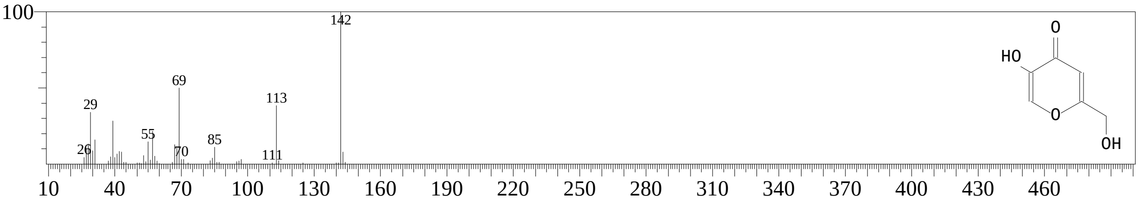

Hit#:4 Entry:7095 Library:NIST05s.LIB
SI:71 Formula:C6H6O4 CAS:501-30-4 MolWeight:142 RetIndex:1306
CompName:4H-Pyran-4-one, 5-hydroxy-2-(hydroxymethyl)- $$ Kojic acid $$ 5-Hydroxy-2-(hydroxymethyl)-4-pyrone $$ 5-Hydrox

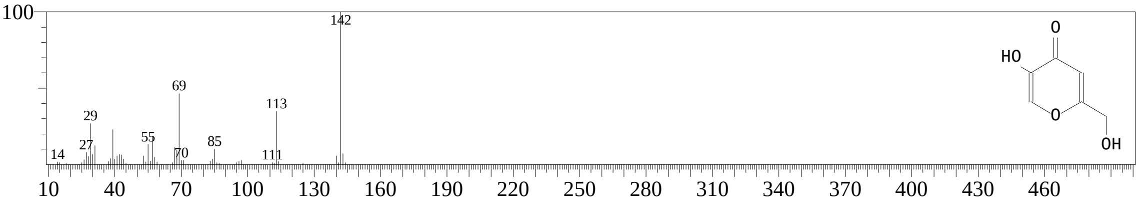

Hit#:5 Entry:11584 Library:NIST05.LIB
SI:71 Formula:C6H10N2O2 CAS:4874-13-9 MolWeight:142 RetIndex:1361
CompName:2,4(1H,3H)-Pyrimidinedione, dihydro-1,3-dimethyl- $$ 1,3-Dimethyldihydro-2,4(1H,3H)-pyrimidinedione # $$

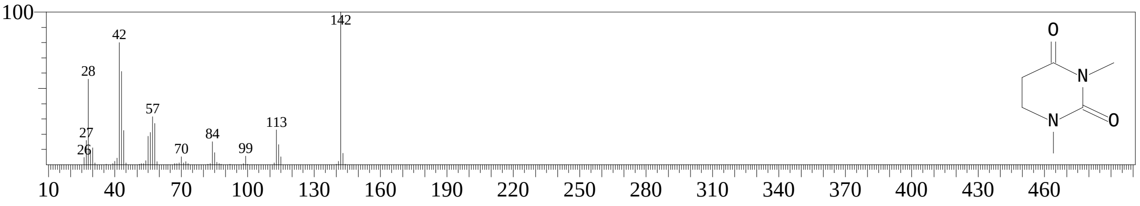

<< Target >>
Line#:10   R.Time:12.975(Scan#:1198) MassPeaks:236   BasePeak:85.95(19851)
RawMode:Averaged 12.967-12.983(1197-1199)   BG Mode:Calc. from Peak

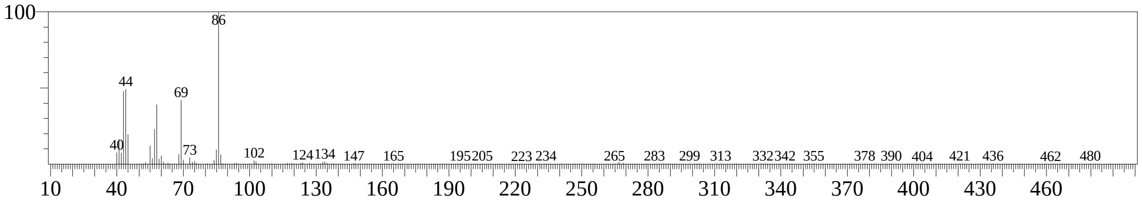

Hit#:1 Entry:13158 Library:NIST05.LIB
SI:92 Formula:C6H10O4 CAS:0-0-0 MolWeight:146 RetIndex:1216
CompName:Dianhydromannitol

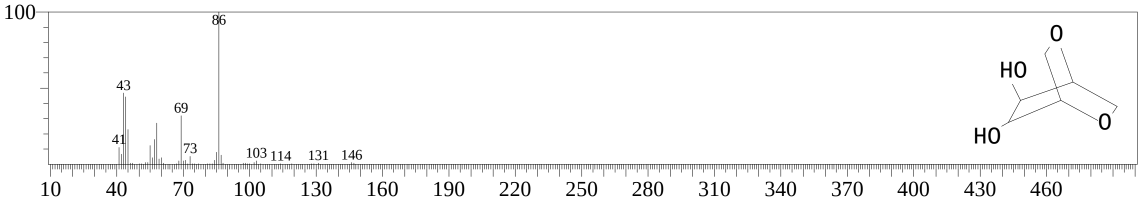

Hit#:2 Entry:7715 Library:NIST05s.LIB
SI:91 Formula:C6H10O4 CAS:652-67-5 MolWeight:146 RetIndex:1216
CompName:Isosorbide $$ D-Glucitol, 1,4:3,6-dianhydro- $$ (+)-D-Isosorbide $$ AT 101 $$ D-Sorbitol, 1,4:3,6-dianhydro(furo(3,2

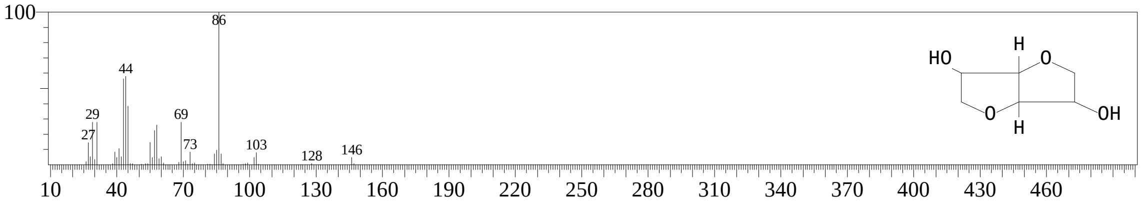

Hit#:3 Entry:13159 Library:NIST05.LIB
SI:91 Formula:C6H10O4 CAS:652-67-5 MolWeight:146 RetIndex:1216
CompName:Isosorbide $$ D-Glucitol, 1,4:3,6-dianhydro- $$ (+)-D-Isosorbide $$ AT 101 $$ D-Sorbitol, 1,4:3,6-dianhydro(furo(3,2

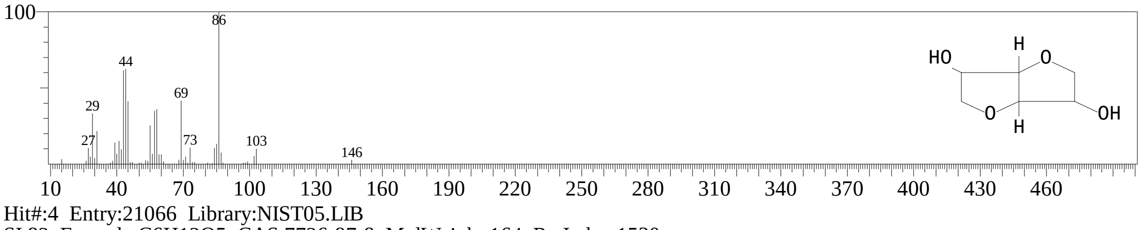

Hit#:4 Entry:21066 Library:NIST05.LIB
SI:82 Formula:C6H12O5 CAS:7726-97-8 MolWeight:164 RetIndex:1530
CompName:d-Mannitol, 1,4-anhydro- $$ 1,4-Anhydro-d-mannitol $$ 1,4-Anhydrohexitol # $$

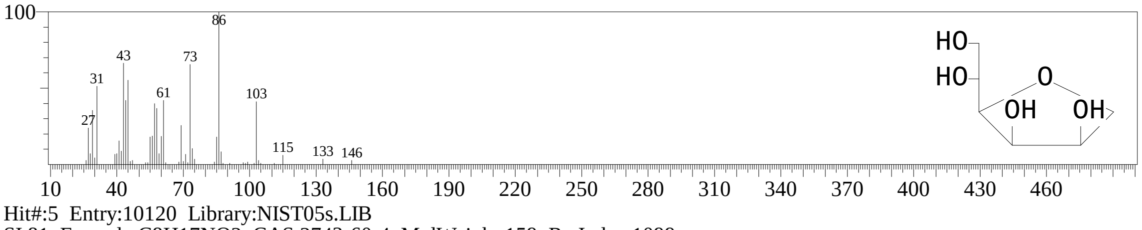

Hit#:5 Entry:10120 Library:NIST05s.LIB
SI:81 Formula:C8H17NO2 CAS:2743-60-4 MolWeight:159 RetIndex:1099
CompName:L-Leucine, ethyl ester $$ Leucine, ethyl ester, L- $$ (+)-L-Leucine ethyl ester $$ Ethyl leucinate $$ Ethyl L-leucinate $

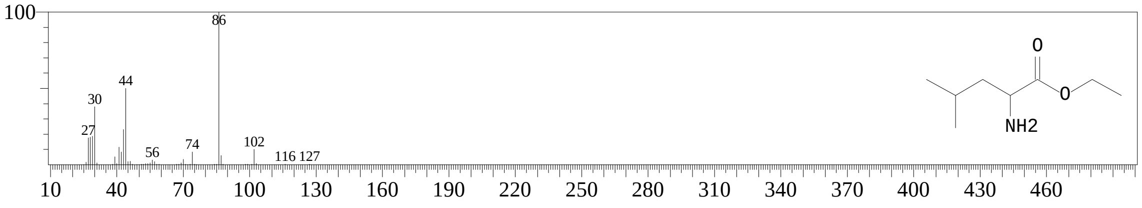

<< Target >>
Line#:11 R.Time:13.258(Scan#:1232) MassPeaks:280 BasePeak:96.95(1180369)
RawMode:Averaged 13.250-13.267(1231-1233) BG Mode:Calc. from Peak

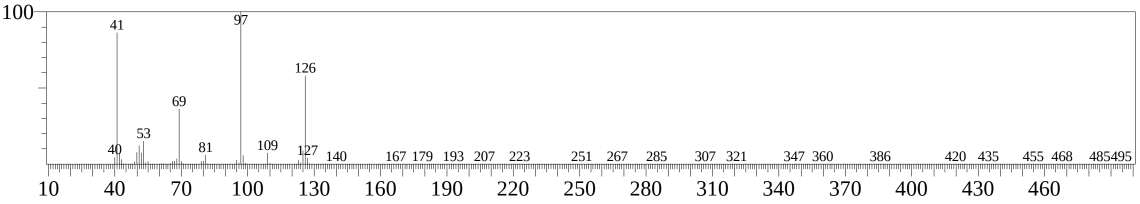

Hit#:1 Entry:6237 Library:NIST05.LIB
SI:95 Formula:C6H6O3 CAS:67-47-0 MolWeight:126 RetIndex:1163
CompName:2-Furancarboxaldehyde, 5-(hydroxymethyl)- $$ 2-Furaldehyde, 5-(hydroxymethyl)- $$ 5-Hydrxoymethylfurfural $$ Hy

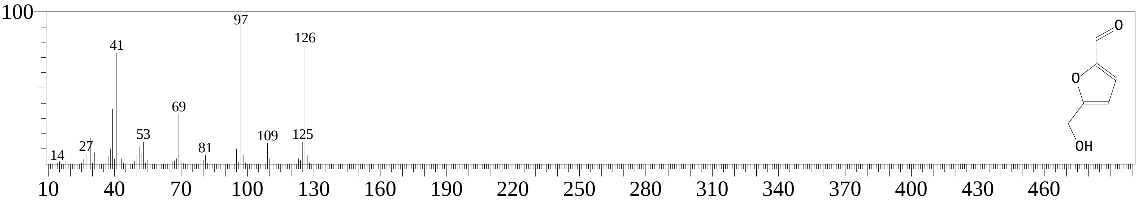

Hit#:2 Entry:4517 Library:NIST05s.LIB
SI:94 Formula:C6H6O3 CAS:67-47-0 MolWeight:126 RetIndex:1163
CompName:2-Furancarboxaldehyde, 5-(hydroxymethyl)- $$ 2-Furaldehyde, 5-(hydroxymethyl)- $$ 5-Hydrxoymethylfurfural $$ Hy

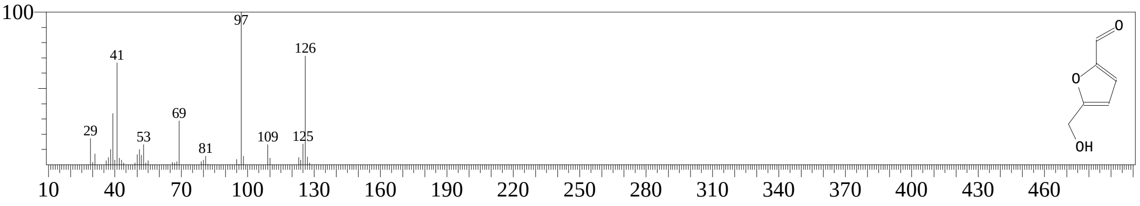

Hit#:3 Entry:6495 Library:NIST05.LIB
SI:84 Formula:C8H14O CAS:1447-26-3 MolWeight:126 RetIndex:938
CompName:4-Hepten-3-one, 5-methyl- $$ (4E)-5-Methyl-4-hepten-3-one # $$

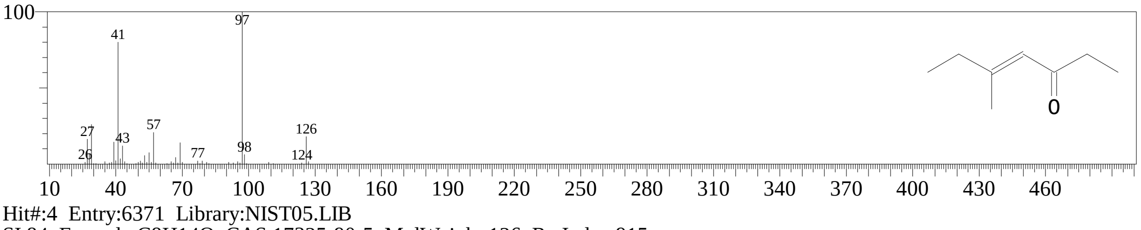

Hit#:4 Entry:6371 Library:NIST05.LIB
SI:84 Formula:C8H14O CAS:17325-90-5 MolWeight:126 RetIndex:915
CompName:4-Hexen-3-one, 4,5-dimethyl- $$ 4,5-Dimethyl-4-hexen-3-one # $$

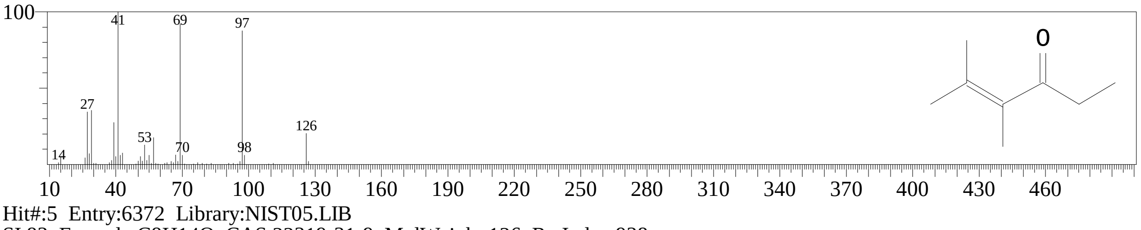

Hit#:5 Entry:6372 Library:NIST05.LIB
SI:83 Formula:C8H14O CAS:22319-31-9 MolWeight:126 RetIndex:938
CompName:4-Hepten-3-one, 4-methyl- $$ (4E)-4-Methyl-4-hepten-3-one # $$

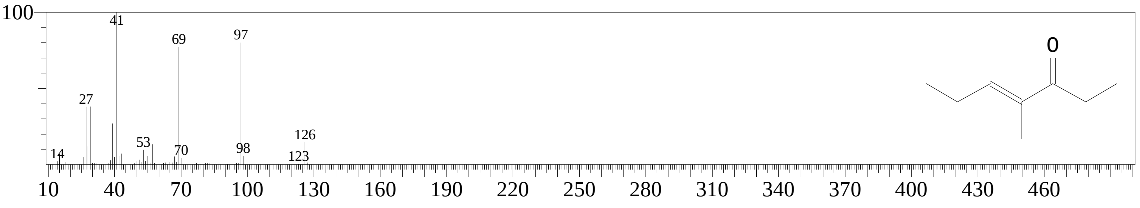

<< Target >>
Line#:12 R.Time:15.917(Scan#:1551) MassPeaks:262 BasePeak:41.00(6279)
RawMode:Averaged 15.908-15.925(1550-1552) BG Mode:Calc. from Peak

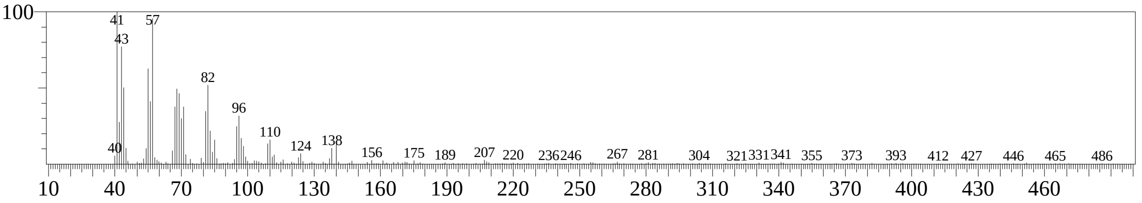

Hit#:1 Entry:14087 Library:NIST05s.LIB
SI:95 Formula:C12H24O CAS:112-54-9 MolWeight:184 RetIndex:1402
CompName:Dodecanal $$ Lauraldehyde $$ n-Dodecanal $$ n-Dodecyl aldehyde $$ n-Dodecylic aldehyde $$ n-Lauraldehyde $$ A

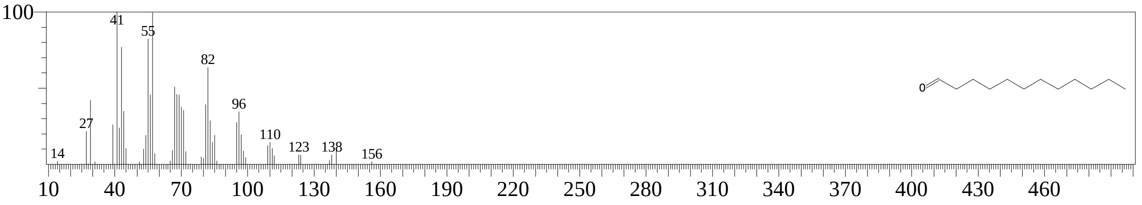

Hit#:2 Entry:14089 Library:NIST05s.LIB
SI:93 Formula:C12H24O CAS:112-54-9 MolWeight:184 RetIndex:1402
CompName:Dodecanal $$ Lauraldehyde $$ n-Dodecanal $$ n-Dodecyl aldehyde $$ n-Dodecylic aldehyde $$ n-Lauraldehyde $$ A

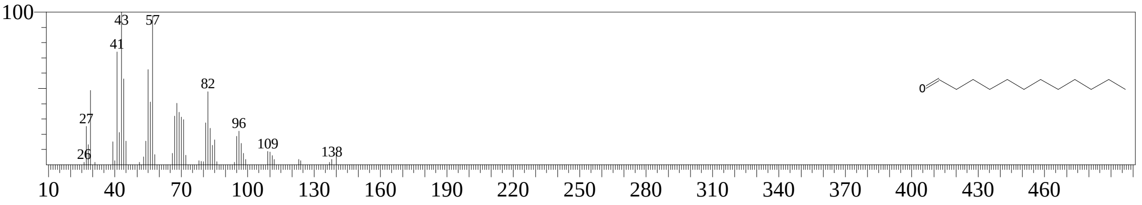

Hit#:3 Entry:17684 Library:NIST05s.LIB
SI:92 Formula:C14H28O CAS:124-25-4 MolWeight:212 RetIndex:1601
CompName:Tetradecanal $$ Myristaldehyde $$ Myristylaldehyde $$ Tetradecylaldehyde $$ n-Tetradecanal $$ Aldehyde C-14 $$ A

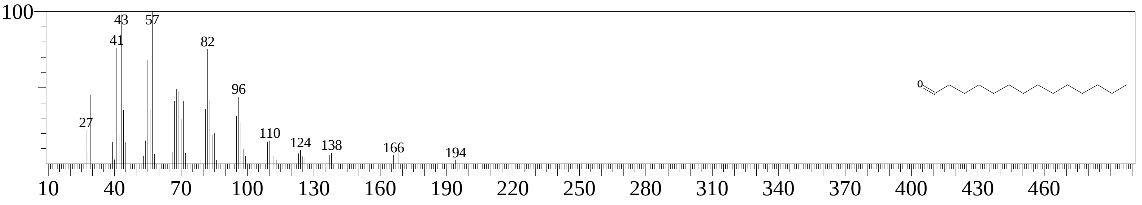

Hit#:4 Entry:65429 Library:NIST05.LIB
SI:92 Formula:C16H32O CAS:629-80-1 MolWeight:240 RetIndex:1800
CompName:Hexadecanal $$ Palmitaldehyde $$

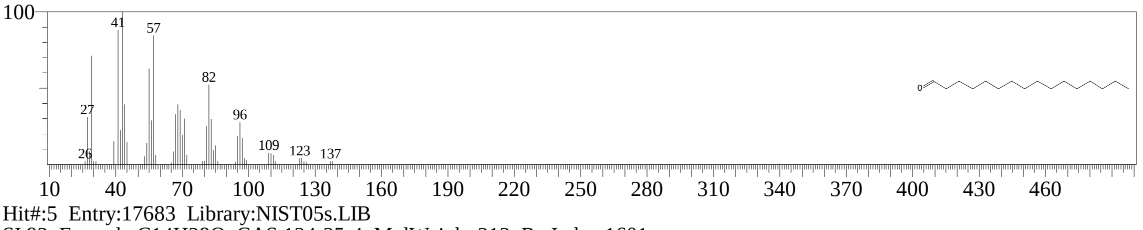

Hit#:5 Entry:17683 Library:NIST05s.LIB
SI:92 Formula:C14H28O CAS:124-25-4 MolWeight:212 RetIndex:1601
CompName:Tetradecanal $$ Myristaldehyde $$ Myristylaldehyde $$ Tetradecylaldehyde $$ n-Tetradecanal $$ Aldehyde C-14 $$ A

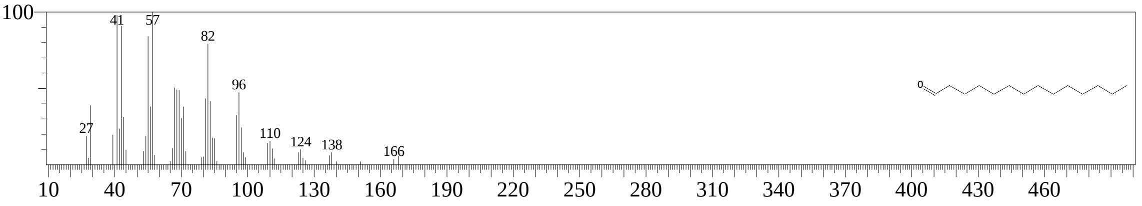

<< Target >>
Line#:13   R.Time:16.700(Scan#:1645) MassPeaks:303   BasePeak:57.00(177319)
RawMode:Averaged 16.692-16.708(1644-1646)   BG Mode:Calc. from Peak

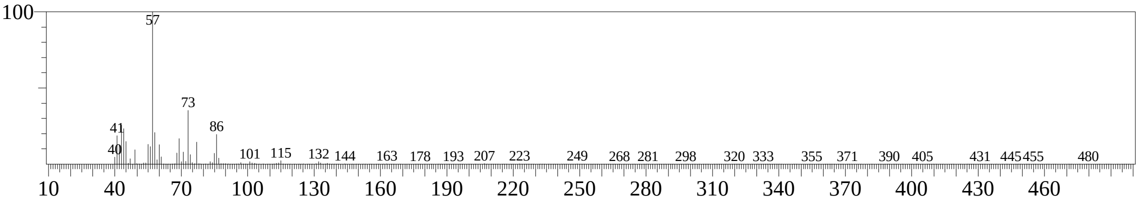

Hit#:1 Entry:8563 Library:NIST05s.LIB
SI:83 Formula:C4H9NO5 CAS:126-11-4 MolWeight:151 RetIndex:1444
CompName:1,3-Propanediol, 2-(hydroxymethyl)-2-nitro- $$ Isobutylglycerol, nitro- $$ Methane, trimethylolnitro- $$ Nitroisobutylg

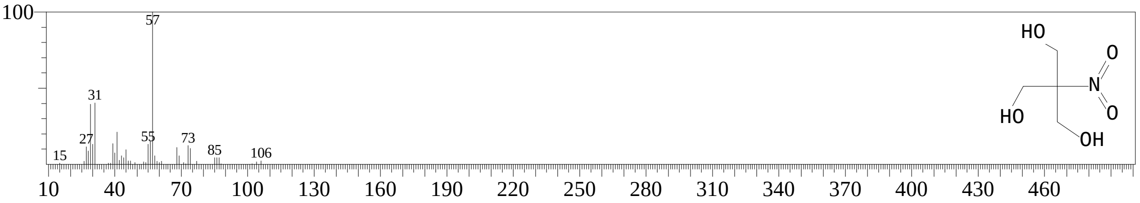

Hit#:2 Entry:123174 Library:NIST05.LIB
SI:83 Formula:C12H22O11 CAS:57-50-1 MolWeight:342 RetIndex:3139
CompName:Sucrose $$ .alpha.-D-Glucopyranoside, .beta.-D-fructofuranosyl $$ .beta.-D-Fructofuranosyl .alpha.-D-glucopyranoside

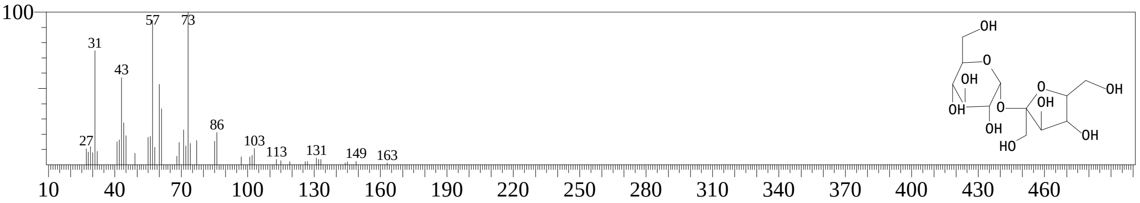

Hit#:3 Entry:15081 Library:NIST05.LIB
SI:82 Formula:C4H9NO5 CAS:126-11-4 MolWeight:151 RetIndex:1444
CompName:1,3-Propanediol, 2-(hydroxymethyl)-2-nitro- $$ Isobutylglycerol, nitro- $$ Methane, trimethylolnitro- $$ Nitroisobutylg

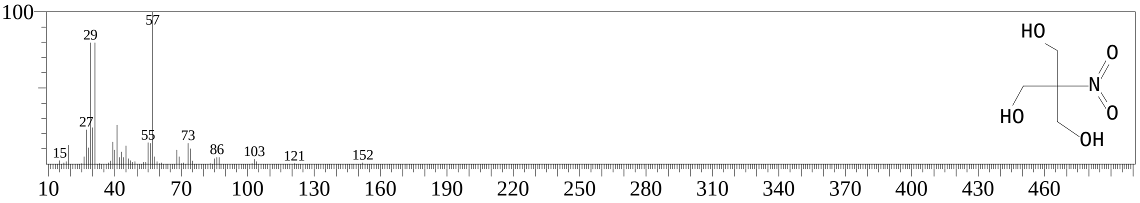

Hit#:4 Entry:5797 Library:NIST05s.LIB
SI:82 Formula:C6H14O3 CAS:77-99-6 MolWeight:134 RetIndex:1261
CompName:1,3-Propanediol, 2-ethyl-2-(hydroxymethyl)- $$ Ethriol $$ Ethyltrimethylolmethane $$ Etriol $$ Ettriol $$ Hexaglyceri

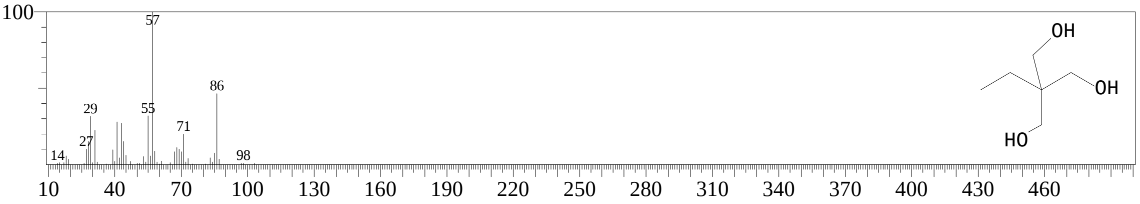

Hit#:5 Entry:1952 Library:NIST05s.LIB
SI:81 Formula:C4H6O3 CAS:108-32-7 MolWeight:102 RetIndex:875
CompName:Propylene Carbonate $$ 1,3-Dioxolan-2-one, 4-methyl- $$ Carbonic acid, cyclic propylene ester $$ Cyclic methylethyl

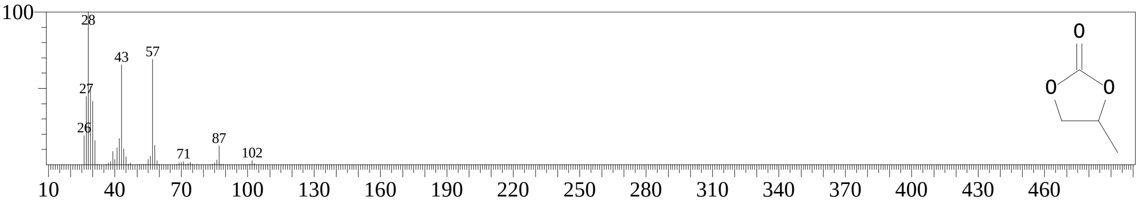

<< Target >>
Line#:14   R.Time:17.383(Scan#:1727) MassPeaks:278   BasePeak:191.05(12907)
RawMode:Averaged 17.375-17.392(1726-1728)   BG Mode:Calc. from Peak

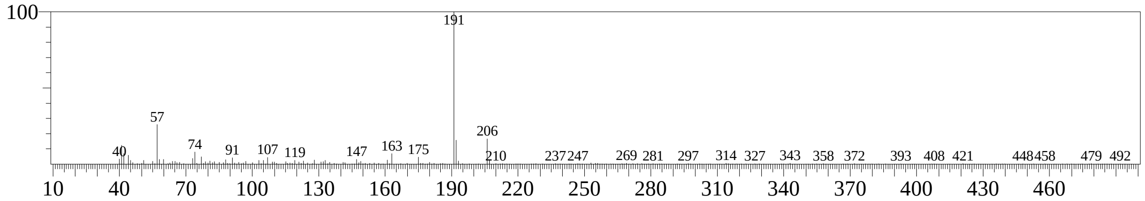

Hit#:1 Entry:16998 Library:NIST05s.LIB
SI:89 Formula:C14H22O CAS:96-76-4 MolWeight:206 RetIndex:1555
CompName:Phenol, 2,4-bis(1,1-dimethylethyl)- $$ Phenol, 2,4-di-tert-butyl- $$ 2,4-Di-tert-butylphenol $$ 2,4-di-t-Butylphenol $$

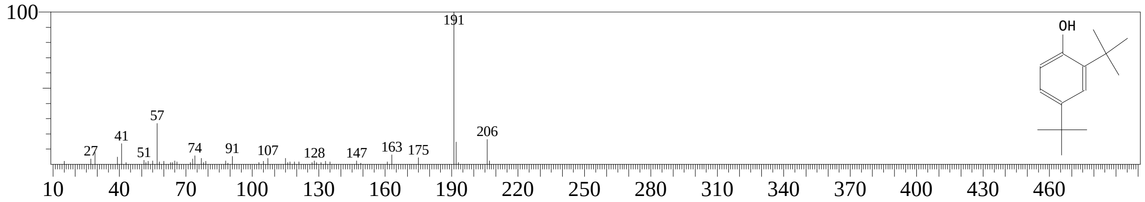

Hit#:2 Entry:44734 Library:NIST05.LIB
SI:88 Formula:C14H22O CAS:96-76-4 MolWeight:206 RetIndex:1555
CompName:Phenol, 2,4-bis(1,1-dimethylethyl)- $$ Phenol, 2,4-di-tert-butyl- $$ 2,4-Di-tert-butylphenol $$ 2,4-di-t-Butylphenol $$

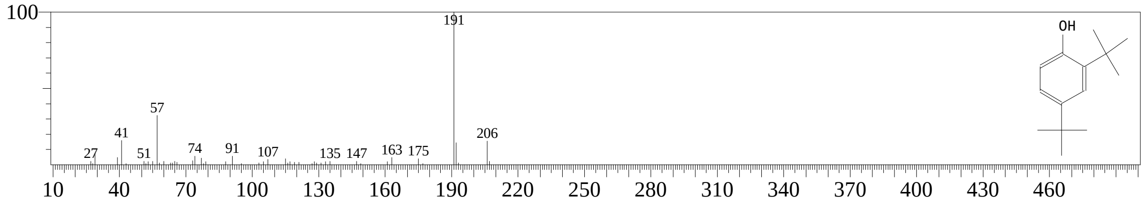

Hit#:3 Entry:16991 Library:NIST05s.LIB
SI:88 Formula:C14H22O CAS:96-76-4 MolWeight:206 RetIndex:1555
CompName:Phenol, 2,4-bis(1,1-dimethylethyl)- $$ Phenol, 2,4-di-tert-butyl- $$ 2,4-Di-tert-butylphenol $$ 2,4-di-t-Butylphenol $$

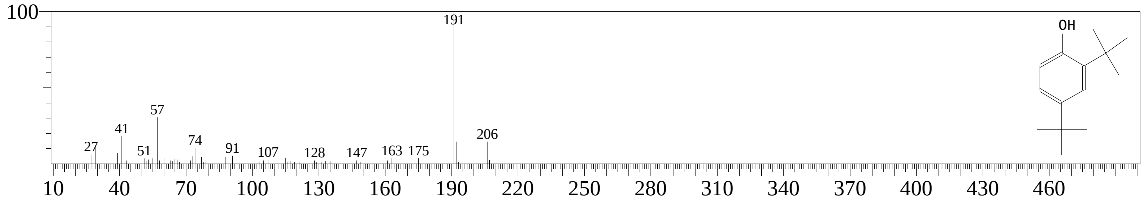

Hit#:4 Entry:16994 Library:NIST05s.LIB
SI:86 Formula:C14H22O CAS:5875-45-6 MolWeight:206 RetIndex:1555
CompName:Phenol, 2,5-bis(1,1-dimethylethyl)- $$ Phenol, 2,5-di-tert-butyl- $$ 2,5-Di-tert-butylphenol $$

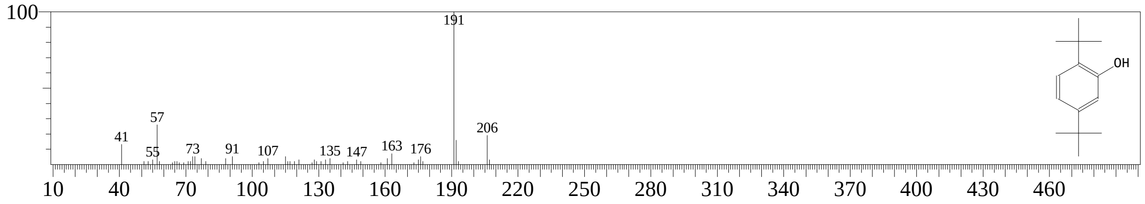

Hit#:5 Entry:44736 Library:NIST05.LIB
SI:86 Formula:C14H22O CAS:128-39-2 MolWeight:206 RetIndex:1555
CompName:Phenol, 2,6-bis(1,1-dimethylethyl)- $$ Phenol, 2,6-di-tert-butyl- $$ 2,6-Bis(tert-butyl)phenol $$ 2,6-Bis(1,1-dimethylet

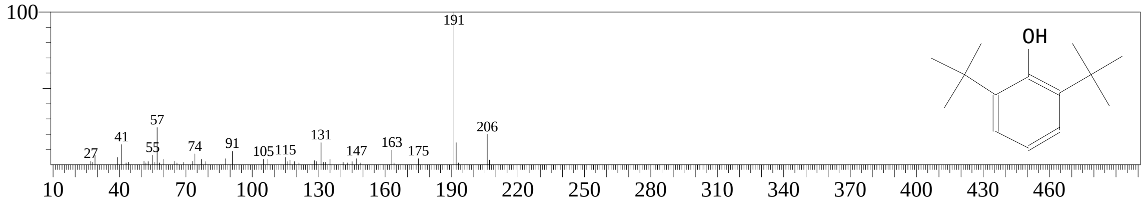

<< Target >>
Line#:15   R.Time:19.417(Scan#:1971) MassPeaks:249   BasePeak:43.00(2436)
RawMode:Averaged 19.408-19.425(1970-1972)   BG Mode:Calc. from Peak

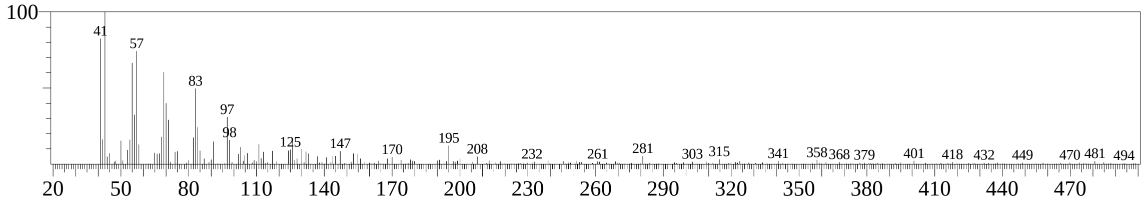

Hit#:1 Entry:114222 Library:NIST05.LIB
SI:83 Formula:C17H31F3O2 CAS:0-0-0 MolWeight:324 RetIndex:1713
CompName:Trifluoroacetic acid, n-heptadecyl ester

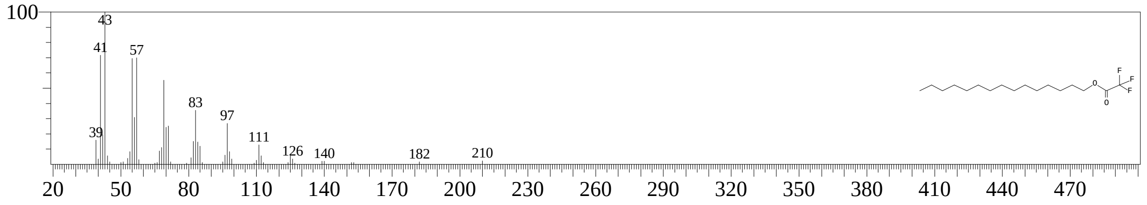

Hit#:2 Entry:17439 Library:NIST05s.LIB
SI:83 Formula:C15H30 CAS:13360-61-7 MolWeight:210 RetIndex:1502
CompName:1-Pentadecene $$ Pentadecene,1- $$ Pentadec-1-ene $$

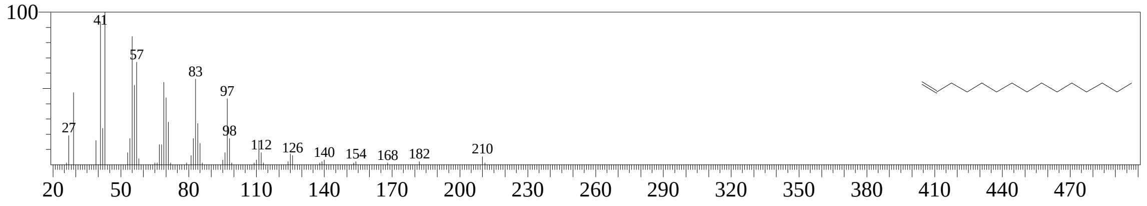

Hit#:3 Entry:15748 Library:NIST05s.LIB
SI:83 Formula:C14H28 CAS:1120-36-1 MolWeight:196 RetIndex:1403
CompName:1-Tetradecene $$ n-Tetradec-1-ene $$ .alpha.-Tetradecene $$ Neodene 14 $$ Tetradec-1-ene $$ Tetradecene-1 $$

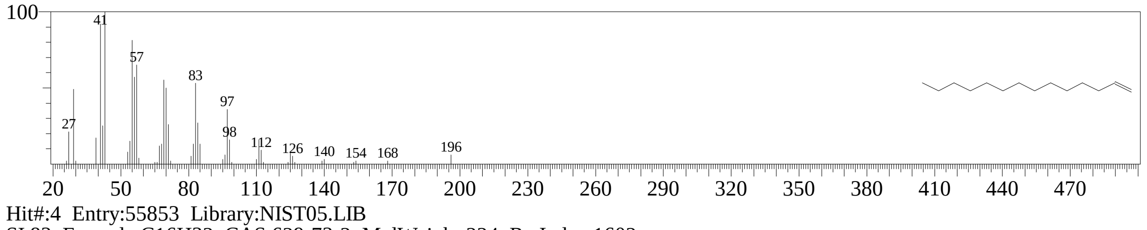

Hit#:4 Entry:55853 Library:NIST05.LIB
SI:83 Formula:C16H32 CAS:629-73-2 MolWeight:224 RetIndex:1602
CompName:1-Hexadecene $$ .alpha.-Hexadecene $$ n-Hexadec-1-ene $$ Cetene $$ 1-Cetene $$ Hexadecylene-1 $$ Hexadec-1-en

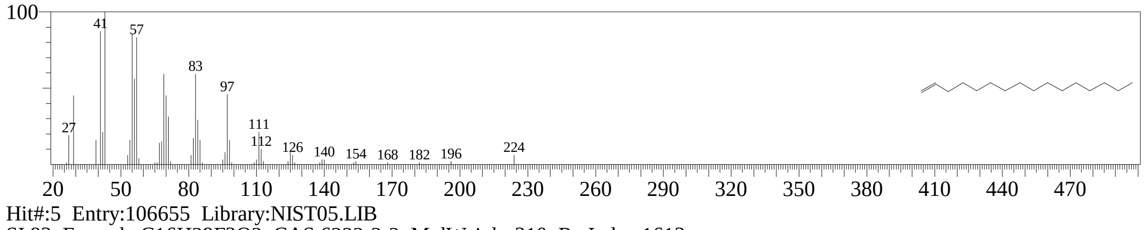

Hit#:5 Entry:106655 Library:NIST05.LIB
SI:83 Formula:C16H29F3O2 CAS:6222-2-2 MolWeight:310 RetIndex:1613
CompName:Acetic acid, trifluoro-, tetradecyl ester $$ Trifluoroacetic acid, n-tetradecyl ester $$ Tetradecyl trifluoroacetate # $$

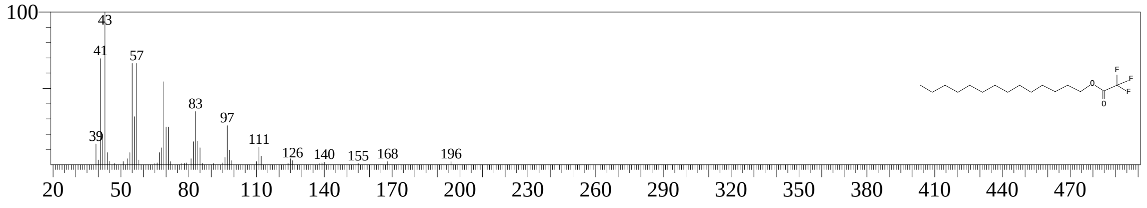

<< Target >>
Line#:16 R.Time:20.375(Scan#:2086) MassPeaks:251 BasePeak:59.95(2551)
RawMode:Averaged 20.367-20.383(2085-2087) BG Mode:Calc. from Peak

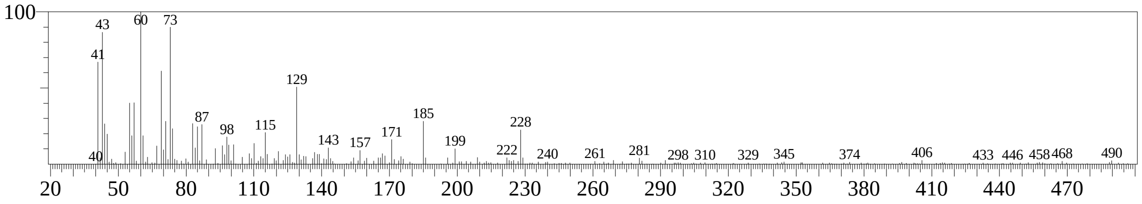

Hit#:1 Entry:19252 Library:NIST05s.LIB
SI:85 Formula:C14H28O2 CAS:544-63-8 MolWeight:228 RetIndex:1769
CompName:Tetradecanoic acid $$ Myristic acid $$ n-Tetradecanoic acid $$ n-Tetradecoic acid $$ Neo-Fat 14 $$ Univol U 316S $

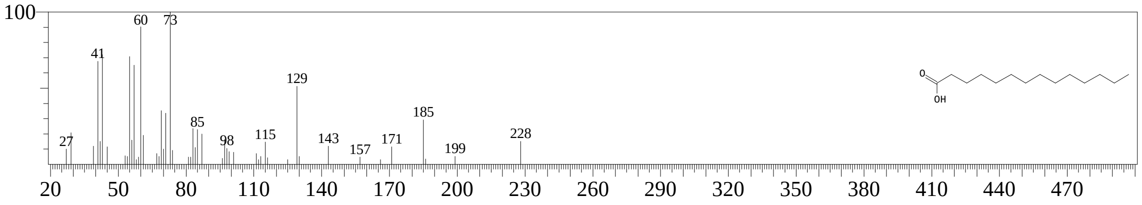

Hit#:2 Entry:19250 Library:NIST05s.LIB
SI:85 Formula:C14H28O2 CAS:544-63-8 MolWeight:228 RetIndex:1769
CompName:Tetradecanoic acid $$ Myristic acid $$ n-Tetradecanoic acid $$ n-Tetradecoic acid $$ Neo-Fat 14 $$ Univol U 316S $

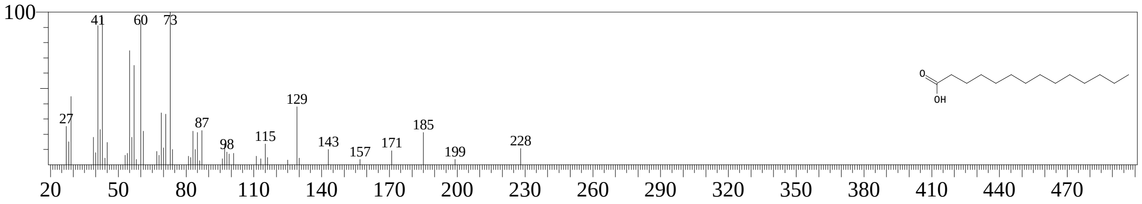

Hit#:3 Entry:58280 Library:NIST05.LIB
SI:84 Formula:C14H28O2 CAS:544-63-8 MolWeight:228 RetIndex:1769
CompName:Tetradecanoic acid $$ Myristic acid $$ n-Tetradecanoic acid $$ n-Tetradecoic acid $$ Neo-Fat 14 $$ Univol U 316S $

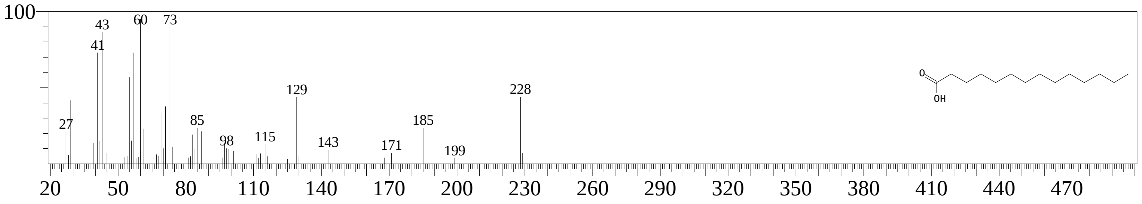

Hit#:4 Entry:19251 Library:NIST05s.LIB
SI:83 Formula:C14H28O2 CAS:544-63-8 MolWeight:228 RetIndex:1769
CompName:Tetradecanoic acid $$ Myristic acid $$ n-Tetradecanoic acid $$ n-Tetradecoic acid $$ Neo-Fat 14 $$ Univol U 316S $

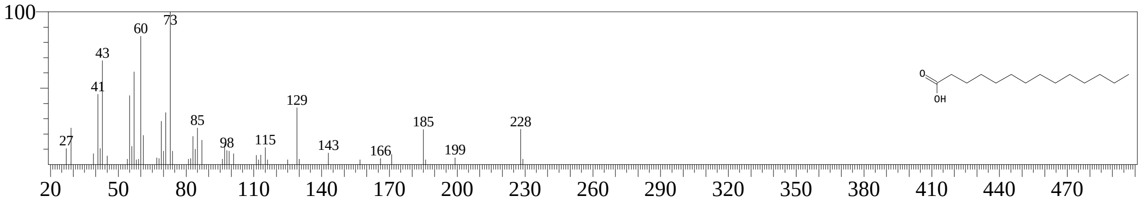

Hit#:5 Entry:20371 Library:NIST05s.LIB
SI:82 Formula:C15H30O2 CAS:1002-84-2 MolWeight:242 RetIndex:1869
CompName:Pentadecanoic acid $$ Pentadecylic acid $$ n-Pentadecanoic acid $$ n-Pentadecylic acid $$

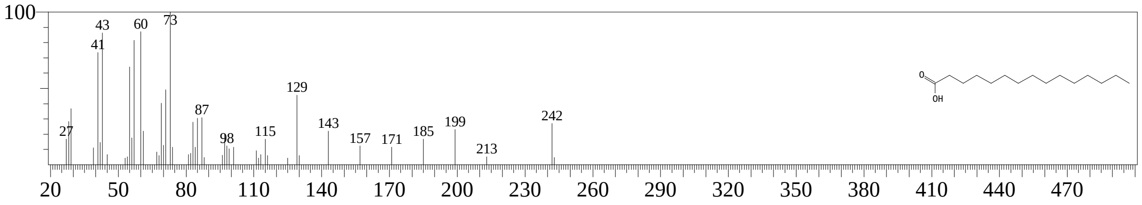

<< Target >>
Line#:17   R.Time:21.492(Scan#:2220) MassPeaks:260   BasePeak:73.00(1019)
RawMode:Averaged 21.483-21.500(2219-2221)   BG Mode:Calc. from Peak

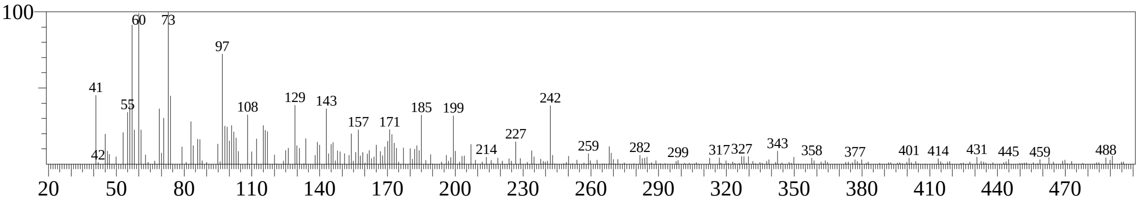

Hit#:1 Entry:20371 Library:NIST05s.LIB
SI:70 Formula:C15H30O2 CAS:1002-84-2 MolWeight:242 RetIndex:1869
CompName:Pentadecanoic acid $$ Pentadecylic acid $$ n-Pentadecanoic acid $$ n-Pentadecylic acid $$

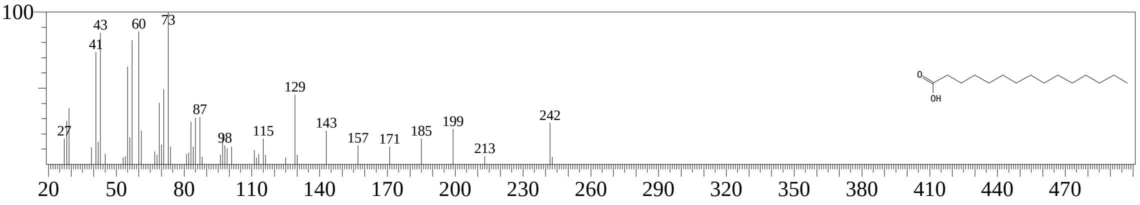

Hit#:2 Entry:19252 Library:NIST05s.LIB
SI:67 Formula:C14H28O2 CAS:544-63-8 MolWeight:228 RetIndex:1769
CompName:Tetradecanoic acid $$ Myristic acid $$ n-Tetradecanoic acid $$ n-Tetradecoic acid $$ Neo-Fat 14 $$ Univol U 316S $

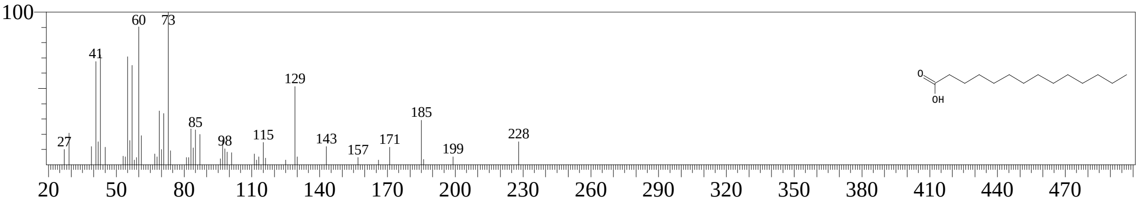

Hit#:3 Entry:17905 Library:NIST05s.LIB
SI:67 Formula:C13H26O2 CAS:638-53-9 MolWeight:214 RetIndex:1670
CompName:Tridecanoic acid $$ n-Tridecanoic acid $$ n-Tridecoic acid $$ Tridecylic acid $$

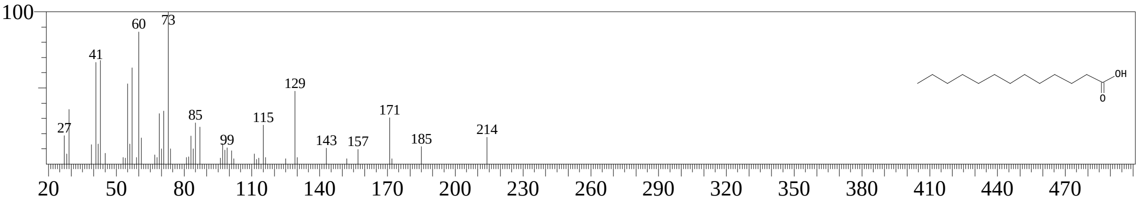

Hit#:4 Entry:19250 Library:NIST05s.LIB
SI:66 Formula:C14H28O2 CAS:544-63-8 MolWeight:228 RetIndex:1769
CompName:Tetradecanoic acid $$ Myristic acid $$ n-Tetradecanoic acid $$ n-Tetradecoic acid $$ Neo-Fat 14 $$ Univol U 316S $

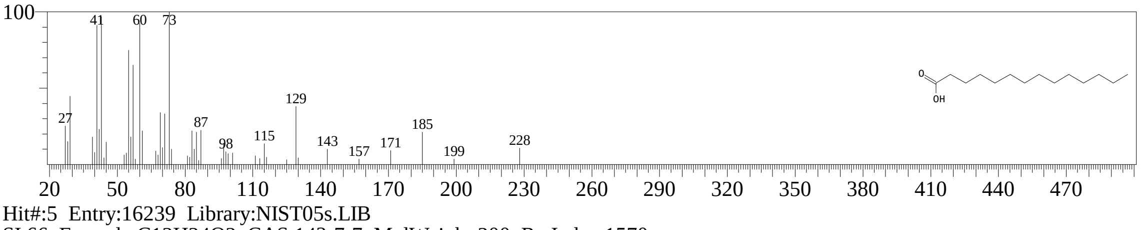

Hit#:5 Entry:16239 Library:NIST05s.LIB
SI:66 Formula:C12H24O2 CAS:143-7-7 MolWeight:200 RetIndex:1570
CompName:Dodecanoic acid $$ n-Dodecanoic acid $$ Neo-fat 12 $$ Aliphat no. 4 $$ Abl $$ Dodecylic acid $$ Lauric acid $$ Lau

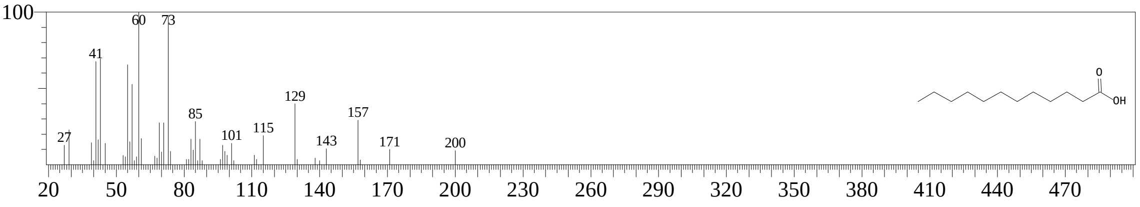

<< Target >>
Line#:18 R.Time:22.208(Scan#:2306) MassPeaks:282 BasePeak:74.00(6919)
RawMode:Averaged 22.200-22.217(2305-2307) BG Mode:Calc. from Peak

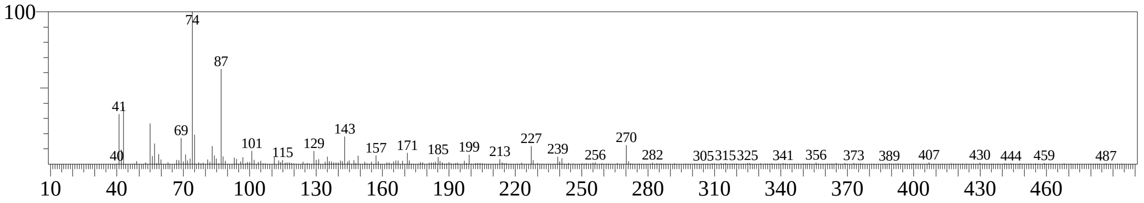

Hit#:1 Entry:83491 Library:NIST05.LIB
SI:91 Formula:C17H34O2 CAS:112-39-0 MolWeight:270 RetIndex:1878
CompName:Hexadecanoic acid, methyl ester $$ Palmitic acid, methyl ester $$ n-Hexadecanoic acid methyl ester $$ Metholene 221

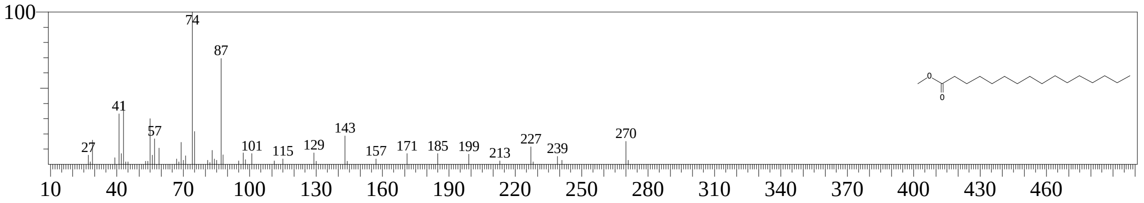

Hit#:2 Entry:22220 Library:NIST05s.LIB
SI:89 Formula:C17H34O2 CAS:112-39-0 MolWeight:270 RetIndex:1878
CompName:Hexadecanoic acid, methyl ester $$ Palmitic acid, methyl ester $$ n-Hexadecanoic acid methyl ester $$ Metholene 221

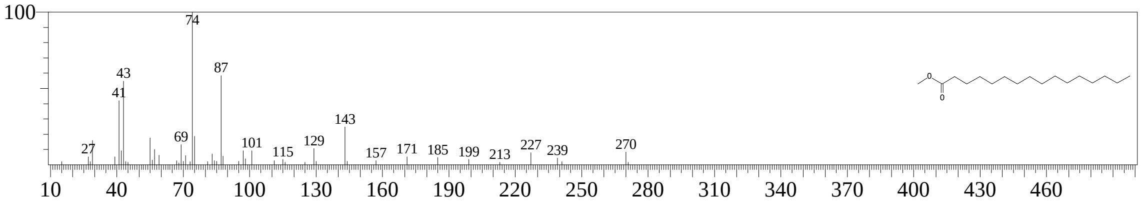

Hit#:3 Entry:22219 Library:NIST05s.LIB
SI:89 Formula:C17H34O2 CAS:112-39-0 MolWeight:270 RetIndex:1878
CompName:Hexadecanoic acid, methyl ester $$ Palmitic acid, methyl ester $$ n-Hexadecanoic acid methyl ester $$ Metholene 221

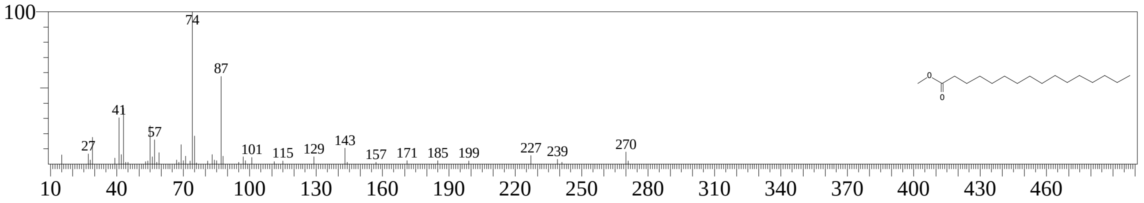

Hit#:4 Entry:83493 Library:NIST05.LIB
SI:88 Formula:C17H34O2 CAS:5129-60-2 MolWeight:270 RetIndex:1814
CompName:Pentadecanoic acid, 14-methyl-, methyl ester $$ Methyl 14-methylpentadecanoate # $$

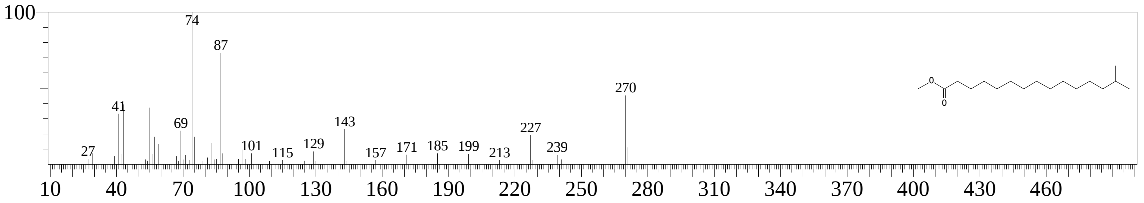

Hit#:5 Entry:22221 Library:NIST05s.LIB
SI:87 Formula:C17H34O2 CAS:112-39-0 MolWeight:270 RetIndex:1878
CompName:Hexadecanoic acid, methyl ester $$ Palmitic acid, methyl ester $$ n-Hexadecanoic acid methyl ester $$ Metholene 221

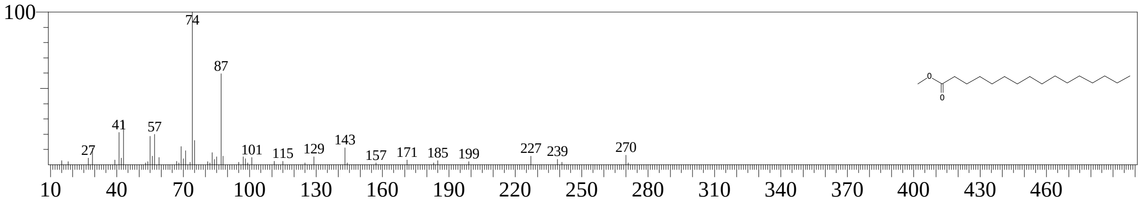

<< Target >>
Line#:19 R.Time:22.375(Scan#:2326) MassPeaks:247 BasePeak:41.05(2193)
RawMode:Averaged 22.367-22.383(2325-2327) BG Mode:Calc. from Peak

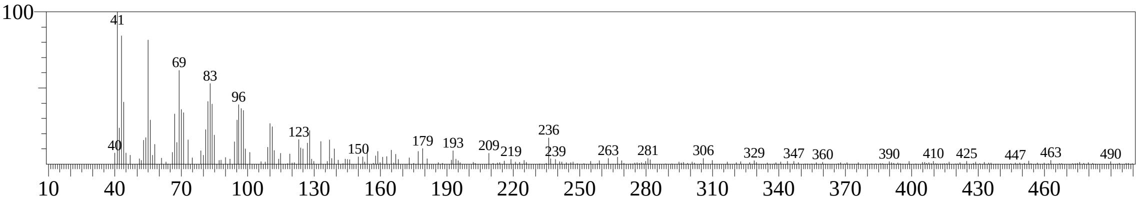

Hit#:1 Entry:65295 Library:NIST05.LIB
SI:82 Formula:C15H28O2 CAS:4727-18-8 MolWeight:240 RetIndex:2158
CompName:Cyclopentadecanone, 2-hydroxy- $$ 2-Hydroxycyclopentadecanone # $$

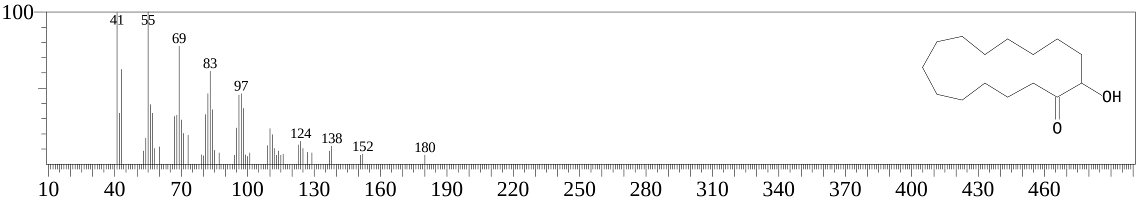

Hit#:2 Entry:103257 Library:NIST05.LIB
SI:81 Formula:C16H32O3S CAS:15224-88-1 MolWeight:304 RetIndex:0
CompName:1,2-Oxathiane, 6-dodecyl-, 2,2-dioxide $$ .delta.-Hexadecanesultone $$ Hexadecyl 1,4-sultone $$ Hexadecane-1-sulfo

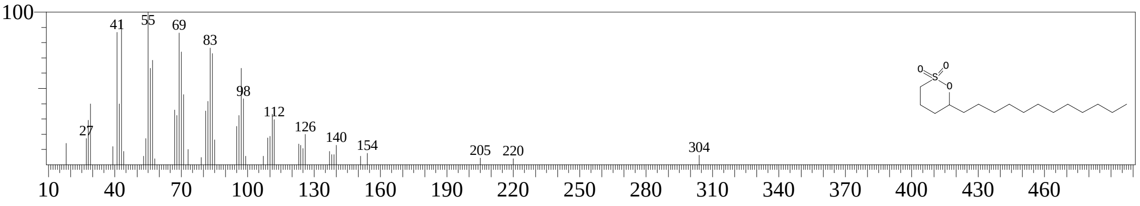

Hit#:3 Entry:20199 Library:NIST05s.LIB
SI:81 Formula:C15H28O2 CAS:106-2-5 MolWeight:240 RetIndex:2144
CompName:Oxacyclohexadecan-2-one $$ Cyclopentadecanolide $$ Exaltolide $$ Muskalactone $$ Muskolactone $$ Pentadecalac

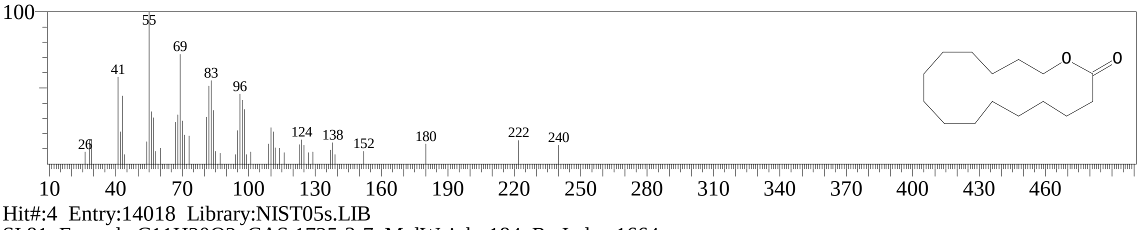

Hit#:4 Entry:14018 Library:NIST05s.LIB
SI:81 Formula:C11H20O2 CAS:1725-3-7 MolWeight:184 RetIndex:1664
CompName:Oxacyclododecan-2-one $$ 11-Hydroxyundecanoic acid, lactone $$

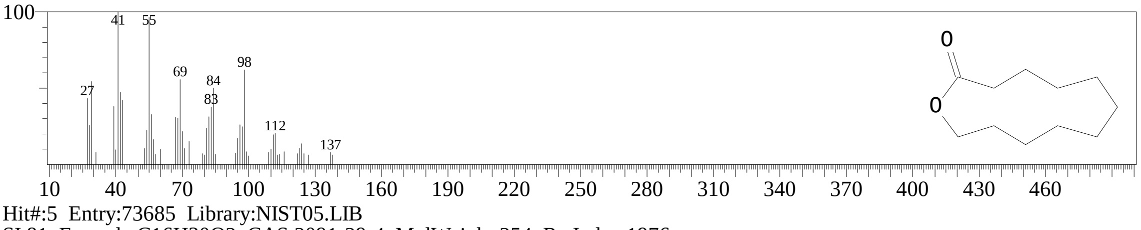

Hit#:5 Entry:73685 Library:NIST05.LIB
SI:81 Formula:C16H30O2 CAS:2091-29-4 MolWeight:254 RetIndex:1976
CompName:9-Hexadecenoic acid $$ (9E)-9-Hexadecenoic acid # $$

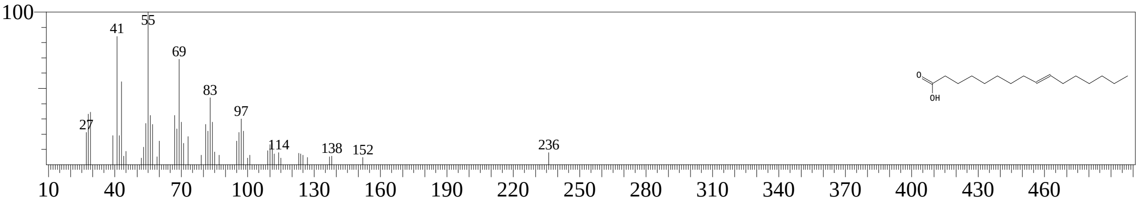

<< Target >>
Line#:20   R.Time:22.575(Scan#:2350) MassPeaks:294   BasePeak:43.00(13872)
RawMode:Averaged 22.567-22.583(2349-2351)   BG Mode:Calc. from Peak

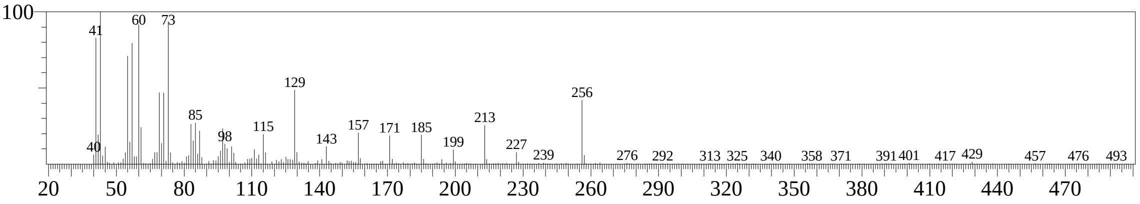

Hit#:1 Entry:21331 Library:NIST05s.LIB
SI:94 Formula:C16H32O2 CAS:57-10-3 MolWeight:256 RetIndex:1968
CompName:n-Hexadecanoic acid $$ Hexadecanoic acid $$ n-Hexadecoic acid $$ Palmitic acid $$ Pentadecanecarboxylic acid $$ 1

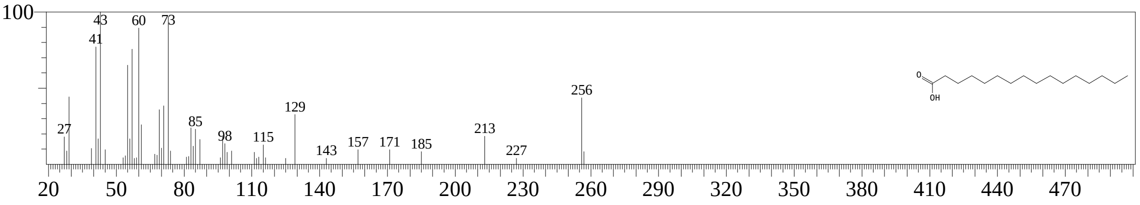

Hit#:2 Entry:19250 Library:NIST05s.LIB
SI:90 Formula:C14H28O2 CAS:544-63-8 MolWeight:228 RetIndex:1769
CompName:Tetradecanoic acid $$ Myristic acid $$ n-Tetradecanoic acid $$ n-Tetradecoic acid $$ Neo-Fat 14 $$ Univol U 316S $

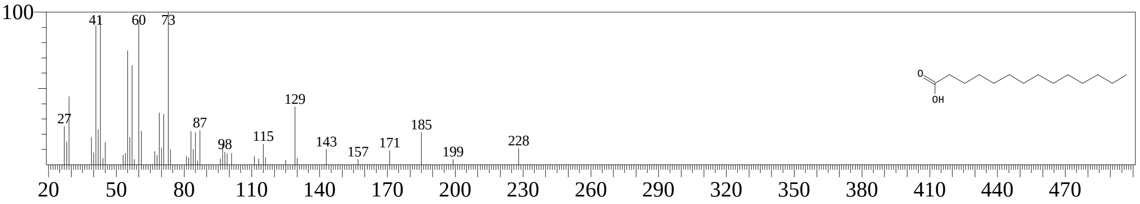

Hit#:3 Entry:161860 Library:NIST05.LIB
SI:90 Formula:C38H68O8 CAS:28474-90-0 MolWeight:652 RetIndex:4765
CompName:l-(+)-Ascorbic acid 2,6-dihexadecanoate

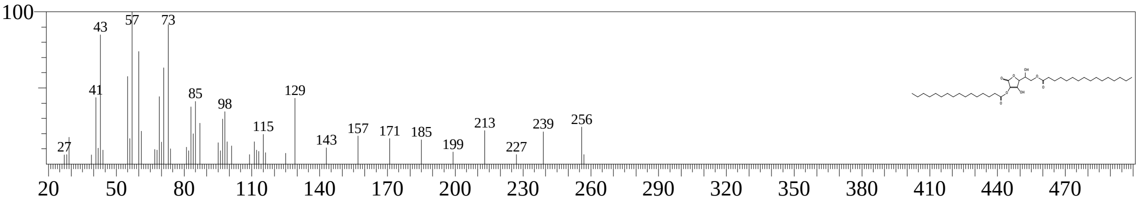

Hit#:4 Entry:20371 Library:NIST05s.LIB
SI:90 Formula:C15H30O2 CAS:1002-84-2 MolWeight:242 RetIndex:1869
CompName:Pentadecanoic acid $$ Pentadecylic acid $$ n-Pentadecanoic acid $$ n-Pentadecylic acid $$

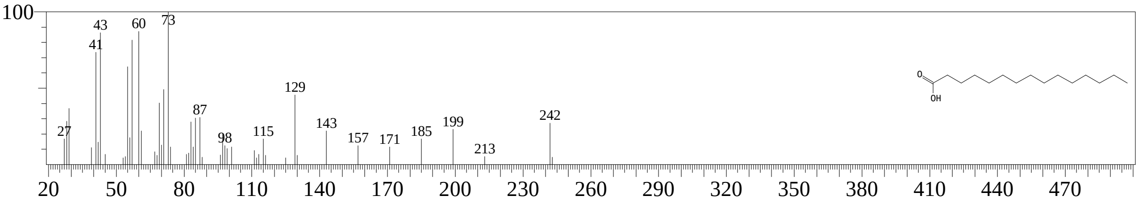

Hit#:5 Entry:22979 Library:NIST05s.LIB
SI:89 Formula:C18H36O2 CAS:57-11-4 MolWeight:284 RetIndex:2167
CompName:Octadecanoic acid $$ Stearic acid $$ n-Octadecanoic acid $$ Humko Industrene R $$ Hydrofol Acid 150 $$ Hystrene

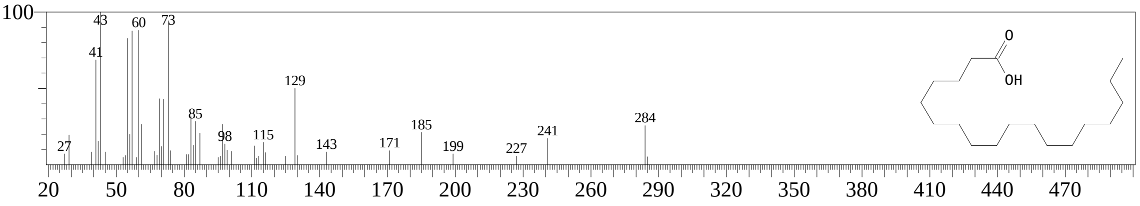

<< Target >>
Line#:21   R.Time:22.717(Scan#:2367) MassPeaks:303   BasePeak:148.95(9658)
RawMode:Averaged 22.708-22.725(2366-2368)   BG Mode:Calc. from Peak

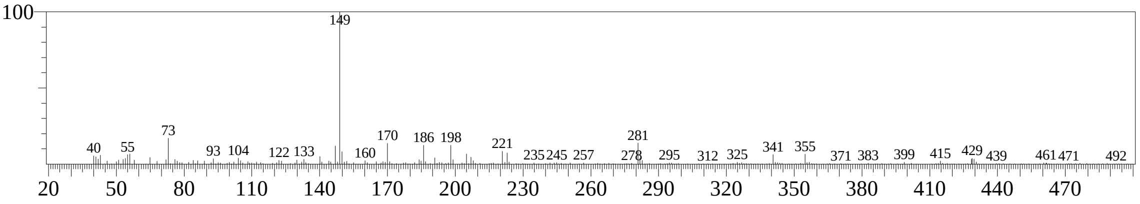

Hit#:1 Entry:25069 Library:NIST05s.LIB
SI:63 Formula:C20H30O4 CAS:84-78-6 MolWeight:334 RetIndex:2434
CompName:1,2-Benzenedicarboxylic acid, butyl octyl ester $$ Phthalic acid, butyl octyl ester $$ Butyl octyl phthalate $$ Octyl but

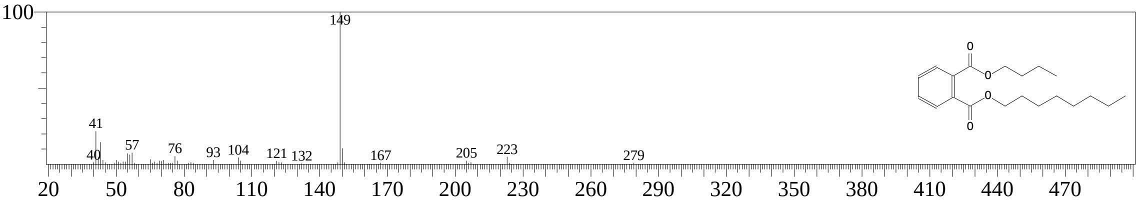

Hit#:2 Entry:88000 Library:NIST05.LIB
SI:63 Formula:C16H22O4 CAS:84-74-2 MolWeight:278 RetIndex:2037
CompName:Dibutyl phthalate $$ 1,2-Benzenedicarboxylic acid, dibutyl ester $$ Phthalic acid, dibutyl ester $$ n-Butyl phthalate $$

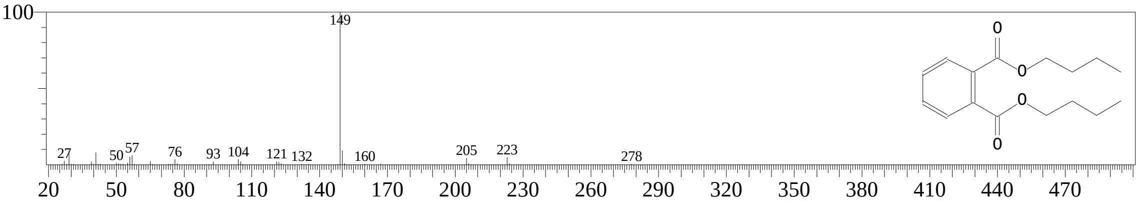

Hit#:3 Entry:88003 Library:NIST05.LIB
SI:63 Formula:C16H22O4 CAS:17851-53-5 MolWeight:278 RetIndex:1973
CompName:1,2-Benzenedicarboxylic acid, butyl 2-methylpropyl ester $$ Phthalic acid, butyl isobutyl ester $$ 1-Butyl 2-isobutyl pl

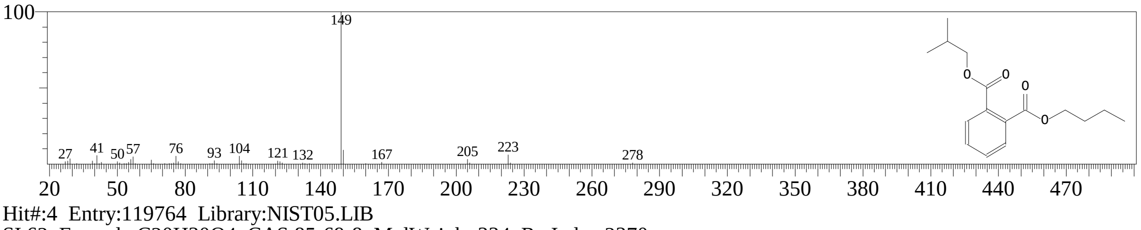

Hit#:4 Entry:119764 Library:NIST05.LIB
SI:62 Formula:C20H30O4 CAS:85-69-8 MolWeight:334 RetIndex:2370
CompName:1,2-Benzenedicarboxylic acid, butyl 2-ethylhexyl ester $$ Phthalic acid, butyl 2-ethylhexyl ester $$ Butyl 2-ethylhexyl

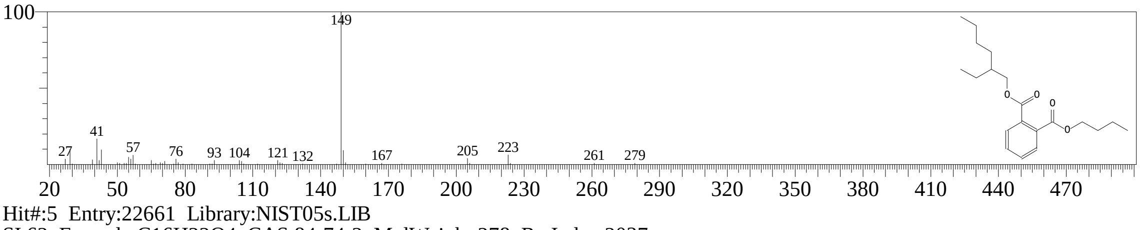

Hit#:5 Entry:22661 Library:NIST05s.LIB
SI:62 Formula:C16H22O4 CAS:84-74-2 MolWeight:278 RetIndex:2037
CompName:Dibutyl phthalate $$ 1,2-Benzenedicarboxylic acid, dibutyl ester $$ Phthalic acid, dibutyl ester $$ n-Butyl phthalate $$

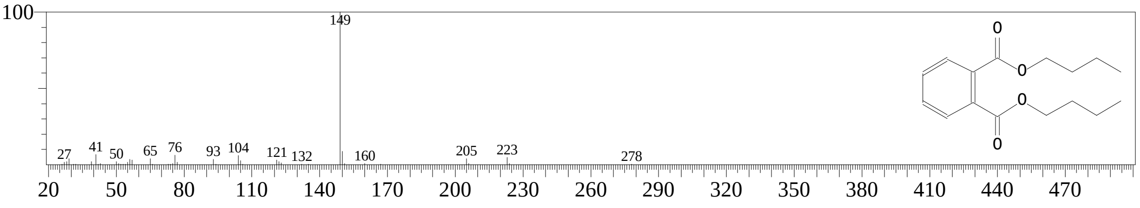

<< Target >>
Line#:22 R.Time:24.392(Scan#:2568) MassPeaks:307 BasePeak:55.00(5779)
RawMode:Averaged 24.383-24.400(2567-2569) BG Mode:Calc. from Peak

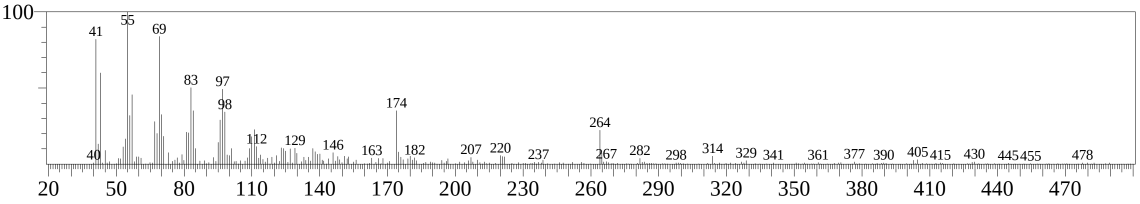

Hit#:1 Entry:90577 Library:NIST05.LIB
SI:89 Formula:C18H34O2 CAS:112-80-1 MolWeight:282 RetIndex:2175
CompName:Oleic Acid $$ 9-Octadecenoic acid (Z)- $$ .delta.(Sup9)-cis-Oleic acid $$ cis-.delta.(Sup9)-Octadecenoic acid $$ cis-C

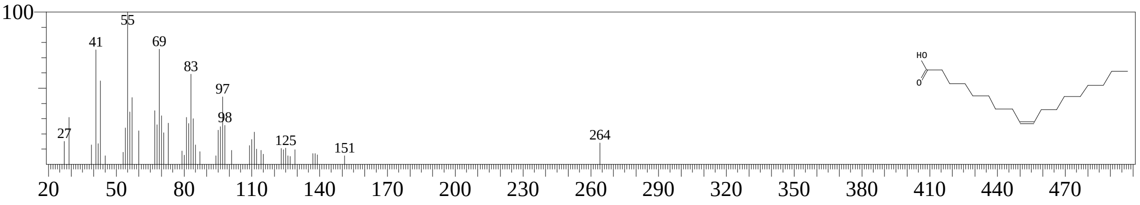

Hit#:2 Entry:22872 Library:NIST05s.LIB
SI:88 Formula:C18H34O2 CAS:112-79-8 MolWeight:282 RetIndex:2175
CompName:9-Octadecenoic acid, (E)- $$ trans-.delta.(sup 9)-Octadecenoic acid $$ trans-.delta.9-Octadecenoic acid $$ trans-Octad

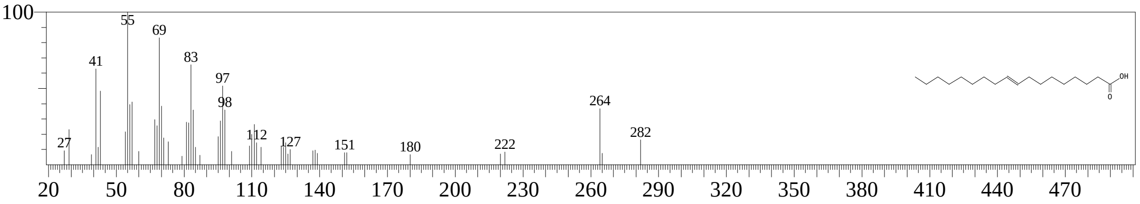

Hit#:3 Entry:90568 Library:NIST05.LIB
SI:88 Formula:C18H34O2 CAS:593-39-5 MolWeight:282 RetIndex:2175
CompName:6-Octadecenoic acid, (Z)- $$ (6Z)-6-Octadecenoic acid # $$

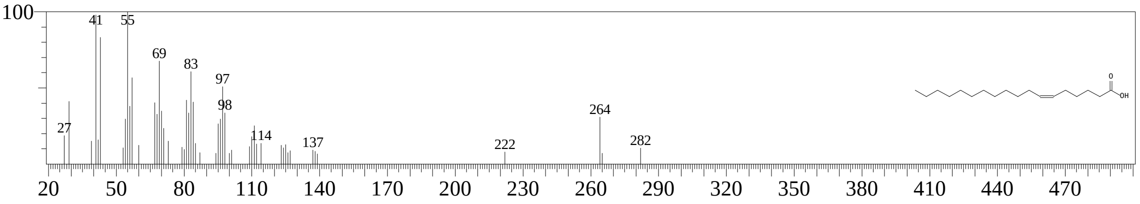

Hit#:4 Entry:90576 Library:NIST05.LIB
SI:87 Formula:C18H34O2 CAS:112-79-8 MolWeight:282 RetIndex:2175
CompName:9-Octadecenoic acid, (E)- $$ trans-.delta.(sup 9)-Octadecenoic acid $$ trans-.delta.9-Octadecenoic acid $$ trans-Octad

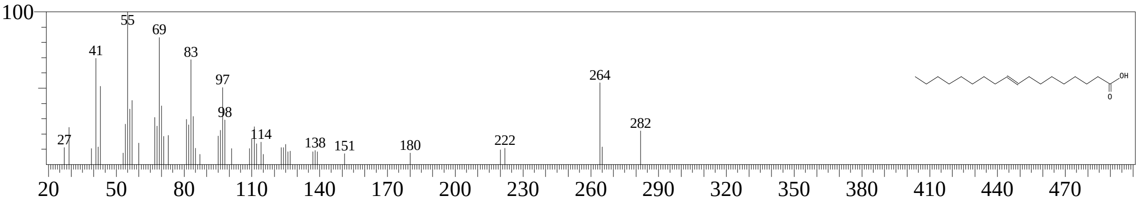

Hit#:5 Entry:90575 Library:NIST05.LIB
SI:87 Formula:C18H34O2 CAS:0-0-0 MolWeight:282 RetIndex:2175
CompName:Octadec-9-enoic acid

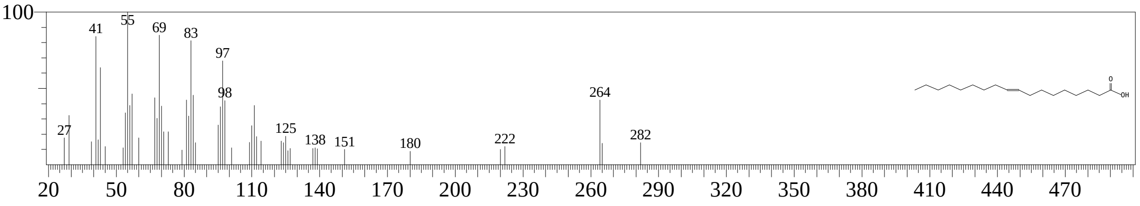

<< Target >>
Line#:23   R.Time:24.583(Scan#:2591) MassPeaks:240   BasePeak:43.00(1810)
RawMode:Averaged 24.575-24.592(2590-2592)   BG Mode:Calc. from Peak

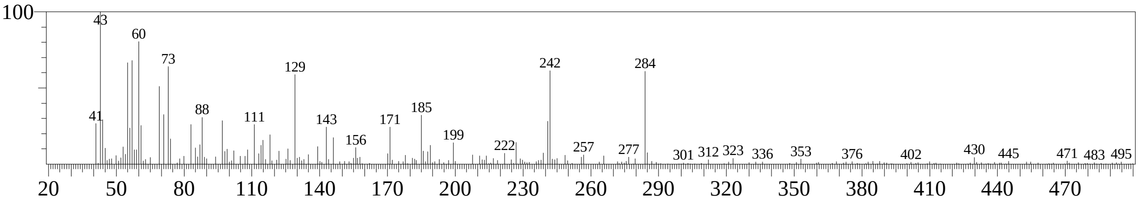

Hit#:1 Entry:22977 Library:NIST05s.LIB
SI:76 Formula:C18H36O2 CAS:57-11-4 MolWeight:284 RetIndex:2167
CompName:Octadecanoic acid $$ Stearic acid $$ n-Octadecanoic acid $$ Humko Industrene R $$ Hydrofol Acid 150 $$ Hystrene

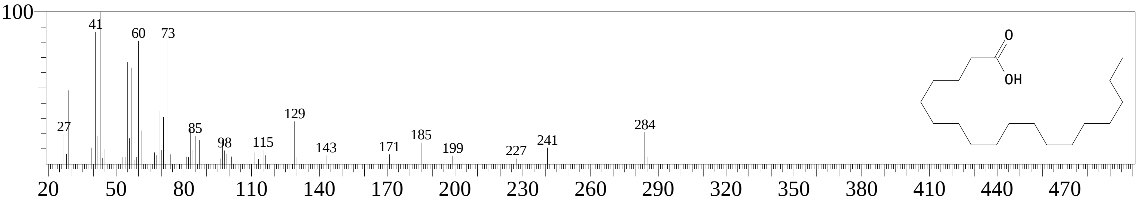

Hit#:2 Entry:22979 Library:NIST05s.LIB
SI:76 Formula:C18H36O2 CAS:57-11-4 MolWeight:284 RetIndex:2167
CompName:Octadecanoic acid $$ Stearic acid $$ n-Octadecanoic acid $$ Humko Industrene R $$ Hydrofol Acid 150 $$ Hystrene

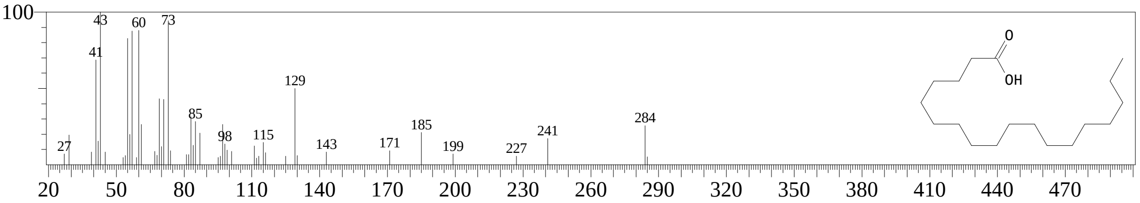

Hit#:3 Entry:136237 Library:NIST05.LIB
SI:75 Formula:C22H44O4 CAS:106-11-6 MolWeight:372 RetIndex:2694
CompName:Octadecanoic acid, 2-(2-hydroxyethoxy)ethyl ester $$ Aqua Cera $$ Atlas G 2146 $$ Cerasynt $$ Cerasynt Special $$

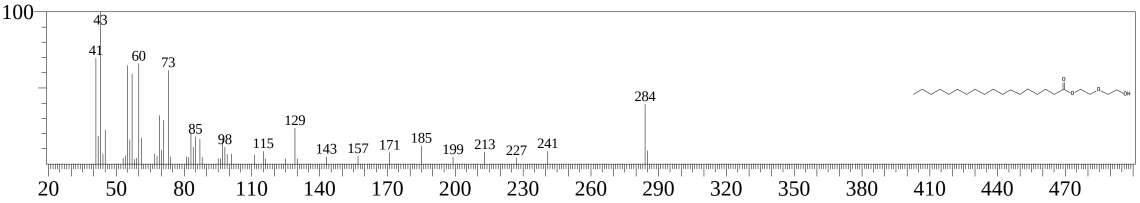

Hit#:4 Entry:22978 Library:NIST05s.LIB
SI:75 Formula:C18H36O2 CAS:57-11-4 MolWeight:284 RetIndex:2167
CompName:Octadecanoic acid $$ Stearic acid $$ n-Octadecanoic acid $$ Humko Industrene R $$ Hydrofol Acid 150 $$ Hystrene

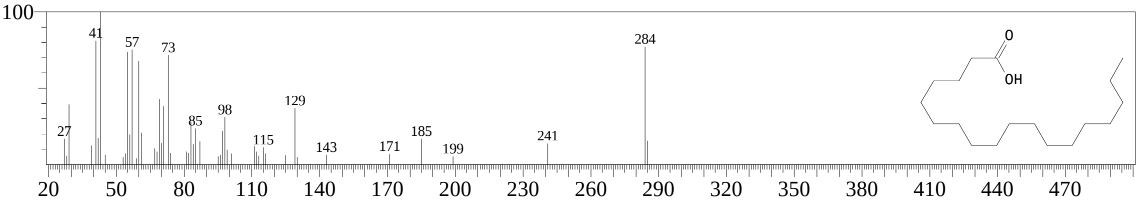

Hit#:5 Entry:91895 Library:NIST05.LIB
SI:74 Formula:C18H36O2 CAS:57-11-4 MolWeight:284 RetIndex:2167
CompName:Octadecanoic acid $$ Stearic acid $$ n-Octadecanoic acid $$ Humko Industrene R $$ Hydrofol Acid 150 $$ Hystrene

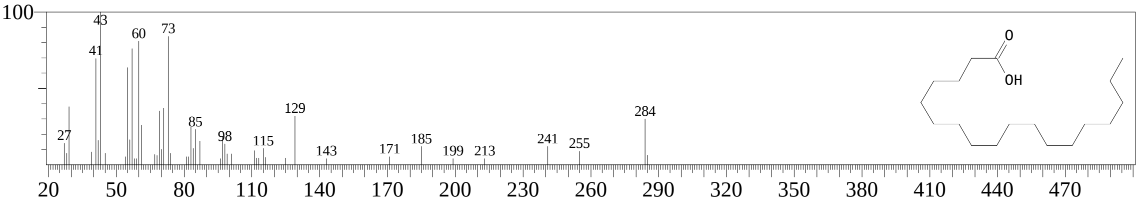

Supplement: Supplemental Information 4 [file peerj-07-6442-s004.pdf]
