# Supplementary material for: Comparative study on the composition of four different varieties of garlic
Source: PeerJ. 2019 Feb 21;7:e6442. doi: 10.7717/peerj.6442 (PMC6387757; doi:10.7717/peerj.6442)

Sample Information

Analyzed by : Admin
Analyzed : 2018-5-7 19:23:31
Sample Type : Unknown
Level # : 1
Sample Name : 2018-5-7-2-3
Sample ID :
IS Amount : [1]=1.000
Sample Amount : 1.000
Dilution Factor : 1.000
Vial # : 1
Injection Volume : 1.000
Data File : E:\陈存\GC-MS硫化物\2018-5-7-2-3.qgd
Org Data File : E:\刘春宏\2018-5-7-2-3.qgd
Method File : E:\刘春宏\2018-5-7-1.qgm
Org Method File : E:\刘春宏\2018-5-7-1.qgm
Report File :
Tuning File : C:\GCMSsolution\System\Tune1\2017-3-21.qgt
Modified by : Admin
Modified : 2018-6-15 12:58:12

Chromatogram 2018-5-7-2-3 E:\陈存\GC-MS硫化物\2018-5-7-2-3.qgd

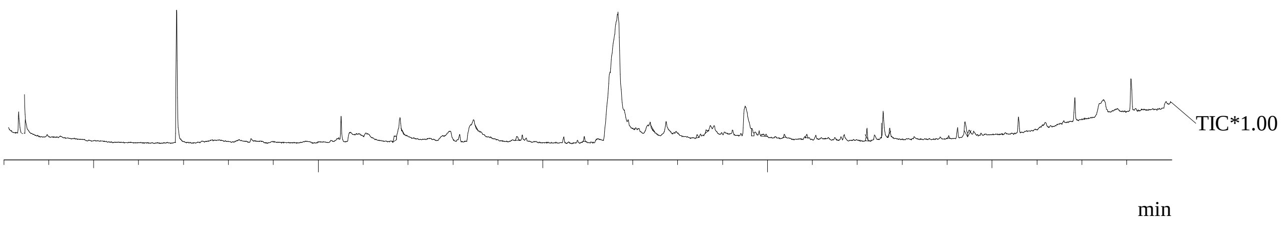

Peak Report TIC

Peak#	R.Time	I.Time	F.Time	Area	Area%	Height	Ieight%	A/H	Mark	Name
1	3.332	3.300	3.408	293061	1.38	156213	4.53	1.87		
2	6.846	6.817	6.958	1927850	9.07	961572	27.89	2.00		
3	11.692	11.658	11.742	138679	0.65	38750	1.12	3.57		
4	11.818	11.742	11.917	617689	2.91	143436	4.16	4.30	V	
5	13.456	13.300	13.567	1037745	4.88	113119	3.28	9.17		
6	14.421	14.383	14.475	80529	0.38	28785	0.83	2.79		
7	15.922	15.892	15.958	61031	0.29	36588	1.06	1.66		
8	16.674	16.342	16.808	12980364	61.07	889653	25.80	14.59		
9	16.817	16.808	16.875	417690	1.97	157784	4.58	2.64	V	
10	17.386	17.275	17.475	350150	1.65	67485	1.96	5.18		
11	18.434	18.400	18.467	51220	0.24	24815	0.72	2.06		
12	18.633	18.608	18.692	47580	0.22	21376	0.62	2.22		
13	19.502	19.442	19.642	1529451	7.20	214796	6.23	7.12		
14	19.667	19.642	19.700	127750	0.60	46599	1.35	2.74	V	
15	19.975	19.850	19.992	92104	0.43	15231	0.44	6.04	V	
16	20.376	20.350	20.408	35317	0.17	22873	0.66	1.54		
17	20.874	20.850	20.908	40258	0.19	24141	0.70	1.66		
18	22.215	22.175	22.250	169537	0.80	93503	2.71	1.81		
19	22.383	22.358	22.542	47486	0.22	29992	0.87	1.58		
20	22.577	22.542	22.667	480158	2.26	193874	5.62	2.47	V	
21	24.033	24.008	24.067	26757	0.13	20344	0.59	1.31		
22	24.399	24.267	24.458	400840	1.89	112402	3.26	3.56		
23	24.592	24.458	24.733	301788	1.42	34584	1.00	8.72	V	
				21255034	100.00	3447915	100.00			

Library

<< Target >>
Line#:1   R.Time:3.333(Scan#:41) MassPeaks:269   BasePeak:43.00(101176)
RawMode:Averaged 3.325-3.342(40-42)   BG Mode:Calc. from Peak

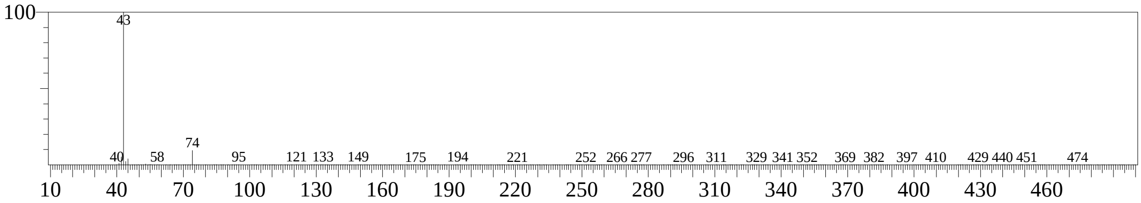

Hit#:1 Entry:415 Library:NIST05s.LIB
SI:98 Formula:C3H6O2 CAS:116-9-6 MolWeight:74 RetIndex:698
CompName:2-Propanone, 1-hydroxy- $$ Acetol $$ CH3C(O)CH2OH $$ Hydroxyacetone $$ Acetone alcohol $$ Acetylcarbinol $$

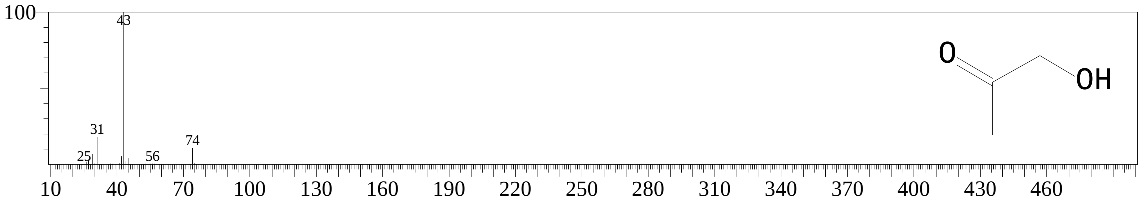

Hit#:2 Entry:368 Library:NIST05.LIB
SI:97 Formula:C3H6O2 CAS:116-9-6 MolWeight:74 RetIndex:698
CompName:2-Propanone, 1-hydroxy- $$ Acetol $$ CH3C(O)CH2OH $$ Hydroxyacetone $$ Acetone alcohol $$ Acetylcarbinol $$

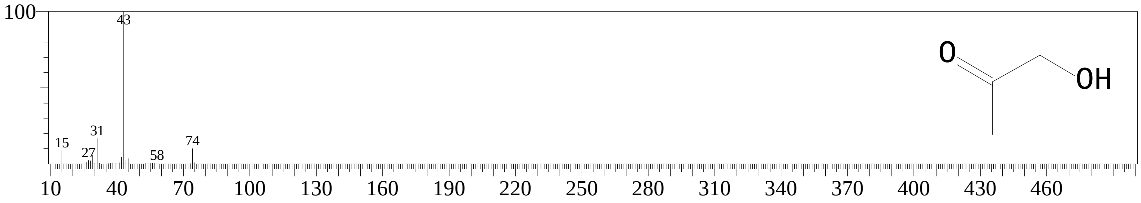

Hit#:3 Entry:414 Library:NIST05s.LIB
SI:96 Formula:C3H6O2 CAS:116-9-6 MolWeight:74 RetIndex:698
CompName:2-Propanone, 1-hydroxy- $$ Acetol $$ CH3C(O)CH2OH $$ Hydroxyacetone $$ Acetone alcohol $$ Acetylcarbinol $$

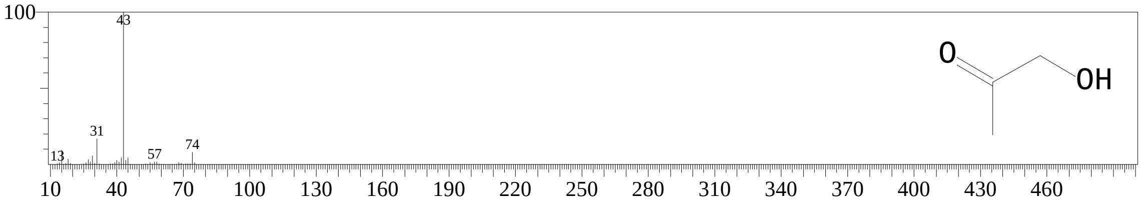

Hit#:4 Entry:369 Library:NIST05.LIB
SI:93 Formula:C3H6O2 CAS:79-20-9 MolWeight:74 RetIndex:487
CompName:Acetic acid, methyl ester $$ Methyl acetate $$ Devoton $$ Tereton $$ CH3COOCH3 $$ Methyl ethanoate $$ Acetate c

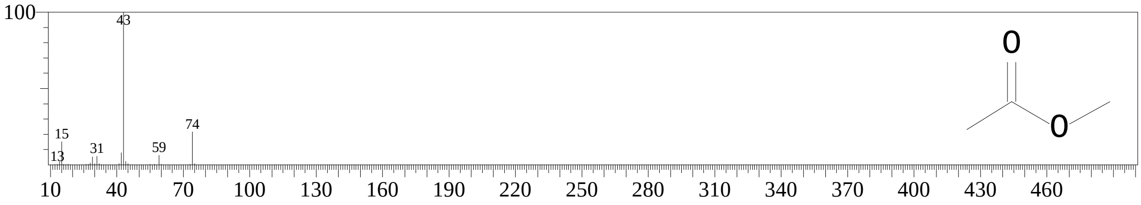

Hit#:5 Entry:1953 Library:NIST05s.LIB
SI:92 Formula:C4H6O3 CAS:108-24-7 MolWeight:102 RetIndex:722
CompName:Acetic anhydride $$ Acetic acid, anhydride $$ Acetic oxide $$ Acetyl anhydride $$ Acetyl ether $$ Acetyl oxide $$ Et

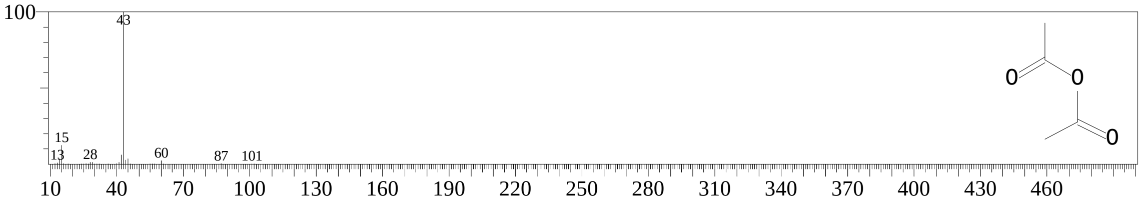

<< Target >>
Line#:2   R.Time:6.842(Scan#:462) MassPeaks:286   BasePeak:132.95(255875)
RawMode:Averaged 6.833-6.850(461-463)   BG Mode:Calc. from Peak

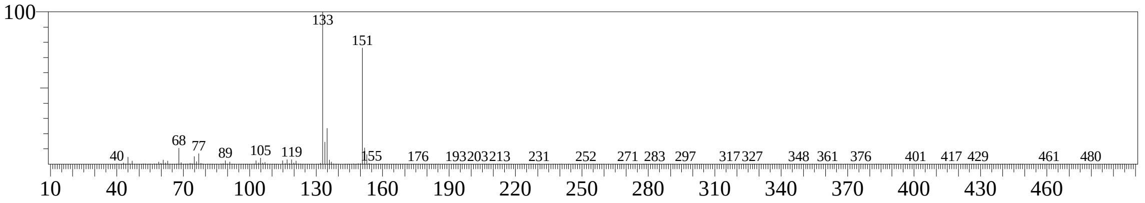

Hit#:1 Entry:15209 Library:NIST05.LIB
SI:85 Formula:C8H9NO2 CAS:0-0-0 MolWeight:151 RetIndex:1301
CompName:Oxime-, methoxy-phenyl-_

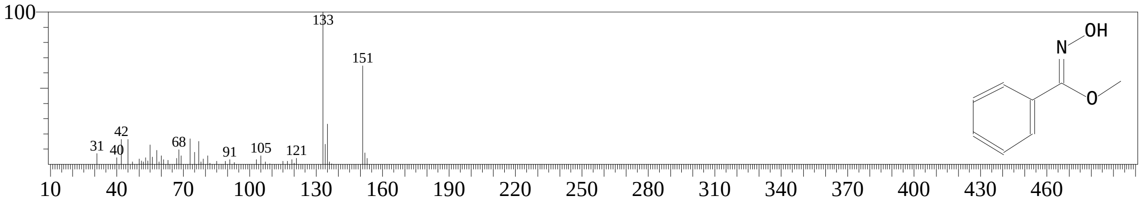

Hit#:2 Entry:15206 Library:NIST05.LIB
SI:79 Formula:C8H9NO2 CAS:2941-78-8 MolWeight:151 RetIndex:1575
CompName:2-Amino-5-methylbenzoic acid $$ 5-Methylanthranilic acid $$ Benzoic acid, 2-amino-5-methyl- $$ m-Toluic acid, 6-a

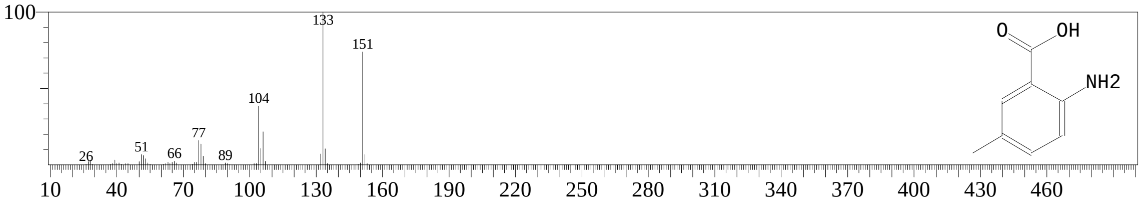

Hit#:3 Entry:51823 Library:NIST05.LIB
SI:79 Formula:C14H18O2 CAS:0-0-0 MolWeight:218 RetIndex:1714
CompName:4-Ethylbenzoic acid, cyclopentyl ester

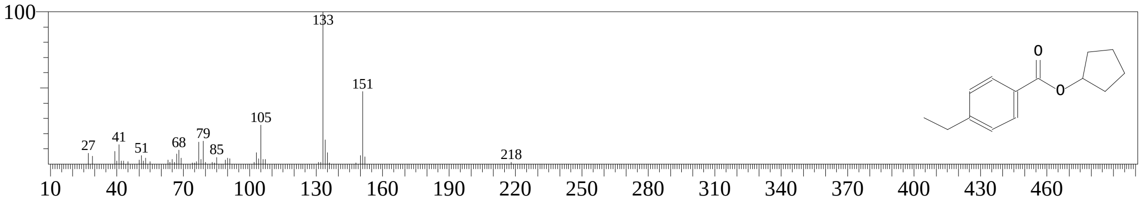

Hit#:4 Entry:61585 Library:NIST05.LIB
SI:78 Formula:C15H22O2 CAS:0-0-0 MolWeight:234 RetIndex:1770
CompName:4-Ethylbenzoic acid, hexyl ester

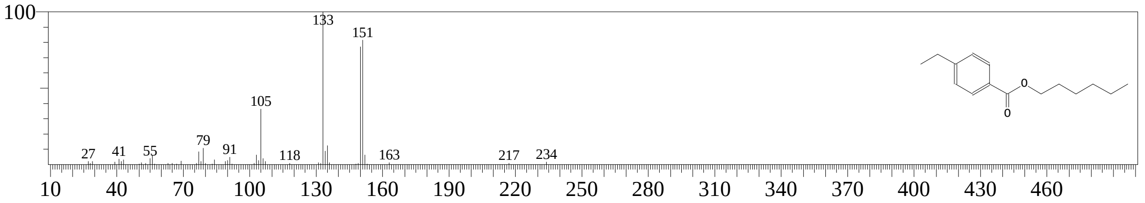

Hit#:5 Entry:15207 Library:NIST05.LIB
SI:76 Formula:C8H9NO2 CAS:2305-36-4 MolWeight:151 RetIndex:1575
CompName:Benzoic acid, 2-amino-4-methyl- $$ p-Toluic acid, 2-amino- $$ 2-Amino-4-methylbenzoic acid # $$

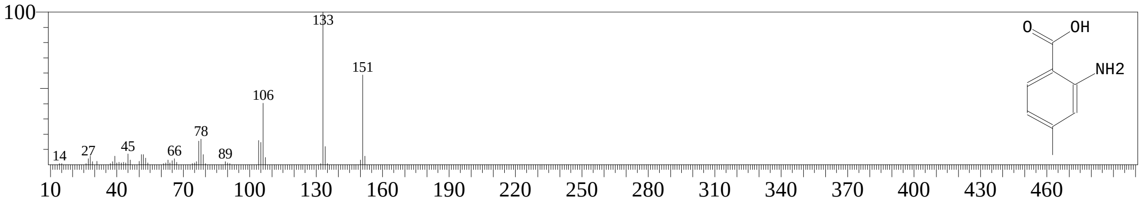

<< Target >>
Line#:3   R.Time:11.692(Scan#:1044) MassPeaks:232   BasePeak:43.00(8837)
RawMode:Averaged 11.683-11.700(1043-1045)   BG Mode:Calc. from Peak

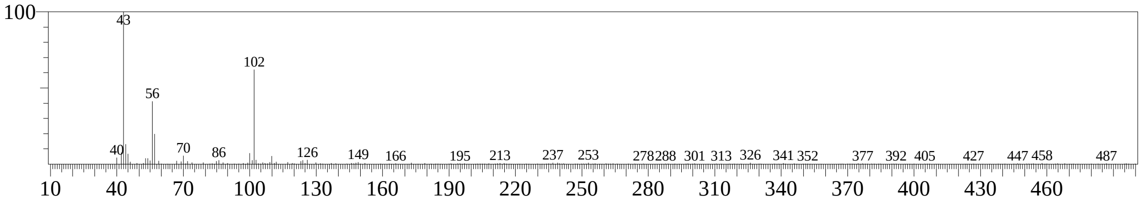

Hit#:1 Entry:2065 Library:NIST05.LIB
SI:77 Formula:C4H7NO2 CAS:1192-20-7 MolWeight:101 RetIndex:1030
CompName:Alpha-amino-gamma-butyrolactone $$ 3-Aminodihydro-2(3H)-furanone # $$

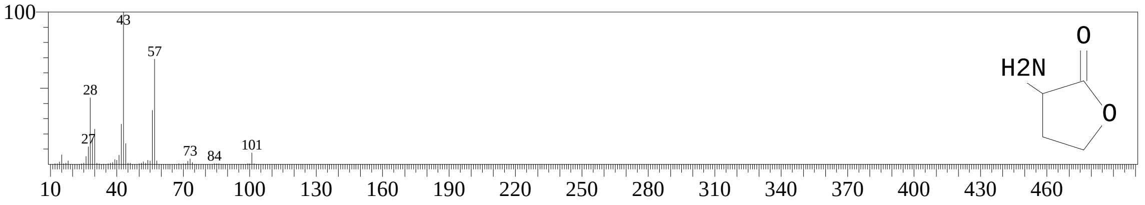

Hit#:2 Entry:7443 Library:NIST05s.LIB
SI:76 Formula:C7H12O3 CAS:609-14-3 MolWeight:144 RetIndex:956
CompName:Butanoic acid, 2-methyl-3-oxo-, ethyl ester $$ Acetoacetic acid, 2-methyl-, ethyl ester $$ .alpha.-Methylacetoacetic est

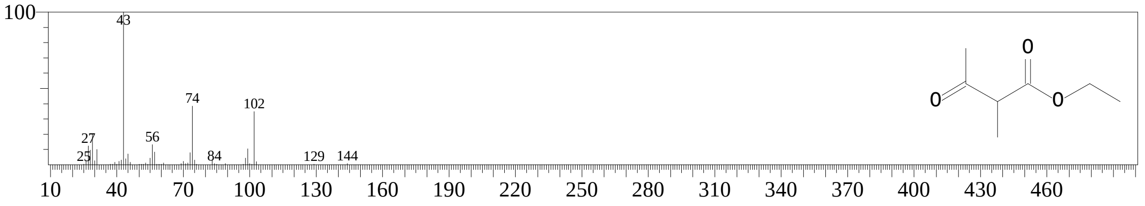

Hit#:3 Entry:7446 Library:NIST05s.LIB
SI:76 Formula:C7H12O3 CAS:609-14-3 MolWeight:144 RetIndex:956
CompName:Butanoic acid, 2-methyl-3-oxo-, ethyl ester $$ Acetoacetic acid, 2-methyl-, ethyl ester $$ .alpha.-Methylacetoacetic est

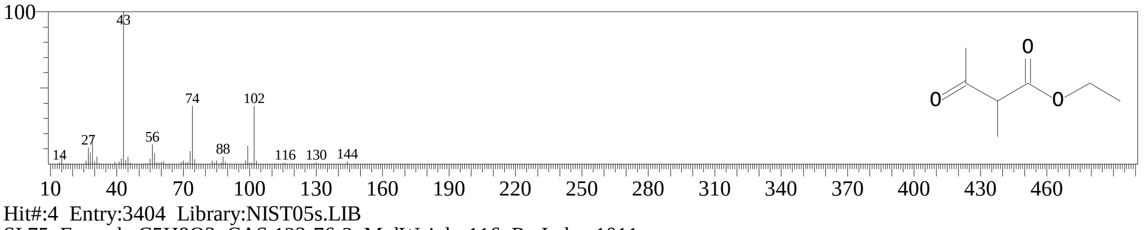

Hit#:4 Entry:3404 Library:NIST05s.LIB
SI:75 Formula:C5H8O3 CAS:123-76-2 MolWeight:116 RetIndex:1011
CompName:Pentanoic acid, 4-oxo- $$ Levulinic acid $$ .beta.-Acetylpropionic acid $$ .gamma.-Ketovaleric acid $$ Laevulinic aci

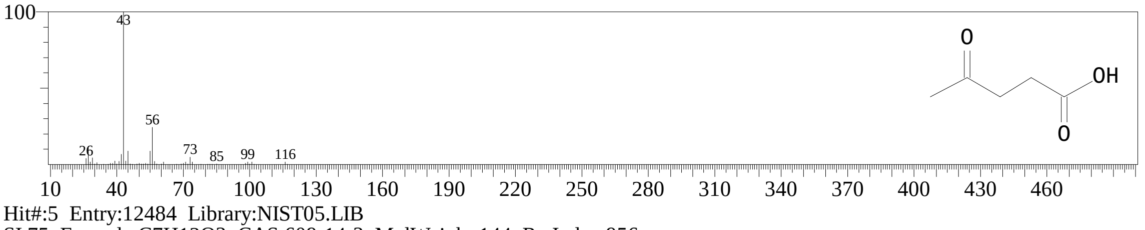

Hit#:5 Entry:12484 Library:NIST05.LIB
SI:75 Formula:C7H12O3 CAS:609-14-3 MolWeight:144 RetIndex:956
CompName:Butanoic acid, 2-methyl-3-oxo-, ethyl ester $$ Acetoacetic acid, 2-methyl-, ethyl ester $$ .alpha.-Methylacetoacetic est

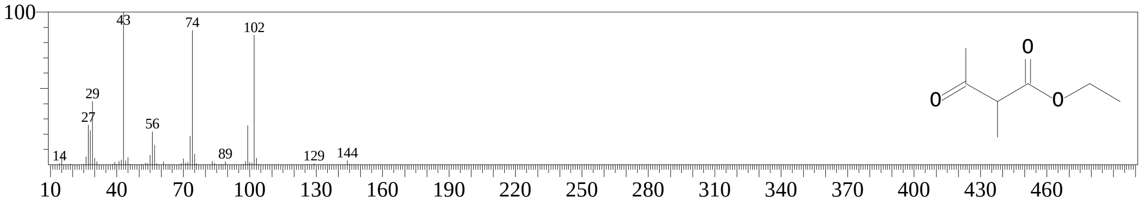

<< Target >>
Line#:4   R.Time:11.817(Scan#:1059) MassPeaks:287   BasePeak:43.00(19013)
RawMode:Averaged 11.808-11.825(1058-1060)   BG Mode:Calc. from Peak

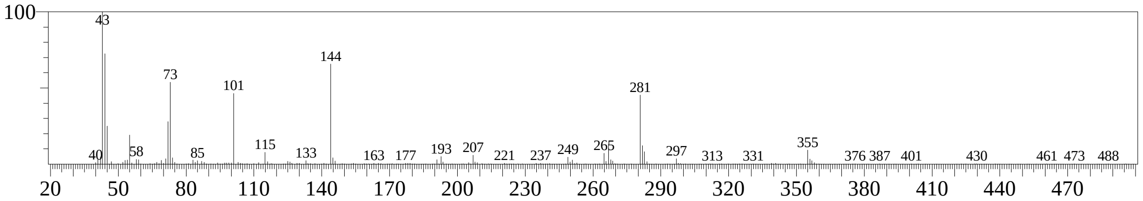

Hit#:1 Entry:7403 Library:NIST05s.LIB
SI:81 Formula:C6H8O4 CAS:28564-83-2 MolWeight:144 RetIndex:1269
CompName:4H-Pyran-4-one, 2,3-dihydro-3,5-dihydroxy-6-methyl- $$ 3,5-Dihydroxy-6-methyl-2,3-dihydro-4H-pyran-4-one # $$

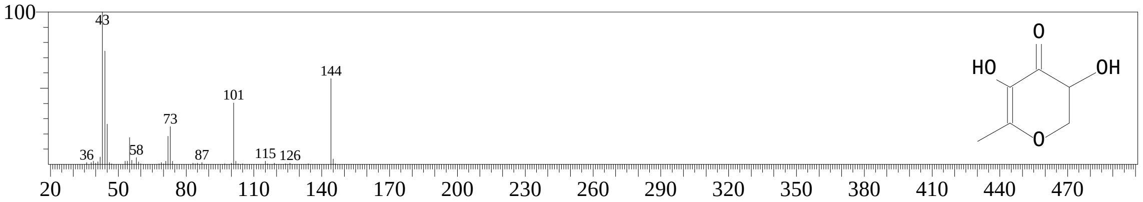

Hit#:2 Entry:12367 Library:NIST05.LIB
SI:77 Formula:C6H8O4 CAS:28564-83-2 MolWeight:144 RetIndex:1269
CompName:4H-Pyran-4-one, 2,3-dihydro-3,5-dihydroxy-6-methyl- $$ 3,5-Dihydroxy-6-methyl-2,3-dihydro-4H-pyran-4-one # $$

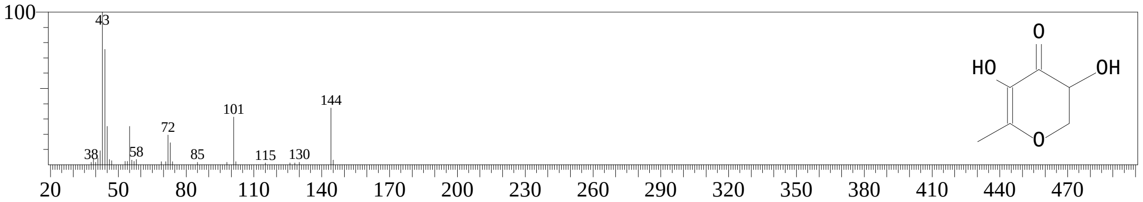

Hit#:3 Entry:4455 Library:NIST05.LIB
SI:71 Formula:C6H12O2 CAS:3299-32-9 MolWeight:116 RetIndex:761
CompName:1,3-Dioxolane, 2,4,5-trimethyl- $$ 2,4,5-Trimethyl-1,3-dioxolane # $$

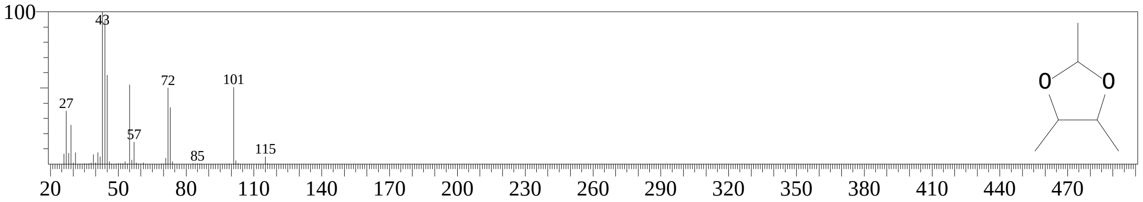

Hit#:4 Entry:12371 Library:NIST05.LIB
SI:70 Formula:C6H8O4 CAS:10230-62-3 MolWeight:144 RetIndex:1173
CompName:2,4-Dihydroxy-2,5-dimethyl-3(2H)-furan-3-one $$ 2,4-Dihydroxy-2,5-dimethyl-3(2H)-furanone # $$

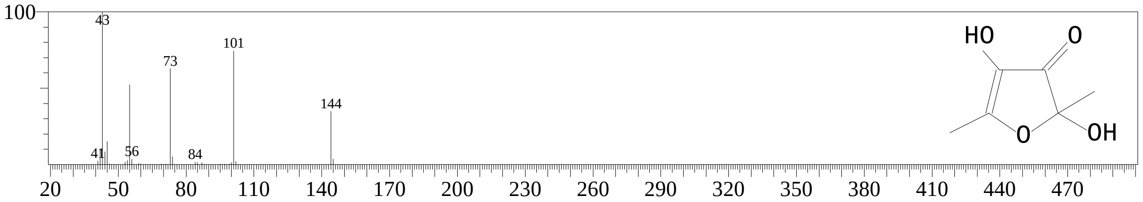

Hit#:5 Entry:12589 Library:NIST05.LIB
SI:67 Formula:C8H16O2 CAS:0-0-0 MolWeight:144 RetIndex:1156
CompName:2-Propyl-tetrahydropyran-3-ol

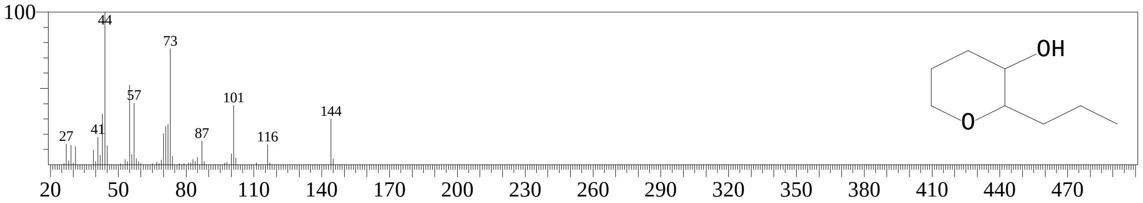

<< Target >>
Line#:5   R.Time:13.458(Scan#:1256) MassPeaks:291   BasePeak:97.00(17719)
RawMode:Averaged 13.450-13.467(1255-1257)   BG Mode:Calc. from Peak

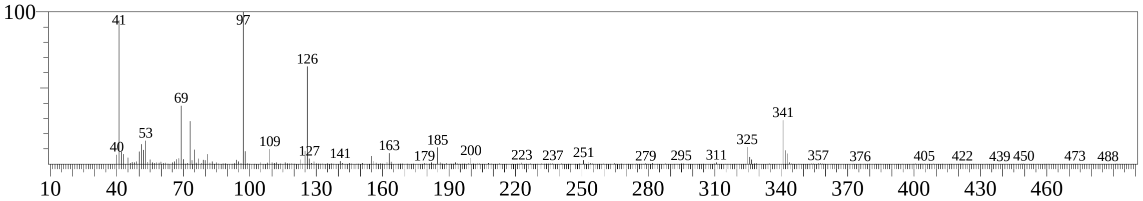

Hit#:1 Entry:6237 Library:NIST05.LIB
SI:79 Formula:C6H6O3 CAS:67-47-0 MolWeight:126 RetIndex:1163
CompName:2-Furancarboxaldehyde, 5-(hydroxymethyl)- $$ 2-Furaldehyde, 5-(hydroxymethyl)- $$ 5-Hydrxoymethylfurfural $$ Hy

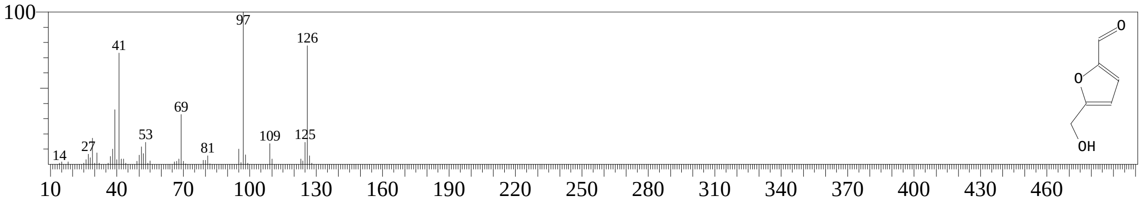

Hit#:2 Entry:4517 Library:NIST05s.LIB
SI:78 Formula:C6H6O3 CAS:67-47-0 MolWeight:126 RetIndex:1163
CompName:2-Furancarboxaldehyde, 5-(hydroxymethyl)- $$ 2-Furaldehyde, 5-(hydroxymethyl)- $$ 5-Hydrxoymethylfurfural $$ Hy

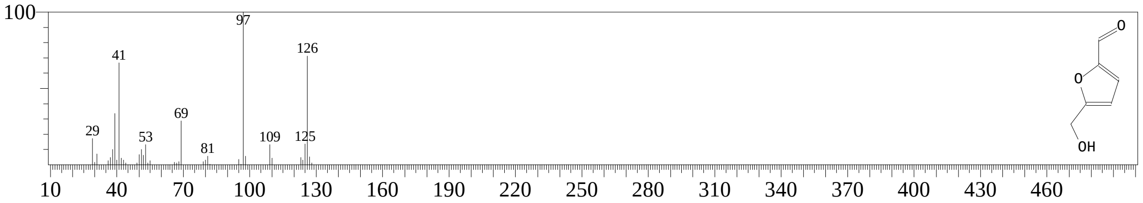

Hit#:3 Entry:6371 Library:NIST05.LIB
SI:72 Formula:C8H14O CAS:17325-90-5 MolWeight:126 RetIndex:915
CompName:4-Hexen-3-one, 4,5-dimethyl- $$ 4,5-Dimethyl-4-hexen-3-one # $$

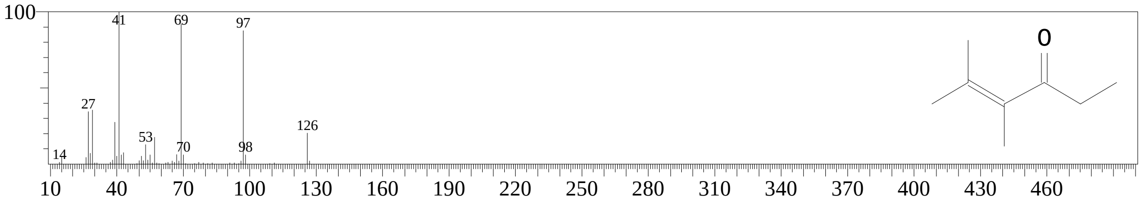

Hit#:4 Entry:6372 Library:NIST05.LIB
SI:70 Formula:C8H14O CAS:22319-31-9 MolWeight:126 RetIndex:938
CompName:4-Hepten-3-one, 4-methyl- $$ (4E)-4-Methyl-4-hepten-3-one # $$

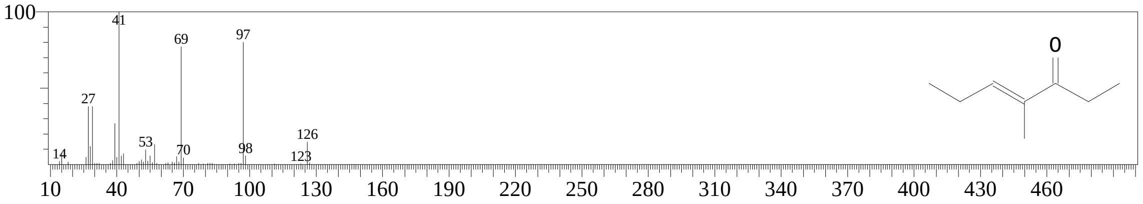

Hit#:5 Entry:4533 Library:NIST05s.LIB
SI:69 Formula:C6H10N2O CAS:3201-20-5 MolWeight:126 RetIndex:1007
CompName:3H-Pyrazol-3-one, 2,4-dihydro-4,4,5-trimethyl- $$ 2-Pyrazolin-5-one, 3,4,4-trimethyl- $$ 3,4,4-Trimethyl-5-pyrazolone

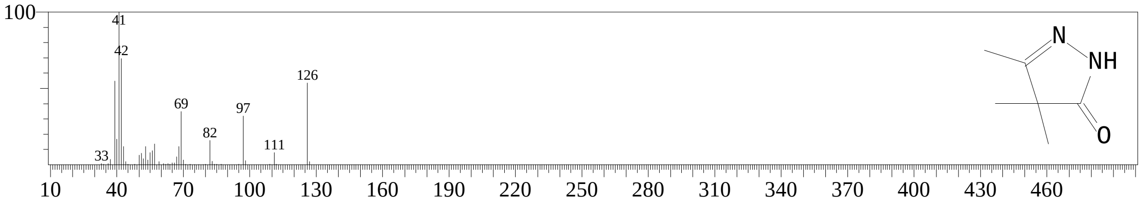

<< Target >>
Line#:6   R.Time:14.425(Scan#:1372) MassPeaks:250   BasePeak:41.00(6254)
RawMode:Averaged 14.417-14.433(1371-1373)   BG Mode:Calc. from Peak

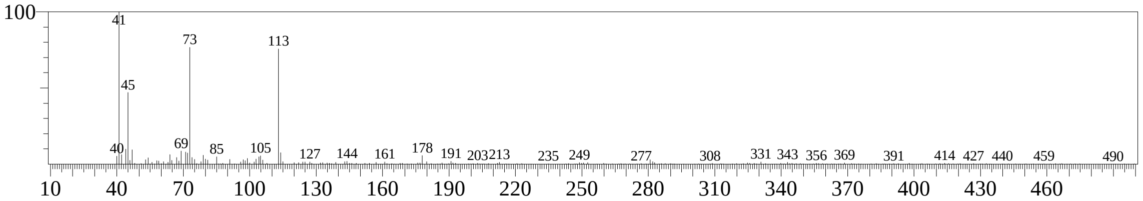

Hit#:1 Entry:28371 Library:NIST05.LIB
SI:89 Formula:C6H10S3 CAS:2050-87-5 MolWeight:178 RetIndex:1350
CompName:Trisulfide, di-2-propenyl $$ Allyl trisulfide $$ Diallyl trisulfide $$ 1,3-Diallyltrisulfane # $$

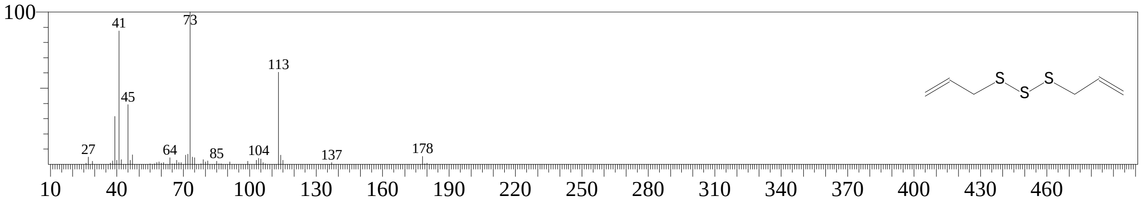

Hit#:2 Entry:12960 Library:NIST05s.LIB
SI:88 Formula:C6H10S3 CAS:2050-87-5 MolWeight:178 RetIndex:1350
CompName:Trisulfide, di-2-propenyl $$ Allyl trisulfide $$ Diallyl trisulfide $$ 1,3-Diallyltrisulfane # $$

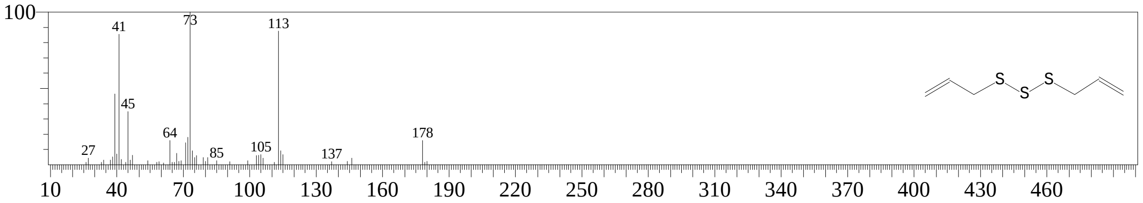

Hit#:3 Entry:3712 Library:NIST05.LIB
SI:81 Formula:C5H7NS CAS:105643-80-9 MolWeight:113 RetIndex:1004
CompName:2-Propenylthioacetonitrile $$ 2-Propenylthioacetonitrile $$ (Allylsulfanyl)acetonitrile # $$

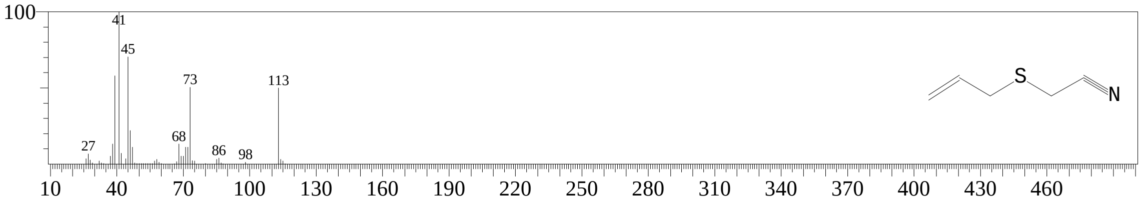

Hit#:4 Entry:13170 Library:NIST05.LIB
SI:78 Formula:C6H10S2 CAS:2179-57-9 MolWeight:146 RetIndex:1099
CompName:Diallyl disulphide $$ Allyl disulfide $$ Diallyl disulfide $$ Disulfide, di-2-propenyl $$ Allyl disulphide $$ 4,5-Dithia-

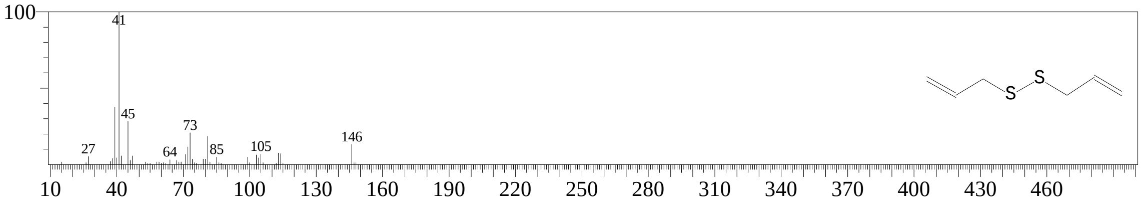

Hit#:5 Entry:3168 Library:NIST05s.LIB
SI:75 Formula:C6H10S CAS:592-88-1 MolWeight:114 RetIndex:849
CompName:1-Propene, 3,3'-thiobis- $$ Allyl sulfide $$ Allyl monosulfide $$ Diallyl monosulfide $$ Diallyl sulfide $$ Diallyl thioa

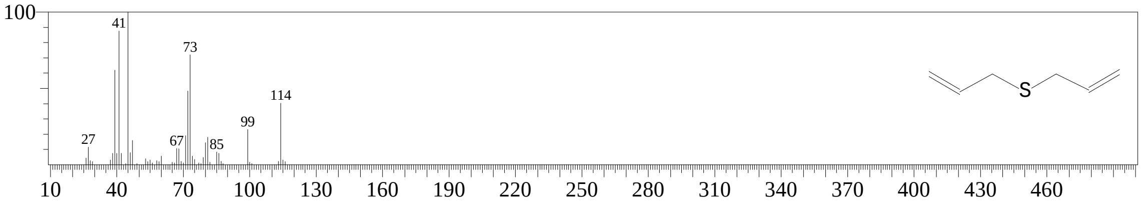

<< Target >>
Line#:7   R.Time:15.925(Scan#:1552) MassPeaks:238   BasePeak:57.00(3531)
RawMode:Averaged 15.917-15.933(1551-1553)   BG Mode:Calc. from Peak

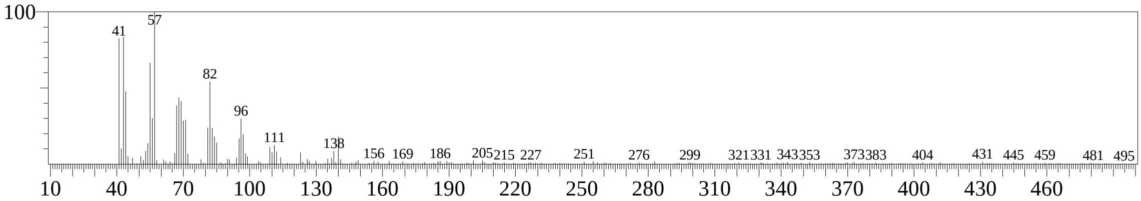

Hit#:1 Entry:14089 Library:NIST05s.LIB
SI:93 Formula:C12H24O CAS:112-54-9 MolWeight:184 RetIndex:1402
CompName:Dodecanal $$ Lauraldehyde $$ n-Dodecanal $$ n-Dodecyl aldehyde $$ n-Dodecylic aldehyde $$ n-Lauraldehyde $$ A[illegible]

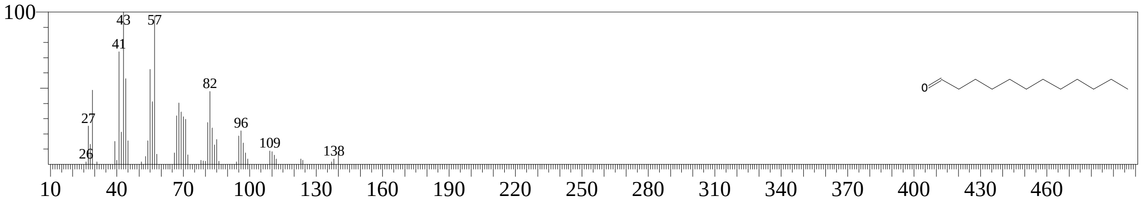

Hit#:2 Entry:65429 Library:NIST05.LIB
SI:92 Formula:C16H32O CAS:629-80-1 MolWeight:240 RetIndex:1800
CompName:Hexadecanal $$ Palmitaldehyde $$

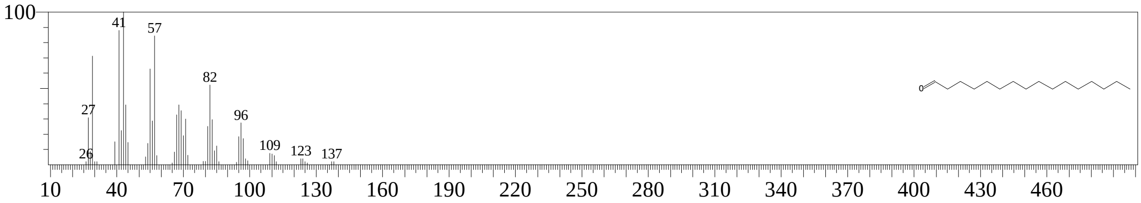

Hit#:3 Entry:14087 Library:NIST05s.LIB
SI:92 Formula:C12H24O CAS:112-54-9 MolWeight:184 RetIndex:1402
CompName:Dodecanal $$ Lauraldehyde $$ n-Dodecanal $$ n-Dodecyl aldehyde $$ n-Dodecylic aldehyde $$ n-Lauraldehyde $$ A[illegible]

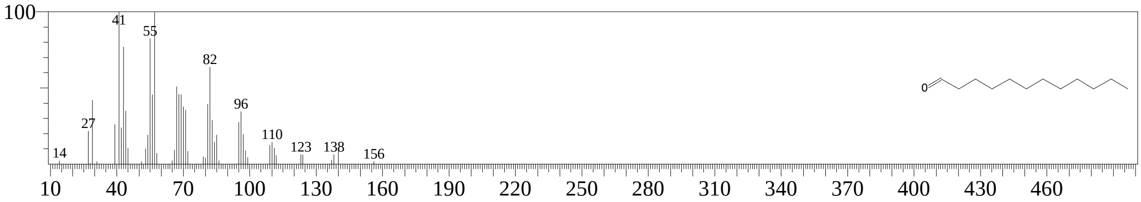

Hit#:4 Entry:40204 Library:NIST05.LIB
SI:91 Formula:C13H26O CAS:10486-19-8 MolWeight:198 RetIndex:1502
CompName:Tridecanal $$ n-Tridecylaldehyde $$ Tridecanaldehyde $$ Tridecyl aldehyde $$ 1-Tridecanal $$ Tridecane aldehyde $[illegible]

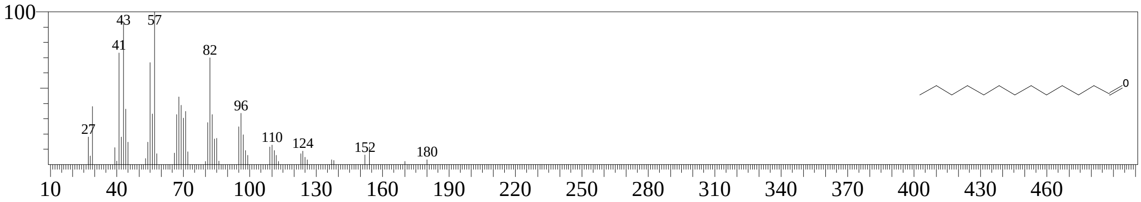

Hit#:5 Entry:16032 Library:NIST05s.LIB
SI:91 Formula:C13H26O CAS:10486-19-8 MolWeight:198 RetIndex:1502
CompName:Tridecanal $$ n-Tridecylaldehyde $$ Tridecanaldehyde $$ Tridecyl aldehyde $$ 1-Tridecanal $$ Tridecane aldehyde $[illegible]

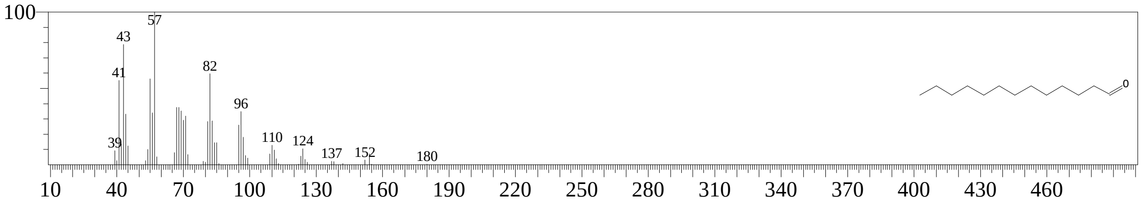

<< Target >>
Line#:8   R.Time:16.675(Scan#:1642) MassPeaks:275   BasePeak:57.00(188855)
RawMode:Averaged 16.667-16.683(1641-1643)   BG Mode:Calc. from Peak

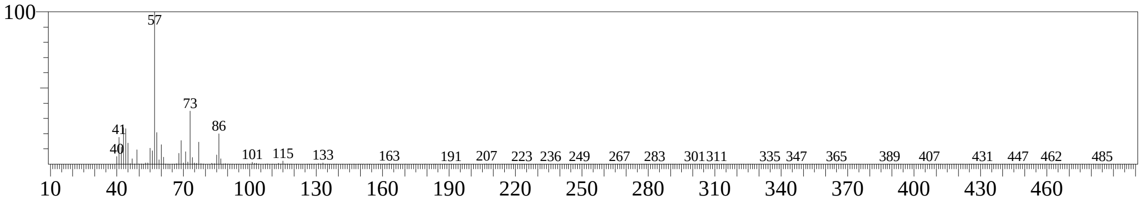

Hit#:1 Entry:8563 Library:NIST05s.LIB
SI:83 Formula:C4H9NO5 CAS:126-11-4 MolWeight:151 RetIndex:1444
CompName:1,3-Propanediol, 2-(hydroxymethyl)-2-nitro- $$ Isobutylglycerol, nitro- $$ Methane, trimethylolnitro- $$ Nitroisobutylg

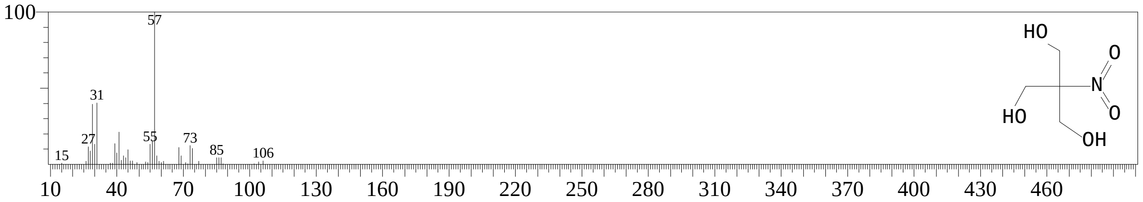

Hit#:2 Entry:15081 Library:NIST05.LIB
SI:82 Formula:C4H9NO5 CAS:126-11-4 MolWeight:151 RetIndex:1444
CompName:1,3-Propanediol, 2-(hydroxymethyl)-2-nitro- $$ Isobutylglycerol, nitro- $$ Methane, trimethylolnitro- $$ Nitroisobutylg

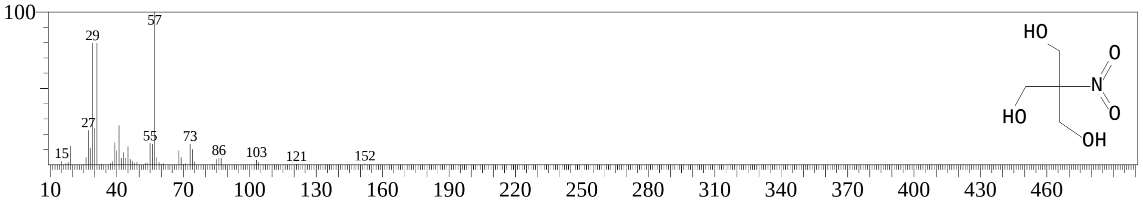

Hit#:3 Entry:5797 Library:NIST05s.LIB
SI:82 Formula:C6H14O3 CAS:77-99-6 MolWeight:134 RetIndex:1261
CompName:1,3-Propanediol, 2-ethyl-2-(hydroxymethyl)- $$ Ethriol $$ Ethyltrimethylolmethane $$ Etriol $$ Ettriol $$ Hexaglyceri

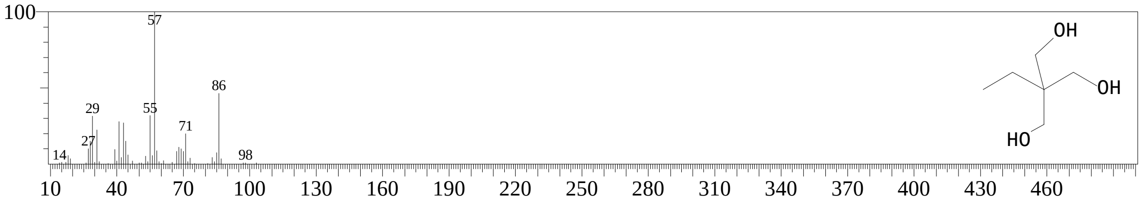

Hit#:4 Entry:123174 Library:NIST05.LIB
SI:82 Formula:C12H22O11 CAS:57-50-1 MolWeight:342 RetIndex:3139
CompName:Sucrose $$ .alpha.-D-Glucopyranoside, .beta.-D-fructofuranosyl $$ .beta.-D-Fructofuranosyl .alpha.-D-glucopyranoside

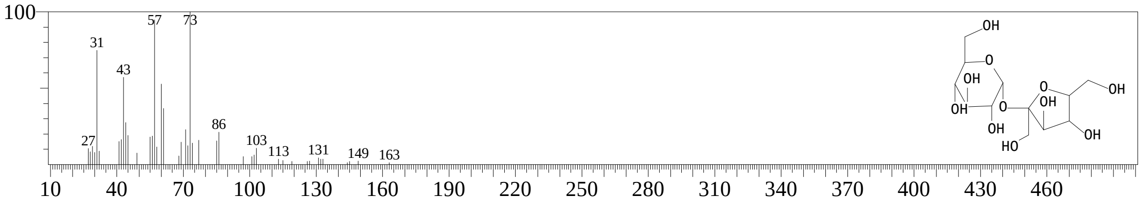

Hit#:5 Entry:1952 Library:NIST05s.LIB
SI:81 Formula:C4H6O3 CAS:108-32-7 MolWeight:102 RetIndex:875
CompName:Propylene Carbonate $$ 1,3-Dioxolan-2-one, 4-methyl- $$ Carbonic acid, cyclic propylene ester $$ Cyclic methylethyl

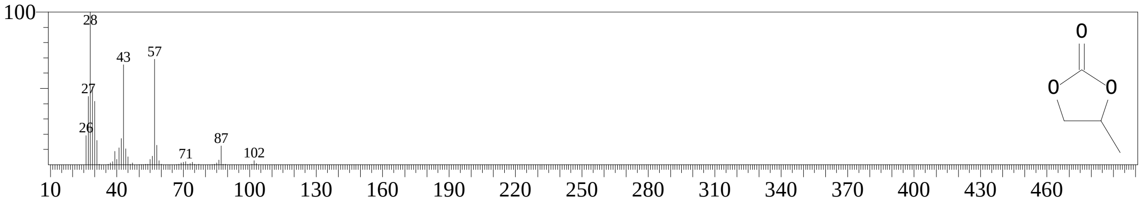

<< Target >>
Line#:9 R.Time:16.817(Scan#:1659) MassPeaks:258 BasePeak:43.00(865)
RawMode:Averaged 16.808-16.825(1658-1660) BG Mode:Calc. from Peak

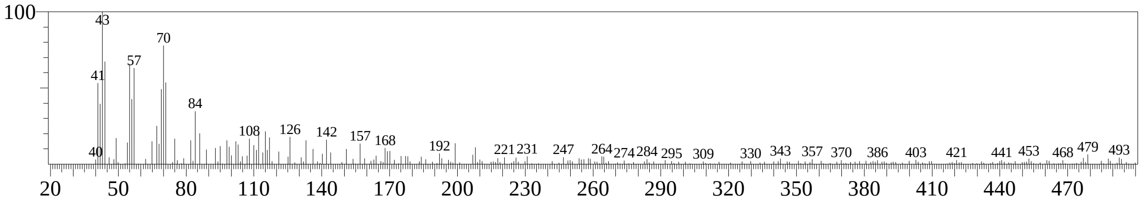

Hit#:1 Entry:24211 Library:NIST05.LIB
SI:72 Formula:C11H23N CAS:14924-95-9 MolWeight:169 RetIndex:1268
CompName:n-Nonane, 1-[1-cycloazapropyl]- $$ 1-Nonylaziridine # $$

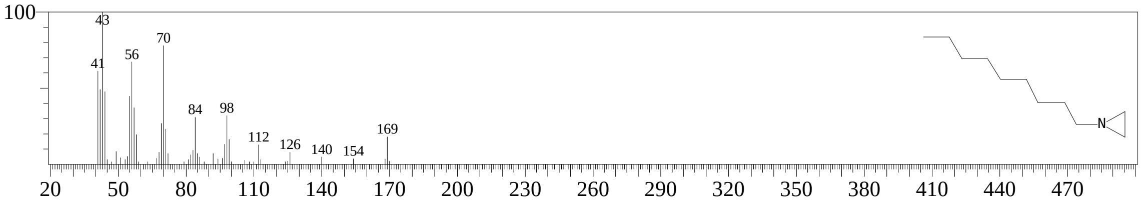

Hit#:2 Entry:101862 Library:NIST05.LIB
SI:72 Formula:C12H21Cl3O2 CAS:65611-33-8 MolWeight:302 RetIndex:1769
CompName:Trichloroacetic acid, decyl ester $$ Decyl trichloroacetate # $$

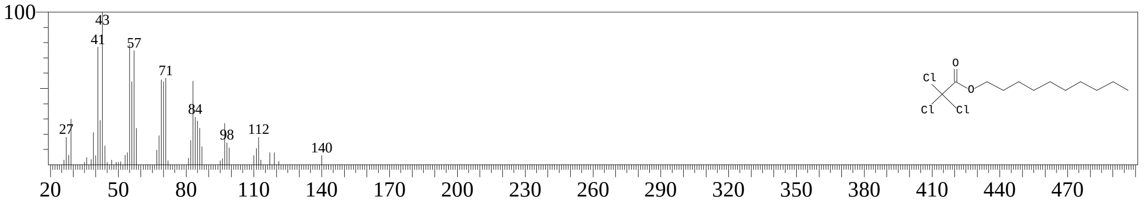

Hit#:3 Entry:93695 Library:NIST05.LIB
SI:72 Formula:C11H19Cl3O2 CAS:65611-32-7 MolWeight:288 RetIndex:1669
CompName:Acetic acid, trichloro-, nonyl ester $$ Trichloroacetic acid, nonyl ester $$ Nonyl trichloroacetate # $$

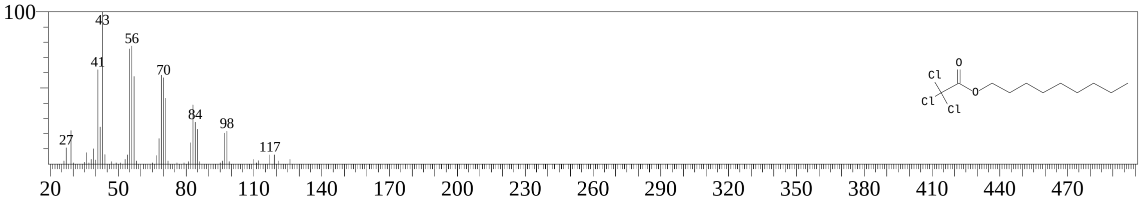

Hit#:4 Entry:85415 Library:NIST05.LIB
SI:71 Formula:C10H17Cl3O2 CAS:16958-78-4 MolWeight:274 RetIndex:1570
CompName:Acetic acid, trichloro-, octyl ester $$ Trichloroacetic acid, octyl ester $$ Octyl trichloroacetate # $$

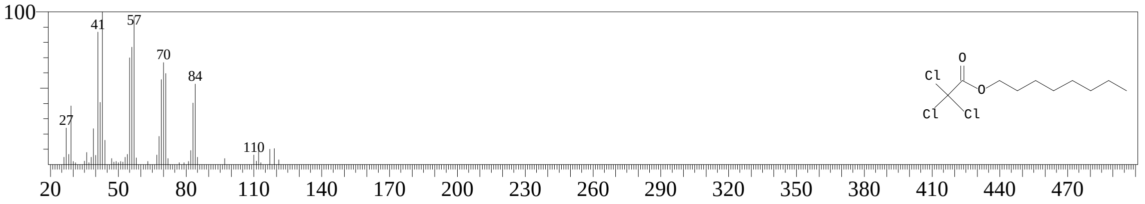

Hit#:5 Entry:9721 Library:NIST05s.LIB
SI:71 Formula:C10H20O CAS:112-31-2 MolWeight:156 RetIndex:1204
CompName:Decanal $$ n-Decaldehyde $$ n-Decanal $$ n-Decyl aldehyde $$ Aldehyde C10 $$ Capraldehyde $$ Capric aldehyde

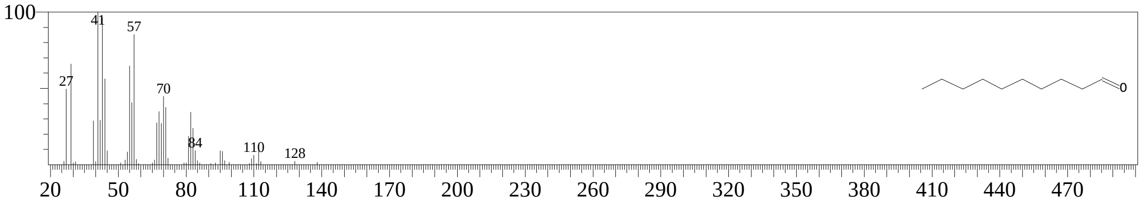

<< Target >>
Line#:10   R.Time:17.383(Scan#:1727) MassPeaks:284   BasePeak:191.10(10016)
RawMode:Averaged 17.375-17.392(1726-1728)   BG Mode:Calc. from Peak

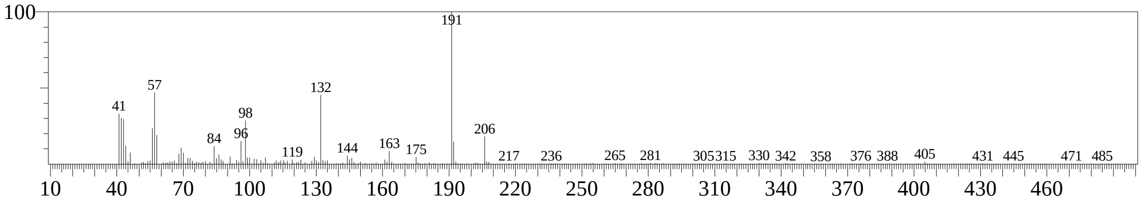

Hit#:1 Entry:16996 Library:NIST05s.LIB
SI:65 Formula:C14H22O CAS:1138-52-9 MolWeight:206 RetIndex:1555
CompName:Phenol, 3,5-bis(1,1-dimethylethyl)- $$ Phenol, 3,5-di-tert-butyl- $$ 3,5-Di-tert-butylphenol $$ Phenol, 3,5-bis(t-butyl) $

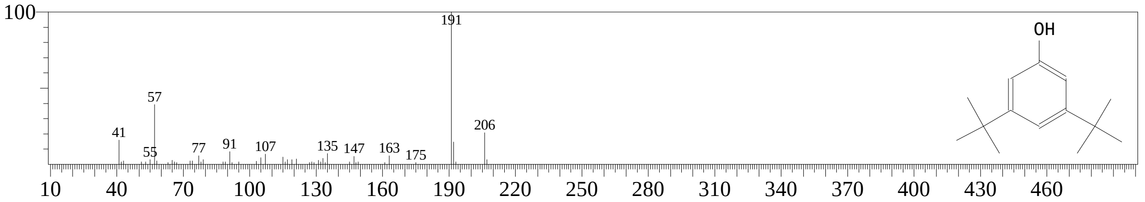

Hit#:2 Entry:44736 Library:NIST05.LIB
SI:63 Formula:C14H22O CAS:128-39-2 MolWeight:206 RetIndex:1555
CompName:Phenol, 2,6-bis(1,1-dimethylethyl)- $$ Phenol, 2,6-di-tert-butyl- $$ 2,6-Bis(tert-butyl)phenol $$ 2,6-Bis(1,1-dimethylet

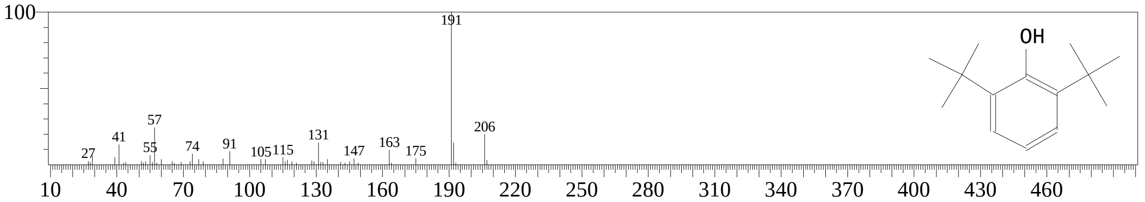

Hit#:3 Entry:16995 Library:NIST05s.LIB
SI:62 Formula:C14H22O CAS:1138-52-9 MolWeight:206 RetIndex:1555
CompName:Phenol, 3,5-bis(1,1-dimethylethyl)- $$ Phenol, 3,5-di-tert-butyl- $$ 3,5-Di-tert-butylphenol $$ Phenol, 3,5-bis(t-butyl) $

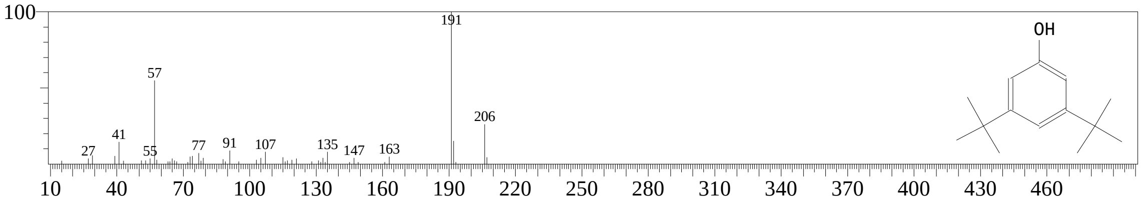

Hit#:4 Entry:104627 Library:NIST05.LIB
SI:61 Formula:C19H30O3 CAS:166273-38-7 MolWeight:306 RetIndex:2255
CompName:Pentanoic acid, 5-hydroxy-, 2,4-di-t-butylphenyl esters $$ 2,4-Ditert-butylphenyl 5-hydroxypentanoate # $$

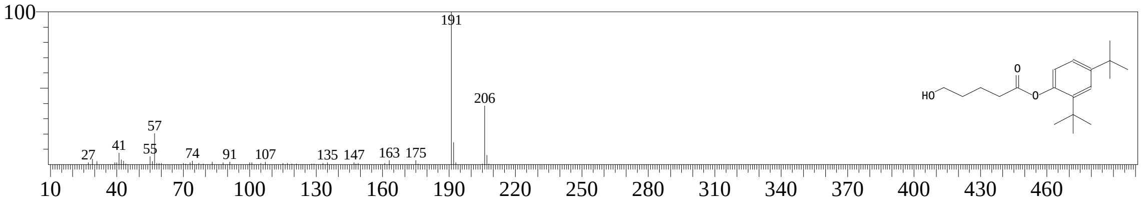

Hit#:5 Entry:16990 Library:NIST05s.LIB
SI:61 Formula:C14H22O CAS:128-39-2 MolWeight:206 RetIndex:1555
CompName:Phenol, 2,6-bis(1,1-dimethylethyl)- $$ Phenol, 2,6-di-tert-butyl- $$ 2,6-Bis(tert-butyl)phenol $$ 2,6-Bis(1,1-dimethylet

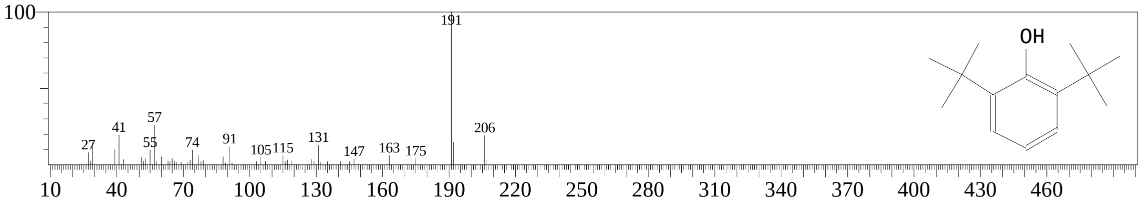

<< Target >>
Line#:11   R.Time:18.433(Scan#:1853) MassPeaks:247   BasePeak:57.00(4750)
RawMode:Averaged 18.425-18.442(1852-1854)   BG Mode:Calc. from Peak

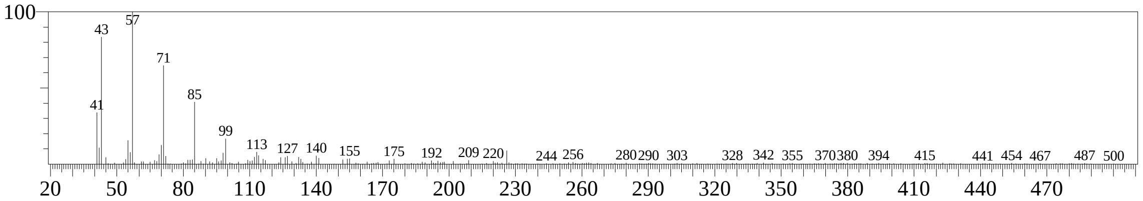

Hit#:1 Entry:19090 Library:NIST05s.LIB
SI:89 Formula:C16H34 CAS:544-76-3 MolWeight:226 RetIndex:1612
CompName:Hexadecane $$ n-Cetane $$ n-Hexadecane $$ Cetane $$

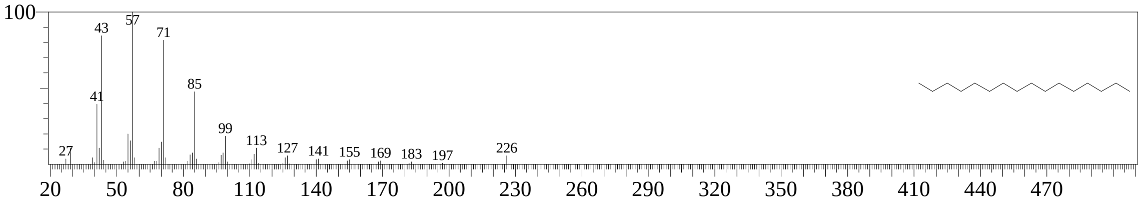

Hit#:2 Entry:19088 Library:NIST05s.LIB
SI:88 Formula:C16H34 CAS:544-76-3 MolWeight:226 RetIndex:1612
CompName:Hexadecane $$ n-Cetane $$ n-Hexadecane $$ Cetane $$

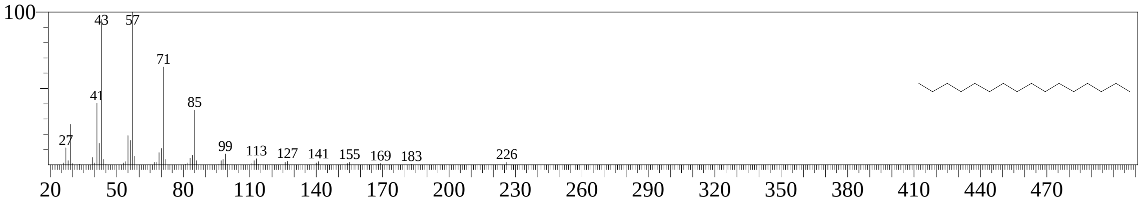

Hit#:3 Entry:16058 Library:NIST05s.LIB
SI:88 Formula:C14H30 CAS:629-59-4 MolWeight:198 RetIndex:1413
CompName:Tetradecane $$ n-Tetradecane $$

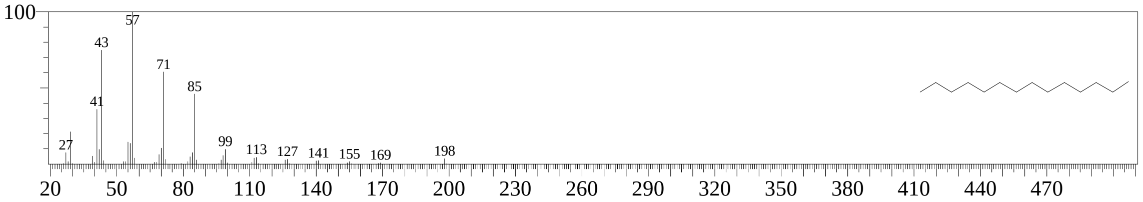

Hit#:4 Entry:17696 Library:NIST05s.LIB
SI:88 Formula:C15H32 CAS:629-62-9 MolWeight:212 RetIndex:1512
CompName:Pentadecane $$ n-Pentadecane $$ CH3(CH2)13CH3 $$

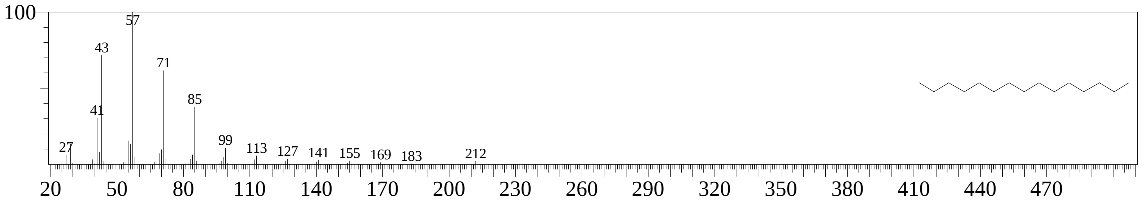

Hit#:5 Entry:16060 Library:NIST05s.LIB
SI:88 Formula:C14H30 CAS:629-59-4 MolWeight:198 RetIndex:1413
CompName:Tetradecane $$ n-Tetradecane $$

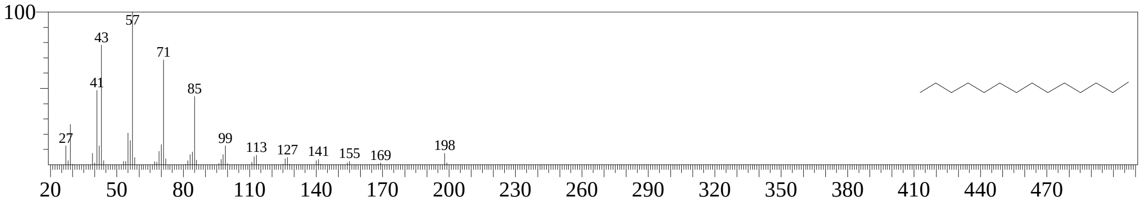

<< Target >>
Line#:12 R.Time:18.633(Scan#:1877) MassPeaks:263 BasePeak:57.00(2222)
RawMode:Averaged 18.625-18.642(1876-1878) BG Mode:Calc. from Peak

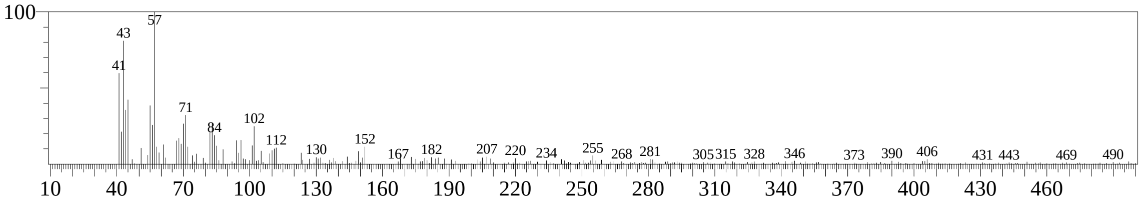

Hit#:1 Entry:14089 Library:NIST05s.LIB
SI:78 Formula:C12H24O CAS:112-54-9 MolWeight:184 RetIndex:1402
CompName:Dodecanal $$ Lauraldehyde $$ n-Dodecanal $$ n-Dodecyl aldehyde $$ n-Dodecylic aldehyde $$ n-Lauraldehyde $$ A

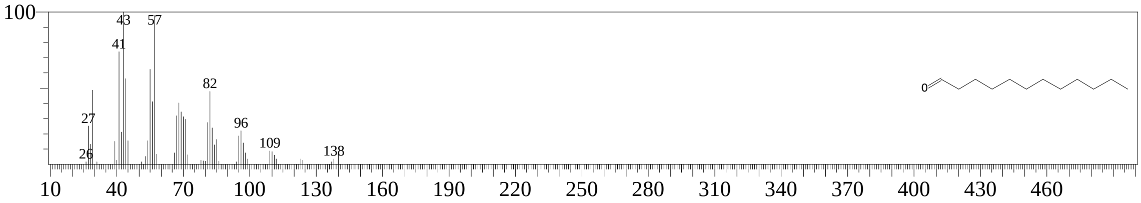

Hit#:2 Entry:9721 Library:NIST05s.LIB
SI:77 Formula:C10H20O CAS:112-31-2 MolWeight:156 RetIndex:1204
CompName:Decanal $$ n-Decaldehyde $$ n-Decanal $$ n-Decyl aldehyde $$ Aldehyde C10 $$ Capraldehyde $$ Capric aldehyde

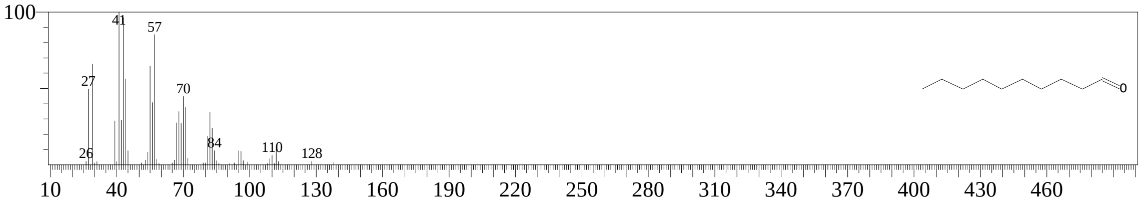

Hit#:3 Entry:9740 Library:NIST05s.LIB
SI:77 Formula:C10H20O CAS:18409-18-2 MolWeight:156 RetIndex:1266
CompName:2-Decen-1-ol, (E)- $$ trans-2-Decenol $$ Dec-2-en-1-ol $$ (2E)-2-Decen-1-ol # $$

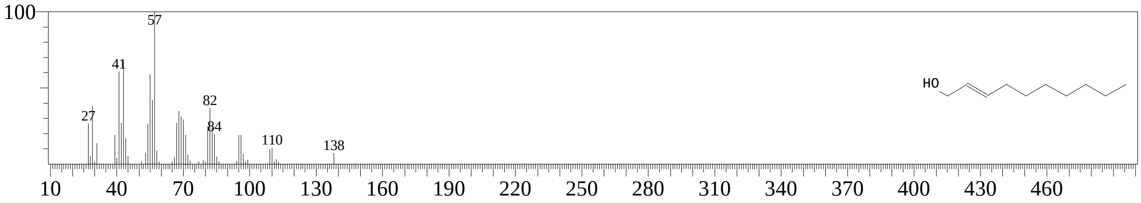

Hit#:4 Entry:23579 Library:NIST05s.LIB
SI:77 Formula:C20H40O CAS:930-2-9 MolWeight:296 RetIndex:2075
CompName:Octadecane, 1-(ethenyloxy)- $$ Ether, octadecyl vinyl $$ Octadecyl vinyl ether $$ Stearyl vinyl ether $$ Vinyl stearyl e

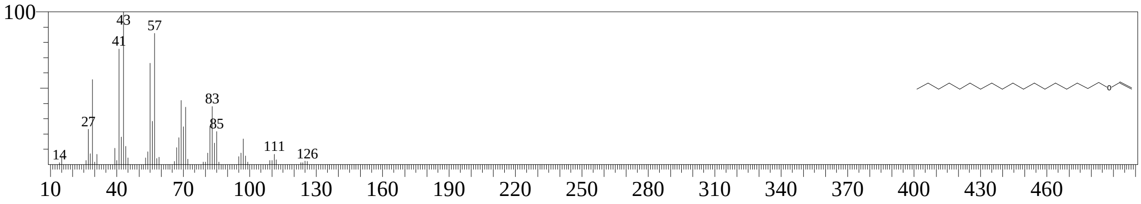

Hit#:5 Entry:16032 Library:NIST05s.LIB
SI:77 Formula:C13H26O CAS:10486-19-8 MolWeight:198 RetIndex:1502
CompName:Tridecanal $$ n-Tridecylaldehyde $$ Tridecanaldehyde $$ Tridecyl aldehyde $$ 1-Tridecanal $$ Tridecane aldehyde $

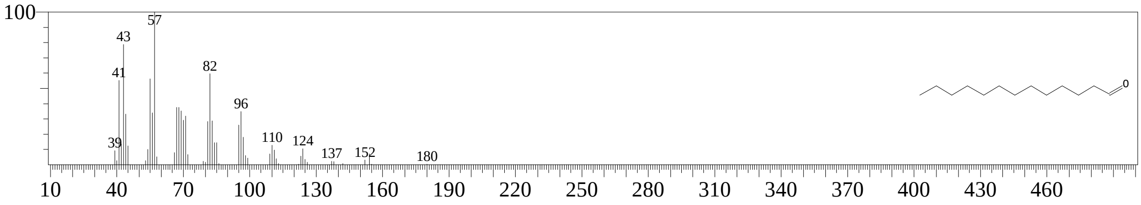

<< Target >>
Line#:13 R.Time:19.500(Scan#:1981) MassPeaks:248 BasePeak:144.00(48864)
RawMode:Averaged 19.492-19.508(1980-1982) BG Mode:Calc. from Peak

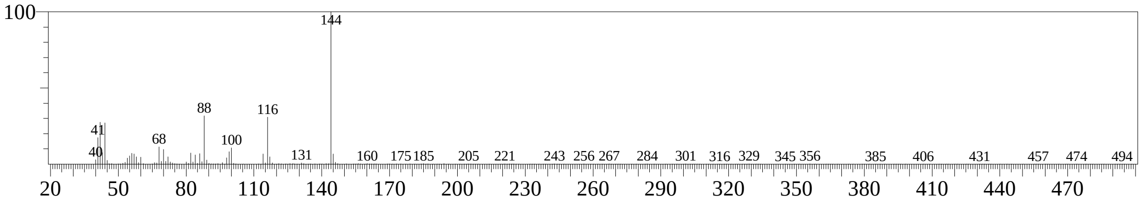

Hit#:1 Entry:67958 Library:NIST05.LIB
SI:82 Formula:C12H23NO4 CAS:0-0-0 MolWeight:245 RetIndex:1619
CompName:l-Alanine, N-butoxycarbonyl-, isobutyl ester

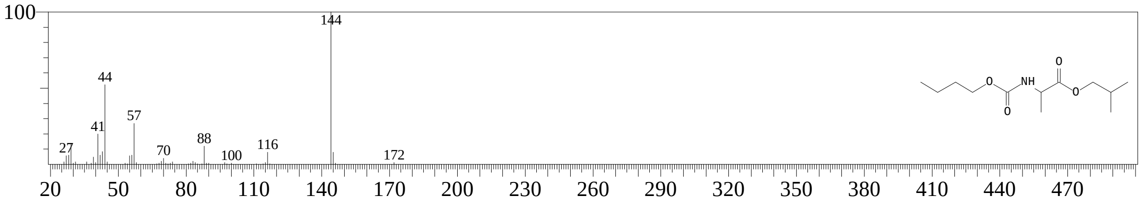

Hit#:2 Entry:109550 Library:NIST05.LIB
SI:81 Formula:C17H33NO4 CAS:0-0-0 MolWeight:315 RetIndex:2180
CompName:l-Alanine, N-butoxycarbonyl-, nonyl ester

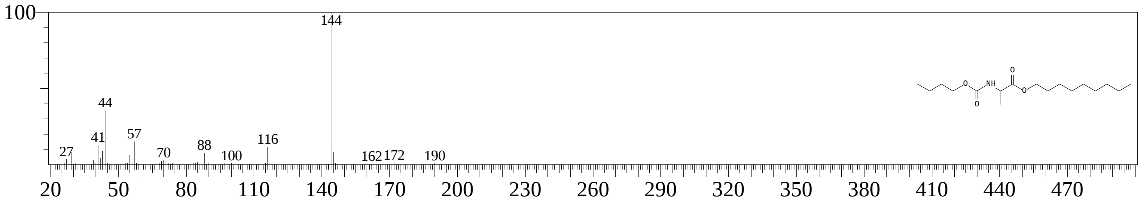

Hit#:3 Entry:93408 Library:NIST05.LIB
SI:81 Formula:C15H29NO4 CAS:0-0-0 MolWeight:287 RetIndex:1981
CompName:l-Alanine, N-butoxycarbonyl-, heptyl ester

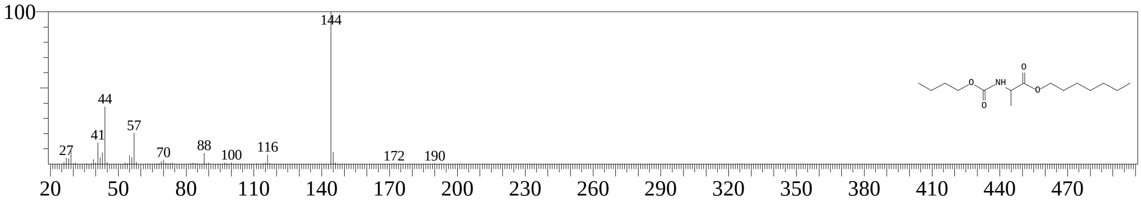

Hit#:4 Entry:76475 Library:NIST05.LIB
SI:80 Formula:C13H25NO4 CAS:0-0-0 MolWeight:259 RetIndex:1782
CompName:l-Alanine, N-butoxycarbonyl-, pentyl ester

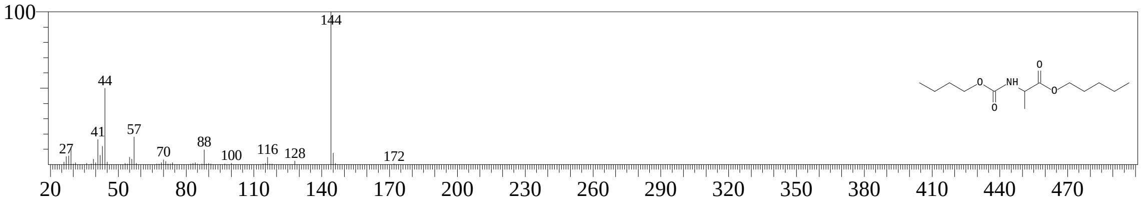

Hit#:5 Entry:59662 Library:NIST05.LIB
SI:80 Formula:C11H21NO4 CAS:0-0-0 MolWeight:231 RetIndex:1584
CompName:l-Alanine, N-butoxycarbonyl-, propyl ester

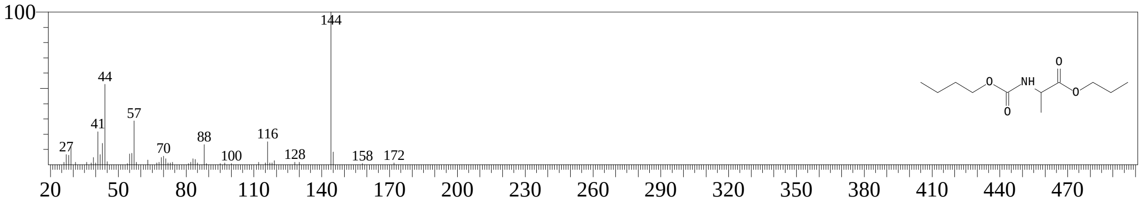

<< Target >>
Line#:14   R.Time:19.667(Scan#:2001) MassPeaks:257   BasePeak:57.00(1508)
RawMode:Averaged 19.658-19.675(2000-2002)   BG Mode:Calc. from Peak

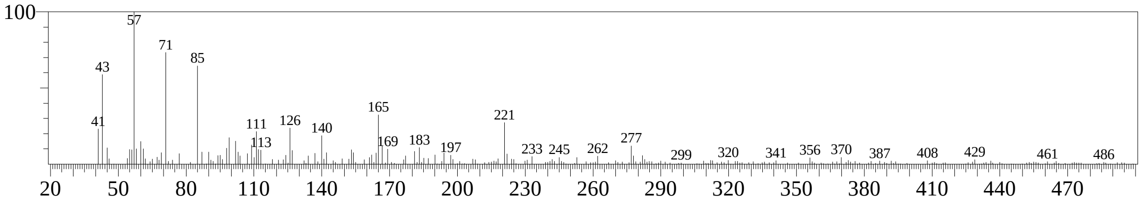

Hit#:1 Entry:22890 Library:NIST05s.LIB
SI:67 Formula:C20H42 CAS:112-95-8 MolWeight:282 RetIndex:2009
CompName:Eicosane $$ n-Eicosane $$ Icosane # $$

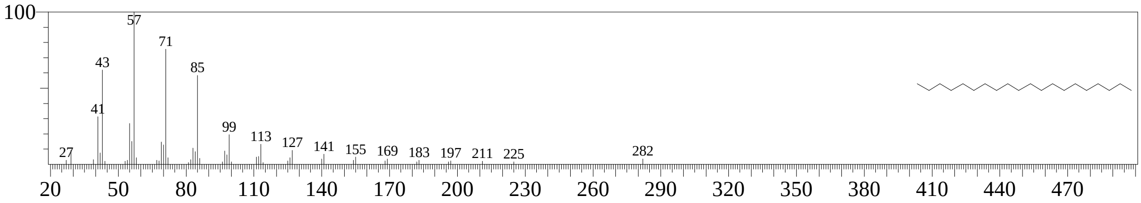

Hit#:2 Entry:73828 Library:NIST05.LIB
SI:67 Formula:C18H38 CAS:13287-23-5 MolWeight:254 RetIndex:1746
CompName:Heptadecane, 8-methyl- $$ 8-Methylheptadecane $$

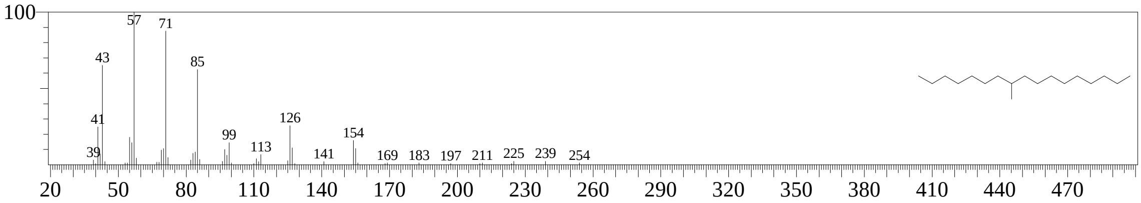

Hit#:3 Entry:23590 Library:NIST05s.LIB
SI:67 Formula:C21H44 CAS:629-94-7 MolWeight:296 RetIndex:2109
CompName:Heneicosane $$ n-Heneicosane $$ Henicosane # $$

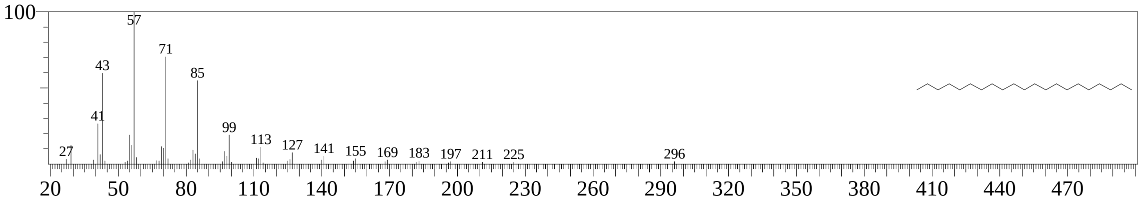

Hit#:4 Entry:20226 Library:NIST05s.LIB
SI:66 Formula:C17H36 CAS:629-78-7 MolWeight:240 RetIndex:1711
CompName:Heptadecane $$ n-Heptadecane $$ Normal-heptadecane $$

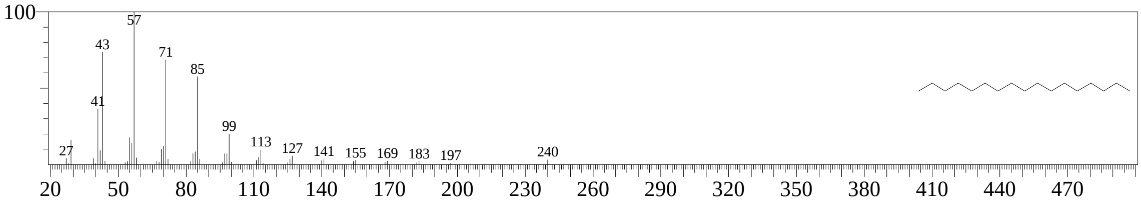

Hit#:5 Entry:22889 Library:NIST05s.LIB
SI:66 Formula:C20H42 CAS:112-95-8 MolWeight:282 RetIndex:2009
CompName:Eicosane $$ n-Eicosane $$ Icosane # $$

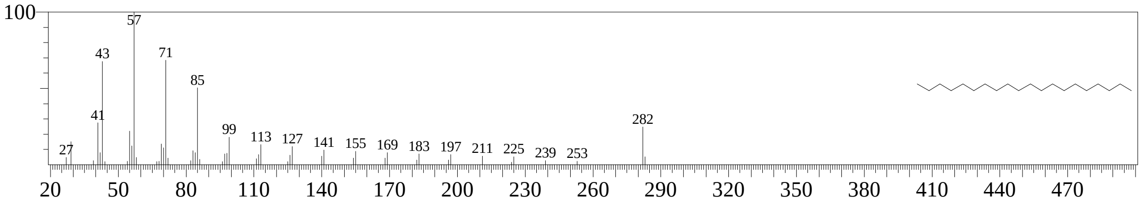

<< Target >>
Line#:15   R.Time:19.975(Scan#:2038) MassPeaks:253   BasePeak:74.00(1450)
RawMode:Averaged 19.967-19.983(2037-2039)   BG Mode:Calc. from Peak

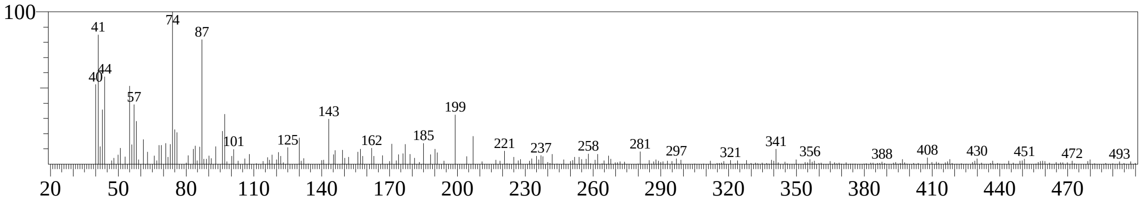

Hit#:1 Entry:100067 Library:NIST05.LIB
SI:70 Formula:C19H38O2 CAS:54934-57-5 MolWeight:298 RetIndex:2013
CompName:Heptadecanoic acid, 9-methyl-, methyl ester $$ Methyl 9-methylheptadecanoate # $$

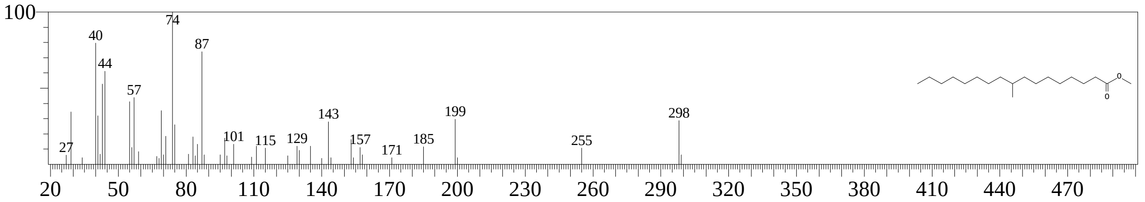

Hit#:2 Entry:149381 Library:NIST05.LIB
SI:68 Formula:C28H56O2 CAS:55682-91-2 MolWeight:424 RetIndex:2972
CompName:Heptacosanoic acid, methyl ester $$ Methyl heptacosanoate # $$

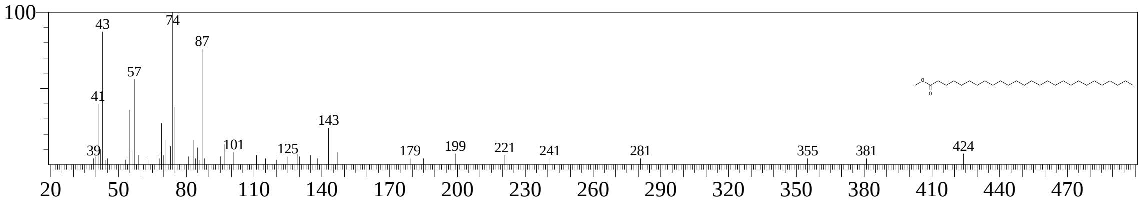

Hit#:3 Entry:26664 Library:NIST05s.LIB
SI:68 Formula:C27H54O2 CAS:5802-82-4 MolWeight:410 RetIndex:2872
CompName:Hexacosanoic acid, methyl ester $$ Methyl hexacosanoate $$ Cerotic acid methyl ester $$

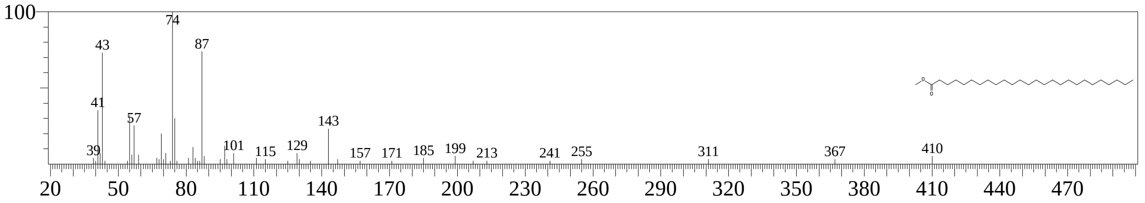

Hit#:4 Entry:100068 Library:NIST05.LIB
SI:68 Formula:C19H38O2 CAS:2490-25-7 MolWeight:298 RetIndex:2013
CompName:Heptadecanoic acid, 10-methyl-, methyl ester $$ Methyl 10-methylheptadecanoate # $$

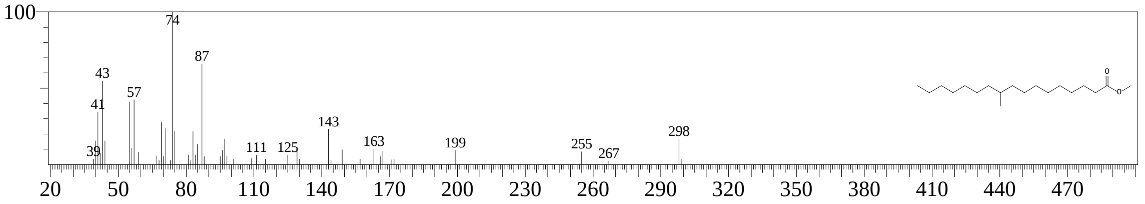

Hit#:5 Entry:27154 Library:NIST05s.LIB
SI:68 Formula:C31H62O2 CAS:629-83-4 MolWeight:466 RetIndex:3270
CompName:Triacontanoic acid, methyl ester $$ Methyl melissate $$ Methyl melissicate $$ Methyl triacontanoate $$

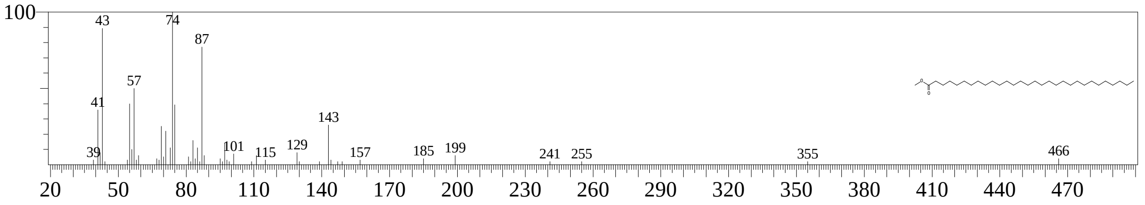

<< Target >>
Line#:16 R.Time:20.375(Scan#:2086) MassPeaks:244 BasePeak:60.00(1756)
RawMode:Averaged 20.367-20.383(2085-2087) BG Mode:Calc. from Peak

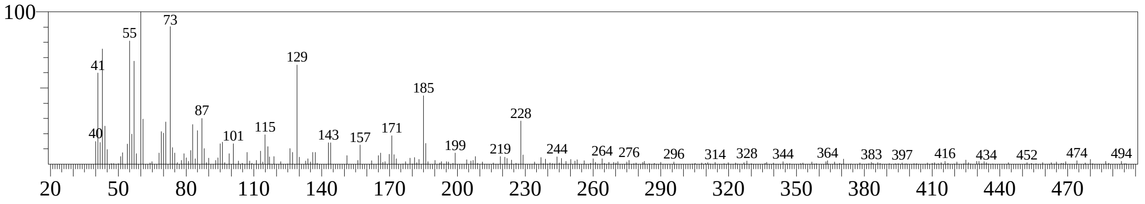

Hit#:1 Entry:19252 Library:NIST05s.LIB
SI:84 Formula:C14H28O2 CAS:544-63-8 MolWeight:228 RetIndex:1769
CompName:Tetradecanoic acid $$ Myristic acid $$ n-Tetradecanoic acid $$ n-Tetradecoic acid $$ Neo-Fat 14 $$ Univol U 316S $

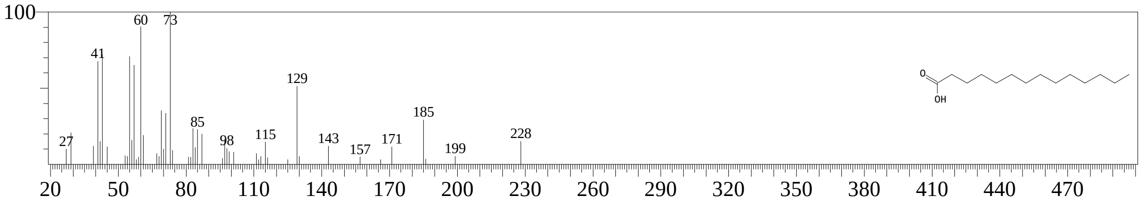

Hit#:2 Entry:19250 Library:NIST05s.LIB
SI:84 Formula:C14H28O2 CAS:544-63-8 MolWeight:228 RetIndex:1769
CompName:Tetradecanoic acid $$ Myristic acid $$ n-Tetradecanoic acid $$ n-Tetradecoic acid $$ Neo-Fat 14 $$ Univol U 316S $

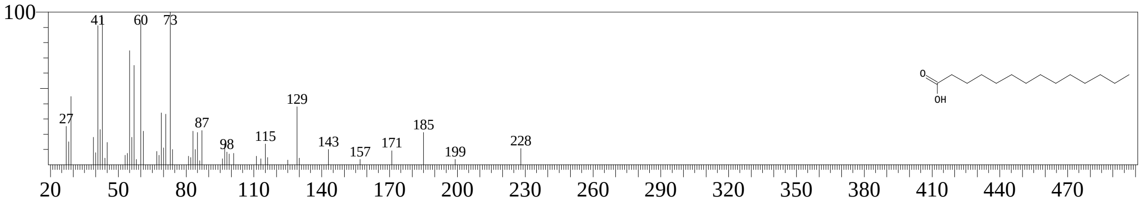

Hit#:3 Entry:58280 Library:NIST05.LIB
SI:82 Formula:C14H28O2 CAS:544-63-8 MolWeight:228 RetIndex:1769
CompName:Tetradecanoic acid $$ Myristic acid $$ n-Tetradecanoic acid $$ n-Tetradecoic acid $$ Neo-Fat 14 $$ Univol U 316S $

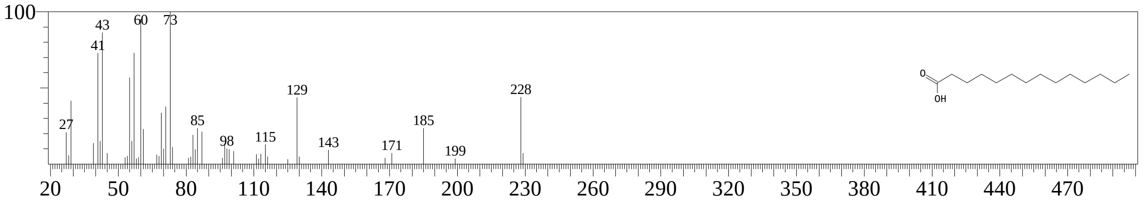

Hit#:4 Entry:19251 Library:NIST05s.LIB
SI:81 Formula:C14H28O2 CAS:544-63-8 MolWeight:228 RetIndex:1769
CompName:Tetradecanoic acid $$ Myristic acid $$ n-Tetradecanoic acid $$ n-Tetradecoic acid $$ Neo-Fat 14 $$ Univol U 316S $

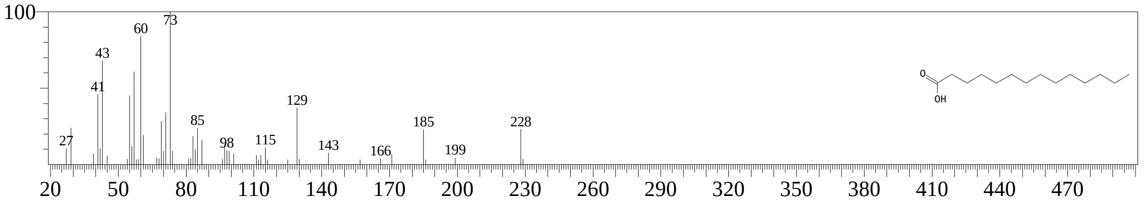

Hit#:5 Entry:20371 Library:NIST05s.LIB
SI:80 Formula:C15H30O2 CAS:1002-84-2 MolWeight:242 RetIndex:1869
CompName:Pentadecanoic acid $$ Pentadecylic acid $$ n-Pentadecanoic acid $$ n-Pentadecylic acid $$

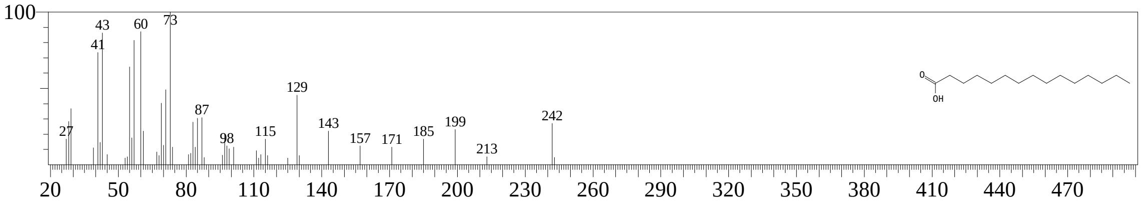

<< Target >>
Line#:17 R.Time:20.875(Scan#:2146) MassPeaks:231 BasePeak:141.00(5626)
RawMode:Averaged 20.867-20.883(2145-2147) BG Mode:Calc. from Peak

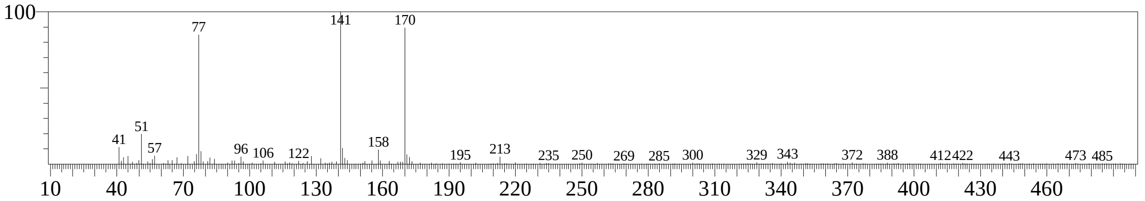

Hit#:1 Entry:17728 Library:NIST05s.LIB
SI:88 Formula:C10H15NO2S CAS:3622-84-2 MolWeight:213 RetIndex:1797
CompName:Benzenesulfonamide, N-butyl- $$ Benzenesulfonic acid butyl amide $$ N-Butylbenzenesulfonamide $$ Plastomoll BM

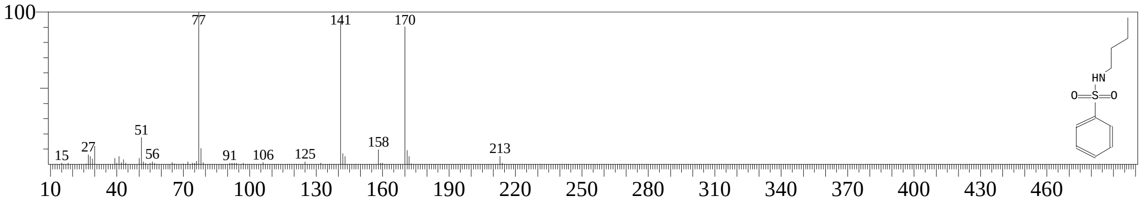

Hit#:2 Entry:48760 Library:NIST05.LIB
SI:88 Formula:C10H15NO2S CAS:3622-84-2 MolWeight:213 RetIndex:1797
CompName:Benzenesulfonamide, N-butyl- $$ Benzenesulfonic acid butyl amide $$ N-Butylbenzenesulfonamide $$ Plastomoll BM

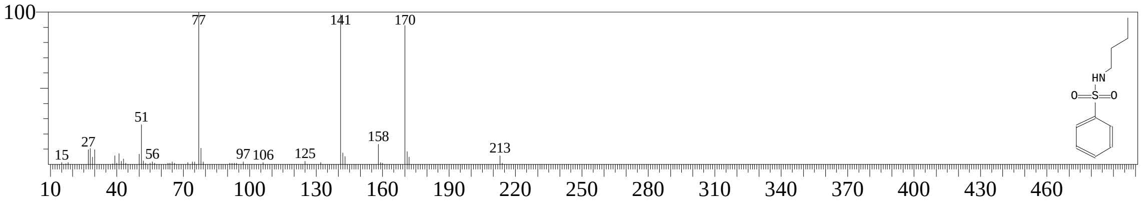

Hit#:3 Entry:46720 Library:NIST05.LIB
SI:73 Formula:C9H10N2O2S CAS:2619-21-8 MolWeight:210 RetIndex:1943
CompName:N-(2-Cyano-ethyl)-benzenesulfonamide

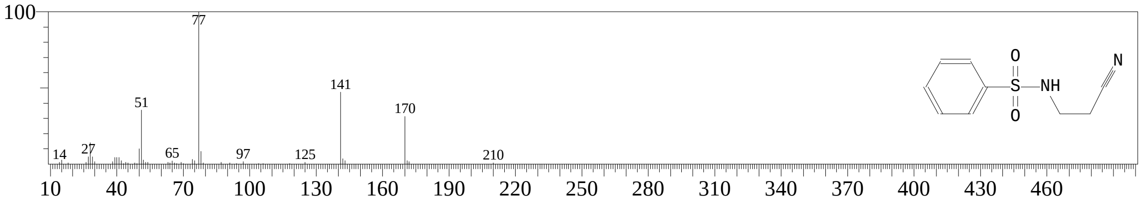

Hit#:4 Entry:143852 Library:NIST05.LIB
SI:72 Formula:C14H24NO4PS3 CAS:741-58-2 MolWeight:397 RetIndex:0
CompName:Bensulide $$ Phosphorodithioic acid, O,O-bis(1-methylethyl) S-[2-[(phenylsulfonyl)amino]ethyl] ester $$ Phosphorodi

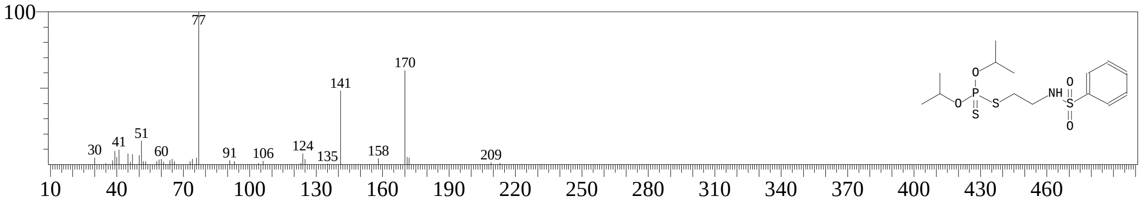

Hit#:5 Entry:77635 Library:NIST05.LIB
SI:71 Formula:C14H15NO2S CAS:77198-99-3 MolWeight:261 RetIndex:2271
CompName:N-Phenethylbenzenesulfonamide $$ N-(2-Phenylethyl)benzenesulfonamide # $$

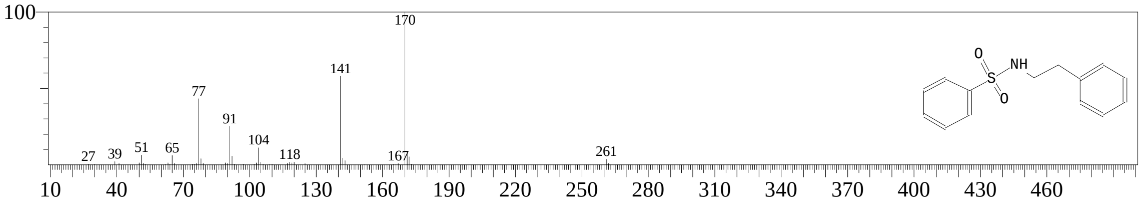

<< Target >>
Line#:18   R.Time:22.217(Scan#:2307) MassPeaks:274   BasePeak:74.00(14788)
RawMode:Averaged 22.208-22.225(2306-2308)   BG Mode:Calc. from Peak

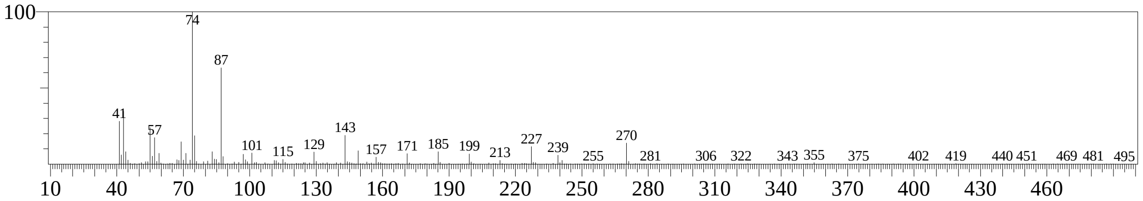

Hit#:1 Entry:83491 Library:NIST05.LIB
SI:95 Formula:C17H34O2 CAS:112-39-0 MolWeight:270 RetIndex:1878
CompName:Hexadecanoic acid, methyl ester $$ Palmitic acid, methyl ester $$ n-Hexadecanoic acid methyl ester $$ Metholene 221

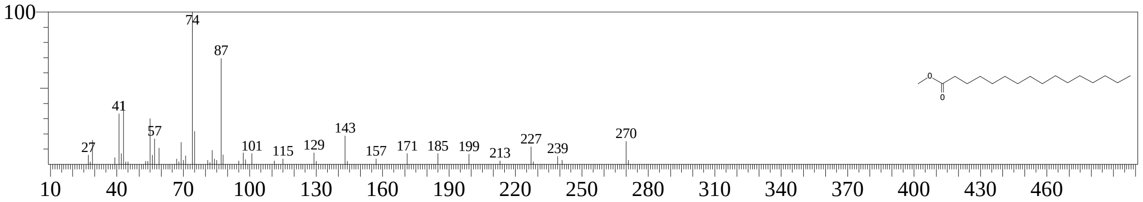

Hit#:2 Entry:22219 Library:NIST05s.LIB
SI:93 Formula:C17H34O2 CAS:112-39-0 MolWeight:270 RetIndex:1878
CompName:Hexadecanoic acid, methyl ester $$ Palmitic acid, methyl ester $$ n-Hexadecanoic acid methyl ester $$ Metholene 221

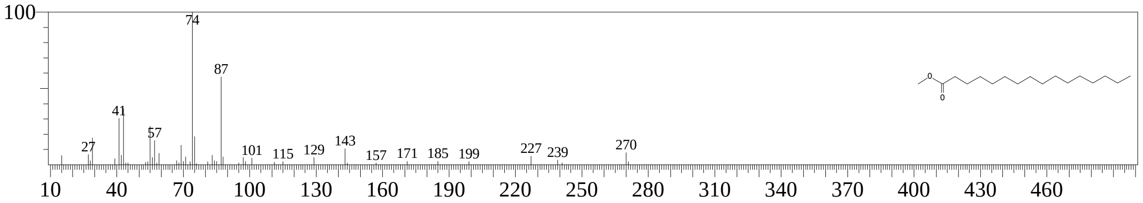

Hit#:3 Entry:22220 Library:NIST05s.LIB
SI:92 Formula:C17H34O2 CAS:112-39-0 MolWeight:270 RetIndex:1878
CompName:Hexadecanoic acid, methyl ester $$ Palmitic acid, methyl ester $$ n-Hexadecanoic acid methyl ester $$ Metholene 221

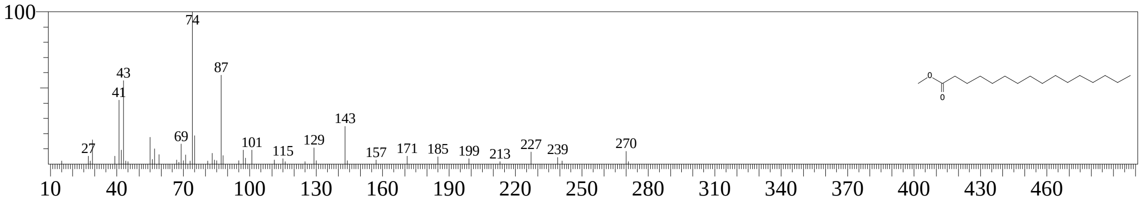

Hit#:4 Entry:22223 Library:NIST05s.LIB
SI:91 Formula:C17H34O2 CAS:5129-60-2 MolWeight:270 RetIndex:1814
CompName:Pentadecanoic acid, 14-methyl-, methyl ester $$ Methyl 14-methylpentadecanoate # $$

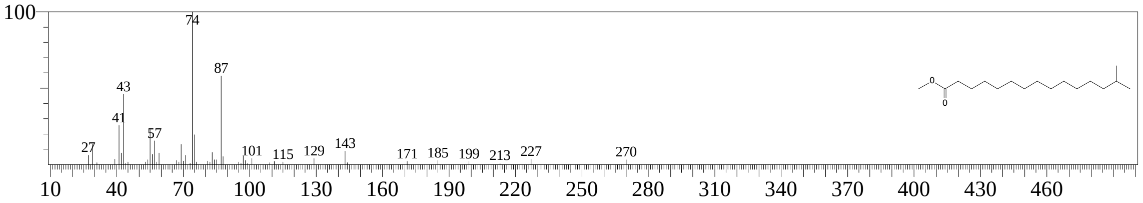

Hit#:5 Entry:22221 Library:NIST05s.LIB
SI:91 Formula:C17H34O2 CAS:112-39-0 MolWeight:270 RetIndex:1878
CompName:Hexadecanoic acid, methyl ester $$ Palmitic acid, methyl ester $$ n-Hexadecanoic acid methyl ester $$ Metholene 221

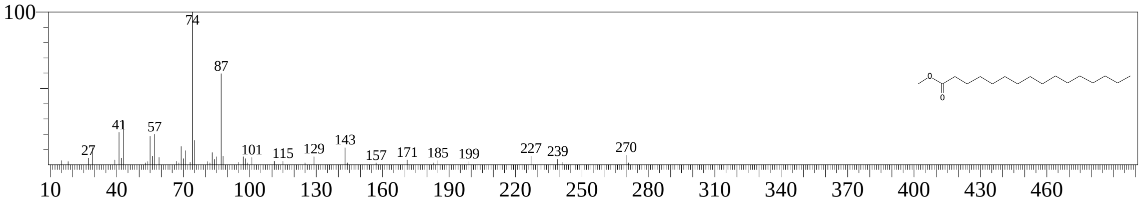

<< Target >>
Line#:19 R.Time:22.383(Scan#:2327) MassPeaks:263 BasePeak:41.00(2346)
RawMode:Averaged 22.375-22.392(2326-2328) BG Mode:Calc. from Peak

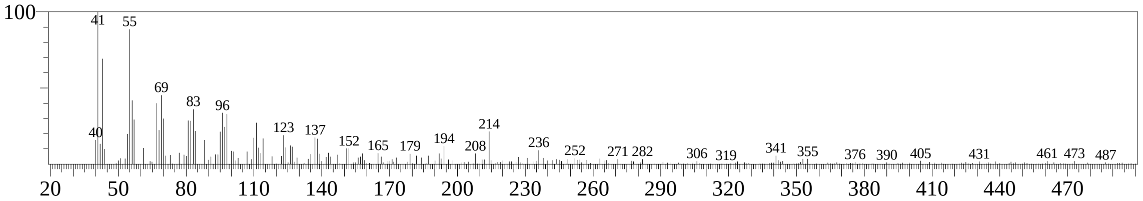

Hit#:1 Entry:73688 Library:NIST05.LIB
SI:81 Formula:C16H30O2 CAS:109-29-5 MolWeight:254 RetIndex:2264
CompName:Oxacycloheptadecan-2-one $$ Cyclohexadecanolide $$ Dihydroambrettolide $$ Hexadecanoic acid, 16-hydroxy-, .omi

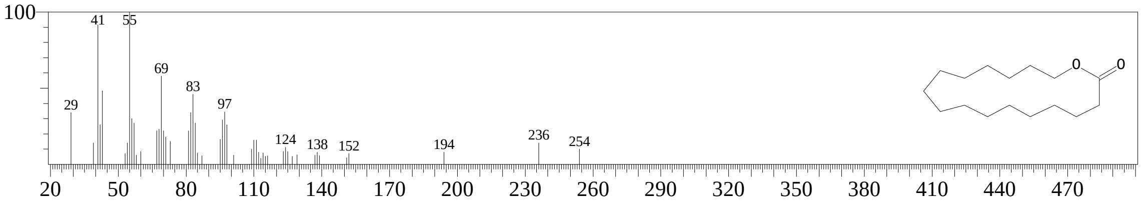

Hit#:2 Entry:65295 Library:NIST05.LIB
SI:80 Formula:C15H28O2 CAS:4727-18-8 MolWeight:240 RetIndex:2158
CompName:Cyclopentadecanone, 2-hydroxy- $$ 2-Hydroxycyclopentadecanone # $$

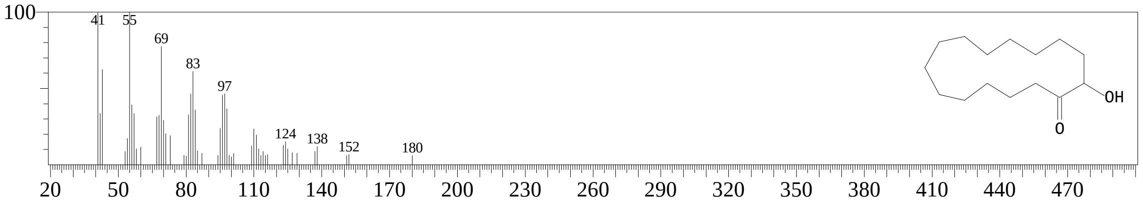

Hit#:3 Entry:73680 Library:NIST05.LIB
SI:80 Formula:C16H30O2 CAS:0-0-0 MolWeight:254 RetIndex:1976
CompName:Z-7-Hexadecenoic acid

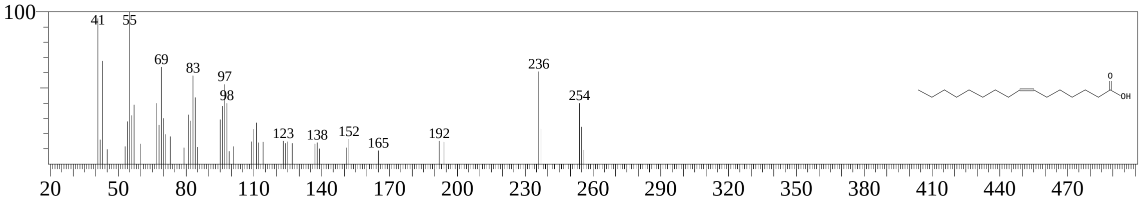

Hit#:4 Entry:73685 Library:NIST05.LIB
SI:80 Formula:C16H30O2 CAS:2091-29-4 MolWeight:254 RetIndex:1976
CompName:9-Hexadecenoic acid $$ (9E)-9-Hexadecenoic acid # $$

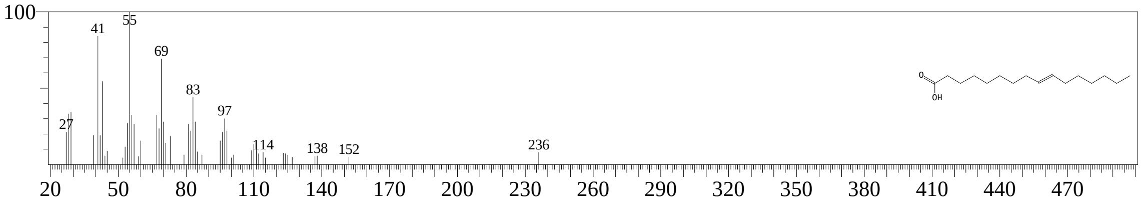

Hit#:5 Entry:17628 Library:NIST05s.LIB
SI:79 Formula:C13H24O2 CAS:1725-4-8 MolWeight:212 RetIndex:1904
CompName:Oxacyclotetradecan-2-one $$ Tridecanoic acid, 13-hydroxy-, .mu.-lactone $$ Tridecanolide $$ 1,13-Tridecanolide $$ -

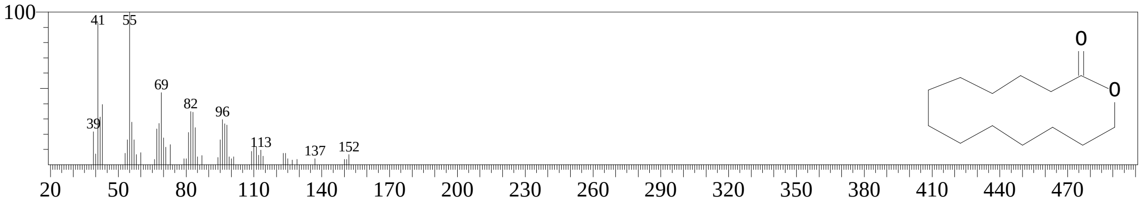

<< Target >>
Line#:20 R.Time:22.575(Scan#:2350) MassPeaks:295 BasePeak:43.00(13412)
RawMode:Averaged 22.567-22.583(2349-2351) BG Mode:Calc. from Peak

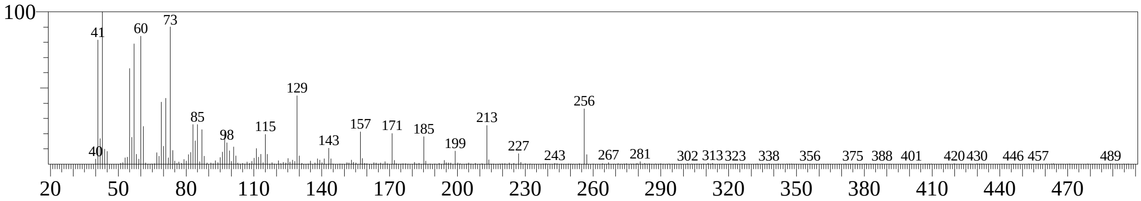

Hit#:1 Entry:21331 Library:NIST05s.LIB
SI:94 Formula:C16H32O2 CAS:57-10-3 MolWeight:256 RetIndex:1968
CompName:n-Hexadecanoic acid $$ Hexadecanoic acid $$ n-Hexadecoic acid $$ Palmitic acid $$ Pentadecanecarboxylic acid $$ [illegible]

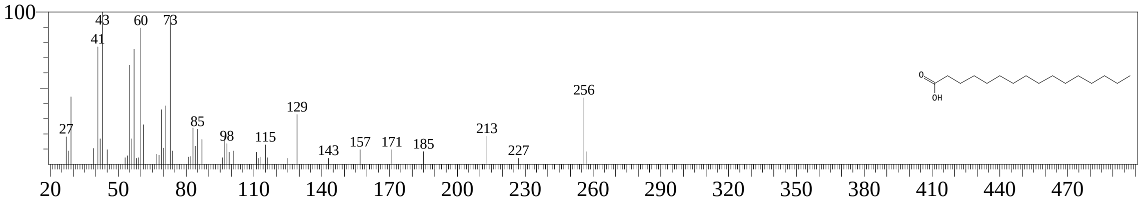

Hit#:2 Entry:20371 Library:NIST05s.LIB
SI:92 Formula:C15H30O2 CAS:1002-84-2 MolWeight:242 RetIndex:1869
CompName:Pentadecanoic acid $$ Pentadecylic acid $$ n-Pentadecanoic acid $$ n-Pentadecylic acid $$

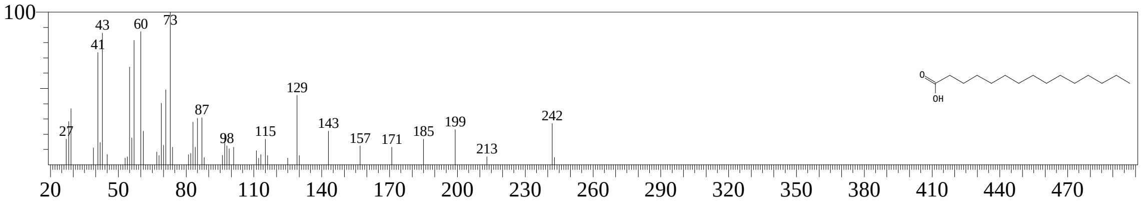

Hit#:3 Entry:161860 Library:NIST05.LIB
SI:91 Formula:C38H68O8 CAS:28474-90-0 MolWeight:652 RetIndex:4765
CompName:l-(+)-Ascorbic acid 2,6-dihexadecanoate

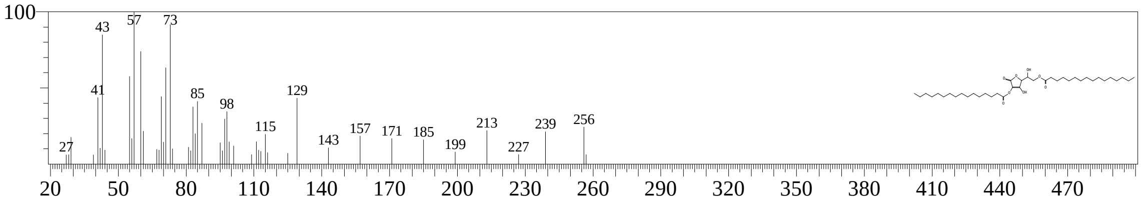

Hit#:4 Entry:74999 Library:NIST05.LIB
SI:90 Formula:C16H32O2 CAS:57-10-3 MolWeight:256 RetIndex:1968
CompName:n-Hexadecanoic acid $$ Hexadecanoic acid $$ n-Hexadecoic acid $$ Palmitic acid $$ Pentadecanecarboxylic acid $$ [illegible]

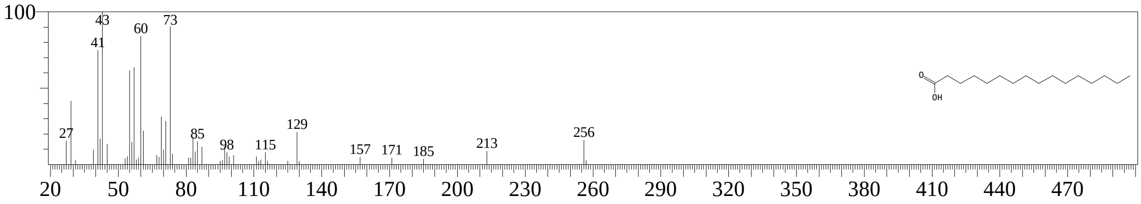

Hit#:5 Entry:19250 Library:NIST05s.LIB
SI:90 Formula:C14H28O2 CAS:544-63-8 MolWeight:228 RetIndex:1769
CompName:Tetradecanoic acid $$ Myristic acid $$ n-Tetradecanoic acid $$ n-Tetradecoic acid $$ Neo-Fat 14 $$ Univol U 316S [illegible]

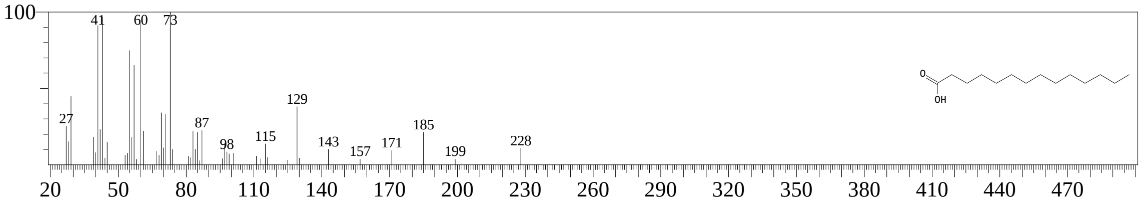

<< Target >>
Line#:21 R.Time:24.033(Scan#:2525) MassPeaks:277 BasePeak:55.00(903)
RawMode:Averaged 24.025-24.042(2524-2526) BG Mode:Calc. from Peak

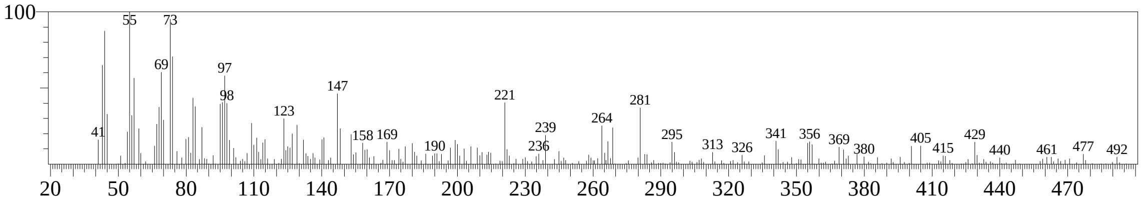

Hit#:1 Entry:121693 Library:NIST05.LIB
SI:69 Formula:C22H42O2 CAS:10152-64-4 MolWeight:338 RetIndex:2339
CompName:Cyclopropanedecanoic acid, 2-octyl-, methyl ester $$ Methyl 10-(2-octylcyclopropyl)decanoate # $$

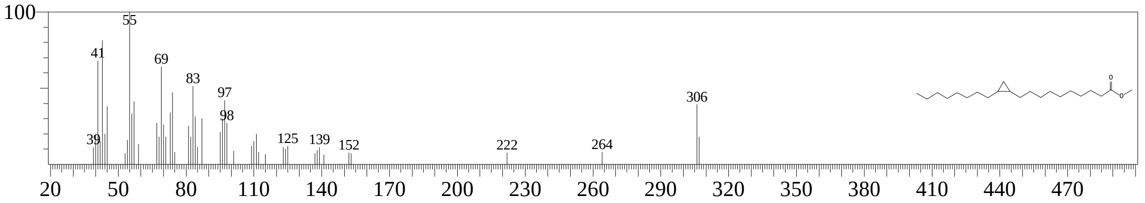

Hit#:2 Entry:98784 Library:NIST05.LIB
SI:67 Formula:C19H36O2 CAS:13481-95-3 MolWeight:296 RetIndex:2085
CompName:10-Octadecenoic acid, methyl ester $$ Methyl (10E)-10-octadecenoate # $$

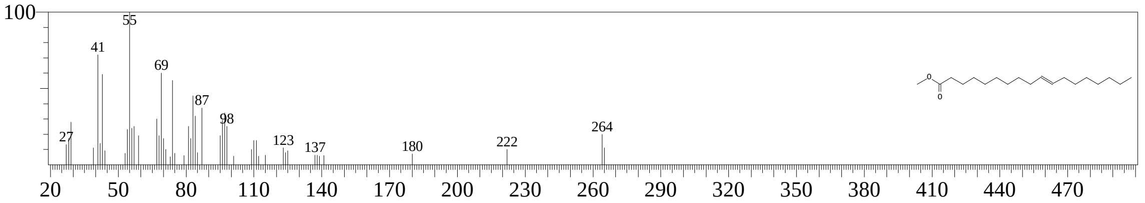

Hit#:3 Entry:98779 Library:NIST05.LIB
SI:67 Formula:C19H36O2 CAS:57396-98-2 MolWeight:296 RetIndex:2085
CompName:7-Octadecenoic acid, methyl ester $$ Methyl (7E)-7-octadecenoate # $$

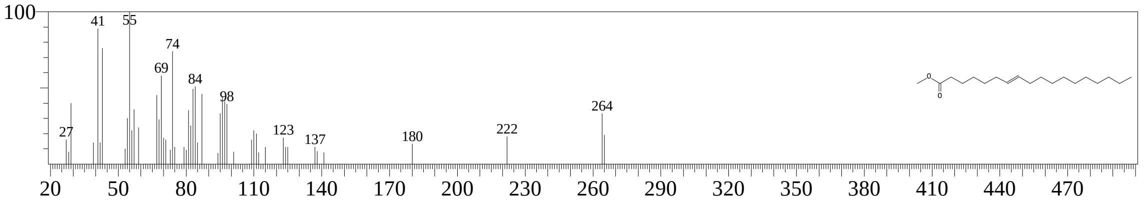

Hit#:4 Entry:98788 Library:NIST05.LIB
SI:66 Formula:C19H36O2 CAS:112-62-9 MolWeight:296 RetIndex:2085
CompName:9-Octadecenoic acid (Z)-, methyl ester $$ Oleic acid, methyl ester $$ Emery oleic acid ester 2301 $$ Methyl cis-9-octa

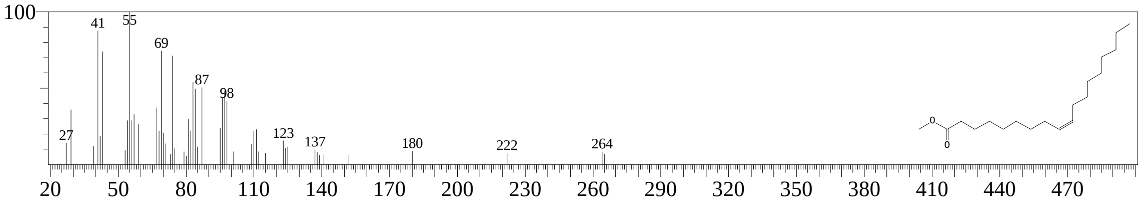

Hit#:5 Entry:82163 Library:NIST05.LIB
SI:66 Formula:C17H32O2 CAS:56875-67-3 MolWeight:268 RetIndex:1886
CompName:7-Hexadecenoic acid, methyl ester, (Z)- $$ Methyl (7E)-7-hexadecenoate # $$

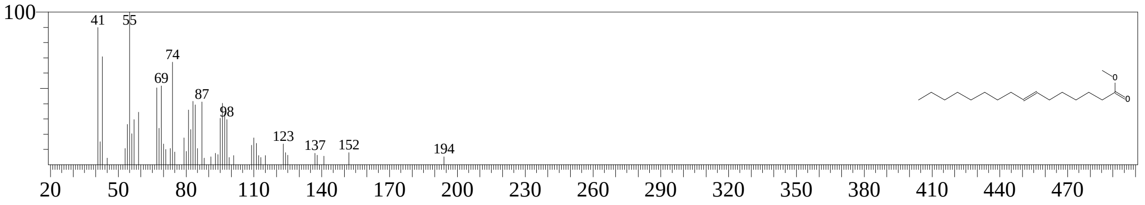

<< Target >>
Line#:22 R.Time:24.400(Scan#:2569) MassPeaks:294 BasePeak:174.05(10798)
RawMode:Averaged 24.392-24.408(2568-2570) BG Mode:Calc. from Peak

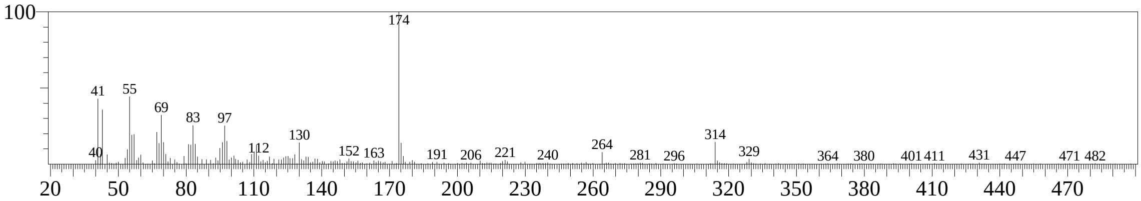

Hit#:1 Entry:65289 Library:NIST05.LIB
SI:77 Formula:C15H28O2 CAS:0-0-0 MolWeight:240 RetIndex:1813
CompName:Z-8-Methyl-9-tetradecenoic acid

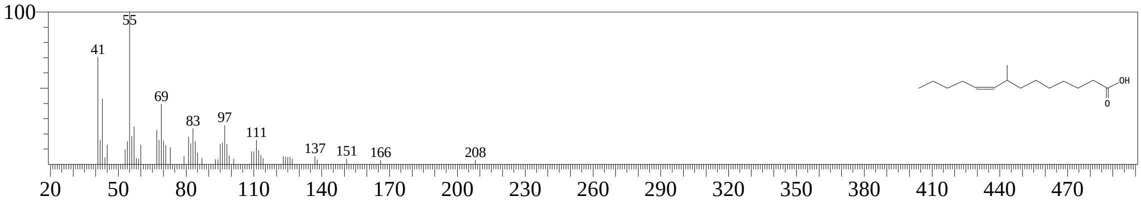

Hit#:2 Entry:22869 Library:NIST05s.LIB
SI:77 Formula:C18H34O2 CAS:112-80-1 MolWeight:282 RetIndex:2175
CompName:Oleic Acid $$ 9-Octadecenoic acid (Z)- $$ .delta.(Sup9)-cis-Oleic acid $$ cis-.delta.(Sup9)-Octadecenoic acid $$ cis-C

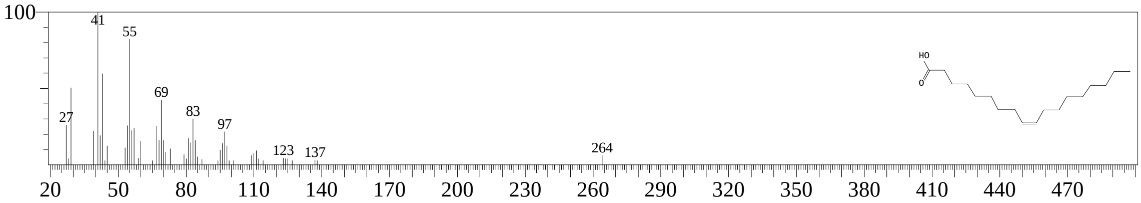

Hit#:3 Entry:21184 Library:NIST05s.LIB
SI:76 Formula:C16H30O2 CAS:2416-20-8 MolWeight:254 RetIndex:1976
CompName:Hexadecenoic acid, Z-11- $$ Z-11-Hexadecenoic acid $$ (11Z)-11-Hexadecenoic acid # $$

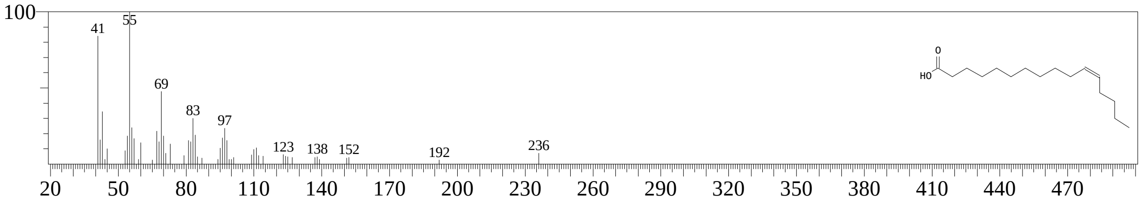

Hit#:4 Entry:73687 Library:NIST05.LIB
SI:76 Formula:C16H30O2 CAS:2416-20-8 MolWeight:254 RetIndex:1976
CompName:Hexadecenoic acid, Z-11- $$ Z-11-Hexadecenoic acid $$ (11Z)-11-Hexadecenoic acid # $$

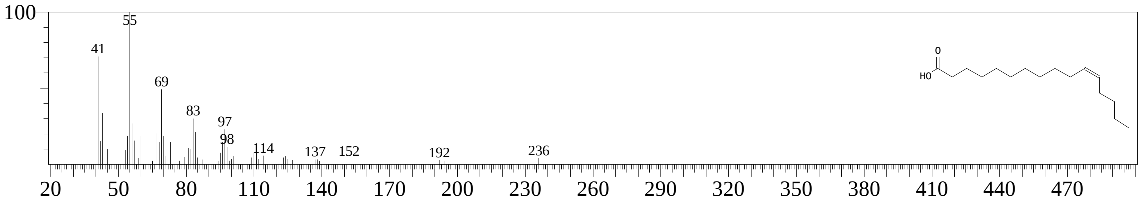

Hit#:5 Entry:57002 Library:NIST05.LIB
SI:76 Formula:C14H26O2 CAS:544-64-9 MolWeight:226 RetIndex:1777
CompName:Myristoleic acid $$ 9-Tetradecenoic acid, (Z)- $$ Z-9-Tetradecenoic acid $$ (9Z)-9-Tetradecenoic acid # $$

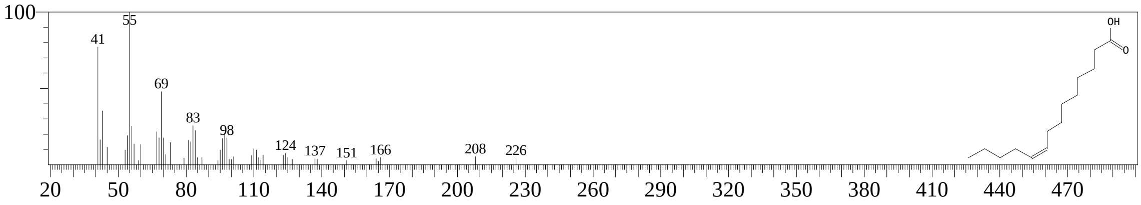

<< Target >>
Line#:23 R.Time:24.592(Scan#:2592) MassPeaks:247 BasePeak:60.00(1440)
RawMode:Averaged 24.583-24.600(2591-2593) BG Mode:Calc. from Peak

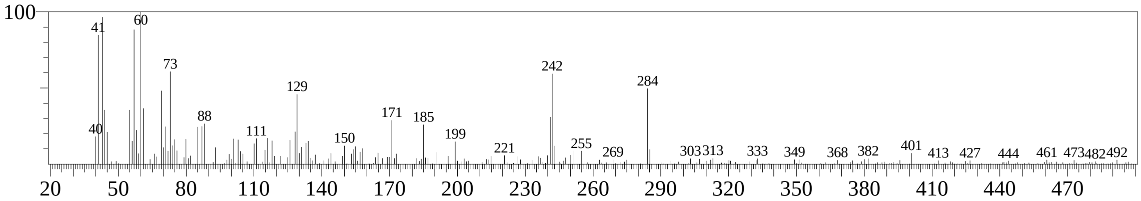

Hit#:1 Entry:66523 Library:NIST05s.LIB
SI:70 Formula:C15H30O2 CAS:1002-84-2 MolWeight:242 RetIndex:1869
CompName:Pentadecanoic acid $$ Pentadecylic acid $$ n-Pentadecanoic acid $$ n-Pentadecylic acid $$

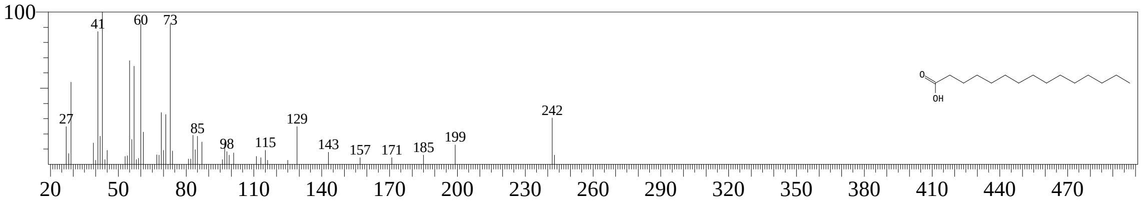

Hit#:2 Entry:22977 Library:NIST05s.LIB
SI:70 Formula:C18H36O2 CAS:57-11-4 MolWeight:284 RetIndex:2167
CompName:Octadecanoic acid $$ Stearic acid $$ n-Octadecanoic acid $$ Humko Industrene R $$ Hydrofol Acid 150 $$ Hystrene

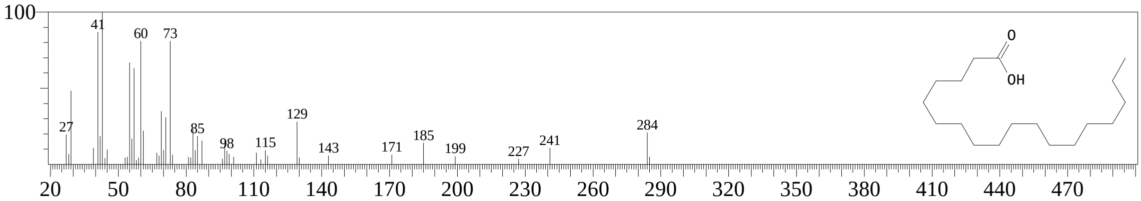

Hit#:3 Entry:91895 Library:NIST05.LIB
SI:70 Formula:C18H36O2 CAS:57-11-4 MolWeight:284 RetIndex:2167
CompName:Octadecanoic acid $$ Stearic acid $$ n-Octadecanoic acid $$ Humko Industrene R $$ Hydrofol Acid 150 $$ Hystrene

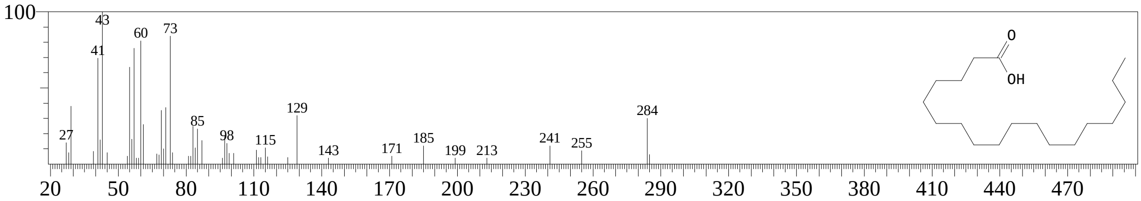

Hit#:4 Entry:136237 Library:NIST05.LIB
SI:70 Formula:C22H44O4 CAS:106-11-6 MolWeight:372 RetIndex:2694
CompName:Octadecanoic acid, 2-(2-hydroxyethoxy)ethyl ester $$ Aqua Cera $$ Atlas G 2146 $$ Cerasynt $$ Cerasynt Special $$

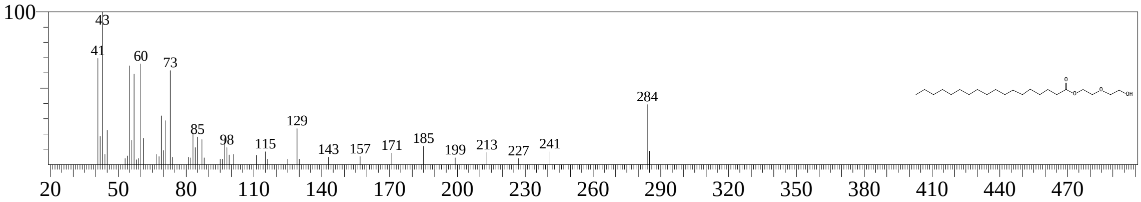

Hit#:5 Entry:22979 Library:NIST05s.LIB
SI:70 Formula:C18H36O2 CAS:57-11-4 MolWeight:284 RetIndex:2167
CompName:Octadecanoic acid $$ Stearic acid $$ n-Octadecanoic acid $$ Humko Industrene R $$ Hydrofol Acid 150 $$ Hystrene

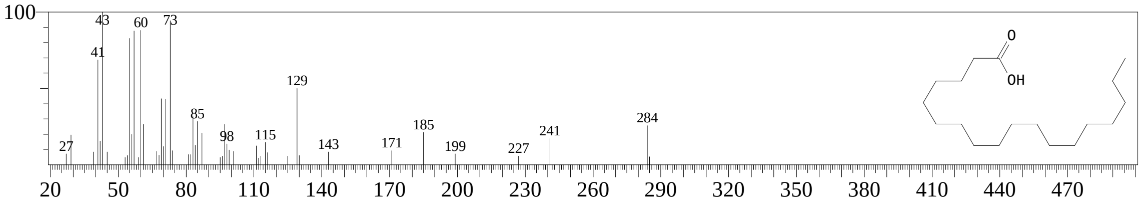

Supplement: Supplemental Information 5 [file peerj-07-6442-s005.pdf]
