# Supplementary material for: Comparative study on the composition of four different varieties of garlic
Source: PeerJ. 2019 Feb 21;7:e6442. doi: 10.7717/peerj.6442 (PMC6387757; doi:10.7717/peerj.6442)

Sample Information

Analyzed by	: Admin
Analyzed	: 2018-11-22 16:16:30
Sample Type	: Unknown
Level #	: 1
Sample Name	: 1.9ml标准品（甲醇）-1
Sample ID	:
IS Amount	: [1]=1.000
Sample Amount	: 1.000
Dilution Factor	: 1.000
Vial #	: 1
Injection Volume	: 1.000
Data File	: E:\刘春宏\1.9ml标准品（甲醇）-1.qgd
Org Data File	: E:\刘春宏\1.9ml标准品（甲醇）-1.qgd
Method File	: E:\刘春宏\2018-7-23-1.qgm
Org Method File	: E:\刘春宏\2018-7-23-1.qgm
Report File	:
Tuning File	: C:\GCMSsolution\System\Tune1_default.qgt
Modified by	: Admin
Modified	: 2018-11-22 16:42:30

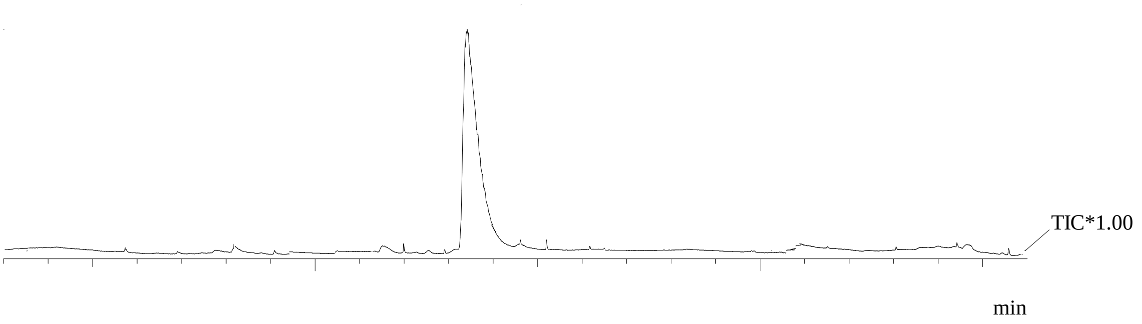

Peak Report TIC

Peak#	R.Time	I.Time	F.Time	Area	Area%	Height	Ieight%	A/H	Mark	Name
1	13.167	12.960	13.792	152246599	100.00	6250409	100.00	31.76	MI	
				152246599	100.00	6250409	100.00			

<< Target >>
Line#:2 R.Time:13.167(Scan#:1221) MassPeaks:254 BasePeak:97.00(1032661)
RawMode:Averaged 13.158-13.175(1220-1222) BG Mode:Calc. from Peak

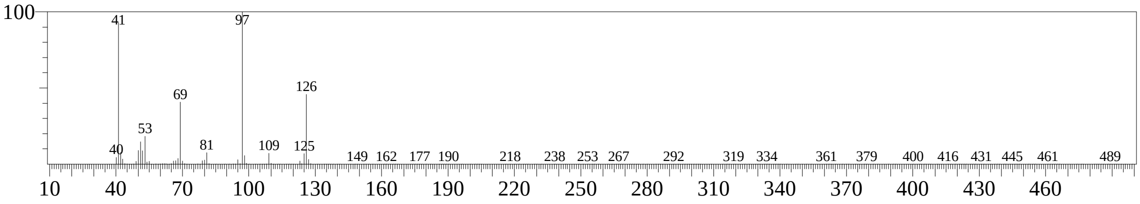

Hit#:1 Entry:6237 Library:NIST05.LIB
SI:93 Formula:C6H6O3 CAS:67-47-0 MolWeight:126 RetIndex:1163
CompName:2-Furancarboxaldehyde, 5-(hydroxymethyl)- $$ 2-Furaldehyde, 5-(hydroxymethyl)- $$ 5-Hydrxoymethylfurfural $$ Hy

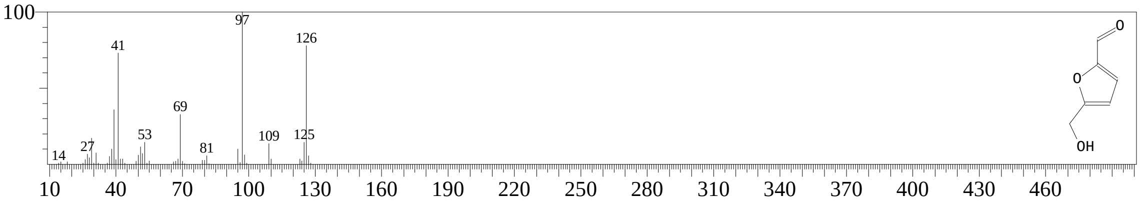

Hit#:2 Entry:4517 Library:NIST05s.LIB
SI:92 Formula:C6H6O3 CAS:67-47-0 MolWeight:126 RetIndex:1163
CompName:2-Furancarboxaldehyde, 5-(hydroxymethyl)- $$ 2-Furaldehyde, 5-(hydroxymethyl)- $$ 5-Hydrxoymethylfurfural $$ Hy

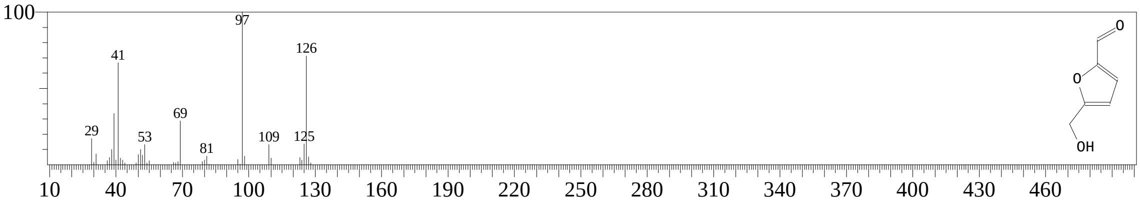

Hit#:3 Entry:6371 Library:NIST05.LIB
SI:85 Formula:C8H14O CAS:17325-90-5 MolWeight:126 RetIndex:915
CompName:4-Hexen-3-one, 4,5-dimethyl- $$ 4,5-Dimethyl-4-hexen-3-one # $$

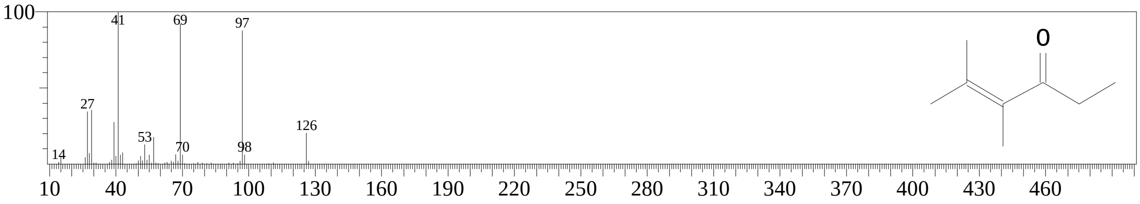

Hit#:4 Entry:6372 Library:NIST05.LIB
SI:84 Formula:C8H14O CAS:22319-31-9 MolWeight:126 RetIndex:938
CompName:4-Hepten-3-one, 4-methyl- $$ (4E)-4-Methyl-4-hepten-3-one # $$

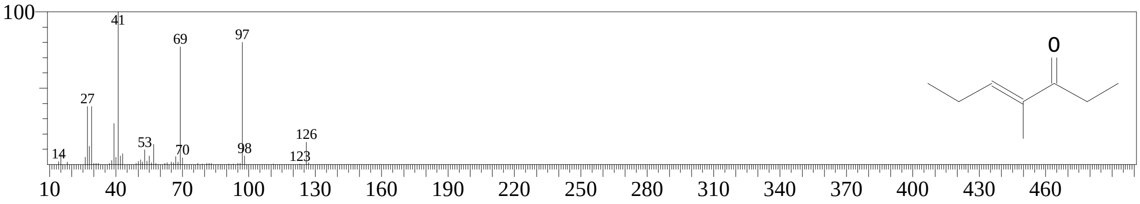

Hit#:5 Entry:6495 Library:NIST05.LIB
SI:84 Formula:C8H14O CAS:1447-26-3 MolWeight:126 RetIndex:938
CompName:4-Hepten-3-one, 5-methyl- $$ (4E)-5-Methyl-4-hepten-3-one # $$

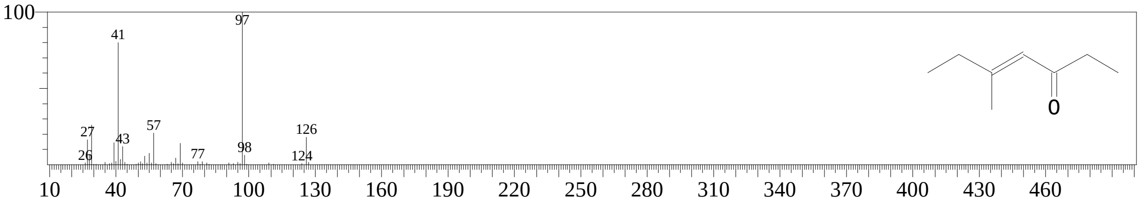

Supplement: Supplemental Information 15 [file peerj-07-6442-s015.pdf]
